# Parallel Optimization of Potency and Pharmacokinetics Leading to the Discovery of a Pyrrole Carboxamide ERK5 Kinase Domain Inhibitor

**DOI:** 10.1021/acs.jmedchem.1c01756

**Published:** 2022-04-25

**Authors:** Duncan C. Miller, Tristan Reuillon, Lauren Molyneux, Timothy Blackburn, Simon J. Cook, Noel Edwards, Jane A. Endicott, Bernard T. Golding, Roger J. Griffin, Ian Hardcastle, Suzannah J. Harnor, Amy Heptinstall, Pamela Lochhead, Mathew P. Martin, Nick C. Martin, Stephanie Myers, David R. Newell, Richard A. Noble, Nicole Phillips, Laurent Rigoreau, Huw Thomas, Julie A. Tucker, Lan-Zhen Wang, Michael J. Waring, Ai-Ching Wong, Stephen R. Wedge, Martin E. M. Noble, Celine Cano

**Affiliations:** † Cancer Research UK Newcastle Drug Discovery Unit, Newcastle University Centre for Cancer, School of Natural and Environmental Sciences, Bedson Building, Newcastle University, Newcastle upon Tyne NE1 7RU, U.K.; ‡ Cancer Research UK Newcastle Drug Discovery Unit, Newcastle University Centre for Cancer, Paul O’Gorman Building, Medical School, Framlington Place, Newcastle upon Tyne NE2 4HH, U.K.; § Signalling Laboratory, The Babraham Institute, Babraham Research Campus, Cambridge CB22 3AT, U.K.; ∥ Cancer Research UK Therapeutic Discovery Laboratories, Jonas Webb Building, Babraham Campus, Babraham, Cambridgeshire CB22 3AT, U.K.; ⊥ Cancer Research UK Therapeutic Discovery Laboratories, London Bioscience Innovation Centre, 2 Royal College Street, London NW1 0NH, U.K.

## Abstract

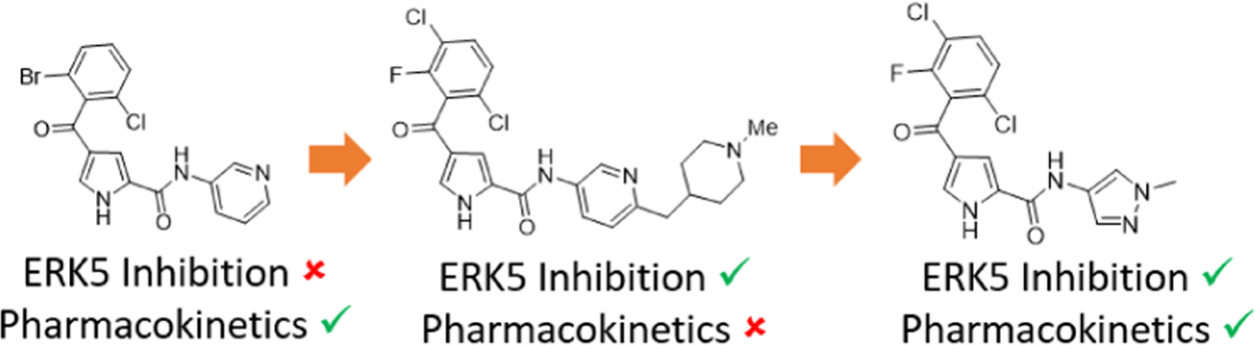

The nonclassical extracellular signal-related kinase 5 (ERK5) mitogen-activated protein kinase pathway has been implicated in increased cellular proliferation, migration, survival, and angiogenesis; hence, ERK5 inhibition may be an attractive approach for cancer treatment. However, the development of selective ERK5 inhibitors has been challenging. Previously, we described the development of a pyrrole carboxamide high-throughput screening hit into a selective, submicromolar inhibitor of ERK5 kinase activity. Improvement in the ERK5 potency was necessary for the identification of a tool ERK5 inhibitor for target validation studies. Herein, we describe the optimization of this series to identify nanomolar pyrrole carboxamide inhibitors of ERK5 incorporating a basic center, which suffered from poor oral bioavailability. Parallel optimization of potency and *in vitro* pharmacokinetic parameters led to the identification of a nonbasic pyrazole analogue with an optimal balance of ERK5 inhibition and oral exposure.

## Introduction

Extracellular signal-regulated kinase 5 (ERK5) is a member of the mitogen-activated protein kinase (MAPK) family, which includes ERK1/2, JNK1/2/3, and p38. Activation of the nonclassical MEK5–ERK5 MAPK pathway is associated with increased cellular proliferation, migration, survival, and angiogenesis.^1−4^ In approximately 50% of hepatocellular carcinomas (HCCs), the *MAPK7* gene encoding for ERK5 is amplified.^5^ ERK5 expression is also upregulated in breast and prostate cancers.^6,7^ Patients with high levels of ERK5 have a median disease-free survival time of 14 months compared with that of 34 months for patients with low expression.^7^ Elevated cytoplasmic and nuclear levels of ERK5 serve as independent prognostic markers for advanced prostate cancer, with nuclear ERK5 expression present only in malignant cells.^6^ Phosphorylated ERK5 associates with, phosphorylates, and activates a number of downstream transcription factors, such as the myocyte enhancer factor (MEF) family, c-Myc, RSK, c-Fos, c-Jun, and Sap1a,^8^ which are involved in the modulation of apoptosis. ERK5 has also been shown to play a role in cellular invasion and metastatic spread, affecting cell migration and attachment to the extracellular matrix.^9^ ERK5 activation has also been implicated as a potential resistance mechanism to therapeutics targeting the RAF–MEK1/2–ERK1/2 pathway.^10^ Selective ERK5 kinase inhibitors will therefore be useful in elucidating the role of this signaling protein in cancer and determining whether they represent potential therapeutics.

**Figure 1:**
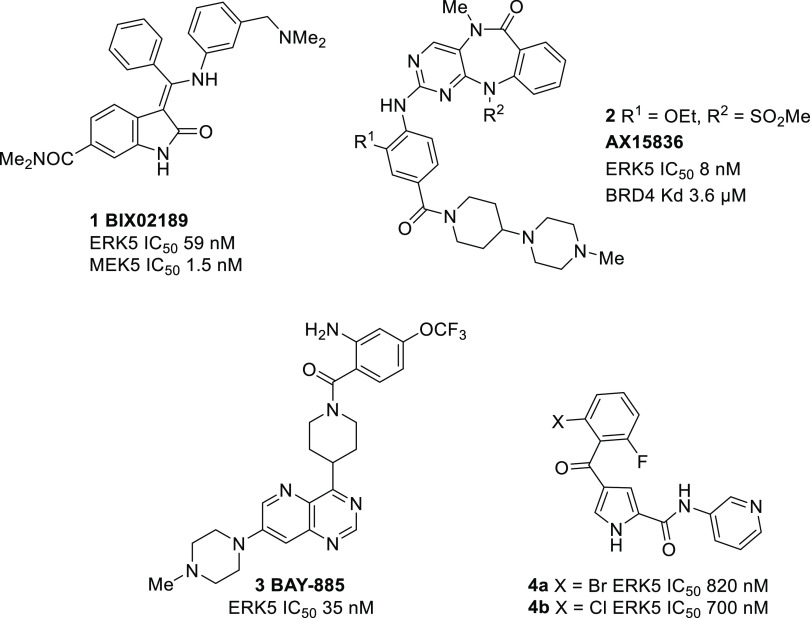
Published ERK5 inhibitors.

There has been significant interest in developing ERK5 inhibitors to interrogate its role in cancer.^11,12^ Oxindole BIX02189 (Figure 1; **1**) was identified as a dual ERK5–MEK5 inhibitor.^13^ Subsequently, AX15836 (**2**), a potent and selective ERK5 inhibitor from the pyrimidodiazepinone series, was reported as a useful ERK5 probe^14^ and BAY-885 (**3**) was disclosed as a structurally differentiated inhibitor.^15^

**Figure 2:**
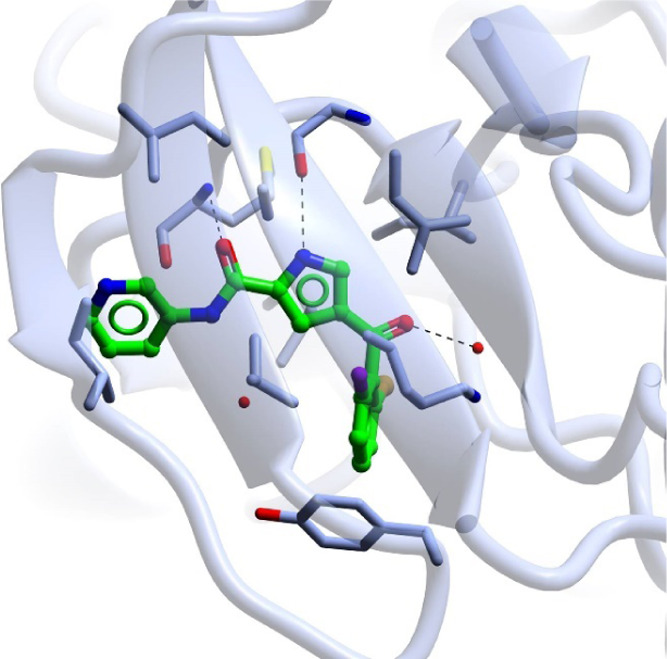
Crystal structure of the ERK5–**4a** complex determined at a 2.4 Å resolution (PDB ID: 5O7I). H-bonds are shown as dashed lines.

We have described the identification of pyrrole carboxamide-based ERK5 inhibitors (**4a**,**b**) with submicromolar potency, excellent kinase selectivity, and encouraging activity in a mouse tumor xenograft model.^16^ To identify a tool compound from this series, improvement in primary ERK5 inhibitory potency while maintaining the attractive pharmacokinetic properties and selectivity profile was required. The X-ray crystal structure of **4a** bound to the adenosine triphosphate (ATP)-binding site of ERK5 (Figure 2) indicates that the ketone and amide carbonyl groups lie coplanar with the pyrrole ring, with the 2,6-disubstituted phenyl ring orthogonal to this plane, occupying a hydrophobic pocket. The pyridyl amide projects toward the solvent-exposed region of the binding pocket, and optimization of this substituent was investigated as a means to improve ERK5 inhibition.

## Results and Discussion

### Chemistry

**Scheme 1:**
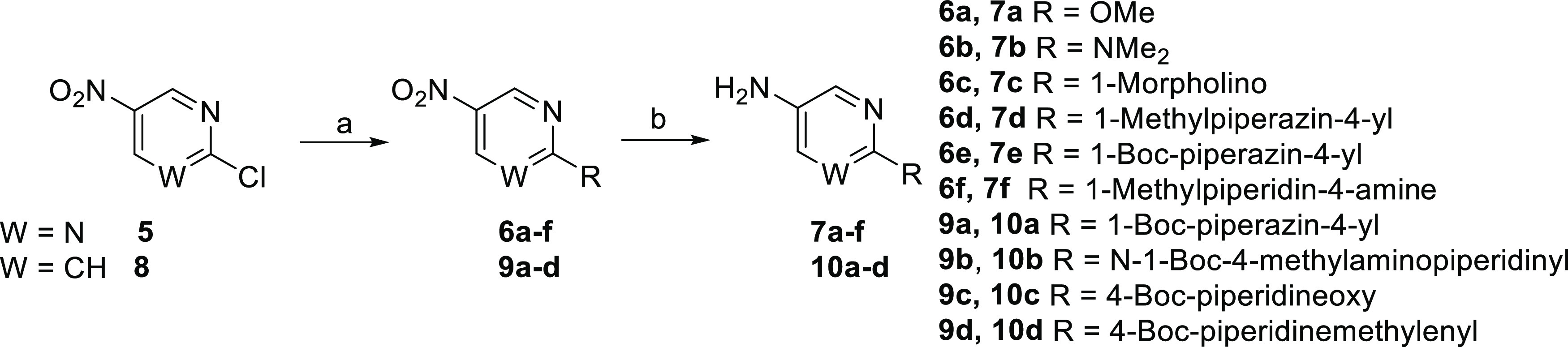
Synthesis of 2-Substituted Aminopyrimidines **7a**–**f** and Aminopyridines **10a**–**d** Reagents and conditions: (a) method 1: W = N, R = R_1_R_2_N: Et_3_N, R_1_R_2_NH, tetrahydrofuran (THF), room temperature (rt) 18 h, 68–87%; method 2: W = N, R = OMe: Na, MeOH, 65 °C, 1 h, 59%; method 3: W = CH, R = R_1_R_2_N: K_2_CO_3_, R_1_R_2_NH, THF, 80 °C, 0.5–3 h; 78–97%; method 4: (i) *tert*-butyl 4-methylidenepiperidine-1-carboxylate, 9-BBN (0.5 M in THF), 67 °C, 3 h; (ii) **5**, K_2_CO_3_, PdCl_2_dppf, dimethylformamide (DMF)/H_2_O (10:1), 60 °C, 18 h, 40%; (b) H_2_, 10% Pd/C, MeOH, CH_2_Cl_2_, 92–100%.

5-Pyridyl and 5-pyrimidylamines substituted at the 2-position with *O* or *NH* linkers (**7a**–**f** and **10a**–**c**) were synthesized from 2-chloro-5-nitro-pyrimidine (**5**) or 2-chloro-5-nitropyridine (**8**), respectively, by nucleophilic aromatic substitution with an appropriate amine or alcohol, followed by palladium-catalyzed hydrogenation of the nitro group (Scheme 1). Methylene-linked piperidine **10d** was prepared by *in situ* hydroboration of *tert*-butyl 4-methylidenepiperidine-1-carboxylate followed by palladium-catalyzed cross-coupling (Scheme 1; method 4).

**Scheme 2:**
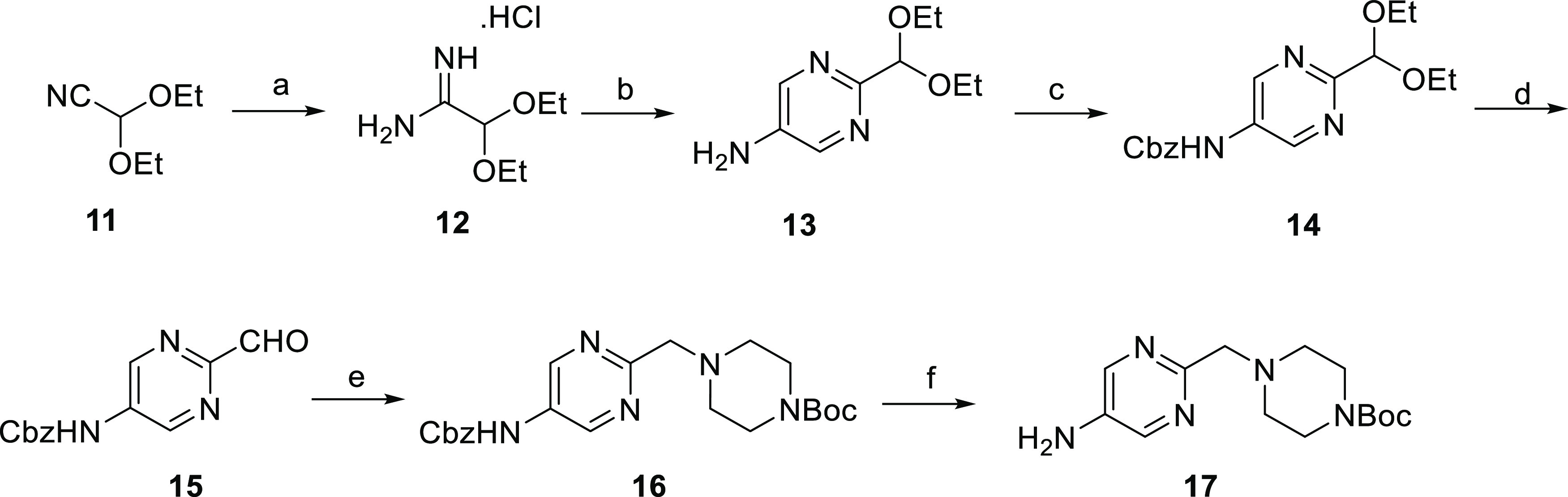
Synthesis of *tert*-Butyl 4-((5-Aminopyrimidin-2-yl)methyl)piperazine-1-carboxylate **17** Reagents and conditions: (a) (i) NaOMe, MeOH, RT, 16 h; (ii) NH_4_Cl, MeOH, RT, 18 h, 89%; (b) (i) *N*-(3-(dimethylamino)-2-[[(dimethylamino)methylene]amino]prop-2-en-1-ylidene)-*N*-methylmethanaminium hydrogen dihexafluorophosphate, NaOMe (1 M in MeOH), EtOH, 78 °C, 2.5 h; (ii) 5% aq K_2_CO_3_, dioxane, 100 °C, 18 h, 49% (over two steps); (c) benzyl chloroformate, K_2_CO_3_, THF/H_2_O (1:1), RT, 24 h, 80%; (d) HCl (1 M aq), MeCN, RT, 8 h, 89%; (e) (i) *tert*-butyl piperazine-1-carboxylate, MgSO_4_, 2,2,2-trifluoroethanol, 1 h, RT; (ii) NaBH_4_, 2,2,2-trifluoroethanol, 0 °C to RT, 1 h, 42%; and (f) H_2_, 10% Pd/C, EtOAc, RT, 24 h, 99%.

A pyrimidine ring synthesis was employed in the synthesis of amine **17** from diethoxyacetonitrile **10** (Scheme 2). Protection of amine **13** as benzyl carbamate **14**, hydrolysis of the diethyl acetal, and reductive amination with 1-Boc-piperazine gave **16**, which was deprotected to give amine **17**.

**Scheme 3:**
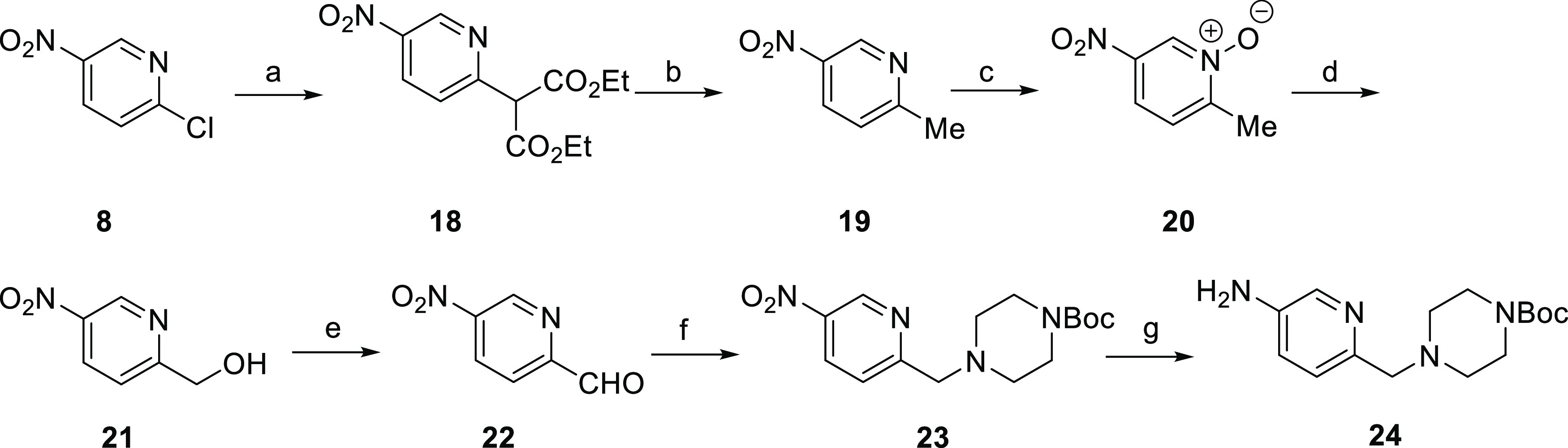
Synthesis of *tert*-Butyl 4-((5-Aminopyridin-2-yl)methyl)piperazine-1-carboxylate **24** Reagents and conditions: (a) (i) NaH (60% dispersion in mineral oil), diethyl malonate, THF, 0 °C to RT, 1 h; (ii) 2-chloro-5-nitropyridine, 0 °C to RT, 20 h, 64%; (b) 20% aq H_2_SO_4_, 100 °C, 2 h, 95%; (c) *m-*CPBA (74%), dichloromethane (DCM), 0 °C to RT, 16 h, 96%; (d) (i) trifluoroacetic anhydride (TFAA), DCM, 0 °C to RT, 16 h; (ii) MeOH, 0 °C to RT, 8 h, 50%; (e) MnO_2_, DCM, RT, 16 h, 61%; (f) (i) *tert*-butyl piperazine-1-carboxylate, MgSO_4_, 2,2,2-trifluoroethanol, 1 h, 38 °C; (ii) NaBH_4_, 2,2,2-trifluoroethanol, 0 °C to RT, 1 h, 51%; and (g) H_2_, 10% Pd/C, MeOH/THF (1:1), 40 °C, 8 h, 95%.

**Scheme 4:**
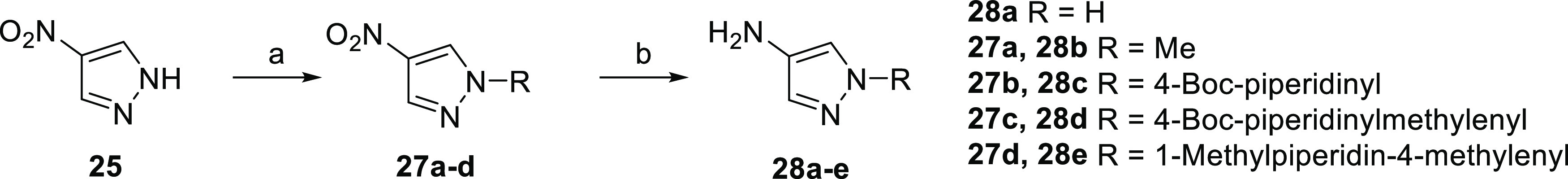
Synthesis of Substituted Aminopyrazoles **28a**–**e** Reagents and conditions: (a) PPh_3_, diethyl azodicarboxylate (DEAD), THF, R-OH, rt 18 h, 34–82% and (b) H_2_, 10% Pd/C, MeOH, 95–100%.

For the synthesis of substituted 2-pyridylmethylpiperazine **24**, the nucleophilic aromatic substitution of **8** with the sodium salt of diethyl malonate followed by double decarboxylation under acidic conditions gave 2-methyl-5-nitropyridine **19** (Scheme 3). *N*-Oxidation and subsequent rearrangement provided alcohol **21**, which was converted to aldehyde **22**. Reductive amination with 1-Boc-piperazine followed by reduction of the nitro group gave amine **24**. Substituted pyrazolamines were synthesized by Mitsunobu alkylation of 4-nitropyrazole, followed by nitro reduction (Scheme 4).

**Scheme 5:**
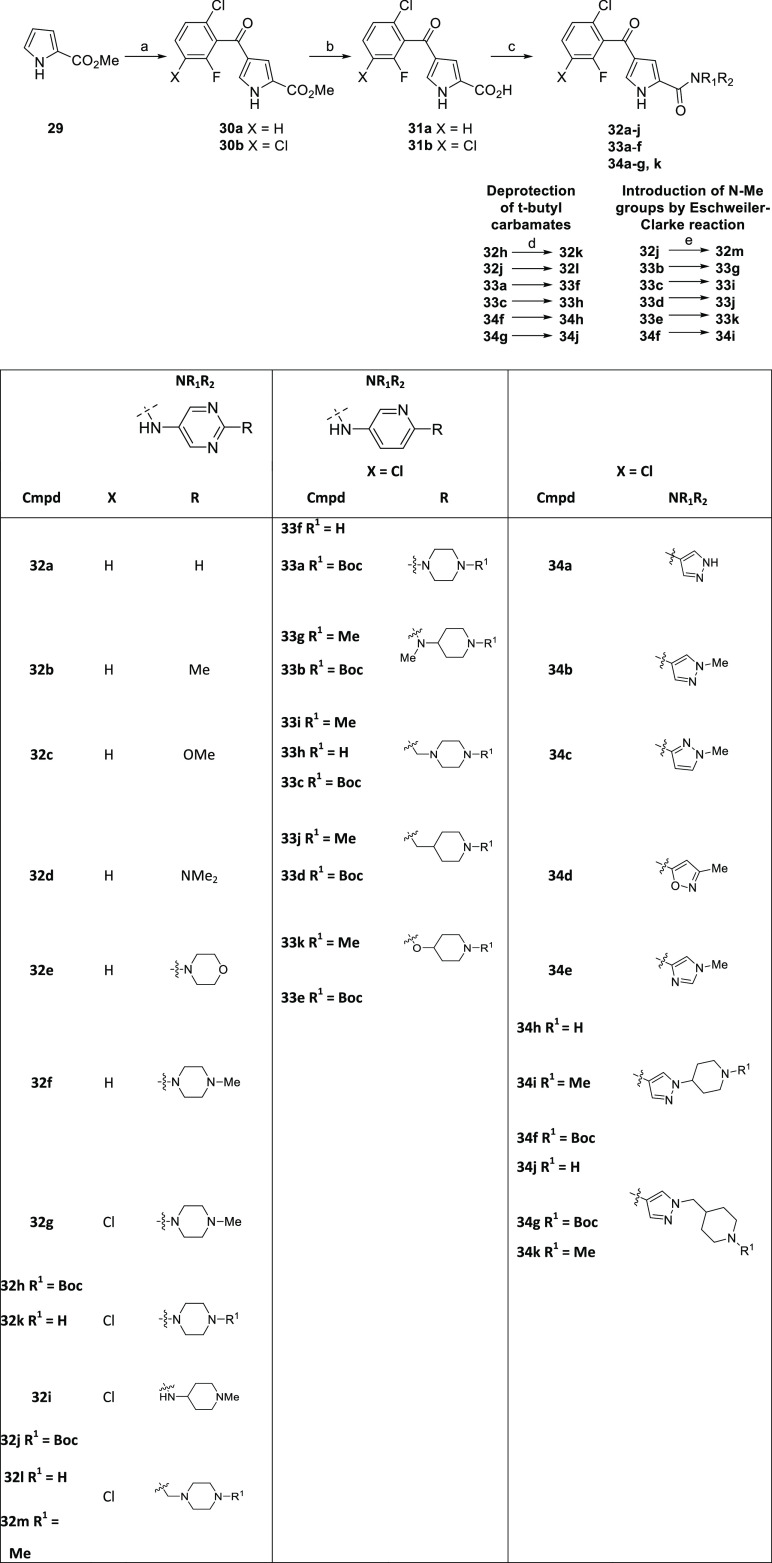
Synthesis of Pyrrole Carboxamides **32a**–**m**, **33a**–**k**, and **34a**–**k** Reagents and conditions: (a) ArCOCl, AlCl_3_, 0 °C to RT, 20 h, 89–92%; (b) LiOH, H_2_O/THF, 67 °C, 18 h, 95–99%; (c) method 1: amine, cyanuric fluoride, pyridine, MeCN, rt, 18 h, 34–76%; method 2: amine, PCl_3_, MeCN, 155 °C, 5 min, 24–79%; method 3: amine, PyBrOP, pyridine, MeCN, rt, 2 h. 39%; method 4: 2-chloro-1-methylpyridinium iodide, NEt_3_, DCM, rt, 18 h, 28–49%; (d) TFA, Et_3_SiH, DCM, rt, 2 h; and (e) HCO_2_H, HCHO, 100 °C, 3 h.

Substituted 4-benzoyl-1*H*-pyrrole-2-carboxylic acids **31a** and **31b** were synthesized according to Scheme 5. Amines were coupled to the appropriate pyrrole carboxylic acid using cyanuric fluoride, PCl_3_, or 2-chloro-1-methylpyridinium iodide as activating agents to give targets **32a**–**k** and **33a**–**f** and **34a**–**i**. *N*-Boc-protected amines were either deprotected under standard acidic conditions (DCM, trifluoroacetic acid, Et_3_SiH, RT, 2 h) or subjected to direct Eschweiler–Clarke *N*-methylation [formic acid, formaldehyde (37% wt in water), 95 °C, 3 h].

Replacement of the 3-pyridyl amide of **4a** with a 4-pyrimidyl amide (**32a**) maintained ERK5 inhibitory activity, enabling rapid diversification at the 2-position of the pyrimidine ring (Table 1). Introduction of small alkyl and heteroalkyl substituents (**32b**–**d**) and morpholine (**32e**) at this position led to a reduction in ERK5 potency. However, *N-*methylpiperazine **32f** exhibited a 6-fold increase in potency relative to **32a**. The ERK5–**4a** crystal structure indicated the presence of a small hydrophobic void adjacent to the phenyl ketone, which was targeted through the introduction of a chloro group into the phenyl ketone and resulted in a further improvement in ERK5 inhibition (**32g**). *NH*-Piperazine **32k** was also tolerated, and pyridinylpiperazine (**33f**) proved to be equipotent with its pyrimidinyl analogue. **32g** was found to be rapidly metabolized in mouse liver microsomes and inhibited the hERG cardiac ion channel. *NH*-Piperazines **32k** and **33f** had superior *in vitro* metabolism and hERG inhibition profiles. In a caco-2 cell permeability assay, both **32k** and **33a** exhibited poor permeability and high efflux ratios.

**Table 1:**
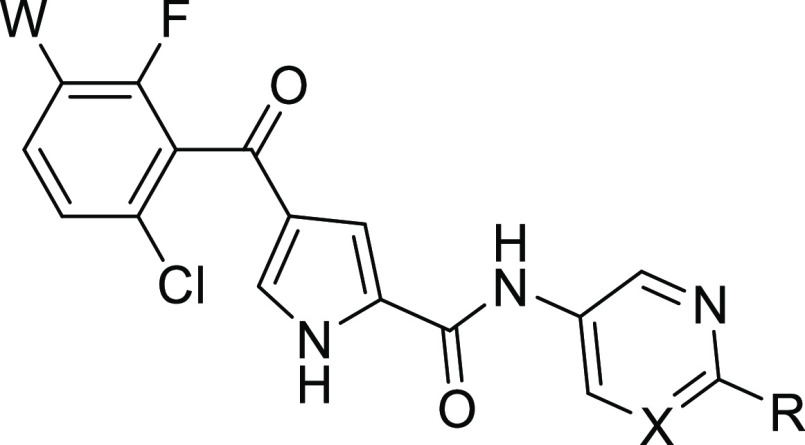

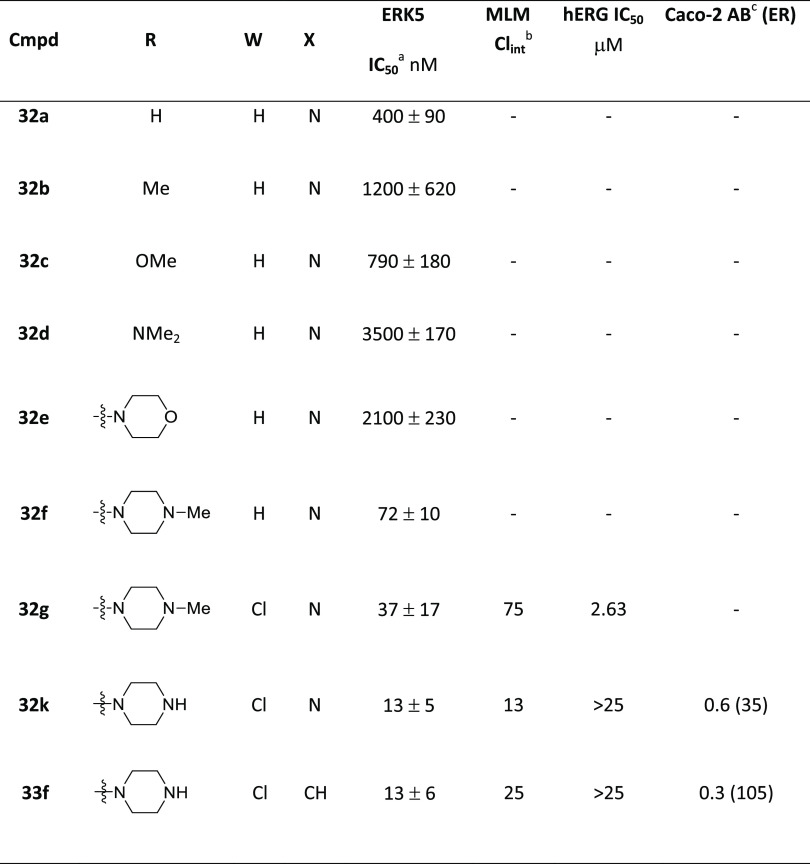
ERK5 Inhibitory Activity of 2-Substituted Pyrimidine Amides

a ERK5 IC_50_’s determined using an IMAP FP progressive binding system kit (Molecular Devices #R8127).

b μL/min/mg.

c *P*_app_ 10^–6^ cm·s^–1^; - = not determined.

**Figure 3:**
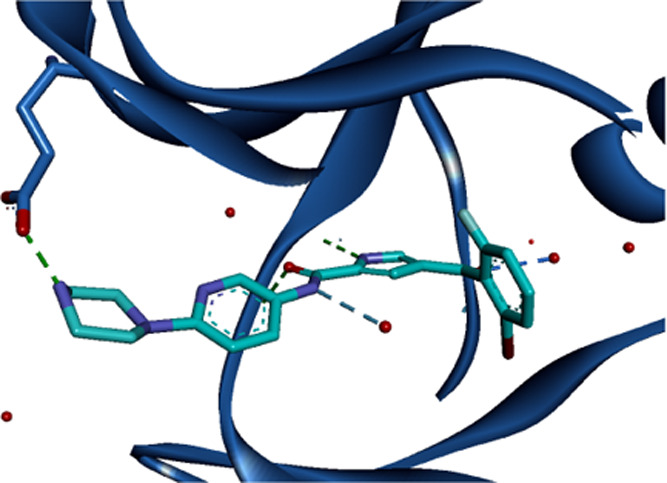
Modeled structure of **33f** in the ATP-binding site of ERK5, demonstrating the close proximity of the piperazine basic center with the carboxylate group from the side chain of Glu_59_.

Modeling of the binding pose of **33f** was performed by manual ligand building from the crystal structure of the complex of ERK5 and 4a (PDB ID: 5O71). This suggested that the basic center of the piperazine analogues may interact with the acidic side chain of Glu_59_ in the mouth of the binding pocket (Figure 3). It was speculated that varying the position and basicity of the ionizable center may allow the optimization of potency and ADME properties.

Structure–activity relationships for the position of the basic center were explored through the introduction of a spacer between the heteroaromatic and aliphatic rings of the amide substituent. Compounds incorporating amine, methylene, and ether linkers all retained low nanomolar ERK5 inhibition in both the pyrimidyl (**32**) and pyridyl (**33**) amide series, yielding potent ERK5 inhibitors with improved microsomal clearance (Table 2). Cell-based inhibition of ERK5 autophosphorylation was assessed in HeLa cells using Western blot densitometry of phospho-ERK, with all compounds exhibiting good cellular ERK5 inhibition. However, modulation of efflux pump recognition in the caco-2 permeability assay through variation of the position, linkage, and p*K*_a_ of the basic center achieved limited success, with **33k** having moderate flux and exhibiting an efflux ratio of less than 10.

**Table 2:**
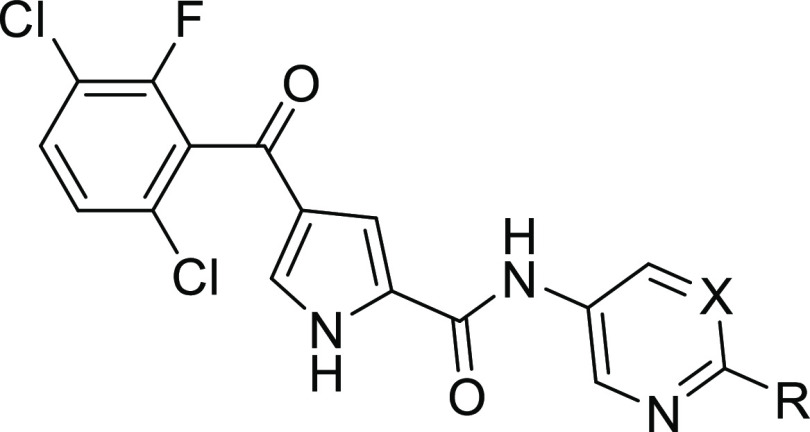

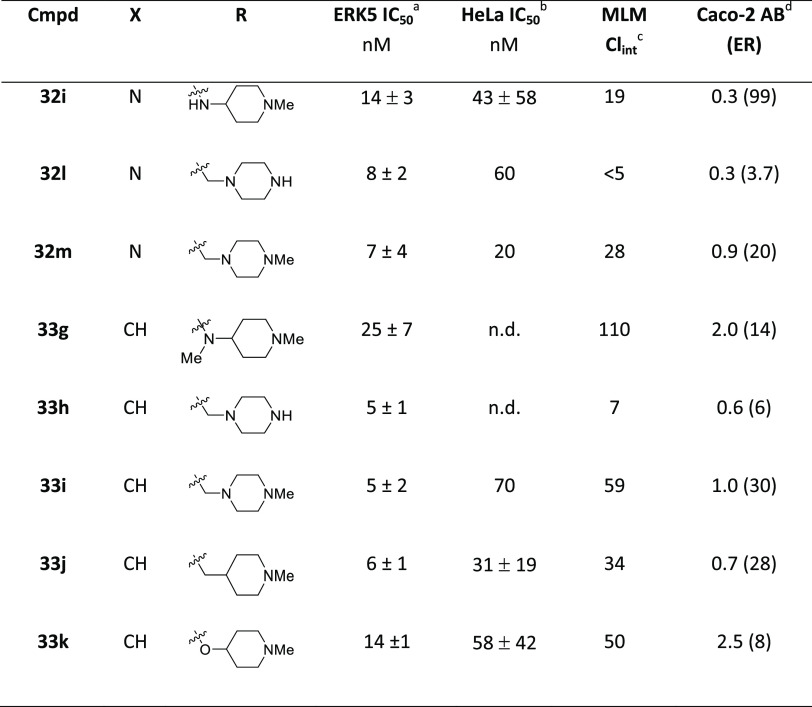
ERK5 Inhibitory Activity and *In Vitro* ADME Data for **32i**–**k** and **33b**–**f**

a ERK5 IC_50_’s determined using an IMAP FP progressive binding system kit (Molecular Devices #R8127).

b IC_50_ determined by phospho-ERK5 Western blot densitometry in HeLa cells (1 h incubation with compounds).

c μL/min/mg protein.

d *P*_app_ 10^–6^ cm·s^–1^.

In a mouse PK study, compounds **33j** and **33k** had low clearance, with terminal elimination half-lives of 263 and 80 min, respectively (Table 3). Oral bioavailability was low consistent with the *in vitro* permeability data.

**Table 3:**
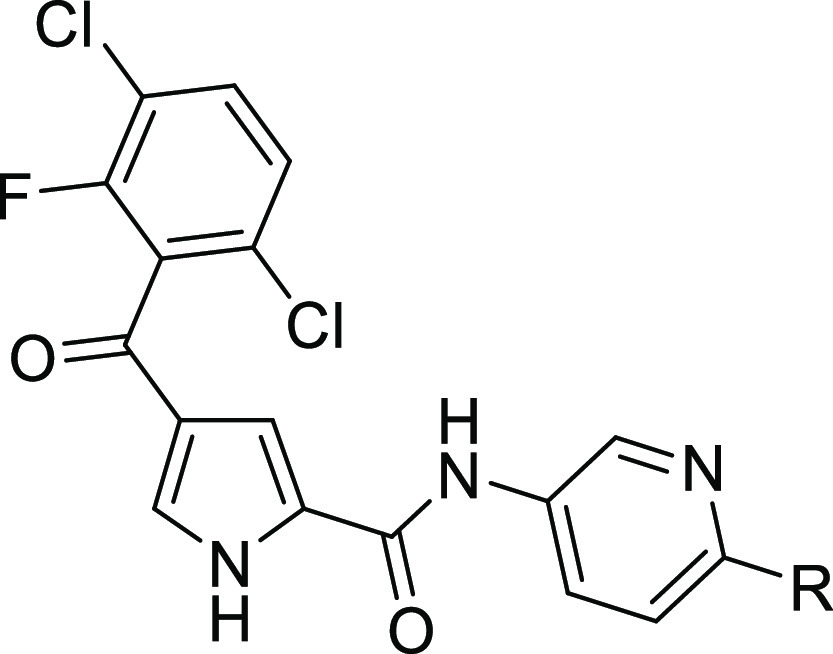

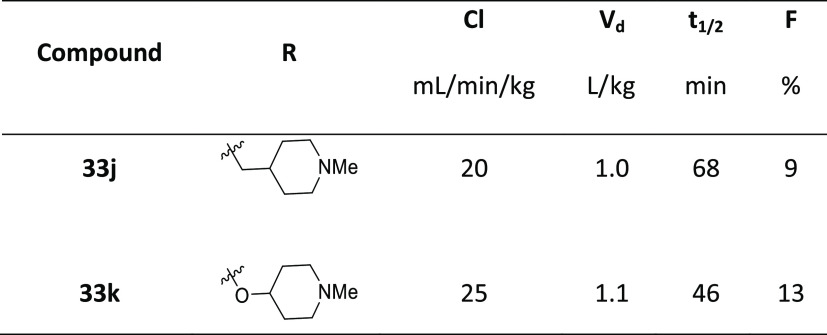
*In Vivo* Mouse Pharmacokinetic Parameters for Selected Compounds^a^

a Dose 10 mg/kg i.v. and p.o.

Five-membered heteroaromatic amides were explored as replacements for the pyridine and pyrimidine amides to determine whether efflux pump recognition could be reduced through subtle changes in size, geometry, and polarity of the amide group. Of the 5-membered heterocycles studied, only 4-pyrazole amides retained low nanomolar ERK5 inhibition (Table 4). *NH*-Pyrazole **34a** was rapidly metabolized in mouse liver microsomes and again suffered from efflux in the caco-2 assay. However, *N*-methylpyrazole **34b** had good permeability and low efflux and was also stable in mouse liver microsomes. Its isomer **34c** and other similar 5-membered heterocyclic amides (**34d, 34e**) incorporating heteroatoms adjacent to the amide linker were significantly less potent.

**Table 4:**
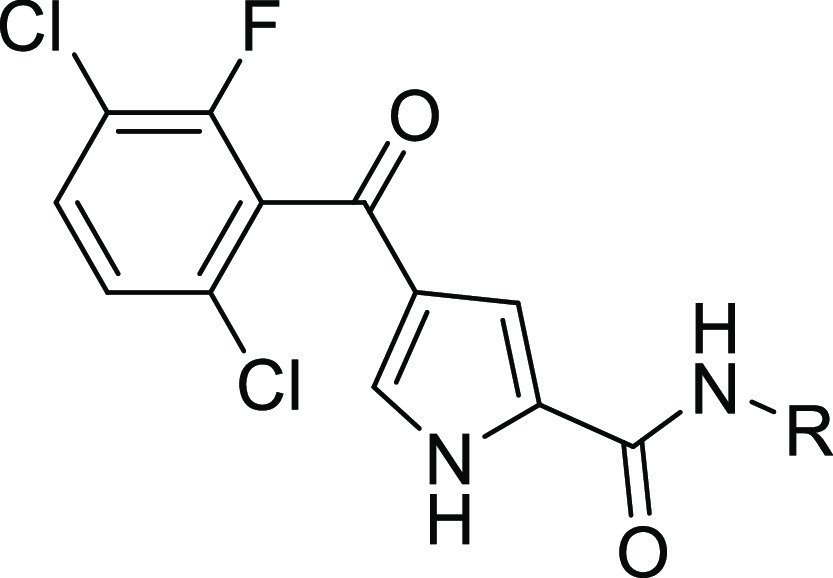

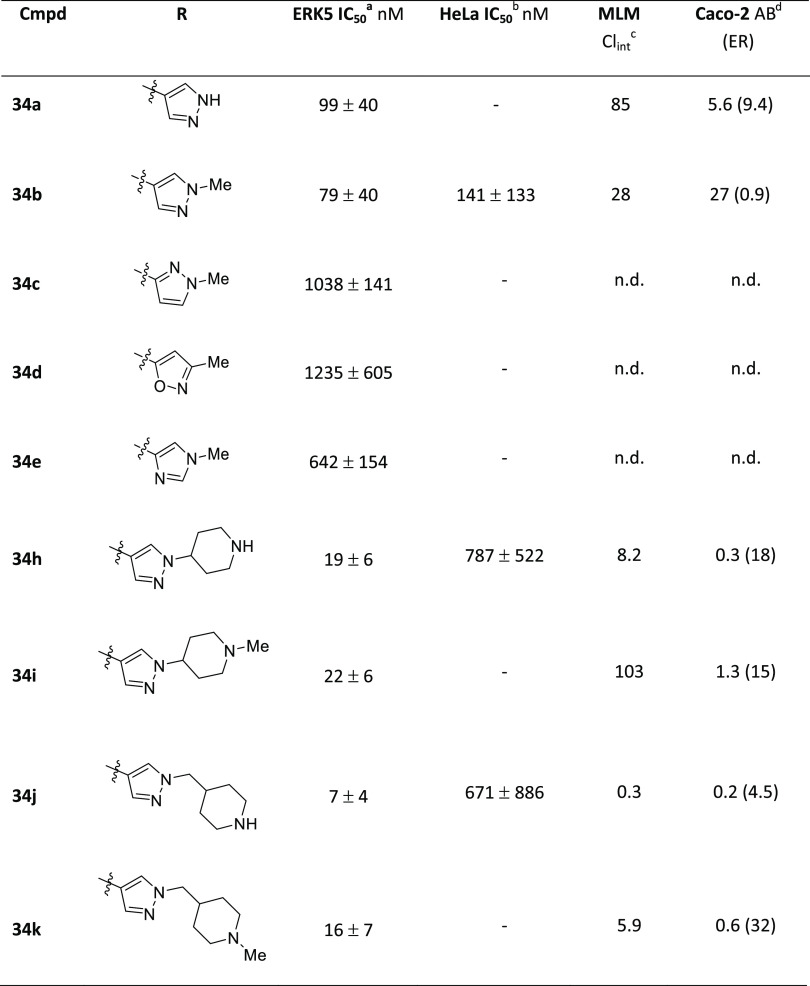
Structures and ERK5 Inhibitory Activity Data for Compounds **34a**–**k**

a ERK5 IC_50_’s determined using an IMAP FP progressive binding system kit (Molecular Devices #R8127).

b IC_50_ determined by phospho-ERK5 Western blot densitometry in HeLa cells (1 h incubation with compounds).

c μL/min/mg protein.

d *P*_app_ 10^–6^ cm·s^–1^.

The pyrazole amides offer an alternative attachment point for the incorporation of a basic center to target the proposed interaction with Glu_59_. Analogues **34h**–**k** were prepared and provided comparable ERK5 inhibition to their pyrimidyl and pyridyl amide analogues. **34j** had the lowest ERK5 IC_50_ in the binding assay and exhibited good mouse microsomal stability and a caco-2 efflux ratio < 5, a significant reduction in the efflux ratio compared to its pyridyl analogue **33j** (ER = 28). However, intrinsic flux remained low. Inhibition of ERK5 autophosphorylation was assessed in HeLa cells. Translation of ERK5 inhibition from the cell-free to the cell-based assay was variable for compounds with poor membrane permeability, with basic pyrazoles **34h** and **34j** exhibiting 40- and 120-fold lower potencies in the cell-based assay compared to those in the binding assay. In contrast, the cell IC_50_ of the more membrane-permeable neutral pyrazole **34b** was just 3-fold lower than in the binding assay.

Pyrazole amide **34b** had low clearance and an oral bioavailability of 42% in the mouse (Table 5). Compound **34b** was selective against the closely related MAP3K, p38 (IC_50_ > 30 μM). No inhibition or the closely related kinases ERK1 and ERK2 and JNKs 1, 2, and 3 were observed in a kinase panel screen, and **34b** had an IC_50_ > 20 μM against BRD4, in contrast to some of the earlier reported ERK5 inhibitors.^14^ Examination of 394 nonmutant kinases in competition binding assays (DiscoverX KINOMEscan) revealed **34b** to inhibit 38 kinases by ≥90% at a concentration of 10 μM (Supporting Information, Table S1). *K*_d_s were determined for 10 of these kinases using this assay platform (CSF1R *K*_d_ 46 nM, DCLK1 *K*_d_ 61 nM, MAPK7 *K*_d_ 180 nM, LRRK2 *K*_d_ 220 nM, AURKA *K*_d_ 290 nM, FGFR1 *K*_d_ 380 nM, KIT *K*_d_ 420 nM, ABL1 *K*_d_ 1.2 μM, JAK3 *K*_d_ 1.3 μM, and MEK5 *K*_d_ 2.8 μM). Thus, **34b** represents a structurally distinct ERK5 inhibitor chemotype, which is a useful addition to the toolkit for interrogating ERK5 signaling. However, the selectivity data should be taken into consideration in biological studies, particularly *in vivo* where activity against CSF1R and FGFR1 activities could influence host responses (e.g., inflammation or angiogenesis).

**Table 5:** *In Vivo* Pharmacokinetic Parameters for **34b**^a^

cmpd	Cl (mL/min/kg)	*V*_d_ (L/kg)	*t*_1/2_ (min)	*F* (%)
**34b**	14	0.6	80	42

a *In vivo* studies were performed at a dose of 10 mg/kg i.v. and 10 mg/kg p.o. in mouse.

**Figure 4:**
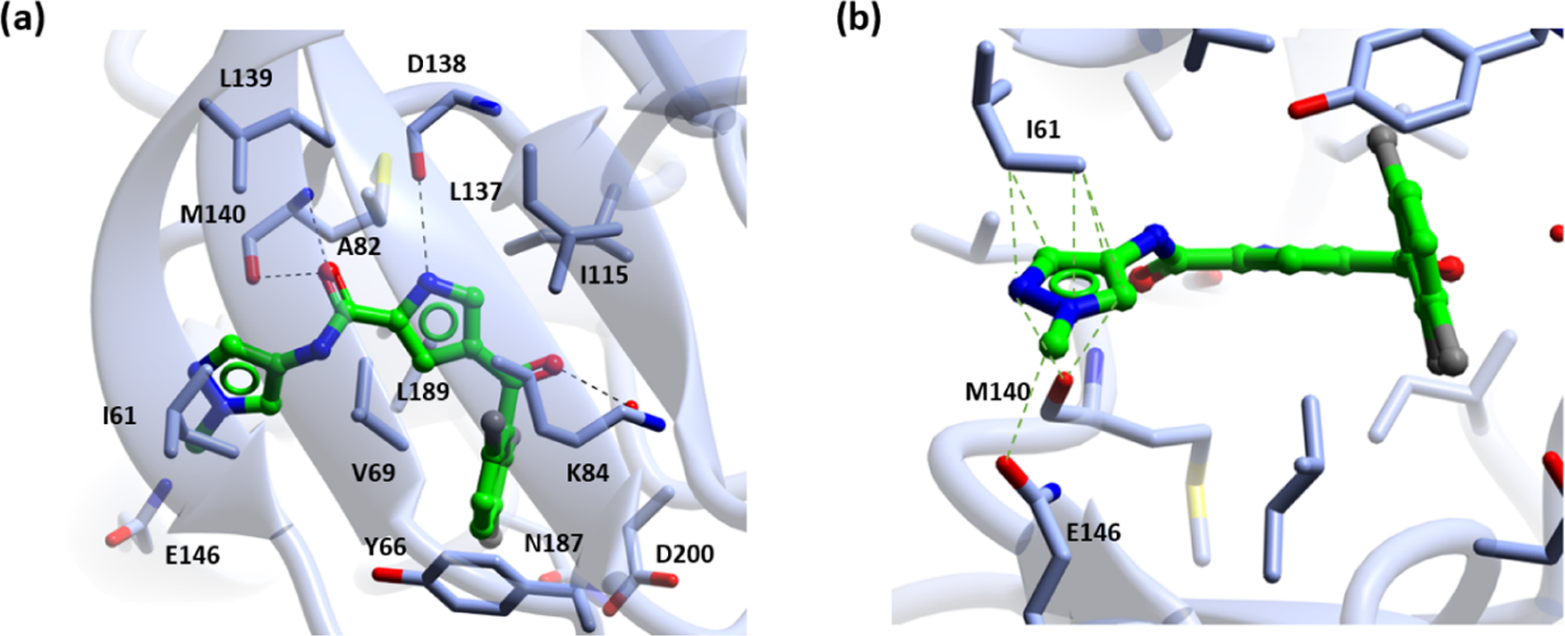
Crystal structure of the ERK5–**34b** complex determined at 2.75 Å (PDB: 7PUS). (a) Hydrogen-bonding interactions of the pyrrole NH and amide carbonyl to the hinge region of ERK5. (b) Interaction of the pyrazole with the side chains of I61, E146, and the backbone of M140.

The structure of **34b** bound to ERK5 was solved to a resolution of 2.75 Å, confirming that the binding mode was maintained, with the pyrrole carboxamide forming a bidentate interaction with the hinge region of the ATP-binding site (Figure 4a). The methylpyrrole amide lies in a small channel at the mouth of the binding pocket, lying between the side chain of E146 and the backbone of M140 and the lipophilic side chain on I61 (Figure 4b). The halogenated phenyl ring adopts a conformation orthogonal to the plane of the pyrrole ketone.

**Figure 5:**
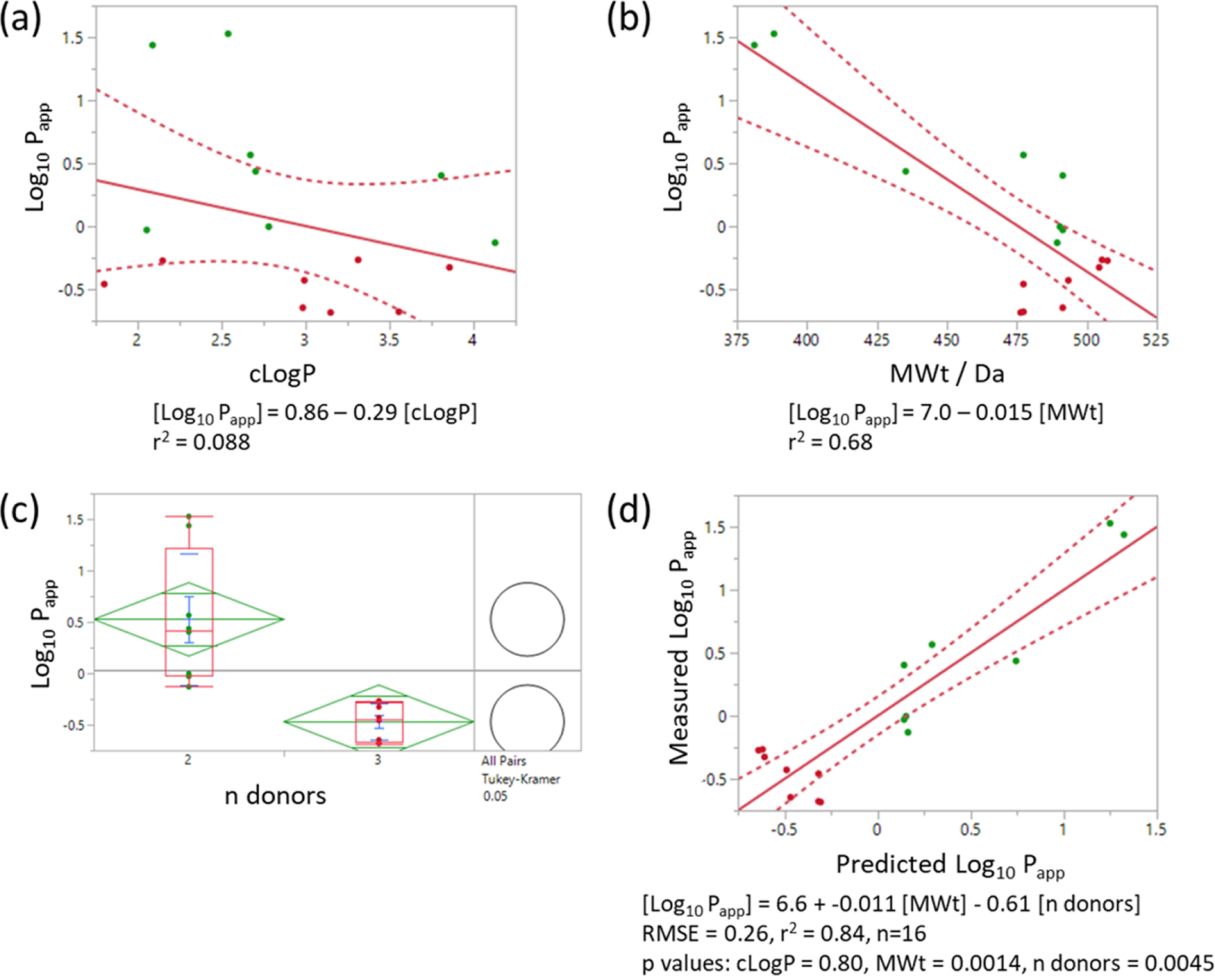
QSAR modeling of the caco-2 *P*_app_ (A to B) data. Correlation with (a) clogP, (b) MWt, (c) distributions against hydrogen bond donor count with paired *t*-test for significance (Tukey–Kramer method) showing mean diamonds (green) and box plots (red), and (d) multilinear regression model using molecular weight and hydrogen bond donor count. Points are colored by hydrogen bond donor count (green = 2, red = 3); red lines show the line of best fit (solid line) and 95% confidence limits for the fit (dotted curves).

The permeability of the compounds within this series varied significantly, with caco-2 *P*_app_ values spanning 2 orders of magnitude. Permeability is usually considered to depend primarily on a combination of lipophilicity, molecular size, and hydrogen-bonding potential (or surrogates thereof).^17−19^ In this series, *P*_app_ correlated with molecular weight and hydrogen bond donor count but, interestingly, no relationship with clogP was apparent (Figure 5a–c). Multilinear regression analysis confirmed the significance of molecular weight and hydrogen bond donors and lack of dependence on clogP (*p* value 0.80 when included in the model; Figure 5d). A multilinear model including molecular weight and donor count alone was able to account for the majority of the variance (RMSE = 0.26). Most significantly, this modeling suggests that it is challenging to achieve a *P*_app_ value > 1 in this series with three hydrogen bond donors, highlighting the need to restrict designs to two donors.^20^ In this case, this effect is likely exacerbated by the presence of two very strong hydrogen-bonding groups (acyl pyrrole and aryl carboxamide) that cannot be readily internally satisfied. It is noteworthy that within this series, molecular weight and basicity are codependent, with the basic compounds also being larger due to the addition of the basic group. The large apparent dependence of permeability on molecular weight within this series may result from the combined effects of both the increased size and basicity as molecular weight increases.

**Figure 6:**
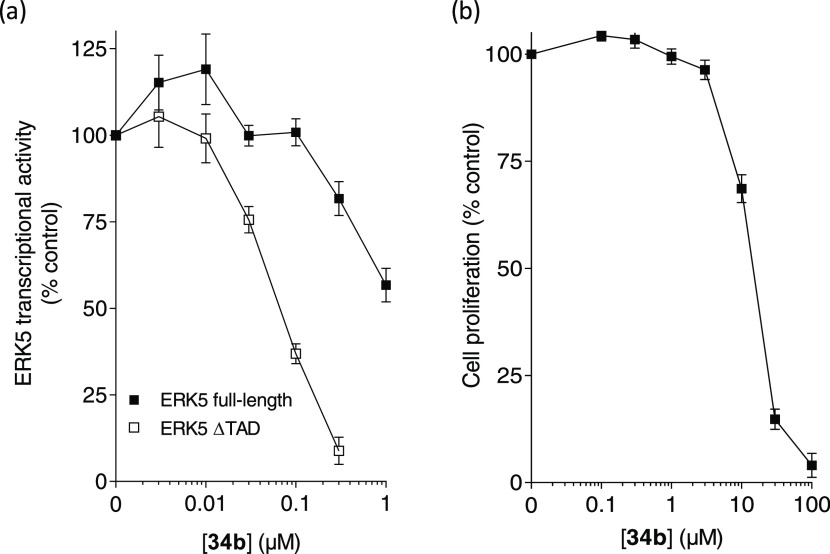
Activity of **34b** in HEK293 cellular assays. (a) Activity of **34b** in an ERK5:MEF2D luciferase reporter assay examining ERK5 ΔTAD, a truncated form of ERK5 containing the kinase domain but lacking the C-terminal extension, or full-length ERK5 (mean ± SEM, *n* = 5 separate experiments); (b) growth inhibition following a 72 h incubation with compound **34b** (mean ± SEM, *n* = 8 separate experiments).

We examined the activity of **34b** in cellular assays to assess the impact on ERK5 kinase and transcriptional activity and proliferation. A recent study, examining the ERK5 kinase inhibitor AX15836 and two derivative compounds, indicated that while these inhibitors suppressed ERK5 kinase activity effectively in HEK293 cells, kinase inhibition also led to a paradoxical activation of ERK5 transcriptional activity by inducing a conformational change in the protein, resulting in the separation of the C-terminal transcriptional activation domain (TAD) from the nuclear localization sequence (NLS), to allow ERK5 nuclear translocation.^21,22^ To examine whether this paradoxical activation extended to another chemotype, we examined the effect of **34b** using the same previously described ERK5:MEF2D luciferase reporter assay.^21^ When examining a truncated ERK5 construct that lacked both the NLS and TAD (ERK5 ΔTAD), **34b** inhibited its kinase activity in cells with an IC_50_ of 77 ± 4 nM (mean ± SEM, *n* = 5) (Figure 6a). However, a greater than 13-fold reduction in activity was observed (i.e., IC_50_ > 1 μM) when **34b** was examined against full-length ERK5, suggesting that this compound also induces a paradoxical activation of ERK5 transcriptional activity. The effect of compound treatment on cellular proliferation over a 72 h period was also examined. The concentration of compound **34b** that prevented a 50% inhibition of HEK293 growth (GI_50_) was 19.6 ± 0.5 μM (mean ± SE) (Figure 6b), a value that is 65-fold greater than that required to inhibit the kinase activity of ERK5 ΔTAD in HEK293 cells by 89% (Figure 5a; 0.3 μM, **34b**). Comparable GI_50_ values were obtained with **34b** in the human renal cell carcinoma cell line A498 (22.3 ± 1.5 μM), the osteosarcoma cell line SJSA-1 (25.0 ± 0.8), and the breast cancer cell line MDA-MB-231 (26.6 ± 1.4 μM) (mean ± SE, three to five separate experiments). While the ERK5 kinase inhibitor XMD8-92 (5 μM) has been previously shown to inhibit the growth of MDA-MB-231 cells by nearly 40%,^23^ none of these three tumor cell lines demonstrate a dependency on ERK5 following siRNA gene silencing in publicly accessible data sets (Supporting Information, Figure S50; https://depmap.org/portal/).^24^ Collectively, these data suggest that the antiproliferative activity of **34b** in cells at concentrations of 10 μM and above is unlikely to result from ERK5 kinase inhibition.

## Conclusions

Parallel optimization of potency and ADME properties has delivered a compound with balanced potency and oral exposure. Introduction of small lipophilic substituents at the 3-position of the benzoyl group of pyrrole carboxamide ERK5 inhibitors led to improved inhibition. Appending a basic center to the heteroaromatic amide substituent provided nanomolar inhibitors. However, the more potent basic analogues suffered from high efflux ratios in the caco-2 membrane permeability assay that translated to low oral bioavailability *in vivo*. Smaller, nonbasic analogue **34b** provided the best balance of potency and *in vitro* ADME properties and had good oral bioavailability in mouse.

While **34b** (10–300 nM) inhibited the kinase activity of ERK5 without a TAD in cells, its reduced activity against the full-length ERK5 protein suggested that it can also activate ERK5 transcriptional activity in a manner comparable to AX15836 and BAY-885.^21,22^ Given that this phenomenon has now been observed with three different chemotypes, it highlights a need to evaluate the effect of any new ERK5 kinase inhibitor on ERK5 transcriptional activity. The conformational activation of ERK5 transcriptional activity by compounds may potentially result in a disconnect between the chemical inhibition of ERK5 kinase and phenotypes observed using siRNA-mediated gene silencing or CRISPR/Cas9 gene editing of *MAPK7*. Nonetheless, such ERK5 kinase inhibitors could find additional utility as ligands for targeted protein degradation strategies that should more closely phenocopy the consequences of ERK5 protein loss following genetic perturbation.

## Experimental Section

### General Procedures

All commercial reagents were purchased from Sigma-Aldrich Chemical Company, Alfa Aesar, Apollo Scientific, or Tokyo Chemical Industry U.K. Ltd. The chemicals were of the highest available purity. Unless otherwise stated, chemicals were used as supplied without further purification. Anhydrous solvents were obtained from AcroSeal or Aldrich SureSeal bottles and were stored under nitrogen. Petrol refers to the fraction with a boiling point between 40 and 60 °C. Thin-layer chromatography utilized to monitor reaction progress was conducted on plates precoated with silica gel Merck 60F254 or Merck NH_2_F254S. The eluent was as stated (where this consisted of more than one solvent, the ratio is stated as volume/volume), and visualization was either by short wave (254 nm) ultraviolet light or by treatment with the visualization reagent stated followed by heating. “Flash” medium-pressure liquid chromatography (MPLC) was carried out either on a Biotage SP4 automated purification system or on a Varian 971-FP automated purification system using prepacked Varian or Grace silica or amino-bonded silica cartridges. All reactions carried out in a microwave were performed in a Biotage Initiator with 60 robots. Melting points were determined using a VWR Stuart SMP40 apparatus and are uncorrected. ^1^H, ^13^C, and ^19^F NMR spectra were obtained as either CDCl_3_, CD_3_OD, or DMSO-*d*_6_ solutions and recorded at 500, 126, and 471 MHz, respectively, on a Bruker Avance III 500 spectrometer. Where ^13^C NMR data are not quoted, insufficient material was available or problems obtaining high-resolution spectra were encountered. Chemical shifts are quoted in parts per million (δ) referenced to the appropriate deuterated solvent employed. Multiplicities are indicated by s (singlet), d (doublet), t (triplet), q (quartet), quin (quintet), m (multiplet), br (broad), or combinations thereof. Coupling constant values are given in Hz. Homonuclear and heteronuclear two-dimensional NMR experiments were used where appropriate to facilitate the assignment of chemical shifts. Liquid chromatography–mass spectrometry (LC–MS) was carried out on a Waters Acquity UPLC system with PDA and ELSD employing positive or negative electrospray modes as appropriate to the individual compound. High-resolution mass spectrometry was performed by the EPSRC U.K. National Mass Spectrometry Facility, University of Wales Swansea, Singleton Park, Swansea, SA2 8PP. FTIR spectra were recorded on either a Bio-Rad FTS 3000MX diamond ATR or an Agilent Cary 630 FTIR as a neat sample. UV spectra were obtained using a U-2001 Hitachi Spectrophotometer with the sample dissolved in ethanol. All compounds are >95% pure by HPLC.

#### General Procedure A

To a suspension of AlCl_3_ (2.5 equiv) in CH_2_Cl_2_ (1 mL/mmol AlCl_3_) at 0 °C was added the relevant acid chloride (2 equiv) followed by methyl-1*H*-pyrrole-2-carboxylate (1 equiv). The resulting mixture was allowed to reach RT and stirred for 16 h. The reaction was quenched at 0 °C with a 1 M hydrochloric acid (20 mL). The product was extracted with CH_2_Cl_2_ (3 × 100 mL) and washed with a saturated aqueous NaHCO_3_ (2 × 100 mL) and brine (100 mL). The combined organic layers were dried over Na_2_SO_4_ and concentrated *in vacuo*.

#### General Procedure B

To a solution of pyrrole ester (1.0 equiv) in THF (8 mL/mmol) was added LiOH (20 equiv) in water (13 mL/mmol). The resulting reaction mixture was heated at 60 °C for 18 h, cooled to RT, and acidified to pH 4–5 with 1 M hydrochloric acid. The product was extracted into EtOAc (100 mL/mmol), washed with water (100 mL/mmol) and brine (100 mL/mmol), and dried over Na_2_SO_4_. The solvent was removed *in vacuo* to obtain the product.

#### General Procedure C

The appropriate carboxylic acid (1.0 equiv) was dissolved in MeCN (5 mL/mmol pyrrole) before the relevant amine (2.5 equiv) was added followed by phosphorus trichloride (1.0 equiv). The mixture was heated using microwave irradiation at 150 °C for 5 min. The reaction was quenched with a few drops of water, and the solvent was removed *in vacuo.* The residue was dissolved in EtOAc (50 mL/mmol pyrrole) and washed with saturated aqueous NaHCO_3_ (50 mL/mmol pyrrole) before being extracted with EtOAc (3 × 30 mL/mmol pyrrole). The combined organic extracts were dried over Na_2_SO_4_, and the filtrate was concentrated *in vacuo* to afford the crude product.

#### General Procedure D

The relevant nitro compound (1 equiv) was dissolved in MeOH (5 mL/mmol) and hydrogenated on a Thales H-cube over a 10% Pd/C CatCart under a full pressure of hydrogen at 40 °C for 2 h with continuous recycling of the reaction mixture at 1 mL/min flow rate. The solvent was removed *in vacuo*.

#### General Procedure E

Cyanuric fluoride (0.7 equiv) was added to the relevant carboxylic acid (1 equiv) and pyridine (1 equiv) in MeCN (2 mL/mmol). The relevant amine (2.5 equiv) was added, and the mixture was stirred at RT for 18 h. The reaction was diluted with EtOAc, washed with water and 0.5 M hydrochloric acid, followed by further washes with saturated aqueous NaHCO_3_ and brine. The organic layer was dried over MgSO_4_, and the solvent was removed *in vacuo*.

#### General Procedure F

The relevant amine (1 equiv), 2-chloro-5-nitropyrimidine (1 equiv), and Et_3_N (1.1 equiv) were combined in THF (5 mL/mmol) at 0 °C, and the mixture was allowed to stir at RT for 1 h. The solvent was removed *in vacuo*, and the residue was partitioned between EtOAc (2 × 30 mL) and water (20 mL). The organic layer was washed with brine, dried over MgSO_4_, and the solvent was removed *in vacuo*.

#### General Procedure G

Diethyl azodicarboxylate (1.5 equiv) was added dropwise to a mixture of 4-nitropyrazole (1 equiv), triphenylphosphine (1.73 g, 6.63 mmol, 1.5 equiv), and the substrate alcohol (1 equiv) in THF at 0 °C. The mixture was stirred at 0 °C for 10 min and then allowed to stir at RT for 18 h. The reaction mixture was partitioned between EtOAc (2 × 30 mL) and water (20 mL), washed with brine (20 mL), dried over MgSO_4_, and the solvent was removed *in vacuo*.

#### General Procedure H

Formaldehyde (37% w/v aqueous, 4 equiv) was added to the substrate carbamate (1 equiv) in formic acid (10 mL/mmol), and the mixture was heated to 100 °C for 3 h in a sealed tube. The mixture was allowed to cool, basified with 10% aqueous K_2_CO_3_, and extracted with EtOAc (2 × 20 mL). The organic extracts were combined, washed with brine, dried over MgSO_4_, and the solvent was removed *in vacuo.*

#### General Procedure I

Pyrrole acid (1 equiv), Et_3_N (2.5 equiv), and 2-chloro-1-methylpyridinium iodide (1.1 equiv) were combined in CH_2_Cl_2_ (15 mL/mmol) and stirred at RT for 10 min, followed by the addition of the substrate amine (1.25 equiv) in CH_2_Cl_2_ (2.5 mL/mmol). The reaction was stirred at RT for 18 h, the solvent was evaporated, and the residue was partitioned between EtOAc (2 × 15 mL) and 10% aqueous K_2_CO_3_ (15 mL). The organic layers were combined, washed with brine, dried over MgSO_4_, and the solvent was removed *in vacuo*.

#### General Procedure J

TFA (2 mL/mmol) and Et_3_SiH (2.5 equiv) were added to the relevant carbamate (1 equiv) in CH_2_Cl_2_ (2 mL/mmol), and the mixture was stirred at RT for 2 h. The solvent was removed *in vacuo*, and the residue was partitioned between EtOAc (5 × 30 mL) and saturated aqueous NaHCO_3_ (40 mL). The organic extracts were combined, dried over MgSO_4_, and the solvent was removed *in vacuo.*

##### Methyl-4-(2-chloro-6-fluorobenzoyl)-1*H*-pyrrole-2-carboxylate (**30a**)

Prepared according to general procedure A, where AlCl_3_ (2.23 g, 16.8 mmol), CH_2_Cl_2_ (17 mL), 2-chloro-6-fluorobenzoyl chloride (1.80 mL, 13.3 mmol), and methyl-1*H*-pyrrole-2-carboxylate (847 mg, 6.70 mmol) were added. The crude mixture was purified by MPLC on SiO_2_ with a gradient elution from 0 to 100% EtOAc/petrol to give a white solid (1.74 g, 92%); *R*_f_ 0.50 (SiO_2_, 5% MeOH/CH_2_Cl_2_); mp 148–150 °C; λ_max_ (EtOH)/nm 280, 233; IR ν_max_/cm^–1^ 3226, 1731, 1638, 1604; ^1^H NMR (500 MHz; DMSO-*d*_6_) δ_H_ 3.83 (3H, s, OMe), 7.02–7.05 (1H, m, H-3), 7.42 (1H, app td, *J* = 8.2 and 0.8 Hz, H-5′), 7.49 (1H, d, *J* = 8.2 Hz, H-3′), 7.57 (1H, dd, *J* = 1.7 and 3.3 Hz, H-5), 7.61 (1H, td, *J* = 8.2 and 6.3 Hz, H-4′), 12.94 (1H, br s, NH); ^13^C NMR (125 MHz; DMSO-*d*_6_) δ_C_ 51.7 (OMe), 114.6 (CH-pyrrole), 115.0 (d, *J*_CF_ = 21.8 Hz, C-5′), 124.5 (C-pyrrole), 125.4 (C-pyrrole), 125.9 (d, *J*_CF_ = 3.2 Hz, C-3′), 127.7 (d, *J*_CF_ = 23.2 Hz, C-1′), 130.1 (C-pyrrole), 130.3 (d, *J*_CF_ = 6.4 Hz, C-2′), 131.9 (d, *J*_CF_ = 9.1 Hz, C-4′), 158.5 (d, *J*_CF_ = 246.9 Hz, C-6′), 160.3 (CO-NH), 183.7 (CO); ^19^F NMR (470 MHz; DMSO-*d*_6_) δ_F_ −114.4; HRMS calcd for C_13_H_10_^35^Cl_1_F_1_O_3_N_1_ [M + H]^+^ 282.0328, found 282.0333.

##### 4-(2-Chloro-6-fluorobenzoyl)-1*H*-pyrrole-2-carboxylic Acid (**31a**)

Prepared according to general procedure B using LiOH (2.90 g, 121 mmol) in water (80 mL) and ester **30a** (1.70 g, 6.05 mmol) in THF (48 mL) to give a white solid (1.60 g, 99%); *R*_f_ 0.15 (SiO_2_, 10% MeOH/CH_2_Cl_2_); mp 220–222 °C; λ_max_ (EtOH)/nm 281, 232; IR ν_max_/cm^–1^ 3313, 1637, 1553; ^1^H NMR (500 MHz; DMSO-*d*_6_) δ_H_ 6.98 (1H, br s, H-pyrrole), 7.42 (td, *J* = 8.2 and 0.6 Hz, H-5′), 7.47–7.51 (2H, m, H-3′ and H-pyrrole), 7.61 (1H, td, *J* = 8.2 and 6.3 Hz, H-4′), 12.75 (1H, br s, NH-pyrrole), 12.97 (1H, br s, CO_2_H); ^13^C NMR (125 MHz; DMSO-*d*_6_) δ_C_ 114.1 (CH-pyrrole), 114.9 (d, *J*_CF_ = 21.3 Hz, C-5′), 125.3 (C-pyrrole), 125.8 (d, *J*_CF_ = 3.2 Hz, C-3′), 125.9 (C-pyrrole), 127.8 (C-pyrrole), 127.9 (d, *J*_CF_ = 23.2 Hz, C-1′), 129.7 (C-pyrrole), 130.3 (d, *J*_CF_ = 6.0 Hz, C-2′), 131.8 (d, *J*_CF_ = 9.1 Hz, C-4′), 158.5 (d, *J*_CF_ = 247 Hz, C-6′), 161.3 (CO-NH), 183.7 (CO); ^19^F NMR (470 MHz; DMSO-*d*_6_) δ_F_ −114.4; HRMS calcd for C_12_H_6_^35^Cl_1_F_1_N_1_O_3_ [M + H]^+^ 266.0026, found 266.0018.

##### Methyl-4-(3,6-dichloro-2-fluorobenzoyl)-1*H*-pyrrole-2-carboxylate (**30b**)

Prepared according to general procedure A, where AlCl_3_ (11.9 g, 89.6 mmol) in CH_2_Cl_2_ (100 mL), 3,6-dichloro-2-fluorobenzoyl chloride (16.3 mL, 71.7 mmol), and methyl-1*H*-pyrrole-2-carboxylate (4.48 g, 35.8 mmol) were added. The crude mixture was purified by MPLC on SiO_2_ with a gradient elution from 0 to 5% EtOAc/petrol to give a white solid (10.1 g, 89%); *R*_f_ 0.80 (SiO_2_, 50:50:0.5 EtOAc/petrol/AcOH); mp 136–138 °C; λ_max_ (EtOH)/nm 282, 229; IR ν_max_/cm^–1^ 3285, 3230, 1689, 1654; ^1^H NMR (500 MHz; DMSO-*d*_6_) δ_H_ 3.84 (3H, s, CH_3_), 7.12 (1H, app t, *J* = 1.5 Hz, H-pyrrole), 7.53 (1H, dd, *J* = 0.9 and 8.5 Hz, H-5′), 7.71 (1H, dd, *J* = 1.5 and 3.2 Hz, H-pyrrole), 7.80 (1H, app t, *J* = 8.5 Hz, H-4′), 12.98 (1H, s, NH-pyrrole); ^13^C NMR (125 MHz; DMSO-*d*_6_) δ_C_ 51.7 (CH_3_), 114.6 (CH-pyrrole), 119.4 (d, *J*_CF_ = 18.1 Hz, C-3′), 124.8 (C-pyrrole), 124.9 (C-pyrrole), 126.8 (d, *J*_CF_ = 4.1 Hz, C-5′), 128.9 (d, *J*_CF_ = 22.7 Hz, C-1′), 129.1 (d, *J*_CF_ = 5.2 Hz, C-6′), 131.0 (C-pyrrole), 131.9 (C-4′), 153.8 (d, *J*_CF_ = 248.5 Hz, C-2′), 160.3 (*C*O_2_Me), 182.5 (CO); ^19^F NMR (470 MHz; DMSO-*d*_6_) δ_F_ −116.7; MS (ES^+^) *m*/*z* 314.2 [M(^35^Cl) + H]^+^, 316.1 [M(^37^Cl) + H]^+^.

##### 4-(3,6-Dichloro-2-fluorobenzoyl)-1*H*-pyrrole-2-carboxylic Acid (**31b**)

Prepared according to general procedure B using LiOH (26.6 g, 632 mmol) in water (140 mL) and ester **30b** (10.0 g, 31.6 mmol) in THF (180 mL) to give a white solid (9.00 g, 95%); *R*_f_ 0.25 (SiO_2_, 50:50:0.5 EtOAc/petrol/AcOH); mp 230–232 °C; λ_max_ (EtOH)/nm 289, 267; IR ν_max_/cm^–1^ 3264br, 1697, 1646; ^1^H NMR (500 MHz; DMSO-*d*_6_) δ_H_ 7.07 (1H, app t, *J* = 1.6 Hz, H-pyrrole), 7.53 (1H, dd, *J* = 1.1 and 8.6 Hz, H-5′), 7.71 (1H, dd, *J* = 1.6 and 3.2 Hz, H-pyrrole), 7.80 (1H, app t, *J* = 8.6 Hz, H-4′), 12.81 (1H, br s, NH-pyrrole), 13.00 (1H, br s, CO_2_H); ^13^C NMR (125 MHz; DMSO-*d*_6_) δ_C_ 114.1 (CH-pyrrole), 119.3 (d, *J*_CF_ = 18.1 Hz, C-3′), 124.8 (C-pyrrole), 126.1 (C-pyrrole), 126.8 (d, *J*_CF_ = 3.6 Hz, C-5′), 129.0 (d, *J*_CF_ = 23.3 Hz, C-1′), 129.1 (d, *J*_CF_ = 5.5 Hz, C-6′), 130.6 (C-pyrrole), 131.9 (C-4′), 153.8 (d, *J*_CF_ = 248.4 Hz, C-2′), 161.3 (CO_2_H), 182.5 (CO); ^19^F NMR (470 MHz; DMSO-*d*_6_) δ_F_ −116.7; MS (ES^+^) *m*/*z* 302.1 [M(^35^Cl) + H]^+^, 304.1 [M(^37^Cl) + H]^+^.

##### 4-(2-Chloro-6-fluorobenzoyl)-*N*-(pyrimidin-5-yl)-1*H*-pyrrole-2-carboxamide (**32a**)

Compound **32a** was synthesized according to general procedure C using 4-(2-chloro-6-fluoro-benzoyl)-1*H*-pyrrole-2-carboxylic acid (**31a**) (100 mg, 0.37 mmol), MeCN (2 mL), 5-aminopyrimidine (88 mg, 0.93 mmol), and PCl_3_ (32 μL, 0.37 mmol) to afford the crude product. Purification was achieved using MPLC on SiO_2_ with a gradient elution from 0 to 10% MeOH/EtOAc to give an orange solid (100 mg, 79%); *R*_f_ 0.52 (SiO_2_, 5% MeOH/EtOAc); mp 227 °C (dec.); λ_max_ (EtOH)/nm 262.0, 292.0; IR 2960, 2862, 1968, 1637 (CO), 1529 (CONH); ^1^H NMR (500 MHz, DMSO-*d*_6_) δ 7.42 (1H, dd, *J* = 1.0 and 9.0 Hz, H-5′), 7.48–7.50 (2H, m, H-3′ and H-3), 7.55 (1H, s, H-5), 7.60 (1H, ddd, *J* = 6.3, 8.3 and 8.3 Hz, H-4′), 8.92 (1H, s, N-CH-N-pyrimidine), 9.13 (2H, s, 2 × CH-N-pyrimidine), 10.44 (1H, s, CONH), 12.93 (1H, s, NH); ^13^C NMR (125 MHz, DMSO-*d*_6_) δ 112.1 (C-pyrimidine), 114.9 (C-Ar), 115.1 (d, *J*_CF_ = 23.4 Hz, C-Ar), 125.3 (C-Ar), 125.9 (C-3), 127.6 (C-2 and C-5), 129.7 (C-4), 130.4 (d, *J*_CF_ = 22.8 Hz, C-Ar), 131.9 (d, *J*_CF_ = 8.6 Hz, C-Ar), 134.3 (C-N-pyrimidine), 147.8 (C-Ar), 153.2 (d, *J*_CF_ = 245.2 Hz, CF), 158.8 (CON), 183.9 (CO); ^19^F NMR (470 MHz, DMSO-*d*_6_) δ −114.3; HRMS *m*/*z* calcd for C_16_H_11_^35^ClFN_4_O_2_ [M + H]^+^ 345.0549, found 345.0550.

##### 4-(2-Chloro-6-fluorobenzoyl)-*N*-(2-methylpyrimidin-5-yl)-1*H*-pyrrole-2-carboxamide (**32b**)

Compound **32b** was synthesized according to general procedure C using 4-(2-chloro-6-fluorobenzoyl)-1*H*-pyrrole-2-carboxylic acid (**31a**) (100 mg, 0.37 mmol), 2-methylpyrimidin-5-amine (102 mg, 0.93 mmol), PCl_3_ (32 μL, 0.37 mmol), and MeCN (2 mL). The crude mixture was purified by MPLC on SiO_2_ with a gradient elution from 0 to 8% MeOH/CH_2_Cl_2_ to give a yellow solid (53 mg, 0.14 mmol, 40%); *R*_f_ 0.45 (SiO_2_, 5% MeOH/CH_2_Cl_2_); mp 270 °C (dec.); λ_max_ (EtOH)/nm 268, 290, 379; IR ν_max_/cm^–1^ 3320, 1637 (CO), 1516 (CONH); ^1^H NMR (500 MHz, MeOD) δ 2.68 (3H, s, CH_3_), 7.25–7.28 (1H, m, H-5′), 7.40 (1H, d, *J* = 8.5 Hz, H-3′), 7.44 (1H, d, *J* = 1.5 Hz, H-3), 7.49 (1H, d, *J* = 1.5 Hz, H-5), 7.53 (1H, ddd, *J* = 6.1, 8.5 and 8.5 Hz, H-4′), 9.07 (2H, s, CH-pyrimidine); ^13^C NMR (125 MHz, MeOD) δ 25.0 (CH_3_), 108.4 (C-pyrimidine), 115.0 (C-Ar), 121.9 (C-Ar), 122.0 (C-3), 126.5 (C-2 and C-5), 127.2 (C-pyrimidine), 131.0 (C-4), 135.6 (C-Ar), 143.2 (C-N-pyridine), 156.7 (N-C-N-pyrimidine), 154.6 (d, *J*_CF_ = 253.2 Hz, CF), 159.8 (CON), 187.0 (CO); ^19^F NMR (470 MHz, DMSO-*d*_6_) δ −115.3; HRMS *m*/*z* calcd for C_17_H_12_^35^ClFN_4_O_2_ [M + H]^+^ 359.0710, found 359.0710.

##### 2-Methoxy-5-nitropyrimidine^25,26^ (**6a**)

Sodium (35 mg, 1.50 mmol) was added to MeOH (5 mL), and the mixture was stirred under nitrogen until the sodium had dissolved. 5-Nitro-2-chloropyrimidine (200 mg, 1.25 mmol) was added, and the reaction mixture was heated at reflux for 1 h. The solvent was removed *in vacuo*, and the residue was purified by MPLC on SiO_2_ with a gradient elution from 10 to 20% EtOAc/petrol to give a yellow solid (115 mg, 59%); *R*_f_ 0.40 (SiO_2_, 20% EtOAc/petrol); mp 65–67 °C (Lit.^25^ 69–70 °C); λ_max_ (EtOH)/nm 270 nm; IR ν_max_/cm^–1^ 1567, 1474, 1404, 1315; ^1^H NMR (500 MHz; DMSO-*d*_6_) δ_H_ 4.08 (3H, s, OMe), 9.42 (2H, s, H-pyrimidine); ^13^C NMR (125 MHz; DMSO-*d*_6_) δ_C_ 56.4 (Me), 138.8 (C-NO_2_), 156.4 (2 × CH-pyrimidine), 166.7 (*C-*O-Me); MS (ES^+^) *m*/*z* 156.2 [M + H]^+^.

##### 2-Methoxypyrimidin-5-amine^27^ (7a)

Prepared according to general procedure D using nitropyrimidine **6a** (140 mg, 0.9 mmol) in MeOH (5 mL) for 90 min. The solvent was removed *in vacuo* to give a white solid (109 mg, 97%); *R*_f_ 0.10 (NH_2_ SiO_2_, 5% MeOH/CH_2_Cl_2_); mp 113–116 °C (lit.^27^ 119–120 °C); λ_max_ (EtOH)/nm 327, 237; IR ν_max_/cm^–1^ 3304, 3180, 1648(w), 1565; ^1^H NMR (500 MHz; DMSO-*d*_6_) δ_H_ 3.78 (3H, s, OMe), 5.01 (2H, s, NH_2_), 7.99 (s, 2H, H-pyrimidine); ^13^C NMR (125 MHz; DMSO-*d*_6_) δ_C_ 53.9 (OMe), 137.9, (C-NH_2_), 144.3 (2 × CH-pyrimidine), 157.8 (*C-*O-Me); MS (ES^+^) *m*/*z* 126.2 [M + H]^+^.

##### 4-(2-Chloro-6-fluorobenzoyl)-*N*-(2-methoxypyrimidin-5-yl)-1*H*-pyrrole-2-carboxamide (**32c**)

Prepared according to general procedure E using carboxylic acid **31a** (86 mg, 0.32 mmol), amine **7a** (100 mg, 0.8 mmol), pyridine (26 μL, 0.32 mmol), and cyanuric fluoride (19 μL, 0.22 mmol). Purification by MPLC on SiO_2_ with a gradient elution from 0 to 3% MeOH/CH_2_Cl_2_ gave a white solid (52 mg, 43%); *R*_f_ 0.15 (SiO_2_, 50% EtOAc/petrol); mp 258 °C dec.; λ_max_ (EtOH)/nm 267; IR ν_max_/cm^–1^ 3337, 2961, 1649, 1616, 1583; ^1^H NMR (500 MHz; DMSO-*d*_6_) δ_H_ 3.94 (3H, s, OMe), 7.41–7.48 (2H, m, H-pyrrole and H-5′), 7.51 (1H, d, *J* = 8.3 Hz, H-3′), 7.54 (1H, br s, H-pyrrole), 7.62 (1H, td, *J* = 8.3 and 6.3 Hz, H-4′), 8.90 (2H, s, 2 × CH-pyrimidine), 10.34 (1H, s, NH), 12.78 (1H, br s, NH); ^19^F NMR (470 MHz; DMSO-*d*_6_) δ_F_ −114.3; ^13^C NMR (125 MHz; DMSO-*d*_6_) δ_C_ 54.7 (OMe), 111.6 (CH-pyrrole), 115.0 (d, *J*_CF_ = 21.3 Hz, C-5′), 125.3 (C-pyrrole), 125.9 (d, *J*_CF_ = 3.0 Hz, C-3′), 127.8 (C-pyrimidine), 128.0 (d, *J*_CF_ = 23.2 Hz, C-1′), 128.6 (C-pyrrole), 129.3 (CH-pyrrole), 130.4 (d, *J*_CF_ = 5.9 Hz, C-2′), 131.9 (d, *J*_CF_ = 9.1 Hz, C-4′), 151.4 (C-pyrimidine), 156.6 (d, *J*_CF_ = 248 Hz, C-6′), 158.55 (C-pyrimidine), 161.3 (CO-NH), 183.9 (CO); MS (ES^+^) *m*/*z* 375.3 [M(^35^Cl) + H]^+^, 377.3 [M(^37^Cl) + H]^+^; HRMS calcd for C_17_H_12_^35^Cl_1_F_1_N_4_O_3_ [M + H]^+^ 375.0655, found 375.0648.

##### *N*,*N*-Dimethyl-5-nitropyrimidin-2-amine^28^ (**6b**)

Prepared according to general procedure F using 2-chloro-5-nitropyrimidine (300 mg, 1.90 mmol, 1 equiv), Me_2_NH (1.40 mL, 2.80 mmol, 1.5 equiv, 2.0 M in THF), and Et_3_N (288 μL, 2.10 mmol, 1.1 equiv) in THF (8 mL) to give a yellow solid (275 mg, 87%); *R*_f_ 0.75 (NH_2_ SiO_2_, 30% EtOAc/petrol); mp 209–212 °C (lit.^28^ 222 °C); λ_max_ (EtOH)/nm 341, 219; IR ν_max_/cm^–1^ 1547, 1301; ^1^H NMR (500 MHz; DMSO-*d*_6_) δ_H_ 3.31 (6H, s, NMe_2_), 9.15 (2H, s, 2 × H-pyrimidine); ^13^C NMR (125 MHz; DMSO-*d*_6_) δ_C_ 37.4 (NMe_2_), 133.4 (C-5-pyrimidine), 154.8 (2H, s, 2 × CH-pyrimidine), 161.6 (C-2-pyrimidine); HRMS calcd for C_6_H_9_N_4_O_2_ [M + H]^+^ 169.0720, found 169.0720.

##### *N*^2^,*N*^2^-Dimethylpyrimidine-2,5-diamine^29^ (**7b**)

Prepared according to general procedure D using nitropyrimidine **6b** (263 mg, 1.60 mmol) in MeOH (60 mL) and EtOAc (60 mL) for 4 h to give a yellow solid (215 mg, 100%); *R*_f_ 0.80 (NH_2_ SiO_2_, EtOAc); mp 68–72 °C; λ_max_ (EtOH)/nm 261; IR ν_max_/cm^–1^ 3206; ^1^H NMR (500 MHz; DMSO-*d*_6_) δ_H_ 3.12 (6H, s, NMe_2_), 4.50 (2H, s, NH_2_), 7.01 (2H, s, 2 × CH-pyrimidine); ^13^C NMR (125 MHz; DMSO-*d*_6_) δ_C_ 37.2 (NMe_2_), 133.2 (C-5-pyrimidine), 144.3 (2 × CH-pyrimidine), 156.7 (C-2-pyrimidine); HRMS calcd for C_6_H_11_N_4_ [M + H]^+^ 139.0978, found 139.0978.

##### 4-(2-Chloro-6-fluorobenzoyl)-*N*-(2-(dimethylamino)pyrimidin-5-yl)-1*H*-pyrrole-2-carboxamide (**32d**)

Prepared according to general procedure E using amine **7b** (100 mg, 0.72 mmol, 2.5 equiv), carboxylic acid **31a** (77 mg, 0.29 mmol, 1 equiv), cyanuric fluoride (25 μL, 0.20 mmol, 0.7 equiv), pyridine (23 μL, 0.29 mmol, 1 equiv), and MeCN (2 mL). Purification by MPLC on NH_2_ SiO_2_ with a gradient elution from 50 to 100% EtOAc/petrol gave a white solid (38 mg, 34%); *R*_f_ 0.50 (NH_2_ SiO_2_, EtOAc); mp 300 °C dec.; λ_max_ (EtOH)/nm 287, 232; IR ν_max_/cm^–1^ 2951, 1645, 1622; ^1^H NMR (500 MHz; DMSO-*d*_6_) δ_H_ 3.15 (6H, s, NMe_2_), 7.39 (1H, s, H-pyrrole), 7.44 (1H, app t, *J* = 8.4 Hz, H-5′), 7.49 (1H, s, H-pyrrole), 7.50 (1H, d, *J* = 8.4 Hz, H-3′), 7.62 (1H, td, *J* = 8.4 and 6.3 Hz, H-4′), 8.61 (2H, s, 2 × H-pyrimidine), 10.02 (1H, s, CO-NH), 12.70 (1H, s, NH); ^19^F NMR (470 MHz; DMSO-*d*_6_) δ_F_ −114.4; ^13^C NMR (125 MHz; DMSO-*d*_6_) δ_C_ 36.9 (NMe_2_), 111.1 (CH-pyrrole), 115.0 (d, *J*_CF_ = 21.5 Hz, C-5′), 122.9 (C-5-pyrimidine), 125.2 (C-pyrrole), 125.8 (d, *J*_CF_ = 3.2 Hz, C-3′), 128.1 (d, *J*_CF_ = 23.2 Hz, C-1′), 128.2 (C-pyrrole), 128.9 (CH-pyrrole), 130.4 (d, *J*_CF_ = 5.9 Hz, C-2′), 131.8 (d, *J* = 9.1 Hz, C-4′), 151.2 (2 × CH-pyrimidine), 158.4 (C-2-pyrimidine), 158.6 (d, *J*_CF_ = 247.0 Hz, C-6′), 159.1 (CO-NH), 183.9 (CO); HRMS calcd for C_18_H_16_^35^Cl_1_F_1_N_5_O_2_ [M + H]^+^ 388.0971, found 388.0977.

##### 4-(5-Nitropyrimidin-2-yl)morpholine (**6c**)

Prepared according to general procedure F using 2-chloro-5-nitropyrimidine (300 mg, 1.88 mmol) morpholine (181 μL, 2.07 mmol), Et_3_N (288 μL, 2.07 mmol), and THF (12 mL). The residue was purified by MPLC on SiO_2_ with a gradient elution from 20 to 100% EtOAc/petrol to give a yellow solid (290 mg, 73%); *R*_f_ 0.35 (SiO_2_, 30% EtOAc/petrol); mp 161–164 °C (lit.^30^ 165–168 °C); λ_max_ (EtOH)/nm 339, 221; IR ν_max_/cm^–1^ 1545 (NO_2_), 1326 (NO_2_); ^1^H NMR (500 MHz; DMSO-*d*_6_) δ_H_ 3.71–3.75 (4H, m, 2 × CH_2_-morpholine), 3.94–3.97 (4H, m, 2 × CH_2_-morpholine), 2.14 (2H, s, 2 × H-pyrimidine); ^13^C NMR (125 MHz; DMSO-*d*_6_) δ_C_ 44.5 (2 × CH_2_-morpholine), 65.8 (2 × CH_2_-morpholine), 133.4 (C-5-pyrimidine), 155.1 (C-4 and C-6-pyrimidine), 161.0 (C-2-pyrimidine); HRMS calcd for C_8_H_11_N_4_O_3_ [M + H]^+^ 211.0826, found 211.0828.

##### 2-Morpholinopyrimidin-5-amine (**7c**)

Prepared according to general procedure D using nitropyrimidine **6c** (278 mg, 1.54 mmol) in MeOH (35 mL) and EtOAc (15 mL) to give a yellow solid (239 mg, 100%); *R*_f_ 0.30 (NH_2_ SiO_2_, 70% EtOAc/petrol); mp 110–113 °C (lit.^30^ 99 °C); λ_max_ (EtOH)/nm 255; IR ν_max_/cm^–1^ 3301, 3203 (NH_2_); ^1^H NMR (500 MHz; DMSO-*d*_6_) δ_H_ 3.46–3.50 (4H, m, 2 × CH_2_-morpholine), 3.63–3.70 (4H, m, 2 × CH_2_-morpholine), 4.68 (2H, s, NH_2_), 7.94 (2H, s, 2 × H-pyrimidine); ^13^C NMR (125 MHz; DMSO-*d*_6_) δ_C_ 45.0 (2 × CH_2_-morpholine), 66.0 (2 × CH_2_-morpholine), 134.8 (C-5-pyrimidine), 143.9 (C-4 and C-6-pyrimidine), 155.9 (C-2-pyrimidine); HRMS calcd for C_8_H_13_N_4_O_1_ [M + H]^+^ 181.1084, found 181.1084.

##### 4-(2-Chloro-6-fluorobenzoyl)-*N*-(2-morpholinopyrimidin-5-yl)-1*H*-pyrrole-2-carboxamide (**32e**)

Prepared according to general procedure E using amine **7c** (110 mg, 0.61 mmol), carboxylic acid **31a** (65 mg, 0.24 mmol), cyanuric fluoride (15 μL, 0.17 mmol), pyridine (20 μL, 0.24 mmol), and MeCN (2 mL). Purification by MPLC on NH_2_ SiO_2_ with a gradient elution from 50 to 100% EtOAc/petrol gave a white solid (52 mg, 50%); *R*_f_ 0.40 (NH_2_ SiO_2_, EtOAc); mp 286–287 °C; λ_max_ (EtOH)/nm 292, 232; IR ν_max_/cm^–1^ 3211, 1656, 1634; ^1^H NMR (500 MHz; DMSO-*d*_6_) δ_H_ 3.70 (8H, s, 4 × CH_2_-morpholine), 7.41 (1H, m, H-pyrrole), 7.44 (1H, app. t, *J* = 8.3 Hz, H-5′), 7.48–7.52 (2H, m, H-pyrrole and H-3′), 7.62 (1H, td, *J* = 8.3 and 6.3 Hz, H-4′), 8.68 (2H, s, 2 × H-pyrimidine), 10.10 (CO-NH), 12.72 (NH-pyrrole); ^13^C NMR (125 MHz; DMSO-*d*_6_) δ_C_ 44.2 (2 × CH_2_-morpholine), 65.9 (2 × CH_2_-morpholine), 111.2 (CH-pyrrole), 115.0 (d, *J*_CF_ = 21.5 Hz, C-5′), 124.1 (C-pyrimidine), 125.2 (C-pyrrole), 125.8 (*J*_CF_ = 2.7 Hz, C-3′), 128.7 (d, *J*_CF_ = 22.7 Hz, C-1′), 128.1 (C-pyrrole), 129.0 (CH-pyrrole), 130.4 (d, *J*_CF_ = 6.4 Hz, C-2′), 131.8 (d, *J*_CF_ = 8.6 Hz, C-4′), 151.0 (2 × CH-pyrimidine), 158.4 (CO-NH and C-pyrimidine), 158.6 (d, *J* = 247.0 Hz, C-6′), 183.9 (CO); ^19^F NMR (470 MHz; DMSO-*d*_6_) δ_F_ −114.4; HRMS calcd for C_20_H_18_^35^Cl_1_F_1_N_5_O_3_ [M + H]^+^ 430.1077, found 430.1083.

##### 2-(4-Methylpiperazin-1-yl)-5-nitropyrimidine^31^ (**6d**)

Prepared according to general procedure F using 1-methylpiperazine (382 μL, 3.45 mmol), 2-chloro-5-nitropyrimidine (500 mg, 3.14 mmol), Et_3_N (480 μL, 3.45 mmol), and THF (15 mL). The residue was purified by MPLC on SiO_2_ with a gradient elution from 0 to 13% MeOH/EtOAc to give a yellow solid (573 mg, 82%); *R*_f_ 0.60 (NH_2_ SiO_2_, EtOAc); mp 149–152 °C; λ_max_ (EtOH)/nm 346, 332, 219; IR ν_max_/cm^–1^ 1567, 1474; ^1^H NMR (500 MHz; DMSO-*d*_6_) δ_H_ 2.26 (3H, s, Me), 2.41–2.46 (4H, m, H-piperazine), 3.92–3.99 (4H, m, H-piperazine), 9.15 (2H, s, H-pyrimidine); ^13^C NMR (125 MHz; DMSO-*d*_6_) δ_C_ 44.0 (2 × C-piperazine), 45.5 (NMe), 54.1 (2 × C-piperazine), 133.2 (C-NO_2_), 155.1 (2 × CH-pyrimidine), 160.8 (C-pyrimidine); MS (ES^+^) *m*/*z* 224.3 [M + H]^+^; HRMS calcd for C_9_H_14_N_5_O_2_ [M + H]^+^ 224.1142, found 224.1136.

##### 2-(4-Methylpiperazin-1-yl)pyrimidin-5-amine^32^ (**7d**)

Prepared according to general procedure D using nitropyrimidine **6d** (195 mg, 0.87 mmol) and MeOH (5 mL) to give a pale yellow solid (168 mg, 99%); *R*_f_ 0.50 (NH_2_ SiO_2_, 5% MeOH/CH_2_Cl_2_); mp 139–142 °C; λ_max_ (EtOH)/nm 353, 255; IR ν_max_/cm^–1^ 3347, 3173, 2967, 2920, 1640, 1606; ^1^H NMR (500 MHz; DMSO-*d*_6_) δ_H_ 2.22 (3H, s, NMe), 2.31–2.40 (4H, m, H-piperazine), 3.48–3.58 (4H, m, H-piperazine), 4.64 (2H, s, NH_2_), 7.92 (2H, s, H-pyrimidine); ^13^C NMR (125 MHz; DMSO-*d*_6_) δ_C_ 44.3 (2 × C-piperazine), 45.9 (NMe), 54.4 (2 × C-piperazine), 134.3 (C-NH_2_), 144.0 (2 × CH-pyrimidine), 156.0 (C-pyrimidine); MS (ES^+^) *m*/*z* 194.3 [M + H]^+^; HRMS calcd for C_9_H_16_N_5_ [M + H]^+^ 194.1400, found 194.1399.

##### 4-(2-Chloro-6-fluorobenzoyl)-*N*-(2-(4-methylpiperazin-1-yl)pyrimidin-5-yl)-1*H*-pyrrole-2-carboxamide (**32f**)

Prepared according to general procedure E using carboxylic acid **31a** (150 mg, 0.78 mmol), amine **7d** (83 mg, 0.31 mmol), cyanuric fluoride (19 μL, 0.22 mmol), pyridine (25 μL, 0.31 mmol), and MeCN (2 mL). Purification by MPLC on NH_2_ SiO_2_ with a gradient elution from 0 to 4% MeOH/CH_2_Cl_2_ gave a white solid (68 mg, 50%); *R*_f_ 0.3 (NH_2_ SiO_2_, 5% MeOH/CH_2_Cl_2_); mp 246 °C (dec.); λ_max_ (EtOH)/nm 289, 231; IR ν_max_/cm^–1^ 3186, 1661, 1641, 1606, 1582; ^1^H NMR (500 MHz; DMSO-*d*_6_) δ_H_ 2.24 (3H, s, NMe), 2.35–2.42 (4H, m, H-piperazine), 3.68–3.76 (4H, m, H-piperazine), 7.40 (1H, s, H-pyrrole), 7.44 (1H, app t, *J* = 8.2 Hz, H-5′), 7.48–7.54 (2H, m, H-pyrrole and H-3′), 7.62 (1H, td, *J* = 8.2 and 6.3 Hz, H-4′), 8.61 (2H, s, 2 × H-pyrimidine), 10.05 (1H, s, CO-NH), 12.69 (1H, s, NH-pyrrole); ^13^C NMR (125 MHz; DMSO-*d*_6_) δ_C_ 43.6 (2 × CH_2_ piperazine), 45.8 (NMe), 54.3 (2 × CH_2_ piperazine), 111.1 (CH-pyrrole), 115.0 (d, *J*_CF_ = 21.5 Hz, C-5′), 125.2 (C-pyrrole), 125.8 (d, *J*_CF_ = 3.2 Hz, C-3′), 127.9 (C-pyrimidine), 128.0 (d, *J*_CF_ = 22.7 Hz, C-1′), 128.1 (C-pyrrole), 129.0 (CH-pyrrole), 130.4 (d, *J*_CF_ = 6.2 Hz, C-2′), 131.8 (d, *J*_CF_ = 9.1 Hz, C-4′), 151.0 (2 × CH-pyrimidine), 158.4 (CO-NH), 158.4 (C-pyrimidine), 158.5 (d, *J*_CF_ = 247 Hz, C-6′), 183.9 (CO); ^19^F NMR (470 MHz; DMSO-*d*_6_) δ_F_ −114.4; MS (ES+) *m*/*z* 443.5 [M(^35^Cl) + H]^+^, 445.4 [M(^37^Cl) + H]^+^; HRMS calcd for C_21_H_21_^35^Cl_1_F_1_N_6_O_2_ [M + H]^+^ 443.193, found 443.193.

##### 4-(3,6-Dichloro-2-fluorobenzoyl)-*N*-(2-(4-methylpiperazin-1-yl)pyrimidin-5-yl)-1*H*-pyrrole-2-carboxamide (**32g**)

Prepared according to general procedure E using amine **7d** (160 mg, 0.83 mmol), carboxylic acid **31b** (100 mg, 0.33 mmol), cyanuric fluoride (20 μL, 0.23 mmol), pyridine (27 μL, 0.33 mmol), and MeCN (2 mL). Purification by MPLC on NH_2_ SiO_2_ with a gradient elution from 0 to 4% MeOH/CH_2_Cl_2_ gave a white solid (65 mg, 41%); *R*_f_ 0.65 (NH_2_ SiO_2_, 5% MeOH/EtOAc); mp 226–228 °C; λ_max_ (EtOH)/nm 287, 227; IR ν_max_/cm^–1^ 3174, 1663, 1639, 1592; ^1^H NMR (500 MHz; DMSO-*d*_6_) δ_H_ 2.25 (3H, s, CH_3_), 2.37–2.41 (4H, m, 2 × CH_2_-piperazine), 3.70–3.76 (4H, m, 2 × CH_2_-piperazine), 7.44 (1H, s, H-pyrrole), 7.56 (1H, dd, *J* = 1.1 and 8.6 Hz, H-5′), 7.64 (1H, s, H-pyrrole), 7.82 (1H, app t, *J* = 8.6 Hz, H-4′), 8.64 (2H, s, 2 × H-pyrimidine), 10.07 (1H, s, CO-NH), 12.78 (1H, s, NH); ^13^C NMR (125 MHz; DMSO-*d*_6_) δ_C_ 43.6 (2 × CH_2_-piperazine), 45.8 (CH_3_), 54.3 (2 × CH_2_-piperazine), 111.0 (CH-pyrrole), 119.3 (d, *J*_CF_ = 18.3 Hz, C-3′), 123.7 (C-pyrimidine), 124.7 (C-pyrrole), 126.9 (d, *J*_CF_ = 3.8 Hz, C-5′), 128.4 (C-pyrrole), 129.1 (d, *J*_CF_ = 22.3 Hz, C-1′), 129.2 (d, *J*_CF_ = 5.6 Hz, C-6′), 129.9 (CH-pyrrole), 131.8 (C-4′), 151.1 (2 × CH-pyrimidine), 153.8 (d, *J*_CF_ = 248.4 Hz, C-2′), 158.4 (C-pyrimidine), 158.4 (CO-NH), 182.6 (NH-pyrrole); ^19^F NMR (470 MHz; DMSO-*d*_6_) δ_F_ −116.7; MS (ES^+^) *m*/*z* 477.3 [M(^35,35^Cl) + H]^+^, 479.3 [M(^35,37^Cl) + H]^+^; HRMS calcd for C_21_H_20_^35^Cl_2_F_1_N_6_O_2_ [M + H]^+^ 477.1003, found 477.1008.

##### *tert*-Butyl-4-(5-Nitropyrimidin-2-yl)piperazine-1-carboxylate^33^ (**6e**)

Prepared according to general procedure F using 2-chloro-5-nitropyrimidine (350 mg, 2.20 mmol), 1-Boc-piperazine (450 mg, 2.40 mmol), Et_3_N (336 μL, 2.40 mmol), and THF (12 mL). The residue was purified by MPLC on SiO_2_ with a gradient elution from 15 to 100% EtOAc/petrol to give a yellow solid (460 mg, 68%); *R*_f_ 0.40 (SiO_2_, 20% EtOAc/petrol); mp 196–199 °C; λ_max_ (EtOH)/nm 339, 221; IR ν_max_/cm^–1^ 1676, 1539, 1325; ^1^H NMR (500 MHz; DMSO-*d*_6_) δ_H_ 1.47 (9H, s, C(CH_3_)_3_), 3.47–4.57 (4H, m, 2 × CH_2_-piperazine), 3.92–3.99 (4H, m, 2 × CH_2_-piperazine), 9.17 (2H, s, 2 × H-pyrimidine); ^13^C NMR (125 MHz; DMSO-*d*_6_) δ_C_ 28.0 (C(*C*H_3_)_3_), 42.5 (2 × CH_2_-piperazine), 43.9 (2 × CH_2_-piperazine), 79.3 (*C*(CH_3_)_3_), 133.4 (C-5-pyrimidine), 153.8 (CO), 155.1 (C-4 and C-6-pyrimidine), 161.0 (C-2-pyrimidine); HRMS calcd for C_13_H_20_N_5_O_4_ [M + H]^+^ 310.1510, found 310.1510.

##### *tert*-Butyl-4-(5-aminopyrimidin-2-yl)piperazine-1-carboxylate^34^ (**7e**)

Prepared according to general procedure D using nitropyrimidine **6e** (440 mg, 1.42 mmol) in MeOH (75 mL) and EtOAc (75 mL) to give a yellow solid (395 mg, 99%); *R*_f_ 0.15 (NH_2_ SiO_2_, 70% EtOAc/petrol); mp 131–133 °C; λ_max_ (EtOH)/nm 252; IR ν_max_/cm^–1^ 3336, 1676; ^1^H NMR (500 MHz; DMSO-*d*_6_) δ_H_ 1.45 (9H, s, C(CH_3_)_3_), 3.38–3.42 (4H, m, 2 × CH_2_-piperazine), 3.50–3.55 (4H, m, 2 × CH_2_-piperazine), 4.68 (2H, s, NH_2_), 7.94 (2H, s, 2 × H-pyrimidine); ^13^C NMR (125 MHz; DMSO-*d*_6_) δ_C_ 28.1 (C(*C*H_3_)_3_), 44.2 (2 × CH_2_-piperazine), 44.7 (2 × CH_2_-piperazine), 78.9 (*C*(CH_3_)_3_), 134.7 (C-5-pyrimidine), 144.0 (C-4 and C-6-pyrimidine), 154.0 (CO), 155.6 (C-2-pyrimidine); HRMS calcd for C_13_H_20_N_5_O_2_ [M – H]^−^ 278.1622, found 278.1609.

##### *tert*-Butyl-4-(5-(4-(3,6-dichloro-2-fluorobenzoyl)-1*H*-pyrrole-2-carboxamido)pyrimidin-2-yl)piperazine-1-carboxylate (**32h**)

Prepared according to general procedure E using amine **7e** (190 mg, 0.68 mmol), carboxylic acid **31b** (82 mg, 0.27 mmol), cyanuric fluoride (16 μL, 0.19 mmol), pyridine (22 μL, 0.27 mmol), and MeCN (4 mL). Purification by MPLC on NH_2_ SiO_2_ with a gradient elution from 40 to 100% EtOAc/petrol gave a white solid (105 mg, 69%); *R*_f_ 0.25 (NH_2_ SiO_2_, 70% EtOAc/petrol); mp 211–213 °C; λ_max_ (EtOH)/nm 283, 223; IR ν_max_/cm^–1^ 3199, 1637, 1573; ^1^H NMR (500 MHz; DMSO-*d*_6_) δ_H_ 1.46 (9H, s, C(CH_3_)_3_), 3.40–3.47 (4H, m, 2 × CH_2_-piperazine), 3.70–3.76 (4H, m, 2 × CH_2_-piperazine), 7.44 (1H, s, H-pyrrole), 7.56 (1H, d, *J* = 8.6 Hz, H-5′), 7.82 (1H, app t, *J* = 8.6 Hz, H-4′), 8.67 (2H, s, 2 × H-pyrimidine), 10.10 (1H, s, CO-NH), 12.79 (1H, s, NH); ^13^C NMR (125 MHz; DMSO-*d*_6_) δ_C_ 28.1 (C(*C*H_3_)_3_), 43.5 (4 × CH_2_-piperazine), 79.0 (C(CH_3_)_3_), 111.1 (CH-pyrrole), 119.3 (d, *J*_CF_ = 18.0 Hz, C-3′), 124.0 (C-pyrimidine), 124.7 (C-pyrrole), 126.9 (d, *J*_CF_ = 3.5 Hz, C-5′), 128.3 (C-1′), 128.3 (C-pyrrole), 129.2 (d, *J*_CF_ = 5.5 Hz, C-6′), 129.2 (CH-pyrrole), 131.8 (C-4′), 151.1 (2 × CH-pyrimidine), 153.8 (d, *J*_CF_ = 248.4 Hz, C-2′), 158.2 (C-pyrimidine), 158.4 (CO-NH), 182.6 (C–O); ^19^F NMR (470 MHz; DMSO-*d*_6_) δ_F_ −116.7; HRMS calcd for C_25_H_24_^35^Cl_2_F_1_N_6_O_4_ [M + H]^+^ 563.1197, found 563.121.

##### 4-(3,6-Dichloro-2-fluorobenzoyl)-*N*-(2-(piperazin-1-yl)pyrimidin-5-yl)-1*H*-pyrrole-2-carboxamide (**32k**)

Prepared according to general procedure J using Et_3_SiH (64 μL, 0.40 mmol), TFA (1 mL), CH_2_Cl_2_ (1 mL), and carbamate **32h** (90 mg, 0.16 mmol) to give a white solid (35 mg, 47%); *R*_f_ 0.2 (NH_2_ SiO_2_, 5% MeOH/EtOAc); mp 230 °C (dec.); λ_max_ (EtOH)/nm 303, 225; IR ν_max_/cm^–1^ 3275 (br), 2922, 2847, 1635; ^1^H NMR (500 MHz; DMSO-*d*_6_) δ_H_ 2.75–2.79 (4H, m, 2 × CH_2_-piperazine), 3.64–3.69 (4H, m, 2 × CH_2_-piperazine), 7.42 (1H, s, H-pyrrole), 7.55 (1H, d, *J* = 8.6 Hz, H-5′), 7.63 (1H, s, H-pyrrole), 7.82 (1H, app t, *J* = 8.6 Hz, H-4′), 8.62 (2H, s, 2 × H-pyrimidine), 10.05 (1H, br s, CO-N*H*); ^13^C NMR (125 MHz; DMSO-*d*_6_) δ_C_ 44.9 (2 × C-piperazine), 45.4 (2 × C-piperazine), 111.0 (CH-pyrrole), 119.3 (d, *J*_CF_ = 18.1 Hz, C-3′), 123.4 (C-pyrimidine), 124.7 (C-pyrrole), 126.9 (d, *J*_CF_ = 3.6 Hz), 128.4 (C-pyrrole), 129.2 (d, *J*_CF_ = 23.2 Hz, C-1′), 129.2, (d, *J*_CF_ = 5.3 Hz, C-6′), 129.9 (CH-pyrrole), 131.8 (C-4′), 151.1 (2 × CH-pyrimidine), 153.8 (d, *J*_CF_ = 248.7 Hz, C-2′), 158.4 (C-pyrimidine), 158.6 (CO-NH), 182.6 (CO); HRMS calcd for C_20_H_18_^35^Cl_2_F_1_N_6_O_2_ [M + H]^+^ 463.0847, found 463.0853.

##### *N*-(1-Methylpiperidin-4-yl)-5-nitropyrimidin-2-amine^35^ (**6f**)

Prepared according to general procedure F using 2-chloro-5-nitropyrimidine (300 mg, 1.90 mmol), 4-amino-1-methylpiperidine (259 μL, 2.10 mmol), Et_3_N (288 μL, 2.10 mmol), and THF (10 mL) to give a yellow solid (370 mg, 83%); *R*_f_ 0.50 (NH_2_ SiO_2_, EtOAc); mp 154–157 °C; λ_max_ (EtOH)/nm 340, 213; IR ν_max_/cm^–1^ 3242, 1587, 1329; ^1^H NMR (500 MHz; DMSO-*d*_6_) δ_H_ 1.61 (2H, qd, *J* = 3.5 and 11.9 Hz, 2 × H-piperidine), 1.80–1.89 (2H, m, 2 × H-piperidine), 1.93–2.03 (2H, m, 2 × H-piperidine), 2.20 (3H, s, CH_3_), 2.77–2.84 (2H, m, 2 × H-piperidine), 3.80–3.90 (1H, m, C*H*-NH), 8.83 (1H, d, *J* = 8.4 Hz, NH), 9.07 (1H, d, *J* = 3.4 Hz, H-pyrimidine), 9.13 (1H, d, *J* = 3.4 Hz, H-pyrimidine); ^13^C NMR (125 MHz; DMSO-*d*_6_) δ_C_ 30.9 (2 × CH_2_-piperidine), 45.9 (N-CH_3_), 48.4 (CH-NH), 54.1 (2 × CH_2_-N-piperidine), 133.5 (C-5-pyrimidine), 155.2 (CH-pyrimidine), 155.4 (CH-pyrimidine), 162.1 (C-2-pyrimidine); HRMS calcd for C_10_H_16_N_5_O_2_ [M + H]^+^ 238.1299, found 238.1301.

##### *N*^2^-(1-Methylpiperidin-4-yl)pyrimidine-2,5-diamine^35^ (**7f**)

Prepared according to general procedure D using nitropyrimidine **6f** (360 mg, 1.52 mmol) and MeOH (30 mL) for 2 h to give a pale yellow solid (290 mg, 92%); *R*_f_ 0.50 (NH_2_ SiO_2_, 5% MeOH/EtOAc); mp 158–161 °C; λ_max_ (EtOH)/nm 248; IR ν_max_/cm^–1^ 3257, 2967, 2789; ^1^H NMR (500 MHz; DMSO-*d*_6_) δ_H_ 1.45 (2H, qd, *J* = 3.6 and 11.7 Hz, 2 × H-piperidine), 1.78–1.86 (2H, m, 2 × H-piperidine), 1.90–1.99 (2H, m, 2 × H-piperidine), 2.17 (3H, s, CH_3_), 2.70–2.77 (2H, m, 2 × H-piperidine), 3.46–3.56 (1H, m, C*H*-NH), 4.41 (2H, s, NH_2_), 6.02 (1H, d, *J* = 8.0 Hz, NH), 7.82 (2H, s, 2 × H-pyrimidine); ^13^C NMR (125 MHz; DMSO-*d*_6_) δ_C_ 31.8 (2 × CH_2_-piperidine), 46.1 (N-CH_3_), 47.5 (CH-NH), 54.6 (2 × CH_2_N-piperidine), 133.5 (C-5 pyrimidine), 144.6 (2 × CH-pyrimidine), 156.0 (C-2 pyrimidine); HRMS calcd for C_10_H_18_N_5_ [M + H]^+^ 208.1557, found 208.1559.

##### 4-(3,6-Dichloro-2-fluorobenzoyl)-*N*-(2-((1-methylpiperidin-4-yl)amino)pyrimidin-5-yl)-1*H*-pyrrole-2-carboxamide (**32i**)

Prepared according to general procedure E using amine **7f** (150 mg, 0.72 mmol), carboxylic acid **31b** (88 mg, 0.29 mmol), cyanuric fluoride (21 μL, 0.24 mmol), pyridine (23 μL, 0.29 mmol), and MeCN (2 mL). Purification by MPLC on NH_2_ SiO_2_ with a gradient elution from 1 to 7% MeOH/EtOAc gave a white solid (50 mg, 35%); *R*_f_ 0.20 (NH_2_ SiO_2_, 5% MeOH/EtOAc); mp 275 °C dec.; λ_max_ (EtOH)/nm 283, 226; IR ν_max_/cm^–1^ 3163, 1642, 1593; ^1^H NMR (500 MHz; DMSO-*d*_6_) δ_H_ 1.54 (2H, qd, *J* = 3.1 and 11.5 Hz, 2 × H-piperidine), 1.81–1.89 (2H, m, 2 × H-piperidine), 1.92–2.02 (2H, m, 2 × H-piperidine), 2.19 (3H, s, CH_3_), 2.73–2.81 (2H, m, 2 × H-piperidine), 3.61–3.72 (1H, m, C*H*-NH-piperidine), 7.02 (1H, d, *J* = 7.8 Hz, NH-piperidine), 7.42 (1H, s, H-pyrrole), 7.56 (1H, d, *J* = 8.6 Hz, H-5′), 7.63 (1H, s, H-pyrrole), 7.82 (1H, app t, *J* = 8.6 Hz, H-4′), 8.51 (2H, s, 2 × H-pyrimidine), 9.98 (1H, s, CO-NH), 12.74 (1H, br s, NH-pyrrole); ^13^C NMR (125 MHz; DMSO-*d*_6_) δ_C_ 31.5 (2 × CH_2_-piperidine), 46.0 (CH_3_), 48.6 (CH-NH-piperidine), 54.5 (2 × CH_2_N-piperidine), 110.9 (CH-pyrrole), 119.3 (d, *J*_CF_ = 18.0 Hz, C-3′), 123.2 (C-pyrimidine), 124.7 (C-pyrrole), 126.9 (d, *J*_CF_ = 3.6 Hz, C-5′), 128.5 (C-pyrrole), 128.8 (CH-pyrrole), 129.2 (d, *J*_CF_ = 22.3 Hz, C-1′), 129.2 (d. *J*_CF_ = 5.1 Hz, C-6′), 131.8 (C-4′), 151.6 (2 × CH-pyrimidine), 153.8 (d, *J*_CF_ = 248.9 Hz, C-2′), 158.4 (CO-NH), 158.9 (C-pyrimidine), 182.6 (CO); ^19^F NMR (470 MHz; DMSO-*d*_6_) δ_F_ −116.7; HRMS calcd for C_22_H_21_^35^Cl_2_F_1_N_6_O_2_ [M + H]^+^ 491.1160, found 491.1150.

##### 2,2-Diethoxyacetimidamide Hydrochloride^36^ (**12**)

Sodium (8 mg, 0.36 mmol) was carefully added to MeOH (5 mL) at RT, and the mixture was stirred under nitrogen until the sodium had dissolved. Diethoxyacetonitrile (**11**) (1.0 mL, 0.93 g, 7.19 mmol) was added, and the resulting mixture was stirred at RT for 16 h. Solid carbon dioxide was added, and the solvent was removed *in vacuo*. The resulting oil was dissolved in Et_2_O (10 mL) and filtered. The filtrate was concentrated *in vacuo* to afford methyl diethoxyacetimidate as a yellow oil, which was used without further purification. The oil was dissolved in MeOH (5 mL), and ammonium chloride (385 mg, 7.19 mmol) was added in one portion. The resulting solution was stirred at RT overnight before the solvent was concentrated *in vacuo*. The resulting oil was triturated with Et_2_O to afford diethoxyacetimidamide hydrochloride as an off-white solid (1.17 g, 89%). The compound was used in the next step without further purification; mp 58.0–60.0 °C (lit. 81.0–82.0 °C);^37^ IR ν_max_/cm^–1^ 3259, 3042, 2975, 1692, 1083; ^1^H NMR (500 MHz, DMSO-*d*_6_) δ 1.19 (6H, t, *J* = 7.0 Hz, OCH_2_C*H*_3_), 3.62 (4H, qd, *J* = 7.0, 3.0 Hz, OC*H*_2_CH_3_), 5.29 (1H, s, C*H*(OEt)_2_), 9.05 (4H, s, NH_2_ and NH_2_^+^); ^13^C NMR (126 MHz, DMSO-*d*_6_) δ 14.9 (OCH_2_*C*H_3_), 63.1 (O*C*H_2_CH_3_), 95.7 (*C*H(OEt)_2_), 165.8 (*C* = NH(NH_2_)); HRMS (ESI) calcd for C_6_H_15_N_2_O_2_ [M – Cl]^+^ 147.1128, found 147.1123; ^1^H NMR data were identical to literature data.

##### *N*-(3-(Dimethylamino)-2-[[(dimethylamino)methylene]amino]prop-2-en-1-ylidene)-*N*-methylmethanaminium Hydrogen Dihexafluorophosphate^38^

Phosphorus (V) oxychloride (7.52 mL, 12.4 g, 80.7 mmol) was added dropwise to DMF (16.1 mL) cooled to 10 °C, maintaining the temperature of the solution between 10 and 15 °C during the addition. Once the addition was complete, the reaction was stirred at RT for 20 min. The resulting solution was cooled to 5 °C before powdered glycine hydrochloride (3.00 g, 26.9 mmol) was added in portions; the temperature of the reaction mixture was maintained below 10 °C during the addition. The resulting reaction mixture was heated at 80 °C for 4 h. The hot, dark orange, solution was carefully poured directly into water (43 mL), precooled to 5 °C. The temperature of the solution was kept below 20 °C. Five minutes after the transfer was complete, the reaction mixture was cooled to −5 °C and treated from a plastic vessel with 60% aqueous hexafluorophosphoric acid (7.93 mL, 53.8 mmol). The thick precipitate was collected by filtration and washed with cold EtOH (100 mL) until a pale yellow solid was obtained (5.24 g, 40%); mp 151.0–153.0 °C; λ_max_ (EtOH)/nm 254.6; IR ν_max_/cm^–1^ 1701, 1611, 1402, 1291; ^1^H NMR (500 MHz, DMSO-*d*_6_) δ 3.19 (9H, s, 3 × NC*H*_3_), 3.24 (3H, s, NC*H*_3_), 3.29 (6H, s, 2 × NC*H*_3_), 7.70 (2H, s, 2 × CH), 8.07 (1H, d, *J* = 10.4 Hz, C*H*NH(CH_3_)_2_^+^), 10.74 (1H, d, *J* = 6.8 Hz, CHN*H*(CH_3_)_2_^+^); ^13^C NMR (126 MHz, DMSO-*d*_6_) δ 37.0 (N*C*H_3_), ca. 40 (overlapping with DMSO) (N*C*H_3_), 43.6 (N*C*H_3_), 48.8 (N*C*H_3_), 100.8 (C_q_), 158.1 (*C*H), 160.7 (*C*HNH(CH_3_)_2_^+^); ^19^F NMR (471 MHz, DMSO-*d*_6_) δ −70.9 (PF_6_^–^), −69.4 (PF_6_^–^); MS (ES^+^) *m*/*z* 197.3 [M-HP_2_F_12_^–^]^+^; HRMS (NSI) calcd for C_10_H_21_N_4_ [M-HP_2_F_12_^–^]^+^ 197.1761, found 197.1760.

##### 2-(Diethoxymethyl)pyrimidin-5-amine^38^ (**13**)

To a slurry of *N*-(3-(dimethylamino)-2-[[(dimethylamino)methylene]amino]prop-2-en-1-ylidene)-*N*-methylmethanaminium hydrogen dihexafluorophosphate (5.70 g, 11.7 mmol) and 2,2-diethoxyacetimidamide hydrochloride (**12**) (2.56 g, 14.0 mmol) in EtOH (25 mL) was added dropwise a solution of NaOMe in MeOH (2.27 g, 42.0 mmol, 25% w/v); the mixture was heated to reflux halfway through the addition. After refluxing for 2.5 h, the mixture was cooled to 0 °C, the inorganic precipitate was filtered off, washed with cold EtOH (3 × 20 mL), and the filtrate was concentrated *in vacuo*. The residue was dissolved in CH_2_Cl_2_ (50 mL), washed with water (3 × 20 mL), dried over MgSO_4_, and concentrated *in vacuo* to give an orange oil. The oil was dissolved in 1,4-dioxane (20 mL), treated with 5% aqueous K_2_CO_3_ (30 mL), and heated to reflux overnight. The reaction mixture was cooled to RT and extracted with EtOAc (3 × 40 mL). The organic extracts were washed with water (50 mL) and brine (50 mL), dried over MgSO_4_, and concentrated *in vacuo*. The crude product was purified by MPLC on SiO_2_ with a gradient elution from 0 to 5% MeOH/CH_2_Cl_2_ to yield an off-white solid (1.12 g, 49%); *R*_f_ 0.25 (SiO_2_, 5% MeOH/CH_2_Cl_2_); mp 134.0–136.0 °C; λ_max_ (EtOH)/nm 250.4, 315.4; IR ν_max_/cm^–1^ 3358, 3324, 3202, 2977, 2929, 2877, 1646, 1583, 1555, 1452; ^1^H NMR (500 MHz, DMSO-*d*_6_) δ 1.09 (6H, t, *J* = 7.1 Hz, OCH_2_C*H*_3_), 3.49 (2H, dq, *J* = 9.7, 7.1 Hz, OC*H*_2_CH_3_), 3.61 (2H, dq, *J* = 9.7, 7.1 Hz, OC*H*_2_CH_3_), 5.27 (1H, s, ArC*H*(OEt)_2_), 5.60 (2H, brs, ArN*H*_2_), 8.07 (2H, s, H-4, 6); ^13^C NMR (126 MHz, DMSO-*d*_6_) δ 15.2 (OCH_2_*C*H_3_), 61.2 (O*C*H_2_CH_3_), 102.0 (Ar*C*H(OEt)_2_), 141.1 (C-4, 6), 142.1 (C-5), 153.5 (C-2); MS (ES^+^) *m*/*z* 198.2 [M + H]^+^; HRMS (NSI) calcd for C_9_H_15_N_3_O_2_Na [M + Na]^+^ 220.1056, found 220.1052; ^1^H and ^13^C NMR data were identical to literature data.^38^

##### Benzyl-(2-(diethoxymethyl)pyrimidin-5-yl)carbamate (**14**)

To 2-(diethoxymethyl)pyrimidin-5-amine (**13**) (1.50 g, 7.60 mmol) in THF/H_2_O (1:1) (20 mL) was added K_2_CO_3_ (2.10 g, 15.2 mmol) in one portion, followed by the dropwise addition of benzyl chloroformate (2.17 mL, 15.2 mmol) in THF (5 mL). The resulting reaction mixture was stirred at RT for 24 h. The reaction mixture was diluted with water (20 mL) and extracted with EtOAc (3 × 40 mL). The combined organic extracts were washed with water (40 mL) and brine (40 mL), dried over MgSO_4_, and concentrated *in vacuo*. The crude product was purified MPLC on SiO_2_ with a gradient elution from 0 to 40% EtOAc/petrol to yield a clear oil (2.03 g, 80%); *R*_f_ 0.32 (SiO_2_, 40% EtOAc/petrol); λ_max_ (EtOH)/nm 238.0; IR ν_max_/cm^–1^ 3227, 3032, 2975, 2933, 2882, 1728, 1586, 1525, 1224; ^1^H NMR (500 MHz, DMSO-*d*_6_) δ 1.11 (6H, t, *J* = 7.0 Hz, 2 × OCH_2_C*H*_3_), 3.54 (2H, dq, *J* = 9.6, 7.0 Hz, OC*H*_2_CH_3_), 3.65 (2H, dq, *J* = 9.6, 7.0 Hz, OC*H*_2_CH_3_), 5.20 (2H, s, OC*H*_2_Ph), 5.42 (1H, s, ArC*H*(OEt)_2_), 7.33–7.47 (5H, m, 5 × Ar*H*), 8.88 (2H, s, H-4, 6), 10.26 (1H, s, ArN*H*Cbz); ^13^C NMR (126 MHz, DMSO-*d*_6_) δ 15.2 (OCH_2_*C*H_3_), 61.6 (O*C*H_2_CH_3_), 66.5 (O*C*H_2_Ph), 101.7 (Ar*C*H(OEt)_2_), 128.2 (CH-Ar), 128.5 (CH-Ar), 133.7 (C-Ar), 136.1 (C-Ar), 146.2 (C-4, 6), 153.4 (ArNH*C*O_2_Bn), 159.3 (C-2); MS (ES^–^) *m*/*z* 330.3 [M – H]^−^; HRMS (NSI) calcd for C_17_H_21_N_3_O_4_ [M + H]^+^ 332.1605, found 332.1600.

##### Benzyl-(2-formylpyrimidin-5-yl)carbamate (15)

To benzyl (2-(diethoxymethyl)pyrimidin-5-yl)carbamate (**14**) (1.90 g, 5.73 mmol) in MeCN (20 mL) was added 1 M hydrochloric acid (3.50 mL) at RT. The resulting mixture was stirred at RT for 8 h. The solvents were removed *in vacuo*, and the white residue was dissolved in saturated aqueous NaHCO_3_ (20 mL). The aqueous layer was extracted with EtOAc (3 × 30 mL), and the organic extracts were washed with water (40 mL) and brine (40 mL), dried over MgSO_4_, and concentrated *in vacuo* to give a white solid (1.31 g, 89%). The crude material was used in the next step without further purification; *R*_f_ 0.31 (SiO_2_, 50% petrol/EtOAc); mp 166.5–168.5 °C; λ_max_ (EtOH)/nm 283.4; IR ν_max_/cm^–1^ 3217, 3062, 3033, 2964, 2876, 1730, 1715, 1586, 1566, 1526; ^1^H NMR (500 MHz, DMSO-*d*_6_) δ 5.24 (2H, s, OC*H*_2_Ph), 7.34–7.49 (5H, m, 5 × Ar*H*), 9.10 (2H, s, H-4, 6), 9.89 (1H, s, ArC*H*O), 10.69 (1H, s, ArN*H*Cbz); ^13^C NMR (126 MHz, DMSO-*d*_6_) δ 66.9 (O*C*H_2_Ph), 128.3 (CH), 128.5 (CH), 135.8 (C-Ar), 136.2 (C-Ar), 146.0 (C-4, 6), 153.2, 153.6, 190.4 (Ar*C*HO); MS (ES^+^) *m*/*z* 258.2 [M + H]^+^; HRMS (NSI) calcd for C_13_H_12_N_3_O_3_ [M + H]^+^ 258.0873, found 258.0875.

##### *tert*-Butyl-4-((5-(((benzyloxy)carbonyl)amino)pyrimidin-2-yl)methyl)piperazine-1-carboxylate (**16**)

To benzyl(2-formylpyrimidin-5-yl)carbamate (**15**) (900 mg, 3.50 mmol) in tetrafluoroethylene (TFE) (25 mL) was added *tert*-butyl piperazine-1-carboxylate (1.30 g, 7.00 mmol). The resulting solution was stirred at 38 °C for 1 h. The reaction mixture was cooled at 0 °C, and sodium borohydride was added portionwise. The resulting mixture was allowed to warm to RT and stirred for 30 min. The solvent was removed *in vacuo*, and the crude residue was dissolved in EtOAc (40 mL), neutralized by washing with saturated aqueous NH_4_Cl (25 mL) and washed with water (20 mL) and brine (20 mL), dried over MgSO_4_, and concentrated *in vacuo*. The crude product was purified by MPLC on NH_2_ SiO_2_ with a gradient elution from 0 to 60% EtOAc/petrol to yield a yellow solid (630 mg, 42%); *R*_f_ 0.34 (NH_2_ SiO_2_, 40% petrol/EtOAc); λ_max_ (EtOH)/nm 236.8 nm; IR ν_max_/cm^–1^ 2974, 1684, 1591, 1528, 1416; ^1^H NMR (500 MHz, DMSO-*d*_6_) δ 1.38 (9H, s, C(C*H*_3_)_3_), 2.35–2.45 (4H, m, CH_2 piperazine_), 3.28 (4H, s, CH_2 piperazine_), 3.65 (2H, s, ArC*H*_2_N), 5.19 (2H, s, OC*H*_2_Ph), 7.33–7.46 (5H, m, 5 × Ar*H*), 8.83 (2H, s, H-4, 6), 10.17 (1H, s, ArN*H*Cbz); ^13^C NMR (126 MHz, DMSO-*d*_6_) δ 28.0 (C(*C*H_3_)_3_), 43.1 (CH_2 piperazine_), 52.3 (CH_2 piperazine_), 63.6 (Ar*C*H_2_N), 66.4 (O*C*H_2_Ph), 78.7 (O*C*(CH_3_)_3_), 128.2 (CH-Ar), 128.2 (CH-Ar), 128.5 (CH-Ar), 132.7 (C-Ar), 136.1 (C-Ar), 146.3 (C-4, 6), 153.4, 153.8, 160.4 (C-2); MS (ES^+^) *m*/*z* 428.5 [M + H]^+^; HRMS (NSI) calcd for C_22_H_30_N_5_O_4_ [M + H]^+^ 428.2292, found 428.2288.

##### *tert*-Butyl-4-((5-aminopyrimidin-2-yl)methyl)piperazine-1-carboxylate (**17**)

*tert*-Butyl-4-((5-(((benzyloxy)carbonyl)amino)pyrimidin-2-yl)methyl)piperazine-1-carboxylate (**16**) (600 mg, 1.40 mmol) in EtOAc (28 mL) was subjected to palladium-catalyzed hydrogenation using an H-Cube reactor and a 10% Pd/C CatCart under a full pressure of hydrogen at RT for 24 h with continuous recycling of the reaction mixture at 1 mL/min flow rate. The reaction mixture was concentrated *in vacuo* to afford a pale yellow solid (407 mg, 99%), which was used in the next step without further purification; *R*_f_ 0.26 (NH_2_ SiO_2_, 3% MeOH/CH_2_Cl_2_); mp 200.5–202.5 °C; λ_max_ (EtOH)/nm 249.0, 318.0; IR ν_max_/cm^–1^ 3381, 3323, 3197, 2972, 2929, 2894, 2863, 2811, 2775, 1673, 1589, 1554, 1453; ^1^H NMR (500 MHz, DMSO-*d*_6_) δ 1.37 (9H, s, C(C*H*_3_)_3_), 2.35 (4H, t, *J* = 5.0 Hz, CH_2 piperazine_), 3.26 (4H, brs, CH_2 piperazine_), 3.50 (2H, s, ArC*H*_2_N), 5.47 (2H, brs, ArN*H*_2_), 8.05 (2H, s); ^13^C NMR (126 MHz, DMSO-*d*_6_) δ 28.1 (C(*C*H_3_)_3_), 43.3 (CH_2 piperazine_), 52.3 (CH_2 piperazine_), 63.8 (Ar*C*H_2_N), 78.7 (O*C*(CH_3_)_3_), 141.1, 141.5, 153.8, 154.1; MS (ES^+^) *m*/*z* 294.3 [M + H]^+^; HRMS (NSI) calcd for C_14_H_24_N_5_O_2_ [M + H]^+^ 294.1925, found 294.1926.

##### *tert*-Butyl-4-((5-(4-(3,6-dichloro-2-fluorobenzoyl)-1*H*-pyrrole-2-carboxamido)pyrimidin-2-yl)methyl)piperazine-1-carboxylate (**32j**)

Compound **32j** was synthesized according to general procedure I using 4-(3,6-dichloro-2-fluorobenzoyl)-1*H*-pyrrole-2-carboxylic acid **31b** (200 mg, 0.66 mmol), triethylamine (231 μL, 167 mg, 1.65 mmol), 2-chloro-1-methylpyridinium iodide (186 mg, 0.73 mmol), *tert*-butyl 4-((5-aminopyrimidin-2-yl)methyl)piperazine-1-carboxylate (**17**) (243 mg, 1.65 mmol), and CH_2_Cl_2_ (6.60 mL). The crude yellow solid was purified by MPLC on SiO_2_ with a gradient elution from 0 to 85% EtOAc/petrol to yield a white solid (160 mg, 42%); *R*_f_ 0.32 (SiO_2_, 15% petrol/EtOAc); mp 162.5–164.5 °C; λ_max_ (EtOH)/nm 292.8; IR ν_max_/cm^–1^ 2967, 2932, 2864, 2815, 1652, 1585, 1555, 1516, 1447, 1423, 1392; ^1^H NMR (500 MHz, DMSO-*d*_6_) δ 1.38 (9H, s, C(C*H*_3_)_3_), 2.43 (4H, t, *J* = 5.1 Hz, CH_2 piperazine_), 3.29 (4H, brs, CH_2 piperazine_), 3.69 (2H, s, ArC*H*_2_N), 7.51 (1H, s), 7.53 (1H, dd, *J* = 8.9, 1.4 Hz), 7.68 (1H, s), 7.79 (1H, dd, *J* = 8.9, 8.4 Hz), 9.08 (2H, s), 10.43 (1H, s, CON*H*Ar), 12.88 (1H, s, NH-pyrrole); ^13^C NMR (126 MHz, DMSO-*d*_6_) δ 28.1 (C(*C*H_3_)_3_), 43.2 (CH_2 piperazine_), 52.3 (CH_2 piperazine_), 63.7 (Ar*C*H_2_N), 78.7 (O*C*(CH_3_)_3_), 111.9 (C-3), 119.4 (d, *J* = 18.2 Hz), 124.9, 126.9 (d, *J* = 3.9 Hz), 128.0, 129.1 (d, *J* = 23.1 Hz), 129.2 (d, *J* = 5.1 Hz), 130, 131.9, 132.5, 147.9, 153.8 (*C*O_2_N), 153.9 (d, *J* = 248.5 Hz), 158.7 (*C*ONHAr), 161.1, 182.6 (ArCO); ^19^F NMR (471 MHz, DMSO-*d*_6_) δ −116.7 (ArF); HRMS (NSI) calcd for C_26_H_28_Cl_2_FN_6_O_4_ [M(^35^Cl^35^Cl) + H]^+^ 577.1528, found 577.1521.

##### 4-(3,6-Dichloro-2-fluorobenzoyl)-*N*-(2-(piperazin-1-ylmethyl)pyrimidin-5-yl)-1*H*-pyrrole-2-carboxamide (**32l**)

Compound **32l** was synthesized according to general procedure J using *tert*-butyl 4-((5-(4-(3,6-dichloro-2-fluorobenzoyl)-1*H*-pyrrole-2-carboxamido)pyrimidin-2-yl)methyl)piperazine-1-carboxylate (**32j**) (60 mg, 0.10 mmol), triethylsilane (41 μL, 30 mg, 0.26 mmol), TFA (0.5 mL), and CH_2_Cl_2_ (0.5 mL). The crude yellow solid was purified by MPLC on NH_2_ SiO_2_ with a gradient elution from 0 to 6% MeOH/CH_2_Cl_2_ to yield an off-white solid (35 mg, 70%); *R*_f_ 0.29 (NH_2_ SiO_2_, 6% MeOH/CH_2_Cl_2_); mp 239.5–241.5 °C; λ_max_ (EtOH)/nm 266.0, 293.2; IR ν_max_/cm^–1^ 3069, 2932, 2812, 1639, 1581, 1558, 1510, 1444, 1389, 1268; ^1^H NMR (500 MHz, DMSO-*d*_6_) δ 2.41 (4H, brs, NC*H*_2 piperazine_), 2.70 (4H, t, *J* = 4.9 Hz, NC*H*_2 piperazine_), 3.63 (2H, s, ArC*H*_2_N), 7.48 (1H, s, H-3), 7.52 (1H, dd, *J* = 8.7, 1.4 Hz, H-5′), 7.66 (1H, s, H-5), 7.78 (1H, dd, *J* = 8.7, 8.4 Hz, H-4′), 9.08 (2H, s, H-4″, 6″), 10.41 (1H, s, CON*H*Ar); ^13^C NMR (126 MHz, DMSO-*d*_6_) δ 45.3 (N*C*H_2 piperazine_), 53.6 (N*C*H_2 piperazine_), 64.5 (Ar*C*H_2_N), 112.0 (C-3), 119.3 (d, *J* = 17.9 Hz, C-3′), 124.9 (C-2 or C-4), 126.9 (d, *J* = 3.8 Hz, C-5′), 128.3 (C-2 or C-4), 129.2 (d, *J* = 23.1 Hz, C-1′), 129.2 (d, *J* = 5.1 Hz, C-6′), 130.7 (C-5), 131.9 (C-4′), 132.4 (C-5″), 147.8 (C-4″, 6″), 153.8 (d, *J* = 248.5 Hz, C-2′), 158.9 (*C*ONHAr), 161.3 (C-2″), 182.5 (ArCO); ^19^F NMR (471 MHz, DMSO-*d*_6_) δ −116.7 (ArF); MS (ES^+^) *m*/*z* 473.3 [M(^35^Cl^35^Cl) + H]^+^, 475.3 [M(^35^Cl^37^Cl) + H]^+^; HRMS (NSI) calcd for C_21_H_20_Cl_2_FN_6_O_2_ [M(^35^Cl^35^Cl) + H]^+^ 477.1003, found 477.0999.

##### 4-(3,6-Dichloro-2-fluorobenzoyl)-*N*-(2-((4-methylpiperazin-1-yl)methyl)pyrimidin-5-yl)-1*H*-pyrrole-2-carboxamide (**32m**)

Compound **32m** was synthesized according to general procedure H using *tert*-butyl 4-((5-(4-(3,6-dichloro-2-fluorobenzoyl)-1*H*-pyrrole-2-carboxamido)pyrimidin-2-yl)methyl)piperazine-1-carboxylate (**32j**) (60 mg, 0.10 mmol), formic acid (0.5 mL), and formaldehyde (37% wt in water) (31 μL, 0.42 mmol). The crude yellow solid was purified by MPLC on NH_2_ SiO_2_ with a gradient elution from 0 to 6% MeOH/CH_2_Cl_2_ to yield a white solid (36 mg, 71%); *R*_f_ 0.30 (NH_2_ SiO_2_, 4% MeOH/CH_2_Cl_2_); mp 183.0–185.0 °C; λ_max_ (EtOH)/nm 293.0; IR ν_max_/cm^–1^ 2935, 2802, 1641, 1581, 1557, 1515, 1447, 1280; ^1^H NMR (500 MHz, DMSO-*d*_6_) δ 2.13 (3H, s, NC*H*_3_), 2.29 (4H, brs, NC*H*_2 piperazine_), 2.47 (4H, brs, NC*H*_2 piperazine_), 3.64 (2H, s, ArC*H*_2_N), 7.50 (1H, s, H-3), 7.52 (1H, dd, *J* = 8.8, 1.4 Hz, H-5′), 7.67 (1H, s, H-5), 7.79 (1H, dd, *J* = 8.8, 8.4 Hz, H-4′), 9.07 (2H, s, H-4″, 6″), 10.41 (1H, s, CON*H*Ar), 12.85 (1H, s, NH-pyrrole); ^13^C NMR (126 MHz, DMSO-*d*_6_) δ 45.7 (N*C*H_3_), 52.5 (N*C*H_2 piperazine_), 54.7 (N*C*H_2 piperazine_), 63.9 (Ar*C*H_2_N), 111.9 (C-3), 119.4 (d, *J* = 18.1 Hz, C-3′), 124.8 (C-2 or C-4), 126.9 (d, *J* = 3.8 Hz, C-5′), 128.0 (C-2 or C-4), 129.1 (d, *J* = 23.2 Hz, C-1′), 129.2 (d, *J* = 5.4 Hz, C-6′), 130.5 (C-5), 131.9 (C-4′), 132.4 (C-5″), 147.9 (C-4″, 6″), 153.8 (d, *J* = 248.7 Hz, C-2′), 158.7 (*C*ONHAr), 161.4 (C-2″), 182.6 (ArCO); ^19^F NMR (471 MHz, DMSO-*d*_6_) δ −116.7 (ArF); MS (ES^+^) *m*/*z* 491.4 [M(^35^Cl^35^Cl) + H]^+^, 493.4 [M(^35^Cl^37^Cl) + H]^+^; HRMS (NSI) calcd for C_22_H_22_Cl_2_FN_6_O_2_ [M(^35^Cl^35^Cl) + H]^+^ 491.1160, found 491.1154.

##### *tert*-Butyl-4-(5-nitropyridin-2-yl)piperazine-1-carboxylate^39^ (**9a**)

Prepared according to general procedure F using *N*^1^*-*Boc*-*piperazine (2.35 g, 12.7 mmol), 2-chloro-5-nitropyridine (1.00 g, 6.3 mmol), and K_2_CO_3_ (1.75 g, 12.7 mmol) in THF (10 mL) for 72 h to give an orange oil (1.85 g, 97%); *R*_f_ 0.5 (SiO_2_, 50% EtOAc/petrol); mp168–170 °C (Lit.^40^ 169 °C); λ_max_ (EtOH)/nm 358, 228; IR ν_max_/cm^–1^ 1688, 1594, 1338; ^1^H NMR (500 MHz; DMSO-*d*_6_) δ_H_ 1.46 (9H, s, C(C*H*_3_)_3_), 3.46–3.52 (4H, m, 4 × H-piperazine), 3.78–3.83 (4H, m, 4 × H-piperazine), 6.97 (1H, d, *J* = 9.6 Hz, H-3-pyridine), 8.29 (1H, dd, *J* = 2.8 and 9.6 Hz, H-4-pyridine), 9.01 (1H, d, *J* = 2.8 Hz, H-6-pyridine); ^13^C NMR (125 MHz; DMSO-*d*_6_) δ_C_ 28.0 (C(*C*H_3_)_3_), 44.1 (C-piperazine), 79.2 (*C*(*C*H_3_)_3_), 105.7 (C-3-pyridine), 132.9 (C-4-pyridine), 134.4 (C-5-pyridine), 146.0 (C-6-pyridine), 153.8 (C-2-pyridine), 160.1 (CO); HRMS calcd for C_14_H_21_N_4_O_4_ [M + H]^+^ 309.1557, found 309.1558.

##### *tert*-Butyl-4-(5-aminopyridin-2-yl)piperazine-1-carboxylate^41^ (10a)

Prepared according to general procedure D using nitropyridine **9a** (1.83 g, 5.9 mmol), MeOH (60 mL), and EtOAc (60 mL) for 24 h to give a beige solid (1.65 g, 100%); *R*_f_ 0.65 (NH_2_ SiO_2_, EtOAc); mp 109 °C (dec.); λ_max_ (EtOH)/nm 255; IR ν_max_/cm^–1^ 3382, 3321, 2975.8, 2820, 1685; ^1^H NMR (500 MHz; DMSO-*d*_6_) δ_H_ 1.45 (9H, s, C(C*H*_3_)_3_), 3.19–3.25 (4H, m, 4 × H-piperazine), 3.40–3.47 (4H, m, 4 × H-piperazine), 4.64 (2H, br s, NH_2_), 6.68 (1H, d, *J* = 8.7 Hz, H-3-pyridine), 6.96 (1H, dd, *J* = 2.9 and 8.7 Hz, H-4-pyridine), 7.64 (1H, d, *J* = 2.9 Hz, H-6-pyridine); ^13^C NMR (125 MHz; DMSO-*d*_6_) δ_C_ 28.1 (C(*C*H_3_)_3_), 43.4 (C-piperazine), 46.3 (C-piperazine), 78.8 (*C*(*C*H_3_)_3_), 108.8 (C-3-pyridine), 124.4 (C-4-pyridine), 133.3 (C-5-pyridine), 137.6 (C-6-pyridine), 152.0 (C-2-pyridine), 153.9 (CO-carbamate); HRMS calcd for C_14_H_21_N_4_O_2_ [M – H]^−^ 277.1670, found 277.1666.

##### *tert*-Butyl-4-(5-(4-(3,6-dichloro-2-fluorobenzoyl)-1*H*-pyrrole-2-carboxamido)pyridin-2-yl)piperazine-1-carboxylate (**33a**)

Prepared according to general procedure E using amine **10a** (436 mg, 1.57 mmol), carboxylic acid **31b** (190 mg, 0.63 mmol), cyanuric fluoride (16 μL, 0.19 mmol), pyridine (51 μL, 0.63 mmol), and MeCN (4 mL) with stirring at 40 °C for 18 h. Purification by MPLC on SiO_2_ with a gradient elution from 20 to 60% EtOAc/petrol gave a gray solid (160 mg, 45%); *R*_f_ 0.5 (NH_2_ SiO_2_, EtOAc); mp 159–160 °C; λ_max_ (EtOH)/nm 293, 213; IR ν_max_/cm^–1^ 1663, 1647; ^1^H NMR (500 MHz; DMSO-*d*_6_) δ_H_ 1.46 (9H, s, C(C*H*_3_)_3_), 3.34–3.38 (4H, br s, 8 × H-piperazine), 6.91 (1H, d, *J* = 9.0 Hz, H-3-pyridine), 7.46 (1H, br s, H-pyrrole), 7.55 (1H, dd, *J* = 1.3, 8.8 Hz, H-3′), 7.61 (1H, br s, H-pyrrole), 7.82 (1H, app t, *J* = 8.8 Hz, H-4′), 7.90 (1H, dd, *J* = 2.5, 9.0 Hz, H-4-pyridine′), 8.46 (1H, d, *J* = 2.5 Hz, H-6-pyridine), 10.01 (1H, s, CO-NH), 12.72 (1H, s, NH-pyrrole); ^13^C NMR (125 MHz; DMSO-*d*_6_, 110 °C) δ_C_ 28.7 (C(*C*H_3_)_3_), 43.6 (2 × CH_2_-piperazine), 45.6 (2 × CH_2_-piperazine), 79.5 (*C*(*C*H_3_)_3_), 107.4 (C-3-pyridine), 111.4 (CH-pyrrole), 119.4 (d, *J*_CF_ = 18.1 Hz), 124.7 (C-pyrrole), 126.4 (C-pyridine), 126.9 (d, *J*_CF_ = 3.6 Hz), 128.8 (C-pyrrole), 129.2 (d, *J*_CF_ = 23.2 Hz), 129.2 (d, *J*_CF_ = 5.0 Hz), 129.8 (CH-pyrrole), 131.0 (C-pyridine), 131.8, 140.0 (C-pyridine), 153.8 (d, *J*_CF_ = 248.4 Hz), 153.9 (CO-carbamate), 155.6 (C-pyridine), 158.2 (CO-NH), 182.6 (CO); ^19^F NMR (470 MHz; DMSO-*d*_6_) δ_F_ −116.7; HRMS calcd for C_26_H_27_^35^Cl_2_F_1_N_5_O_4_ [M + H]^+^ 562.1419, found 562.1415.

##### 4-(3,6-Dichloro-2-fluorobenzoyl)-*N*-(6-(piperazin-1-yl)pyridin-3-yl)-1*H*-pyrrole-2-carboxamide (**33f**)

Prepared according to general procedure J using carbamate **33a** (145 mg, 0.26 mmol), Et_3_SiH (102 μL, 0.64 mmol), TFA (1.5 mL), and CH_2_Cl_2_ (1.5 mL). The reaction was purified by MPLC on NH_2_ SiO_2_ with a gradient elution from 0 to 4% MeOH/EtOAc to give a yellow solid of 55 mg (46%); *R*_f_ 0.4 (NH_2_ SiO_2_, 5% MeOH/EtOAc); mp 195 °C (dec.); λ_max_ (EtOH)/nm 293, 213; IR ν_max_/cm^–1^ 1633; ^1^H NMR (500 MHz; DMSO-*d*_6_) δ_H_ 2.78–2.84 (4H, m, 4 × H-piperazine), 3.36–3.41 (4H, m, 4 × H-piperazine), 6.85 (1H, d, *J* = 9.1 Hz, H-3-pyridine), 7.45 (1H, s, H-pyrrole), 7.55 (1H, dd, *J* = 1.2, 8.6 Hz), 7.61 (1H, s, H-pyrrole), 7.82 (1H, app t, *J* = 8.6 Hz), 7.86 (1H, dd, *J* = 2.6, 9.1 Hz, H-4-pyridine), 8.43 (1H, d, *J* = 2.6 Hz, H-6-pyridine), 9.98 (1H, s, CO-NH); ^13^C NMR (125 MHz; DMSO-*d*_6_) δ_C_ 45.4 (2 × CH_2_-piperazine), 46.2 (2 × CH_2_-piperazine), 106.6 (C-3-pyridine), 110.7 (CH-pyrrole), 119.3 (d, *J*_CF_ = 18.1 Hz, C-3′), 124.7 (C-pyrrole), 125.9 (C-pyridine), 126.9 (d, *J*_CF_ = 3.7 Hz, C-5′), 128.9 (C-pyrrole), 129.2 (d, *J*_CF_ = 5.1 Hz), 129.2 (d, *J*_CF_ = 23.2 Hz), 129.8 (CH-pyrrole), 130.9 (C-2-pyridine), 131.8 (C-4-pyridine), 140.1 (C-6-pyridine), 153.8 (d, *J*_CF_ = 248.4 Hz), 156.3 (C-5-pyridine), 158.2 (CO-NH), 182.6 (CO); ^19^F NMR (470 MHz; DMSO-*d*_6_) δ_F_ −116.7; HRMS calcd for C_21_H_19_^35^Cl_2_F_1_N_5_O_2_ [M + H]^+^ 462.0894, found 462.0884.

##### *tert*-Butyl-4-(methyl(5-nitropyridin-2-yl)amino)piperidine-1-carboxylate (**9b**)

Prepared according to general procedure F using 2-chloro-5-nitropyridine (672 mg, 4.24 mmol) in THF (20 mL), triethylamine (650 μL, 472 mg, 4.67 mmol), and *tert*-butyl 4-(methylamino)piperidine-1-carboxylate (995 μL, 1.00 g, 4.67 mmol). The resulting solution was stirred at reflux overnight. The crude yellow solid was purified by MPLC on SiO_2_ with a gradient elution from 0 to 25% EtOAc/petrol to yield a yellow solid (1.07 g, 75%); *R*_f_ 0.31 (SiO2, 75% petrol/EtOAc); mp 158.5–160.5 °C; λ_max_ (EtOH)/nm 368.6; IR ν_max_/cm^–1^ 2963, 2926, 1691, 1595, 1571, 1509, 1477, 1410, 1334, 1295, 1241; ^1^H NMR (500 MHz, DMSO-*d*_6_) δ 1.41 (9H, s, C(C*H*_3_)_3_), 1.55–1.74 (4H, m), 2.84 (2H, brs), 2.98 (3H, s, NC*H*_3_), 4.06 (2H, brs), 4.79 (1H, brs), 6.81 (1H, d, *J* = 9.7 Hz), 8.22 (1H, dd, *J* = 9.7, 2.9 Hz), 8.97 (1H, d, *J* = 2.9 Hz); ^13^C NMR (126 MHz, DMSO-*d*_6_) δ 28.1 (C(*C*H_3_)_3_), 28, 30.3 (N*C*H_3_), 42.7, 78.8 (O*C*(CH_3_)_3_), 105.6, 132.7, 134.1, 146.0, 153.7 (*C*O_2_N), 160.1; HRMS (NSI) calcd for C_16_H_25_N_4_O_4_ [M + H]^+^ 337.1870, found 337.1871.

##### *tert*-Butyl-4-((5-aminopyridin-2-yl)(methyl)amino)piperidine-1-carboxylate (**10b**)

Prepared according to general procedure D using *tert*-butyl 4-(methyl(5-nitropyridin-2-yl)amino)piperidine-1-carboxylate (**9b**) (750 mg, 2.23 mmol), THF (22.5 mL), and MeOH (22.5 mL). The crude pale red solid (650 mg, 95%) was used in the next step without further purification; *R*_f_ 0.32 (SiO_2_, EtOAc); mp 108.0–110.0 °C; λ_max_ (EtOH)/nm 257.0; IR ν_max_/cm^–1^ 3411, 3339, 3232, 3004, 2945, 1673, 1562, 1494, 1412, 1290, 1270, 1243; ^1^H NMR (500 MHz, DMSO-*d*_6_) δ 1.40 (9H, s, C(C*H*_3_)_3_), 1.46–1.54 (4H, m, H-3′, 5′), 2.66 (3H, s, NC*H*_3_), 2.79 (2H, brs, H-2′, 6′), 4.03 (2H, brs, H-2′, 6′), 4.31–4.46 (3H, m, ArN*H*_2_ and H-4′), 6.46 (1H, d, *J* = 8.9 Hz, H-3), 6.91 (1H, dd, *J* = 8.9, 2.9 Hz, H-4), 7.58 (1H, d, *J* = 2.9 Hz, H-6); ^13^C NMR (126 MHz, DMSO-*d*_6_) δ 28.1 (C(*C*H_3_)_3_), 28.4 (C-3′, 5′), 29.9 (N*C*H_3_), 43.0 (C-2′, 6′), 52.4 (C-4′), 78.5 (O*C*(CH_3_)_3_), 106.9 (C-3), 125.1 (C-4), 133.5 (C-6), 135.6 (C-5), 151.6 (C-2), 153.8 (*C*O_2_N); MS (ES^+^) *m*/*z* 307.4 [M + H]^+^; HRMS (NSI) calcd for C_16_H_27_N_4_O_2_ [M + H]^+^ 307.2129, found 307.2128.

##### *tert*-Butyl-4-((5-(4-(3,6-dichloro-2-fluorobenzoyl)-1*H*-pyrrole-2-carboxamido)pyridin-2-yl)(methyl)amino)piperidine-1-carboxylate (**33b**)

Compound **33b** was synthesized according to general procedure I using 4-(3,6-dichloro-2-fluorobenzoyl)-1*H*-pyrrole-2-carboxylic acid (**31b**) (300 mg, 0.99 mmol), triethylamine (346 μL, 251 mg, 2.48 mmol), 2-chloro-1-methylpyridinium iodide (279 mg, 1.09 mmol), *tert*-butyl 4-((5-aminopyridin-2-yl)(methyl)amino)piperidine-1-carboxylate (**10b**) (380 mg, 1.24 mmol), and CH_2_Cl_2_ (9.9 mL). The crude yellow solid was purified by MPLC on SiO_2_ with a gradient elution from 0 to 50% EtOAc/petrol to yield a pale orange solid (285 mg, 49%); *R*_f_ 0.31 (SiO_2_, 50% petrol/EtOAc); mp 173.0–175.0 °C; λ_max_ (EtOH)/nm 290.0; IR ν_max_/cm^–1^ 3223, 2958, 2931, 2865, 1656, 1638, 1527, 1494, 1448, 1421, 1393, 1365, 1291; ^1^H NMR (500 MHz, DMSO-*d*_6_) δ 1.41 (9H, s, C(C*H*_3_)_3_), 1.52–1.63 (4H, m, C*H*_2_CH_2_NBoc), 2.79 (3H, s, NC*H*_3_), 2.81 (2H, brs, CH_2_C*H*_2_NBoc), 4.06 (2H, brs, CH_2_C*H*_2_NBoc), 4.58 (1H, tt, *J* = 10.5, 5.9 Hz, ArN (CH_3_)C*H*), 6.67 (1H, d, *J* = 9.1 Hz, H-3″), 7.41 (1H, s, H-3), 7.51 (1H, dd, *J* = 8.7, 1.4 Hz, H-5′), 7.56 (1H, s, H-5), 7.71–7.83 (2H, m, H-4′ and H-4″), 8.36 (1H, d, *J* = 2.7 Hz, H-6″), 9.91 (1H, s, CON*H*Ar), 12.67 (1H, s, NH-pyrrole); ^13^C NMR (126 MHz, DMSO-*d*_6_) δ 28.1 (C(*C*H_3_)_3_), 28.6 (*C*H_2_CH_2_NBoc), 29.6 (N*C*H_3_), 43.4 (CH_2_*C*H_2_NBoc), 51.9 (ArN (CH_3_)*C*H), 78.6 (O*C*(CH_3_)_3_), 105.6 (C-3″), 110.6 (C-3), 119.3 (d, *J* = 18.2 Hz, C-3′), 124.7 (C-2 or C-4 and C-5″), 126.9 (d, *J* = 3.3 Hz, C-5′), 128.9 (C-2 or C-4), 129.2 (d, *J* = 5.2 Hz, C-6′), 129.2 (d, *J* = 23.0 Hz, C-1′), 129.6 (C-5), 131.4 (C-4′ or C-4″), 131.8 (C-4′ or C-4″), 140.4 (C-6″), 153.8 (*C*O_2_N or C-2″), 153.8 (d, *J* = 248.4 Hz, C-2′), 155.2 (*C*O_2_N or C-2″), 158.2 (*C*ONHAr), 182.6 (ArCO); ^19^F NMR (471 MHz, DMSO-*d*_6_) δ −116.7; MS (ES^–^) *m*/*z* 588.3 [M(^35^Cl^35^Cl)-H]^−^, 590.3 [M(^35^Cl^37^Cl)-H]^−^; HRMS (NSI) calcd for C_28_H_31_Cl_2_FN_5_O_4_ [M(^35^Cl^35^Cl) + H]^+^ 590.1732, found 590.1725.

##### 4-(3,6-Dichloro-2-fluorobenzoyl)-*N*-(6-(methyl(1-methylpiperidin-4-yl)amino)pyridin-3-yl)-1*H*-pyrrole-2-carboxamide (**33g**)

Prepared according to general procedure H using *tert*-butyl 4-((5-(4-(3,6-dichloro-2-fluorobenzoyl)-1*H*-pyrrole-2-carboxamido)pyridin-2-yl)(methyl)amino)piperidine-1-carboxylate (**33b**) (100 mg, 0.17 mmol), formic acid (0.85 mL), and formaldehyde (37% wt in water) (50 μL, 0.68 mmol). The crude yellow solid was purified by MPLC on NH_2_ SiO_2_ with a gradient elution from 0 to 3% MeOH/CH_2_Cl_2_ to yield a white solid (63 mg, 74%); *R*_f_ 0.32 (NH_2_ SiO_2_, 3% MeOH/CH_2_Cl_2_); mp 217.5–219.5 °C; λ_max_ (EtOH)/nm 291.4; IR ν_max_/cm^–1^ 3313, 2981, 2971, 2782, 1646, 1584, 1532, 1500, 1448, 1394, 1296, 1281, 1264; ^1^H NMR (500 MHz, DMSO-*d*_6_) δ 1.42 −1.57 (2H, m, C*H*_2_CH_2_NMe), 1.75 (2H, dddd, *J* = 12.2, 12.2, 12.2 and 3.8 Hz, C*H*_2_CH_2_NMe), 1.99 (2H, ddd, *J* = 12.2, 12.2 and 2.5 Hz, CH_2_C*H*_2_NMe), 2.17 (3H, s, NC*H*_3_), 2.81 (3H, s, NC*H*_3_), 2.82–2.85 (2H, m, CH_2_C*H*_2_NMe), 4.34 (1H, tt, *J* = 12.2, 3.8 Hz, ArN (CH_3_)C*H*), 6.64 (1H, d, *J* = 9.1 Hz, H-5″), 7.41 (1H, s, H-3), 7.51 (1H, dd, *J* = 8.8, 1.4 Hz, H-5′), 7.56 (1H, s, H-5), 7.75 (1H, dd, *J* = 9.1, 2.7 Hz, H-4″), 7.77 (1H, dd, *J* = 8.8, 8.5 Hz, H-4′), 8.35 (1H, d, *J* = 2.7 Hz, H-2″), 9.89 (1H, s, CON*H*Ar), 12.66 (1H, s, NH-pyrrole); ^13^C NMR (126 MHz, DMSO-*d*_6_) δ 28.4 (*C*H_2_CH_2_NMe), 29.6 (N*C*H_3_), 46.0 (N*C*H_3_), 51.8 (ArN (CH_3_)*C*H), 55.1 (CH_2_*C*H_2_NMe), 105.5 (C-5″), 110.6 (C-3), 119.3 (d, *J* = 18.1 Hz, C-3′), 124.5 (C-3″), 124.7 (C-2 or C-4), 126.9 (d, *J* = 3.8 Hz, C-5′), 128.9 (C-2 or C-4), 129.2 (d, *J* = 5.5 Hz, C-6′), 129.2 (d, *J* = 23.1 Hz, C-1′), 129.7 (C-5), 131.3 (C-4′ or C-4″), 131.8 (C-4′ or C-4″), 140.4 (C-2″), 153.8 (d, *J* = 247.2 Hz, C-2′), 155.3 (C-6″), 158.2 (*C*ONHAr), 182.6 (ArCO); ^19^F NMR (471 MHz, DMSO-*d*_6_) δ −116.7 (ArF); MS (ES^–^) *m*/*z* 502.3 [M(^35^Cl^35^Cl)-H]^−^, 504.3 [M(^35^Cl^37^Cl)-H]^−^; HRMS (NSI) calcd for C_24_H_25_Cl_2_FN_5_O_2_ [M(^35^Cl^35^Cl) + H]^+^ 504.1364, found 504.1352.

##### Diethyl-2-(5-nitropyridin-2-yl)malonate (**18**)

To a suspension of sodium hydride (60% dispersion in mineral oil, 4.04 g, 101 mmol) in THF (60 mL), cooled in an ice bath, was added diethyl malonate (7.66 mL, 8.08 g, 50.5 mmol). The resulting solution was stirred at 0 °C for 10 min and allowed to warm to RT. After 1 h, the reaction mixture was cooled in an ice bath and a solution of 2-chloro-5-nitropyridine (8.0 g, 50.5 mmol) in THF (20 mL) was added dropwise. The resulting mixture was then stirred overnight at RT. Upon completion, the mixture was diluted with EtOAc (40 mL), quenched by the cautious addition of 1 M hydrochloric acid (20 mL), and extracted with EtOAc (3 × 60 mL). The pooled organic extracts were washed with water and brine (40 mL), dried over MgSO_4_, and concentrated *in vacuo*. The crude product was purified by MPLC on SiO_2_ with a gradient elution from 0 to 15% EtOAc/petrol to yield a yellow solid (9.05 g, 64%); *R*_f_ 0.32 (SiO_2_, 15% EtOac/petrol); mp 86.5–88.5 °C (lit. 97.0–99.0 °C);^42^ λ_max_ (EtOH)/nm 247.6, 272.4; IR ν_max_/cm^–1^ 3129, 3074, 2986, 2939, 1662, 1637, 1588, 1529, 1509, 1341; ^1^H NMR (500 MHz, DMSO-*d*_6_) δ 1.19 (6H, t, *J* = 7.1 Hz, OCH_2_C*H*_3_), 4.19 (4H, qd, *J* = 7.1, 1.9 Hz, OC*H*_2_CH_3_), 5.41 (1H, s, ArC*H*(CO_2_Et)_2_), 7.77 (1H, d, *J* = 8.6 Hz, H-3), 8.64 (1H, dd, *J* = 8.6, 2.7 Hz, H-4), 9.33 (1H, d, *J* = 2.7 Hz, H-6); ^13^C NMR (126 MHz, DMSO-*d*_6_) δ 13.9 (OCH_2_*C*H_3_), 59.2 (Ar*C*H(CO_2_Et)_2_), 61.8 (O*C*H_2_CH_3_), 125.0 (C-3), 132.4 (C-4), 143.7 (C-5), 144.3 (C-6), 158.9 (C-2), 166.5 (ArCH(*C*O_2_Et)_2_); MS (ES^+^) *m*/*z* 283.3 [M + H]^+^; HRMS (ESI) calcd for C_12_H_15_N_2_O_6_ [M + H]^+^ 283.0925, found 283.0917.

##### 2-Methyl-5-nitropyridine (**19**)

To diethyl-2-(5-nitropyridin-2-yl)malonate (18) (8.0 g, 28.3 mmol) was added cold 20% aqueous sulfuric acid (80 mL). The resulting solution was stirred at 100 °C for 2 h. Upon completion, the mixture was cooled in an ice bath, neutralized by the cautious addition of 2 M aqueous sodium hydroxide until pH 8–9, and extracted with CH_2_Cl_2_ (3 × 100 mL). The pooled organic extracts were washed with water (100 mL) and brine (100 mL), dried over MgSO_4_, and concentrated *in vacuo* to give a pale yellow solid (3.71 g, 95%), which was used without further purification; *R*_f_ 0.32 (10% EtOAc/petrol); mp 109.5–110.5 °C (lit. 110–111);^43^ λ_max_ (EtOH)/nm 252.8, 276.4; IR ν_max_/cm^–1^ 3039, 3019, 2950, 2854, 1600, 1572, 1512, 1469; ^1^H NMR (500 MHz, DMSO-*d*_6_) δ 2.62 (3H, s, ArC*H*_3_), 7.57 (1H, d, *J* = 8.6 Hz, H-3), 8.48 (1H, dd, *J* = 8.6, 2.8 Hz, H-4), 9.24 (1H, d, *J* = 2.8 Hz, H-6); ^13^C NMR (126 MHz, DMSO-*d*_6_) δ 24.3 (Ar*C*H_3_), 123.8 (C-3), 131.6 (C-4), 142.5 (C-5), 144.1 (C-6), 165.2 (C-2); MS (ES^+^) *m*/*z* 139.1 [M + H]^+^; HRMS (APCI) calcd for C_6_H_7_N_2_O_2_ [M + H]^+^ 139.0502, found 139.0500; ^1^H NMR, ^13^C NMR and IR data were identical to literature data.^44,45^

##### 2-Methyl-5-nitropyridine-1-oxide (**20**)

To 2-methyl-5-nitropyridine (19) (2.0 g, 14.5 mmol) in CH_2_Cl_2_ (50 mL), cooled in an ice bath, was added 3-chloroperbenzoic acid (74%, 5.06 g, 21.7 mmol) in portions. The resulting solution was stirred in an ice bath for 1 h and allowed to warm to RT. After 16 h, the reaction was quenched by the addition of saturated aqueous NaHCO_3_ (50 mL) and stirred for 30 min. The aqueous layer was extracted with CH_2_Cl_2_ (3 × 40 mL). The pooled organic extracts were washed with water (50 mL) and brine (50 mL), dried over MgSO_4_, and concentrated *in vacuo*. The crude product was purified by MPLC on SiO_2_ with a gradient elution from 0 to 100% EtOAc/petrol to yield a yellow solid (2.15 g, 96%); *R*_f_ 0.27 (100% EtOAc); mp 149.5–151.5 °C; λ_max_ (EtOH)/nm 247.8, 278.2; IR ν_max_/cm^–1^ 3126, 3102, 3036, 2920, 1564, 1519, 1491, 1350, 1285; ^1^H NMR (500 MHz, DMSO-*d*_6_) δ 2.44 (3H, s, ArC*H*_3_), 7.76 (1H, d, *J* = 8.7 Hz, H-3), 8.05 (1H, dd, *J* = 8.7, 2.2 Hz, H-4), 9.01 (1H, d, *J* = 2.2 Hz, H-6); ^13^C NMR (126 MHz, DMSO-*d*_6_) δ 17.4 (Ar*C*H_3_), 119.2 (C-4), 126.4 (C-3), 134.6 (C-6), 145.0 (C-5), 154.7 (C-2); MS (ES^+^) *m*/*z* 155.2 [M + H]^+^; HRMS (NSI) calcd for C_6_H_7_N_2_O_3_ [M + H]^+^ 155.0451, found 155.0448.

##### (5-Nitropyridin-2-yl)methanol (**21**)

To 2-methyl-5-nitropyridine 1-oxide (**20**) (1.0 g, 6.49 mmol) in CH_2_Cl_2_ (20 mL), cooled in an ice bath, was added dropwise trifluoroacetic anhydride (1.80 mL, 2.73 g, 13.0 mmol). The resulting solution was stirred in an ice bath for 1 h and allowed to warm to RT. After 16 h, the reaction was cooled in an ice bath, quenched by the addition of MeOH (15 mL), and stirred for 8 h. The volatiles were concentrated *in vacuo*. The crude residue was dissolved in EtOAc (30 mL), washed with saturated aqueous NaHCO_3_ (30 mL), and extracted with EtOAc (2 × 35 mL). The combined organic extracts were washed with water (40 mL) and brine (40 mL), dried over MgSO_4_, and concentrated *in vacuo*. The crude product was purified by MPLC on SiO_2_ with a gradient elution from 0 to 45% EtOAc/petrol to yield a yellow solid (500 mg, 50%); *R*_f_ 0.31 (45% EtOAc/petrol); mp 95.0–97.0 °C; λ_max_ (EtOH)/nm 253.2, 276.2; IR ν_max_/cm^–1^ 3161, 3040, 2917, 2852, 1596, 1575, 1515, 1451, 1434, 1345; ^1^H NMR (500 MHz, DMSO-*d*_6_) δ 4.69 (2H, s, ArC*H*_2_OH), 5.77 (1H, brs, ArCH_2_O*H*), 7.75 (1H, d, *J* = 8.7 Hz, H-3), 8.61 (1H, dd, *J* = 8.7, 2.7 Hz, H-4), 9.28 (1H, d, *J* = 2.7 Hz, H-6); ^13^C NMR (126 MHz, DMSO-*d*_6_) δ 64.0 (Ar*C*H_2_OH), 120.4 (C-3), 132.1 (C-4), 143.0 (C-5), 143.9 (C-6), 168.9 (C-2); MS (ES^+^) *m*/*z* 155.2 [M + H]^+^; HRMS (APCI) calcd for C_6_H_7_N_2_O_3_ [M + H]^+^ 155.0451, found 155.0450.

##### 5-Nitropicolinaldehyde (**22**)

To (5-nitropyridin-2-yl)methanol (**21**) (1.2 g, 7.79 mmol) in CH_2_Cl_2_ (30 mL) was added manganese oxide (6.77 g, 77.9 mmol). The resulting solution was stirred at RT for 16 h. Upon completion, the heterogeneous mixture was filtered through celite and washed with CH_2_Cl_2_ (15 mL). The filtrate was concentrated *in vacuo* to give a yellow solid. The crude solid was purified by MPLC on SiO_2_ with a gradient elution from 0 to 20% EtOAc/petrol to yield an orange solid (720 mg, 61%); *R*_f_ 0.28 (20% EtOAc/petrol); mp 65.0–67.0 °C (lit. 55 °C);^44^ λ_max_ (EtOH)/nm 248.2, 273.6; IR ν_max_/cm^–1^ 3102, 2981, 2889, 2845, 1712, 1598, 1528, 1349; ^1^H NMR (500 MHz, DMSO-*d*_6_) δ 8.16 (1H, dd, *J* = 8.5, 0.7 Hz, H-3), 8.80 (1H, dd, *J* = 8.5, 2.5 Hz, H-4), 9.56 (1H, d, *J* = 2.5 Hz, H-6), 10.08 (1H, d, *J* = 0.7 Hz, ArC*H*O); ^13^C NMR (126 MHz, DMSO-*d*_6_) δ 122.4 (C-3), 133.4 (C-4), 145.3 (C-6), 146.1 (C-5), 155.2 (C-2), 192.0 (Ar*C*HO); MS (ES^+^) *m*/*z* 153.2 [M + H]^+^; HRMS (APCI) calcd for C6H5N2O3 [M + H]^+^ 153.0295, found 153.0292; ^1^H and ^13^C NMR data were identical to literature data.^44^

##### *tert*-Butyl-4-((5-nitropyridin-2-yl)methyl)piperazine-1-carboxylate (**23**)

To 5-nitropicolinaldehyde (**22**) (300 mg, 1.97 mmol) in TFE (10 mL) was added *tert*-butyl piperazine-1-carboxylate (367 mg, 1.97 mmol). The resulting solution was stirred at 38 °C for 1 h. Once cooled at 0 °C, sodium borohydride was carefully added. The resulting mixture was allowed to warm to RT and then stirred for 30 min. Upon completion, the solvent was removed *in vacuo*. The crude residue was dissolved in EtOAc (30 mL), neutralized by washing with saturated aqueous NH_4_Cl (20 mL), washed with water (20 mL) and brine (20 mL), dried over MgSO_4_, and concentrated *in vacuo*. The crude product was purified by MPLC on SiO_2_ with a gradient elution from 0 to 50% EtOAc/petrol to yield a white solid (325 mg, 51%); *R*_f_ 0.34 (petrol/EtOAc, 1:1); mp 107.5–109.5 °C; λ_max_ (EtOH)/nm 246.4, 305.4; IR ν_max_/cm^–1^ 2981, 2941, 2881, 2820, 1686, 1601, 1580, 1523, 1420, 1356, 1345; ^1^H NMR (500 MHz, DMSO-*d*_6_) δ 1.39 (9H, s, C(C*H*_3_)_3_), 2.40 (4H, t, *J* = 5.0 Hz, CH_2 piperazine_), 3.34 (4H, t, *J* = 5.0 Hz, CH_2 piperazine_), 3.76 (2H, brs, ArC*H*_2_N), 7.75 (1H, d, *J* = 8.6 Hz, H-3), 8.57 (1H, dd, *J* = 8.6, 2.7 Hz, H-4), 9.29 (1H, d, *J* = 2.7 Hz, H-6); ^13^C NMR (126 MHz, DMSO-*d*_6_) δ 28.1 (C(*C*H_3_)_3_), 43.0 (CH_2 piperazine_), 52.5 (CH_2 piperazine_), 63.0 (Ar*C*H_2_N), 78.8 (O*C*(CH_3_)_3_), 123.1 (C-3), 132.0 (C-4), 143.2 (C-5), 144.1 (C-6), 153.8 (*C*O_2_N), 165.2 (C-2); MS (ES^–^) *m*/*z* 321.2 [M – H]^−^; HRMS (NSI) calcd for C_15_H_23_N_4_O_4_ [M + H]^+^ 323.1714, found 323.1712.

##### *tert*-Butyl-4-((5-aminopyridin-2-yl)methyl)piperazine-1-carboxylate (**24**)

Compound **24** was synthesized according to general procedure D using *tert*-butyl 4-((5-nitropyridin-2-yl)methyl)piperazine-1-carboxylate (**23**) (300 mg, 0.93 mmol), THF (9.3 mL**)**, and MeOH (9.3 mL). The crude colorless oil (258 mg, 95%) was used in the next step without further purification; *R*_f_ 0.32 (100%, EtOAc); λ_max_ (EtOH)/nm 246.4, 305.4; IR ν_max_/cm^–1^ 3410, 3327, 3204, 2980, 2925, 2891, 2808, 2769, 1671, 1598, 1574, 1495, 1455, 1423; ^1^H NMR (500 MHz, DMSO-*d*_6_) δ 1.38 (9H, s, C(C*H*_3_)_3_), 2.29 (4H, t, *J* = 5.1 Hz, CH_2 piperazine_), 3.28 (4H, t, *J* = 5.1 Hz, CH_2 piperazine_), 3.39 (2H, s, ArC*H*_2_N), 5.18 (2H, brs, ArN*H*_2_), 6.88 (1H, dd, *J* = 8.3, 2.8 Hz, H-4), 7.03 (1H, d, *J* = 8.3 Hz, H-3), 7.83 (1H, d, *J* = 2.8 Hz, H-6); ^13^C NMR (126 MHz, DMSO-*d*_6_) δ 28.0 (C(*C*H_3_)_3_), 43.5 (CH_2 piperazine_), 52.4 (CH_2 piperazine_), 63.3 (Ar*C*H_2_N), 78.7 (O*C*(CH_3_)_3_), 120.4 (C-4), 123.1 (C-3), 135.2 (C-6), 143.5 (C-2 or C-5), 144.6 (C-2 or C-5), 153.8 (*C*O_2_N); MS (ES^+^) *m*/*z* 293.5 [M + H]^+^; HRMS (NSI) calcd for C_15_H_25_N_4_O_2_ [M + H]^+^ 293.1972, found 293.1971.

##### *tert*-Butyl-4-((5-(4-(3,6-dichloro-2-fluorobenzoyl)-1*H*-pyrrole-2-carboxamido)pyridin-2-yl)methyl)piperazine-1-carboxylate (**33c**)

Prepared according to general procedure I using 4-(3,6-dichloro-2-fluorobenzoyl)-1*H*-pyrrole-2-carboxylic acid (**31b**) (211 mg, 0.70 mmol), triethylamine (243 μL, 177 mg, 1.75 mmol), 2-chloro-1-methylpyridinium iodide (196 mg, 0.77 mmol), *tert*-butyl 4-((5-aminopyridin-2-yl)methyl)piperazine-1-carboxylate (**24**) (255 mg, 0.87 mmol), and CH_2_Cl_2_ (7 mL). The crude yellow solid was purified by MPLC on SiO_2_ with a gradient elution from 0 to 80% EtOAc/petrol to yield a pale orange solid (113 mg, 28%); *R*_f_ 0.28 (petrol/EtOAc, 8:2); mp 147.0–149.0 °C; λ_max_ (EtOH)/nm 295.8; IR ν_max_/cm^–1^ 3238, 2964, 2932, 2871, 2819, 1652, 1531, 1448, 1423, 1393; ^1^H NMR (500 MHz, DMSO-*d*_6_) δ 1.39 (9H, s, C(C*H*_3_)_3_), 2.32–2.42 (4H, m, CH_2 piperazine_), 3.33 (4H, brs, CH_2 piperazine_), 3.56 (2H, s, ArC*H*_2_N), 7.42 (1H, d, *J* = 8.5 Hz, H-3″), 7.48–7.54 (2H, m, H-3 and H-5′), 7.62 (1H, s, H-5), 7.78 (1H, dd, *J* = 8.7, 8.2 Hz, H-4′), 8.11 (1H, dd, *J* = 8.5, 2.6 Hz, H-4″), 8.80 (1H, d, *J* = 2.6 Hz, H-6″), 10.23 (1H, s, CON*H*Ar), 12.77 (1H, s, NH-pyrrole); ^13^C NMR (126 MHz, DMSO-*d*_6_) δ 28.1 (C(*C*H_3_)_3_), 43.2 (CH_2 piperazine_), 52.5 (CH_2 piperazine_), 63.2 (Ar*C*H_2_N), 78.7 (O*C*(CH_3_)_3_), 111.4 (C-5), 119.3 (d, *J* = 18.0 Hz, C-3′), 122.7 (C-3″), 124.8 (C-2 or C-4), 126.9 (d, *J* = 3.7 Hz, C-5′), 127.6 (C-4″), 128.5 (C-2 or C-4), 129.1 (d, *J* = 22.9 Hz, C-1′), 129.2 (d, *J* = 5.3 Hz, C-6′), 130.3 (C-5), 131.9 (C-4′), 134.1 (C-5″), 140.7 (C-6″), 152.8 (*C*O_2_N or C-2″), 153.8 (*C*O_2_N or C-2″), 153.9 (d, *J* = 248.7 Hz, C-2′), 158.6 (*C*ONHAr), 182.6 (ArCO); ^19^F NMR (471 MHz, DMSO-*d*_6_) δ −116.7 (ArF); MS (ES^+^) *m*/*z* 576.5 [M(^35^Cl^35^Cl) + H]^+^, 578.5 [M(^35^Cl^37^Cl) + H]^+^; HRMS (NSI) calcd for C_27_H_29_Cl_2_FN_5_O_4_ [M(^35^Cl^35^Cl) + H]^+^ 576.1575, found 576.1568.

##### 4-(3,6-Dichloro-2-fluorobenzoyl)-*N*-(6-((4-methylpiperazin-1-yl)methyl)pyridin-3-yl)-1*H*-pyrrole-2-carboxamide (**33i**)

Prepared according to general procedure H using *tert*-butyl 4-((5-(4-(3,6-dichloro-2-fluorobenzoyl)-1*H*-pyrrole-2-carboxamido)pyridin-2-yl)methyl)piperazine-1-carboxylate (**33c**) (45 mg, 0.08 mmol), formic acid (0.4 mL), and formaldehyde (37% wt in water) (23 μL, 0.31 mmol). The crude yellow solid was purified by MPLC on NH_2_ SiO_2_ with a gradient elution from 0 to 5% MeOH/CH_2_Cl_2_ to yield a white solid (25 mg, 66%); *R*_f_ 0.28 (NH_2_ SiO_2_, 0–5% MeOH/CH_2_Cl_2_); mp 161.5–163.5 °C; λ_max_ (EtOH)/nm 296.0; IR ν_max_/cm^–1^ 3215, 2933, 2802, 1639, 1590, 1527, 1491, 1446, 1391; ^1^H NMR (500 MHz, DMSO-*d*_6_) δ 2.16 (3H, s, NC*H*_3_), 2.22–2.49 (8H, m, NC*H*_2 piperazine_), 3.53 (2H, s, ArC*H*_2_N), 7.39 (1H, d, *J* = 8.5 Hz, H-5″), 7.47–7.55 (2H, m, H-3 and H-5′), 7.61 (1H, s, H-5), 7.78 (1H, dd, *J* = 8.8, 8.4 Hz, H-4′), 8.10 (1H, dd, *J* = 8.5, 2.5 Hz, H-4″), 8.79 (1H, d, *J* = 2.5 Hz, H-2″), 10.22 (1H, s, CON*H*Ar), 12.76 (1H, s, NH-pyrrole); ^13^C NMR (126 MHz, DMSO-*d*_6_) δ 45.7 (N*C*H_3_), 52.6 (N*C*H_2 piperazine_), 54.7 (N*C*H_2 piperazine_), 63.3 (Ar*C*H_2_N), 111.4 (C-3), 119.4 (d, *J* = 18.0 Hz), 122.6 (C-5″), 124.8 (C-2 or C-4), 126.9 (d, *J* = 3.7 Hz, C-5′), 127.6 (C-4″), 128.5 (C-2 or C-4), 129.1 (d, *J* = 23.2 Hz, C-1′), 129.2 (d, *J* = 4.9 Hz, C-6′), 130.2 (C-5), 131.9 (C-4′), 134.0 (C-3″), 140.7 (C-2″), 153.2 (C-6″), 153.9 (d, *J* = 248.5 Hz, C-2′), 158.6 (*C*ONHAr), 182.6 (Ar*C*O); ^19^F NMR (471 MHz, DMSO-*d*_6_) δ −116.7 (ArF); MS (ES^+^) *m*/*z* 490.4 [M(^35^Cl^35^Cl) + H]^+^, 492.5 [M(^35^Cl^37^Cl) + H]^+^; HRMS (NSI) calcd for C_23_H_23_Cl_2_FN_5_O_2_ [M(^35^Cl^35^Cl) + H]^+^ 490.1207, found 490.1195.

##### 4-(3,6-Dichloro-2-fluorobenzoyl)-*N*-(6-(piperazin-1-ylmethyl)pyridin-3-yl)-1*H*-pyrrole-2-carboxamide (**33h**)

Prepared according to general procedure J using *tert*-butyl 4-((5-(4-(3,6-dichloro-2-fluorobenzoyl)-1*H*-pyrrole-2-carboxamido)pyridin-2-yl)methyl)piperazine-1-carboxylate (**33c**) (50 mg, 0.09 mmol), triethylsilane (35 μL, 25 mg, 0.22 mmol), TFA (0.45 mL), and CH_2_Cl_2_ (0.45 mL). The crude yellow solid was purified by MPLC on NH_2_ SiO_2_ with a gradient elution from 0 to 10% MeOH/CH_2_Cl_2_ to yield a white solid (30 mg, 73%); *R*_f_ 0.25 (NH_2_ SiO_2_, 0–10% MeOH/CH_2_Cl_2_); mp 179.5–181.5 °C; λ_max_ (EtOH)/nm 296.0; IR ν_max_/cm^–1^ 3105, 2921, 2812, 1637, 1589, 1525, 1490, 1445, 1389; ^1^H NMR (500 MHz, DMSO-*d*_6_) δ 2.33 (4H, brs, NC*H*_2 piperazine_), 2.70 (4H, t, *J* = 4.8 Hz, NC*H*_2 piperazine_), 3.51 (2H, s, ArC*H*_2_N), 7.40 (1H, d, *J* = 8.5 Hz, H-5″), 7.49 (1H, s, H-3), 7.52 (1H, dd, *J* = 8.8, 0.6 Hz, H-5′), 7.60 (1H, s, H-5), 7.78 (1H, dd, *J* = 8.8, 8.3 Hz, H-4′), 8.09 (1H, dd, *J* = 8.5, 2.6 Hz, H-4″), 8.79 (1H, d, *J* = 2.6 Hz, H-2″), 10.20 (1H, s, CON*H*Ar); ^13^C NMR (126 MHz, DMSO-*d*_6_) δ 45.5 (N*C*H_2 piperazine_), 54.0 (N*C*H_2 piperazine_), 64.1 (Ar*C*H_2_N), 111.4 (C-3), 119.3 (d, *J* = 17.9 Hz, C-3′), 122.6 (C-5″), 124.8 (C-2 or C-4), 126.9 (d, *J* = 3.7 Hz, C-5′), 127.5 (C-4″), 128.7 (C-2 or C-4), 129.2 (d, *J* = 23.0 Hz, C-1′), 129.2 (d, *J* = 5.2 Hz, C-6′), 130.4 (C-5), 131.8 (C-4′), 134.0 (C-3″), 140.6 (C-2″), 153.2 (C-6″), 154.8 (d, *J* = 248.8 Hz, C-2′), 158.7 (*C*ONHAr), 182.5 (ArCO); ^19^F NMR (471 MHz, DMSO-*d*_6_) δ −116.6 (ArF); MS (ES^+^) *m*/*z* 476.5 [M(^35^Cl^35^Cl) + H]^+^, 478.5 [M(^35^Cl^37^Cl) + H]^+^; HRMS (NSI) calcd for C_22_H_21_Cl_2_FN_5_O_2_ [M(^35^Cl^35^Cl) + H]^+^ 476.1051, found 476.1040.

##### *tert*-Butyl-4-((5-nitropyridin-2-yl)methyl)piperidine-1-carboxylate (**9d**)

To a degassed sample of *tert*-butyl 4-methylidenepiperidine-1-carboxylate (750 mg, 3.80 mmol) was added 9-BBN (0.5 M in THF) (7.60 mL, 3.80 mmol). The resulting solution was sparged with nitrogen for 15 min and then reflux for 3 h. After cooling to RT, *N*,*N*-dimethylformamide (7 mL) and water (0.7 mL) were added and the resulting solution was sparged with nitrogen for 15 min. To the degassed mixture were added 2-chloro-5-nitropyridine (1.20 g, 7.60 mmol), potassium carbonate (788 mg, 5.70 mmol), and [1,1′-bis(diphenylphosphino)ferrocene]dichloropalladium(II) complex with CH_2_Cl_2_ (233 mg, 0.28 mmol). The resulting mixture was heated at 60 °C overnight. Upon completion, the heterogeneous mixture was filtered through celite and the solvent was removed *in vacuo*. The crude residue was dissolved in a mixture of EtOAc (30 mL) and water (30 mL) and extracted with EtOAc (3 × 30 mL). The combined organic extracts were washed with water (40 mL) and brine (40 mL), dried over MgSO_4_, and concentrated *in vacuo*. The crude product was purified by MPLC on SiO_2_ with a gradient elution from 0 to 25% EtOAc/petrol to yield a yellow solid (485 mg, 40%); *R*_f_ 0.31 (25% EtOAc/petrol/EtOAc); mp 80.5–82.5 °C; λ_max_ (EtOH)/nm 254.8, 278.4; IR ν_max_/cm^–1^ 3044, 2972, 2922, 2851, 1683, 1597, 1576, 1515, 1468, 1423, 1354, 1287, 1238; ^1^H NMR (500 MHz, DMSO-*d*_6_) δ 1.04–1.15 (2H, m, H-3′, 5′), 1.38 (9H, s, C(C*H*_3_)_3_), 1.48–1.56 (2H, m, H-3′, 5′), 1.91–2.03 (1H, m, H-4′), 2.66 (2H, brs, H-2′, 6′), 2.82 (2H, d, *J* = 7.1 Hz, ArC*H*_2_), 3.89 (2H, d, *J* = 13.2 Hz, H-2′, 6′), 7.56 (1H, d, *J* = 8.6 Hz, H-3), 8.50 (1H, dd, *J* = 8.6, 2.7 Hz, H-4), 9.29 (1H, d, *J* = 2.7 Hz, H-6); ^13^C NMR (126 MHz, DMSO-*d*_6_) δ 28.1 (C(C*H*_3_)_3_), 31.3 (C-3′, 5′), 35.8 (C-4′), 43.1 (C-2′, 6′), 44.0 (Ar*C*H_2_), 78.4 (O*C*(CH_3_)_3_), 124.2 (C-3), 131.6 (C-4), 142.6 (C-5), 144.2 (C-6), 153.8 (*C*O_2_N), 167.0 (C-2); MS (ES^+^) *m*/*z* 320.3 [M + H]^+^; HRMS (NSI) calcd for C_16_H_24_N_3_O_4_ [M + H]^+^ 322.1761, found 322.1763.

##### *tert*-Butyl-4-((5-aminopyridin-2-yl)methyl)piperidine-1-carboxylate (**10d**)

Prepared according to general procedure D using *tert*-butyl 4-((5-nitropyridin-2-yl)methyl)piperidine-1-carboxylate (**9d**) (300 mg, 0.93 mmol), THF (9.3 mL), and MeOH (9.3 mL). The crude orange solid (261 mg, 96%) was used in the next step without further purification; *R*_f_ 0.26 (NH_2_ SiO_2_, 50% EtOAc/petrol); mp 107.5–109.5 °C; λ_max_ (EtOH)/nm 243.6, 307.4; IR ν_max_/cm^–1^ 3398, 3323, 3219, 2981, 2917, 2852, 1668, 1573, 1493, 1425, 1366; ^1^H NMR (500 MHz, DMSO-*d*_6_) δ 1.00 (2H, dddd, *J* = 12.3, 12.3, 12.3 and 4.3 Hz, H-3′, 5′), 1.38 (9H, s, C(C*H*_3_)_3_), 1.47–1.54 (2H, m, H-3′, 5′), 1.76 (1H, ttt, *J* = 12.3, 7.2 and 4.3 Hz, H-4′), 2.44 (2H, d, *J* = 7.2 Hz, ArC*H*_2_CH), 2.64 (2H, brs, H-2′, 6′), 3.88 (2H, d, *J* = 13.1 Hz, H-2′, 6′), 5.03 (2H, brs, ArN*H*_2_), 6.81–6.84 (2H, m, H-3 and H-4), 7.84 (1H, d, *J* = 1.7 Hz, H-6); ^13^C NMR (126 MHz, DMSO-*d*_6_) δ 28.1 (C(C*H*_3_)_3_), 31.5 (C-3′, 5′), 36.2 (C-4′), 42.9 (C-2′, 6′), 43.3 (Ar*C*H_2_), 78.3 (O*C*(CH_3_)_3_), 120.4 (C-3 or C-4), 123.0 (C-3 or C-4), 135.7 (C-6), 142.5 (C-5), 146.8 (C-2), 153.8 (*C*O_2_N); MS (ES^+^) *m*/*z* 292.4 [M + H]^+^; HRMS (NSI) calcd for C_16_H_26_N_3_O_2_ [M + H]^+^ 292.2020, found 292.2019.

##### *tert*-Butyl-4-((5-(4-(3,6-dichloro-2-fluorobenzoyl)-1*H*-pyrrole-2-carboxamido)pyridin-2-yl)methyl)piperidine-1-carboxylate (**33d**)

Prepared according to general procedure I using 4-(3,6-dichloro-2-fluorobenzoyl)-1*H*-pyrrole-2-carboxylic acid (**31b**) (250 mg, 0.83 mmol), triethylamine (288 μL, 209 mg, 2.07 mmol), 2-chloro-1-methylpyridinium iodide (233 mg, 0.91 mmol), *tert*-butyl 4-((5-aminopyridin-2-yl)methyl)piperidine-1-carboxylate (**10d**) (301 mg, 1.03 mmol), and CH_2_Cl_2_ (8.30 mL). The crude yellow solid was purified by MPLC on SiO_2_ with a gradient elution from 0 to 100% EtOAc/petrol to yield an orange solid (201 mg, 42%); *R*_f_ 0.29 (50% EtOAc/petrol); mp 132.0–134.0 °C; λ_max_ (EtOH)/nm 295.6; IR ν_max_/cm^–1^ 3238, 2926, 2853, 1651, 1592, 1531, 1448, 1424, 1394, 1366; ^1^H NMR (500 MHz, DMSO-*d*_6_) δ 1.01–1.10 (2H, m, C*H*_2_CH_2_NBoc), 1.38 (9H, s, C(C*H*_3_)_3_), 1.53 (2H, dd, *J* = 12.6, 3.6 Hz, C*H*_2_CH_2_NBoc), 1.83–1.90 (1H, m, ArCH_2_C*H*), 2.62 (2H, d, *J* = 7.1 Hz, ArC*H*_2_CH), 2.66 (2H, brs, CH_2_C*H*_2_NBoc), 3.90 (2H, d, *J* = 13.1 Hz, CH_2_C*H*_2_NBoc), 7.21 (1H, d, *J* = 8.4 Hz, H-3″), 7.50 (1H, s, H-3), 7.52 (1H, dd, *J* = 8.8, 1.2 Hz, H-5′), 7.61 (1H, s, H-5), 7.78 (1H, dd, *J* = 8.8, 8.4 Hz, H-4′), 8.02 (1H, dd, *J* = 8.4, 2.5 Hz, H-4″), 8.78 (1H, d, *J* = 2.5 Hz, H-6″), 10.18 (1H, s, CON*H*Ar), 12.75 (1H, s, NH-pyrrole);^13^C NMR (126 MHz, DMSO-*d*_6_) δ 28.1 (*C*(CH_3_)_3_), 31.5 (*C*H_2_CH_2_NBoc), 36.0 (ArCH_2_*C*H), 43.6 (Ar*C*H_2_CH), 43.9 (CH_2_*C*H_2_NBoc), 78.4 (O*C*(CH_3_)_3_), 111.3 (C-3), 119.3 (d, *J* = 17.8 Hz, C-3′), 123.1 (C-3″), 124.8 (C-2 or C-4), 126.9 (d, *J* = 3.7 Hz, C-5′), 127.5 (C-4″), 128.5 (C-2 or C-4), 129.1 (d, *J* = 22.8 Hz, C-1′), 129.2 (d, *J* = 5.4 Hz, C-6′), 130.2 (C-5), 131.9 (C-4′), 133.2 (C-5″), 141.1 (C-6″), 153.8 (*C*O_2_N or C-2″), 153.9 (d, *J* = 248.6 Hz, C-2′), 154.8 (*C*O_2_N or C-2″), 158.5 (*C*ONHAr), 182.6 (ArCO); ^19^F NMR (471 MHz, DMSO-*d*_6_) δ −116.7 (ArF); MS (ES^+^) *m*/*z* 573.4 [M(^35^Cl^35^Cl) + H]^+^, 575.4 [M(^35^Cl^37^Cl) + H]^+^; HRMS (NSI) calcd for C_28_H_30_Cl_2_FN_4_O_4_ [M(^35^Cl^35^Cl) + H]^+^ 575.1623, found 575.1616.

##### 4-(3,6-Dichloro-2-fluorobenzoyl)-*N*-(6-((1-methylpiperidin-4-yl)methyl)pyridin-3-yl)-1*H*-pyrrole-2-carboxamide (**33j**)

Prepared according to general procedure H using *tert*-butyl 4-((5-(4-(3,6-dichloro-2-fluorobenzoyl)-1*H*-pyrrole-2-carboxamido)pyridin-2-yl)methyl)piperidine-1-carboxylate (**33d**) (100 mg, 0.17 mmol), formic acid (0.85 mL), and formaldehyde (37% wt in water) (52 μL, 0.69 mmol). The crude yellow solid was purified by MPLC on NH_2_ SiO_2_ with a gradient elution from 0 to 4% MeOH/CH_2_Cl_2_ to yield an off-white solid (64 mg, 75%); *R*_f_ 0.26 (NH_2_ SiO_2_, 4% MeOH/CH_2_Cl_2_); mp 148.0–150.0 °C; λ_max_ (EtOH)/nm 261.4, 296.0; IR ν_max_/cm^–1^ 3286, 2926, 2846, 2788, 1646, 1592, 1525, 1493, 1447, 1392, 1282, 1237, 1224; ^1^H NMR (500 MHz, DMSO-*d*_6_) δ 1.11–1.31 (2H, m, C*H*_2_CH_2_NMe), 1.50 (2H, d, *J* = 12.0 Hz, C*H*_2_CH_2_NMe), 1.57–1.70 (1H, m, ArCH_2_C*H*), 1.77 (2H, dd, *J* = 12.0, 12.0 Hz, CH_2_C*H*_2_NMe), 2.11 (3H, s, NC*H*_3_), 2.59 (2H, d, *J* = 7.0 Hz, ArC*H*_2_CH), 2.70 (2H, d, *J* = 10.9 Hz, CH_2_C*H*_2_NMe), 7.20 (1H, d, *J* = 8.4 Hz, H-5″), 7.49 (1H, s, H-3), 7.52 (1H, d, *J* = 8.8 Hz, H-5′), 7.61 (1H, s, H-5), 7.78 (1H, dd, *J* = 8.8, 8.3 Hz, H-4′), 8.02 (1H, d, *J* = 8.4 Hz, H-4″), 8.78 (1H, s, H-2″), 10.17 (1H, s, CON*H*Ar), 12.72 (1H, s, NH-pyrrole); ^13^C NMR (126 MHz, DMSO-*d*_6_) δ 31.7 (*C*H_2_CH_2_NMe), 35.6 (ArCH_2_*C*H), 43.8 (Ar*C*H_2_CH), 46.2 (NC*H*_3_), 55.3 (CH_2_*C*H_2_NMe), 111.3 (C-3), 119.3 (d, *J* = 18.1 Hz, C-3′), 123.0 (C-5″), 124.8 (C-2 or C-4), 126.9 (d, *J* = 3.7 Hz, C-5′), 127.4 (C-4″), 128.6 (C-2 or C-4), 129.2 (d, *J* = 23.0 Hz, C-1′), 129.2 (d, *J* = 5.0 Hz, C-3′), 130.2 (C-5), 131.8 (C-4′), 133.1 (C-3″), 141.0 (C-2″), 153.9 (d, *J* = 248.9 Hz, C-2′), 155.1 (*C*ONHAr or C-6″), 158.6 (*C*ONHAr or C-6″), 182.6 (ArCO); ^19^F NMR (471 MHz, DMSO-*d*_6_) δ −116.6 (ArF); MS (ES^–^) *m*/*z* 487.3 [M(^35^Cl^35^Cl)-H]^−^, 489.3 [M(^35^Cl^37^Cl)-H]^−^; HRMS (NSI) calcd for C_24_H_24_Cl_2_FN_4_O_2_ [M(^35^Cl^35^Cl) + H]^+^ 489.1255, found 489.1243.

##### *tert*-Butyl-4-((5-nitropyridin-2-yl)oxy)piperidine-1-carboxylate^46^ (**9c**)

To a suspension of sodium hydride (60% dispersion in mineral oil, 246 mg, 6.15 mmol) in THF (20 mL), cooled in an ice bath, was added 1-Boc-4-hydroxypiperidine (1.24 g, 6.15 mmol). The resulting solution was stirred at 0 °C for 10 min and allowed to warm to RT. After 1 h, the reaction mixture was cooled in an ice bath and 2-chloro-5-nitropyridine (650 mg, 4.10 mmol) was added in small portions. The resulting mixture was then stirred overnight at RT. Upon completion, the mixture was diluted with EtOAc (20 mL), quenched by the cautious addition of saturated aqueous NaHCO_3_ (20 mL), and extracted with EtOAc (3 × 20 mL). The combined organic extracts were washed with water (40 mL) and brine (40 mL), dried over MgSO_4_, and concentrated *in vacuo*. The crude product was purified by MPLC on SiO_2_ with a gradient elution from 0 to 100% EtOAc/petrol to yield an off-white solid (1.03 g, 78%); *R*_f_ 0.30 (10% petrol/EtOAc); mp 109.5–111.5 °C; λ_max_ (EtOH)/nm 295.0; IR ν_max_/cm^–1^ 2961, 2924, 2873, 1675, 1605, 1579, 1513, 1473, 1425, 1349, 1318, 1273; ^1^H NMR (500 MHz, DMSO-*d*_6_) δ 1.41 (9H, s, C(C*H*_3_)_3_), 1.60 (2H, dddd, *J* = 12.9, 8.8, 8.8 and 4.0 Hz, H-3′, 5′_axial_), 2.03–1.90 (2H, m, H-3′, 5′_equ_), 3.20 (2H, brs, H-2′, 6′_axial_), 3.69 (2H, ddd, *J* = 12.9, 4.6 and 4.6 Hz, H-2′, 6′_equ_), 5.32 (1H, tt, *J* = 8.8, 4.0 Hz, H-4′), 7.02 (1H, d, *J* = 9.1 Hz, H-3), 8.47 (1H, dd, *J* = 9.1, 2.9 Hz, H-4), 9.07 (1H, d, *J* = 2.9 Hz, H-6); ^13^C NMR (126 MHz, DMSO-*d*_6_) δ 28.0 (C(*C*H_3_)_3_), 30.2 (C-3′, 5′), 40.6 (C-2′, 6′) 72.3 (C-4′), 78.8 (O*C*(CH_3_)_3_), 111.8 (C-3), 134.9 (C-4), 139.3 (C-5), 144.6 (C-6), 153.9 (*C*O_2_N), 165.9 (C-2); HRMS (NSI) calcd for C_15_H_22_N_3_O_5_ [M + H]^+^ 324.1554, found 324.1553; ^1^H NMR data were identical to literature data.

##### *tert*-Butyl-4-((5-aminopyridin-2-yl)oxy)piperidine-1-carboxylate (**10c**)

Prepared according to general procedure D using *tert*-butyl 4-((5-nitropyridin-2-yl)oxy)piperidine-1-carboxylate (**9c**) (800 mg, 2.47 mmol), THF (24.7 mL), and MeOH (24.7 mL). The crude pale yellow solid (700 mg, 96%) was used in the next step without further purification; *R*_f_ 0.29 (100% EtOAc); mp 160.0–162.0 °C; λ_max_ (EtOH)/nm 236.6, 313.8; IR ν_max_/cm^–1^ 3384, 3335, 2980, 2961, 2925, 2865, 1681, 1485, 1418, 1369, 1270, 1249, 1236; ^1^H NMR (500 MHz, DMSO-*d*_6_) δ 1.40 (9H, s, C(C*H*_3_)_3_), 1.46 (2H, dddd, *J* = 13.2, 9.2, 9.1 and 4.1 Hz, H-3′, 5′_axial_), 1.87 (2H, ddd, *J* = 13.2, 5.8 and 3.3 Hz, H-3′, 5′_equ_), 3.12 (2H, brs, H-2′, 6′_axial_), 3.66 (2H, ddd, *J* = 13.2, 4.9 and 4.9 Hz, H-2′, 6′_equ_), 4.74 (2H, brs, ArN*H*_2_), 4.93 (1H, tt, *J* = 8.3, 3.8 Hz, H-4′), 6.51 (1H, d, *J* = 8.6 Hz, H-3), 6.98 (1H, dd, *J* = 8.6, 2.9 Hz, H-4), 7.48 (1H, d, *J* = 2.9 Hz, H-6); ^13^C NMR (126 MHz, DMSO-*d*_6_) δ 28.1 (C(*C*H_3_)_3_), 30.7 (C-3′, 5′), 40.6 (C-2′, 6′), 69.3 (C-4′), 78.6 (O*C*(CH_3_)_3_), 110.9 (C-3), 126.3 (C-4), 131.1 (C-6), 139.4 (C-5), 153.9 (C-2 or *C*O_2_N), 154.3 (C-2 or *C*O_2_N); MS (ES^+^) *m*/*z* 294.4 [M + H]^+^; HRMS (ESI) calcd for C_15_H_24_N_3_O_3_ [M + H]^+^ 294.1812, found 294.1811.

##### *tert*-Butyl-4-((5-(4-(3,6-dichloro-2-fluorobenzoyl)-1*H*-pyrrole-2-carboxamido)pyridin-2-yl)oxy)piperidine-1-carboxylate (**33e**)

Prepared according to general procedure J using 4-(3,6-dichloro-2-fluorobenzoyl)-1*H*-pyrrole-2-carboxylic acid (**31b**) (300 mg, 0.99 mmol), triethylamine (346 μL, 251 mg, 2.48 mmol), 2-chloro-1-methylpyridinium iodide (279 mg, 1.09 mmol), *tert*-butyl 4-((5-aminopyridin-2-yl)oxy)piperidine-1-carboxylate (**10e**) (364 mg, 1.24 mmol), and CH_2_Cl_2_ (9.9 mL). The crude yellow solid was purified by MPLC on SiO_2_ with a gradient elution from 0 to 40% EtOAc/petrol to yield a pale orange solid (275 mg, 48%); *R*_f_ 0.30 (60% petrol/EtOAc); mp 226.5–228.5 °C; λ_max_ (EtOH)/nm 261.6; IR ν_max_/cm^–1^ 3166, 2980, 2936, 2857, 1658, 1647, 1593, 1556, 1538, 1484, 1433, 1365, 1263;^1^H NMR (500 MHz, DMSO-*d*_6_) δ 1.41 (9H, s, C(C*H*_3_)_3_), 1.53 (2H, dddd, *J* = 13.0, 9.1, 9.0 and 4.0 Hz, C*H*_2_CH_2_NBoc), 1.94 (2H, ddd, *J* = 13.0, 5.9 and 3.4 Hz, C*H*_2_CH_2_NBoc), 3.16 (2H, brs, CH_2_C*H*_2_NBoc), 3.70 (2H, ddd, *J* = 13.0, 4.8 and 4.8 Hz, CH_2_C*H*_2_NBoc), 5.13 (1H, tt, *J* = 8.3, 3.8 Hz, ArOC*H*), 6.81 (1H, d, *J* = 8.9 Hz, H-3″), 7.45 (1H, s, H-3), 7.52 (1H, dd, *J* = 8.7, 1.3 Hz, H-5′), 7.59 (1H, s, H-5), 7.78 (1H, dd, *J* = 8.7, 8.5 Hz, H-4′), 7.98 (1H, dd, *J* = 8.9, 2.7 Hz, H-4″), 8.44 (1H, d, *J* = 2.7 Hz, H-6″), 10.10 (1H, s, CON*H*Ar), 12.73 (1H, s, NH-pyrrole); ^13^C NMR (126 MHz, DMSO-*d*_6_) δ 28.1 (C(*C*H_3_)_3_), 30.6 (*C*H_2_CH_2_NBoc), 40.7 (CH_2_*C*H_2_NBoc), 70.0 (ArO*C*H), 78.7 (O*C*(CH_3_)_3_), 110.8 (C-3″), 111.0 (C-3), 119.3 (d, *J* = 18.1 Hz, C-3′), 124.7 (C-2 or C-4), 126.9 (d, *J* = 3.4 Hz, C-5′), 128.6 (C-2 or C-4), 129.2 (d, *J* = 23.3 Hz, C-1′), 129.2 (d, *J* = 5.3 Hz, C-6′), 129.5 (C-5″), 130.0 (C-5), 131.8 (C-4′), 132.5 (C-4″), 138.6 (C-6″), 153.8 (d, *J* = 248.3 Hz, C-2′), 153.9 (*C*O_2_N), 158.4 (*C*ONHAr or C-2″), 158.7 (*C*ONHAr or C-2″), 182.6 (ArCO); ^19^F NMR (471 MHz, DMSO-*d*_6_) δ −116.7 (ArF); MS (ES^–^) *m*/*z* 575.3 [M(^35^Cl^35^Cl)-H]^−^, 577.3 [M(^35^Cl^37^Cl)-H]^−^; HRMS (NSI) calcd for C_27_H_28_Cl_2_FN_4_O_5_ [M(^35^Cl^35^Cl) + H]^+^ 577.1415, found 577.1408.

##### 4-(3,6-Dichloro-2-fluorobenzoyl)-*N*-(6-((1-methylpiperidin-4-yl)oxy)pyridin-3-yl)-1*H*-pyrrole-2-carboxamide (**33k**)

Prepared according to general procedure H using *tert*-butyl 4-((5-(4-(3,6-dichloro-2-fluorobenzoyl)-1*H*-pyrrole-2-carboxamido)pyridin-2-yl)oxy)piperidine-1-carboxylate (33e) (100 mg, 0.17 mmol), formic acid (0.85 mL), and formaldehyde (37% wt in water) (52 μL, 0.69 mmol). The crude yellow solid was purified by MPLC on NH_2_ SiO_2_ with a gradient elution from 0 to 3% MeOH/CH_2_Cl_2_ to yield a white solid (60 mg, 71%); *R*_f_ 0.28 (NH_2_ SiO_2_, 3% MeOH/CH_2_Cl_2_); mp 189.5–191.5 °C; λ_max_ (EtOH)/nm 263.0; IR ν_max_/cm^–1^ 3357, 2981, 2971, 1670, 1635, 1588, 1528, 1485, 1450, 1289, 1275, 1231; ^1^H NMR (500 MHz, DMSO-*d*_6_) δ 1.64 (2H, dddd, *J* = 12.9, 9.3, 9.3 and 3.6 Hz, C*H*_2_CH_2_NMe), 1.89–2.00 (2H, m, C*H*_2_CH_2_NMe), 2.14 (2H, dd, *J* = 12.9, 12.9 Hz, CH_2_C*H*_2_NMe), 2.17 (3H, s, NC*H*_3_), 2.63 (2H, ddd, *J* = 12.9, 4.5 and 4.5 Hz, CH_2_C*H*_2_NMe), 4.92 (1H, tt, *J* = 8.6, 3.9 Hz, ArOC*H*), 6.79 (1H, d, *J* = 8.9 Hz, H-5″), 7.45 (1H, d, *J* = 1.9 Hz, H-3), 7.52 (1H, dd, *J* = 8.8, 1.4 Hz, H-5′), 7.59 (1H, d, *J* = 1.9 Hz, H-5), 7.78 (1H, dd, *J* = 8.8, 8.4 Hz, H-4′), 7.96 (1H, dd, *J* = 8.9, 2.7 Hz, H-4″), 8.43 (1H, d, *J* = 2.7 Hz, H-2″), 10.09 (1H, s, CON*H*Ar), 12.72 (1H, s, NH-pyrrole); ^13^C NMR (126 MHz, DMSO-*d*_6_) δ 30.7 (*C*H_2_CH_2_NMe), 45.8 (NC*H*_3_), 52.8 (CH_2_*C*H_2_NMe), 70.2 (ArO*C*H), 110.8 (C-5″), 111.0 (C-3), 119.3 (d, *J* = 18.2 Hz, C-3′), 124.7 (C-2 or C-4), 126.9 (d, *J* = 3.8 Hz, C-5′), 128.6 (C-2 or C-4), 129.2 (d, *J* = 24.0 Hz, C-1′ and C-3″), 129.2 (d, *J* = 5.2 Hz, C-6′), 130.0 (C-5), 131.8 (C-4′), 132.5 (C-4″), 138.6 (C-2″), 153.8 (d, *J* = 248.6 Hz, C-2′), 158.4 (*C*ONHAr or C-6″), 158.9 (*C*ONHAr or C-6″), 182.6 (ArCO); ^19^F NMR (471 MHz, DMSO-*d*_6_) δ −116.7 (ArF); MS (ES^–^) *m*/*z* 489.3 [M(^35^Cl^35^Cl)-H]^−^, 491.2 [M(^35^Cl^37^Cl)-H]^−^; HRMS (NSI) calcd for C_23_H_22_Cl_2_FN_4_O_3_ [M(^35^Cl^35^Cl) + H]^+^ 491.1048, found 491.1038.

##### 1*H*-Pyrazol-4-amine^47^ (**28a**)

Prepared according to general procedure D using 4-nitropyrazole (500 mg, 4.40 mmol) and MeOH (30 mL) to give a red gum (360 mg, 98%); λ_max_ (EtOH)/nm 238; IR ν_max_/cm^–1^ 3374, 3114, 2955, 2891, 2842, 1585; ^1^H NMR (500 MHz; DMSO-*d*_6_) δ_H_ 7.03 (2H, s, 2 × H-pyrazole); ^13^C NMR (125 MHz; DMSO-*d*_6_) δ_C_ 122.5, 130.0; MS: No mass ion detected.

##### 4-(3,6-Dichloro-2-fluorobenzoyl)-*N*-(1*H*-pyrazol-4-yl)-1*H*-pyrrole-2-carboxamide (**34a**)

Prepared according to general procedure E using amine **28a** (69 mg, 0.83 mmol), carboxylic acid **31b** (100 mg, 0.33 mmol), cyanuric fluoride (20 μL, 0.24 mmol), pyridine (27 μL, 0.34 mmol), and MeCN (2 mL) with stirring at RT for 18 h. Purification by MPLC on SiO_2_ with a gradient elution from 2 to 8% MeOH/EtOAc gave a yellow solid (78 mg, 64%); *R*_f_ 0.35 (NH_2_ SiO_2_, 5% MeOH/EtOAc); mp 300–304 °C; λ_max_ (EtOH)/nm 255, 223; IR ν_max_/cm^–1^ 3361.0, 3241.5 br, 2971.9, 1636.3, 1586.2; ^1^H NMR (500 MHz; DMSO-*d*_6_) δ_H_ 7.36 (1H, s, H-pyrrole), 7.56 (1H, dd, *J* = 1.1 & 8.5 Hz, H-5′), 7.60 (1H, s, H-pyrrole), 7.57 (1H, s, H-pyrazole), 7.82 (1H, app t, *J* = 8.5 Hz, H-4′), 7.97 (1H, s, H-pyrazole), 10.29 (1H, s, CO-NH), 12.60–12.73 (2H, m, NH-pyrrole and NH-pyrazole); ^13^C NMR (125 MHz; DMSO-*d*_6_) δ_C_ 110.1 (CH-pyrrole), 119.3 (d, *J*_CF_ = 18.1 Hz, C-3′), 120.8 (CH-pyrazole), 124.7 (C-pyrrole), 126.9 (d, *J*_CF_ = 4.1 Hz, C-5′), 128.8 (C-pyrrole), 129.2 (d, *J*_CF_ = 5.2 Hz, C-6′), 129.2 (d, *J*_CF_ = 22.7 Hz, C-1′), 129.5 (C-pyrrole), 131.0 (CH-pyrazole), 131.8 (C-4′), 153.8 (d, *J*_CF_ = 248.8 Hz, C-2′), 156.9 (CO-NH), 182.6 (CO); ^19^F NMR (470 MHz; DMSO-*d*_6_) δ_F_ −116.7; HRMS calcd for C_15_H_10_^35^Cl_2_F_1_N_4_O_2_ [M + H]^+^ 367.0159, found 367.0158.

##### 1-Methyl-4-nitro-1*H*-pyrazole^48^ (**27a**)

Prepared according to general procedure G using MeOH (11 μL, 2.65 mmol), PPh_3_ (1.04 g, 4.0 mmol), 4-nitropyrazole (300 mg, 2.7 mmol), and DEAD (626 μL, 4.0 mmol) in THF (5 mL) with stirring at RT for 18 h. The residue was purified by MPLC on SiO_2_ with a gradient elution from 20 to 50% EtOAc/petrol to give an impure product, which was repurified by MPLC on SiO_2_ with a gradient elution from 10 to 40% EtOAc/petrol to give a white solid (227 mg, 67%); *R*_f_ 0.80 (SiO_2_, EtOAc); mp 90–94 °C (Lit.^48^ 91–92 °C); λ_max_ (EtOH)/nm 266; IR ν_max_/cm^–1^ 1504, 1310; ^1^H NMR (500 MHz; DMSO-*d*_6_) δ_H_ 3.92 (3H, s, CH_3_), 8.25 (1H, s, H-pyrazole), 8.86 (1H, s, H-pyrazole); ^13^C NMR (125 MHz; DMSO-*d*_6_) δ_C_ 39.6 (CH_3_), 131.0 (CH-pyrazole), 134.8 (CH-pyrazole), 135.5 (C-NO_2_). MS (ES+) 128.1 [M + H]^+^.

##### 1-Methyl-1*H*-pyrazol-4-amine (**28b**)

Prepared according to general procedure D using nitropyrazole **27a** (200 mg, 1.57 mmol) and MeOH (10 mL) for 2 h to give an orange oil (150 mg, 98%); *R*_f_ 0.10 (SiO_2_, 100% EtOAc); λ_max_ (EtOH)/nm 244; IR ν_max_/cm^–1^ 3322, 3111; ^1^H NMR (500 MHz; DMSO-*d*_6_) δ_H_ 3.68 (3H, s, CH_3_), 3.82 (2H, br s, NH_2_), 6.90 (1H, d, *J* = 0.6 Hz, H-pyrazole), 7.01 (1H, d, *J* = 0.6 Hz, H-pyrazole); ^13^C NMR (125 MHz; DMSO-*d*_6_) δ_C_ 38.3 (CH_3_), 117.2 (CH-pyrazole), 128.9 (CH-pyrazole), 131.0 (C-pyrazole); MS: No mass ion detected.

##### 4-(3,6-Dichloro-2-fluorobenzoyl)-*N*-(1-methyl-1*H*-pyrazol-4-yl)-1*H*-pyrrole-2-carboxamide (**34b**)

Prepared according to general procedure E using amine **28b** (130 mg, 1.3 mmol) and carboxylic acid **31b** (162 mg, 0.54 mmol), cyanuric fluoride (32 μL, 0.37 mmol), pyridine (43 μL, 0.50 mmol), and MeCN (2 mL) with stirring at RT for 18 h. Purification by MPLC on SiO_2_ with a gradient elution from 30 to 60% EtOAc/petrol gave a yellow solid (120 mg, 59%); *R*_f_ 0.10 (SiO_2_, 50% EtOAc/petrol); mp 216–219 °C; λ_max_ (EtOH)/nm 252, 225; IR ν_max_/cm^–1^ 3195, 3121, 2938, 1630; ^1^H NMR (500 MHz; DMSO-*d*_6_) δ_H_ 3.85 (3H, s, CH_3_), 7.36 (1H, s, H-pyrrole), 7.54 (1H, s, H-pyrazole), 7.56 (1H, dd, *J* = 1.2 and 8.5 Hz, H-5′), 7.59 (1H, s, H-pyrrole), 7.82 (1H, app t, *J* = 8.5 Hz, H-4′), 7.98 (1H, s, H-pyrazole), 10.28 (1H, s, CO-NH), 12.68 (NH-pyrrole); ^13^C NMR (125 MHz; DMSO-*d*_6_) δ_C_ 38.7 (CH_3_), 110.2 (CH-pyrrole), 119.3 (d, *J*_CF_ = 18.2 Hz, C-3′), 121.2 (CH-pyrazole), 121.4 (C-pyrazole), 124.7 (C-pyrrole), 126.9 (d, *J*_CF_ = 3.7 Hz, C-5′), 128.7 (C-pyrrole), 129.2 (d, *J*_CF_ = 23.2 Hz, C-1′), 129.2 (d, *J*_CF_ = 5.2 Hz, C-6′), 129.5 (C-pyrrole), 129.9 (CH-pyrazole), 131.8 (C-4′), 153.8 (d, *J*_CF_ = 248.4 Hz, C-2′), 156.8 (CO-NH), 182.6 (CO); ^19^F NMR (470 MHz; DMSO-*d*_6_) δ_F_ −116.7; HRMS calcd for C_16_H_12_^35^Cl_2_F_1_N_4_O_2_ [M + H]^+^ 381.0319, found 381.0318.

##### 4-(3,6-Dichloro-2-fluorobenzoyl)-*N*-(1-methyl-1*H*-pyrazol-3-yl)-1*H*-pyrrole-2-carboxamide (**34c**)

Prepared according to general procedure C using carboxylic acid **31b** (100 mg, 0.33 mmol), 3-amino-5-methylpyrazole (113 mg, 1.16 mmol), PCl_3_ (29 μL, 0.33 mmol), and MeCN (1.50 mL). Purification by MPLC on SiO_2_ with a gradient elution from 50 to 80% EtOAc/petrol gave a white solid, which was repurified by MPLC on NH_2_ SiO_2_ with a gradient elution from 50 to 80% EtOAc/petrol to give a white solid (85 mg, 67%); *R*_f_ 0.30 (SiO_2_, 75% EtOAc/petrol); mp 240–242 °C; λ_max_ (EtOH)/nm 254sh; IR ν_max_/cm^–1^ 3200, 3131, 1636, 1574; ^1^H NMR (500 MHz; DMSO-*d*_6_) δ_H_ 3.81 (3H, s, CH_3_), 6.57 (1H, d, *J* = 2.2 Hz, H-pyrazole), 7.55 (1H, dd, *J* = 1.4 and 8.6 Hz, H-5′), 7.56 (1H, br s, H-pyrrole), 7.60 (1H, br s, H-pyrrole), 7.62 (1H, d, *J* = 2.2 Hz, H-pyrazole), 7.81 (1H, app t, *J* = 8.6 Hz, H-4′), 10.75 (1H, s, CO-NH), 12.64 (1H, br s, NH-pyrrole); ^13^C NMR (125 MHz; DMSO-*d*_6_) δ_C_ 38.3 (CH_3_), 97.2 (C-4-pyrazole), 111.5 (CH-pyrrole), 119.2 (d, *J*_CF_ = 18.2 Hz, C-3′), 124.7 (C-pyrrole), 126.8 (d, *J*_CF_ = 3.6 Hz, C-5′), 128.5 (CH-pyrrole), 129.2 (d, *J*_CF_ = 5.1 Hz, C-6′), 129.3 (d, *J*_CF_ = 23.2 Hz, C-1′), 129.6 (C-pyrrole), 130.9 (C-5-pyrazole), 131.7 (C-4′), 146.6 (C-3-pyrazole), 153.8 (d, *J*_CF_ = 248.4 Hz, C-2′), 157.4 (CO-NH), 170.3, 182.5 (CO); ^19^F NMR (470 MHz; DMSO-*d*_6_) δ_F_ −116.6; HRMS calcd for C_16_H_12_^35^Cl_2_F_1_N_4_O_2_ [M + H]^+^ 381.0316, found 381.0313.

##### 4-(3,6-Dichloro-2-fluorobenzoyl)-*N*-(3-methylisoxazol-5-yl)-1*H*-pyrrole-2-carboxamide (**34d**)

Prepared according to general procedure C using carboxylic acid **31b** (75 mg, 0.25 mmol), 5-amino-3-methylisoxazole (85 mg, 0.87 mmol), PCl_3_ (22 μL, 0.25 mmol), and MeCN (1 mL). Purification by MPLC on SiO_2_ with a gradient elution from 10 to 40% EtOAc/petrol gave a white solid (32 mg, 34%); *R*_f_ 0.60 (SiO_2_, 40% EtOAc/petrol); mp 228 °C dec.; λ_max_ (EtOH)/nm 294, 240; IR ν_max_/cm^–1^ 3253, 3126, 3055, 1680, 1640; ^1^H NMR (500 MHz; DMSO-*d*_6_) δ_H_ 2.25 (3H, s, CH_3_), 6.29 (1H, s, H-isoxazole), 7.57 (1H, dd, *J* = 1.3 and 8.4 Hz, H-3′), 7.62 (1H, br s, H-pyrrole), 7.74 (1H, s, H-pyrrole), 7.83 (1H, app t, *J* = 8.6 Hz, H-4′), 11.80 (1H, s, CO-NH), 12.94 (1H, s, NH-pyrrole); ^13^C NMR (125 MHz; DMSO-*d*_6_) δ_C_ 11.3 (CH_3_), 89.2 (CH-isoxazole), 113.0 (CH-pyrrole), 119.3 (d, *J*_CF_ = 18.0 Hz, C-3′), 124.9 (C-pyrrole), 126.9 (d, *J*_CF_ = 3.8 Hz, C-5′), 127.1 (C-pyrrole), 129.0 (d, *J*_CF_ = 23.0 Hz, C-1′), 129.2 (d, *J*_CF_ = 5.1 Hz, C-6′), 130.7 (C-pyrrole), 131.9 (C-4′), 153.8 (d, *J*_CF_ = 248.4 Hz, C-2′), 156.3 (CO-NH), 160.7 (C-isoxazole), 161.1 (C-isoxazole), 182.6, (CO); ^19^F NMR (470 MHz; DMSO-*d*_6_) δ_F_ −116.6; HRMS calcd for C_16_H_11_^35^Cl_2_F_1_N_3_O_3_ [M + H]^+^ 382.0156, found 382.0153.

##### 4-(3,6-Dichloro-2-fluorobenzoyl)-*N*-(1-methyl-1*H*-imidazol-4-yl)-1*H*-pyrrole-2-carboxamide (**34e**)

Prepared according to general procedure C using carboxylic acid **31b** (125 mg, 0.33 mmol), 1-methyl-1*H*-imidazol-4-amine (100 mg, 1.03 mmol), PCl_3_ (29 μL, 0.33 mmol), and MeCN (1.50 mL). Purification by MPLC on NH_2_ SiO_2_ with a gradient elution from 0 to 4% MeOH/EtOAc gave a white solid (30 mg, 24%); *R*_f_ 0.70 (5% MeOH/EtOAc); mp 258–260 °C; λ_max_ (EtOH)/nm 227, 250 sh; IR ν_max_/cm^–1^ 3262, 3122, 1657, 1637; ^1^H NMR (500 MHz; DMSO-*d*_6_) δ_H_ 3.68 (3H, CH_3_), 7.33 (1H, s, H-imidazole), 7.47 (1H, s, H-imidazole), 7.52–7.60 (3H, m, H-5′ and 2 × H-pyrrole), 7.81 (1H, app t, *J* = 8.3 Hz, H-4′), 10.67 (1H, s, CO-NH), 12.61 (1H, s, NH-pyrrole); ^13^C NMR (125 MHz; DMSO-*d*_6_) δ_C_ 33.1 (CH_3_), 108.2 (CH-imidazole), 111.2 (CH-pyrrole), 119.2 (d, *J*_CF_ = 18.3 Hz, C-3′), 124.7 (C-pyrrole), 126.8 (d, *J*_CF_ = 3.6 Hz, C-5′), 128.5 (CH-pyrrole), 129.2 (d, *J*_CF_ = 5.4 Hz, C-6′), 129.3 (d, *J*_CF_ = 22.7 Hz, C-1′), 131.7 (C-4′), 133.9 (CH-imidazole), 137.6 (C-imidazole), 153.8 (d, *J*_CF_ = 248.4 Hz, C-2′), 156.6 (CO-NH), 182.6 (CO); ^19^F NMR (470 MHz; DMSO-*d*_6_) δ_F_ −116.2; HRMS calcd for C_16_H_12_^35^Cl_2_F_1_N_4_O_2_ [M + H]^+^ 381.0316, found 381.0315.

##### *tert*-Butyl-4-(4-nitro-1*H*-pyrazol-1-yl)piperidine-1-carboxylate^49,50^ (**27b**)

Prepared according to general procedure G using Boc-4-piperidinol (889 mg, 4.4 mmol), PPh_3_ (1.73 g, 6.6 mmol), 4-nitropyrazole (500 mg, 4.4 mmol), and DEAD (1.04 mL, 5.3 mmol) in THF (10 mL). The residue was purified by MPLC on SiO_2_ with a gradient elution from 20 to 40% EtOAc/petrol to give a white solid (880 mg, 67%); *R*_f_ 0.50 (SiO_2_, 20% EtOAc/petrol); mp 116–118 °C; λ_max_ (EtOH)/nm 274; IR ν_max_/cm^–1^ 3099, 2971, 2875, 1671, 1301; ^1^H NMR (500 MHz; DMSO-*d*_6_) δ_H_ 1.45 (9H, s, C(C*H*_3_)_3_), 1.85 (2H, qd, *J* = 4.2 and 11.6 Hz, 2 × H-piperidine), 2.04–2.11 (2H, m, 2 × H-piperidine), 2.80–3.07 (2H, m, 2 × H-piperidine), 3.99–4.19 (2H, m, 2 × H-piperidine), 4.50 (1H, tt, *J* = 4.2 and 11.6 Hz, CH_2_C*H*CCH_2_-piperidine), 8.32 (1H, s, H-pyrazole), 9.00 (1H, s, H-pyrazole); ^13^C NMR (125 MHz; DMSO-*d*_6_) δ_C_ 28.0 (C(*C*H_3_)_3_), 31.3 (2 × CH_2_-piperidine), 42.4 (2 × CH_2_-piperidine), 59.5 (CH-N-piperidine), 78.9 (*C*(CH_3_)_3_), 128.9 (C-pyrazole), 134.8 (C-pyrazole), 135.3 (C-pyrazole), 153.7 (CO); HRMS calcd for C_13_H_19_N_4_O_4_ [M – H]^−^ 295.1412, found 295.1408.

##### *tert*-Butyl-4-(4-amino-1*H*-pyrazol-1-yl)piperidine-1-carboxylate^49,50^ (**28c**)

Prepared according to general procedure D using nitropyrazole **27b** (310 mg, 1.05 mmol) in MeOH (15 mL) and EtOAc (15 mL) for 3 h to give a white solid (279 mg, 100%); *R*_f_ 0.40 (NH_2_ SiO_2_, 100% EtOAc); mp 86–89 °C dec.; λ_max_ (EtOH)/nm 248; IR ν_max_/cm^–1^ 3238, 2972, 2930, 2865, 1697, 1669; ^1^H NMR (500 MHz; DMSO-*d*_6_) δ_H_ 1.45 (9H, s, C(C*H*_3_)_3_), 1.71 (2H, qd, *J* = 4.2 and 11.7 Hz, 2 × H-piperidine), 1.90–1.97 (2H, m, 2 × H-piperidine), 2.75–3.04 (2H, m, 2 × H-piperidine), 3.80 (2H, br s, NH_2_), 3.96–4.09 (2H, m, 2 × H-piperidine), 4.16 (1H, tt, *J* = 4.2 and 11.7 Hz, CH_2_C*H*CCH_2_-piperidine), 6.94 (1H, s, H-pyrazole), 7.10 (1H, s, H-pyrazole); ^13^C NMR (125 MHz; DMSO-*d*_6_) δ_C_ 28.0 (C(*C*H_3_)_3_), 31.9 (2 × CH_2_-piperidine), 43.2 (2 × CH_2_-piperidine), 57.6 (CHN-piperidine), 78.7 (*C*(CH_3_)_3_), 114.4 (CH-pyrazole), 128.9 (CH-pyrazole), 131.1 (C-pyrazole), 153.8 (CO); HRMS calcd for C_13_H_23_N_4_O_2_ [M + H]^+^ 267.1816, found 267.1815.

##### *tert*-Butyl-4-(4-(4-(3,6-dichloro-2-fluorobenzoyl)-1*H*-pyrrole-2-carboxamido)-1*H*-pyrazol-1-yl)piperidine-1-carboxylate (**34f**)

Prepared according to general procedure E using amine **28c** (200 mg, 0.75 mmol), carboxylic acid **31b** (91 mg, 0.30 mmol), cyanuric fluoride (18 μL, 0.21 mmol), pyridine (24 μL, 0.30 mmol), and MeCN (2 mL). Purification by MPLC on SiO_2_ with a gradient elution from 40 to 70% EtOAc/petrol gave a yellow oil (125 mg, 76%); *R*_f_ 0.35 (SiO_2_, 70% EtOAc/petrol); mp 144 °C dec.; IR ν_max_/cm^–1^ 3123 br, 2975, 1637; ^1^H NMR (500 MHz; DMSO-*d*_6_) δ_H_ 1.46 (9H, s, C(CH_3_)_3_), 1.80 (2H, app qd, *J* = 4.1 and 11.6 Hz, 2 × H-piperidine), 1.97–2.04 (2H, m, 2 × H-piperidine), 2.82–3.02 (2H, m, 2 × H-piperidine), 4.01–4.12 (2H, m, 2 × H-piperidine), 4.38 (1H, tt, *J* = 3.9 and 11.5 Hz, CH_2_C*H*CCH_2_-piperidine), 7.36 (1H, s, CH-pyrrole), 7.55 (1H, dd, *J* = 1.1 and 8.6 Hz, H-5′), 7.58–7.62 (2H, m, H-pyrrole and H-pyrazole), 7.82 (1H, app t, *J* = 8.6 Hz, H-4′), 8.02 (1H, s, H-pyrazole), 10.30 (1H, s, CO-NH), 12.67 (1H, s, NH-pyrazole); ^13^C NMR (125 MHz; DMSO-*d*_6_) δ_C_ 28.0 (C(*C*H_3_)_3_), 31.9 (2 × CH_2_-piperidine), 42.8 (2 × CH_2_-piperidine), 58.0 (CHN-piperidine), 78.8 (*C*(CH_3_)_3_), 110.2 (CH-pyrrole), 118.9 (CH-pyrazole), 119.3 (d, *J*_CF_ = 18.1 Hz, C-3′), 120.9 (C-pyrazole), 124.7 (C-pyrrole), 126.9 (d, *J*_CF_ = 4.1 Hz, C-5′), 128.7 (C-pyrrole), 129.2 (*J*_CF_ = 23.1 Hz, C-1′), 129.2 (*J*_CF_ = 5.1 Hz, C-6′), 129.6 (C-pyrrole), 130.0 (CH-pyrazole), 131.8 (C-4′), 153.8 (*J*_CF_ = 248.4 Hz, C-2′), 153.8 (CO-carbamate), 156.8 (CO-NH), 182.6 (CO); ^19^F NMR (470 MHz; DMSO-*d*_6_) δ_F_ −116.7; HRMS calcd for C_25_H_27_^35^Cl_2_F_1_N_5_O_4_ [M + H]^+^ 550.1419, found 550.1414.

##### 4-(3,6-Dichloro-2-fluorobenzoyl)-*N*-(1-(piperidin-4-yl)-1*H*-pyrazol-4-yl)-1*H*-pyrrole-2-carboxamide (**34h**)

Prepared according to general procedure J using carbamate **34f** (110 mg, 0.20 mmol), Et_3_SiH (80 μL, 0.50 mmol), TFA (1 mL), and CH_2_Cl_2_ (1 mL). The residue was purified by MPLC on NH_2_ SiO_2_ with a gradient elution from 1 to 4% MeOH/CH_2_Cl_2_ to give a white solid (62 mg, 69%); *R*_f_ 0.30 (NH_2_ SiO_2_, 10% MeOH/CH_2_Cl_2_); mp 199 °C dec.; λ_max_ (EtOH)/nm 255, 223; IR ν_max_/cm^–1^ 3122 br, 2949, 1633, 1592; ^1^H NMR (500 MHz; DMSO-*d*_6_) δ_H_ 1.78 (2H, app qd, *J* = 3.9 and 11.8 Hz, 2 × H-piperidine), 1.91–1.98 (2H, m, 2 × H-piperidine), 2.57–2.66 (2H, m, 2 × H-piperidine), 3.01–3.11 (2H, m, 2 × H-piperidine), 4.21 (1H, tt, *J* = 4.1 and 11.7 Hz, N-CH-piperidine), 7.35 (1H, s, H-pyrrole), 7.56 (1H, d, *J* = 8.6 Hz, H-5′), 7.57–7.61 (2H, m, H-pyrrole and H-pyrazole), 7.82 (1H, app t, *J* = 8.6 Hz, H-4′), 7.98 (1H, s, H-pyrazole), 10.27 (1H, s, CO-NH); ^13^C NMR (125 MHz; DMSO-*d*_6_) δ_C_ 33.5 (2 × CH_2_-piperidine), 45.1 (2 × CH_2_-piperidine), 59.0 (CHN-piperidine), 110.2 (CH-pyrrole), 118.4 (CH-pyrazole), 119.3 (d, *J*_CF_ = 18.0 Hz, C-3′), 120.8 (C-pyrazole), 124.7 (C-pyrrole), 126.9 (d, *J*_CF_ = 3.6 Hz, C-5′), 128.8 (CH-pyrrole), 129.2 (*J*_CF_ = 22.9 Hz, C-1′), 129.2 (*J*_CF_ = 4.8 Hz, C-6′), 129.7 (C-pyrrole), 130.0 (CH-pyrazole), 131.8 (C-4′), 153.8 (*J*_CF_ = 248.5 Hz, C-2′), 156.9 (CO-NH), 182.5 (CO); ^19^F NMR (470 MHz; DMSO-*d*_6_) δ_F_ −116.7; HRMS calcd for C_20_H_19_^35^Cl_2_F_1_N_5_O_2_ [M + H]^+^ 450.0894, found 450.0888.

##### 4-(3,6-Dichloro-2-fluorobenzoyl)-*N*-(1-(1-methylpiperidin-4-yl)-1*H*-pyrazol-4-yl)-1*H*-pyrrole-2-carboxamide (**34i**)

Prepared according to general procedure H using carbamate **34f** (75 mg, 0.137 mmol), formic acid (1.5 mL), and formaldehyde (44 μL, 0.55 mmol). The residue was purified by MPLC on NH_2_ SiO_2_ with a gradient elution from 2 to 6% MeOH/EtOAc to give a white solid (63 mg, 100%); *R*_f_ 0.25 (5% MeOH/EtOAc); mp 220–222 °C; λ_max_ (EtOH)/nm 252; IR ν_max_/cm^–1^ 3121, 2938, 2788, 1631; ^1^H NMR (500 MHz; DMSO-*d*_6_) δ_H_ 1.93–2.03 (4H, m, 4 × H-piperidine), 2.09–2.21 (2H, m, 2 × H-piperidine), 2.28 (3H, s, NCH_3_), 2.88–2.98 (2H, m, 2 × H-piperidine), 4.10–4.211 (1H, m, N-CH-piperidine), 7.36 (1H, s, H-pyrrole), 7.56 (1H, dd, *J* = 1.3 and 8.6 Hz, H-5′), 7.58–7.61 (2H, m, H-pyrrole and H-pyrazole), 7.82 (1H, app t, *J* = 8.6 Hz, H-4′), 8.01 (1H, s, H-pyrazole), 10.29 (1H, s, CO-NH), 12.67 (1H, s, NH-pyrazole); ^13^C NMR (125 MHz; DMSO-*d*_6_) δ_C_ 31.8 (2 × CH_2_-piperidine), 45.5 (N-CH_3_), 54.0 (2 × CH_2_-piperidine), 57.8 (CHN-piperidine), 110.1 (CH-pyrrole), 118.8 (CH-pyrazole), 119.3 (d, *J*_CF_ = 18.2 Hz, C-3′), 120.8 (C-pyrazole), 124.7 (C-pyrrole), 126.9 (d, *J*_CF_ = 3.7 Hz, C-5′), 128.7 (CH-pyrrole), 129.2 (d, *J*_CF_ = 23.1 Hz, C-1′), 129.2 (d, *J*_CF_ = 4.8 Hz, C-6′), 129.5 (C-pyrrole), 129.9 (CH-pyrazole), 131.8 (C-4′), 154.8 (d, *J*_CF_ = 248.5 Hz, C-2′), 156.8 (CO-NH), 182.6 (CO); ^19^F NMR (470 MHz; DMSO-*d*_6_) δ_F_ −116.7; HRMS calcd for C_21_H_21_^35^Cl_2_F_1_N_5_O_2_ [M + H]^+^ 464.1051, found 464.1043.

##### *tert*-Butyl-4-((4-nitro-1*H*-pyrazol-1-yl)methyl)piperidine-1-carboxylate (**27c**)

Prepared according to general procedure G using Boc-4-piperidinemethanol (951 mg, 4.4 mmol), PPh_3_ (1.74 g, 6.6 mmol), 4-nitropyrazole (500 mg, 4.4 mmol), and DEAD (1.04 mL, 6.6 mmol) in THF (10 mL). The residue was purified by MPLC on SiO_2_ with a gradient elution from 10 to 60% EtOAc/petrol to give a white solid (1.135 g, 82%); *R*_f_ 0.35 (SiO_2_, 40% EtOAc/petrol); mp 158–160 °C; λ_max_ (EtOH)/nm 271; IR ν_max_/cm^–1^ 1665, 1506, 1312.; ^1^H NMR (500 MHz; DMSO-*d*_6_) δ_H_ 1.10 (2H, qd, *J* = 4.1 and 12.6 Hz, 2 × H-piperidine), 1.42 (9H, s, C(CH_3_)_3_), 1.45–1.53 (2H, m, 2 × H-piperidine), 2.02–2.12 (1H, m, H-piperidine), 2.61–2.82 (m, 2 × CH-N-piperidine), 3.88–4.01 (2H, m, 2 × CH-N-piperidine), 4.13 (2H, d, *J* = 7.2 Hz, N-C*H*_2_-CH), 8.31 (H-pyrazole), 8.92 (H-pyrazole); ^13^C NMR (125 MHz; DMSO-*d*_6_) δ_C_ 28.0 (C(*C*H_3_)_3_), 28.7 (C-piperidine), 35.9 (2 × C-piperidine), 42.8 (2 × C-piperidine), 57.2 (N-*C*H_2_-CH), 78.5 (*C*(CH_3_)_3_), 130.8 (CH-pyrazole), 134.7 (C-4-pyrazole), 135.6 (CH-pyrazole), 153.8 (CO); HRMS calcd for C_14_H_21_O_4_N_4_ [M – H]^−^ 309.1568, found 309.1565.

##### *tert*-Butyl-4-((4-amino-1*H*-pyrazol-1-yl)methyl)piperidine-1-carboxylate (**28d**)

Prepared according to general procedure D using nitropyrazole **27c** (1.1 g, 3.5 mmol) in MeOH (40 mL) and EtOAc (40 mL) for 5 h to give an orange solid (945 mg, 95%); *R*_f_ 0.35 (NH_2_ SiO_2_, 100% EtOAc); mp 104–107 °C; λ_max_ (EtOH)/nm 247; IR ν_max_/cm^–1^ 2966, 2929, 1672; ^1^H NMR (500 MHz; DMSO-*d*_6_) δ_H_ 1.04 (2H, qd, *J* = 4.3 and 12.3 Hz, 2 × H-piperidine), 1.42 (9H, s, C(CH_3_)_3_), 1.42–1.49 (2H, m, 2 × H-piperidine), 1.85–1.97 (1H, m, H-piperidine), 2.58–2.82 (m, 2 × CH-N-piperidine), 3.79–3.87 (4H, m, N-C*H*_2_-CH and NH_2_), 3.88–3.99 (2H, m, 2 × H-N-piperidine), 6.92 (H-pyrazole), 7.03 (H-pyrazole); ^13^C NMR (125 MHz; DMSO-*d*_6_) δ_C_ 28.1 (C(*C*H_3_)_3_), 29.0 (C-piperidine), 36.7 (2 × C-piperidine), 42.5 (2 × C-piperidine), 56.2 (N-*C*H_2_-CH), 78.4 (*C*(CH_3_)_3_), 117.0 (C-2-pyrazole), 129.2 (C-4-pyrazole), 130.5 (C-3-pyrazole), 153.8 (CO); HRMS calcd for C_14_H_25_N_4_O_4_ [M + H]^+^ 281.1972, found 281.1972.

##### *tert*-Butyl-4-((4-(4-(3,6-dichloro-2-fluorobenzoyl)-1*H*-pyrrole-2-carboxamido)-1*H*-pyrazol-1-yl)methyl)piperidine-1-carboxylate (**34g**)

Prepared according to general procedure E using amine **28d** (450 mg, 1.6 mmol), carboxylic acid **31a** (195 mg, 0.64 mmol), cyanuric fluoride (18 μL, 0.21 mmol), and pyridine (52 μL, 0.64 mmol) in MeCN (2 mL) with stirring at RT for 18 h. Purification by MPLC on SiO_2_ with a gradient elution from 50 to 100% EtOAc/petrol gave a yellow solid (214 mg, 59%); *R*_f_ 0.10 (NH_2_ SiO_2_, 100% EtOAc); mp 211 °C dec.; λ_max_ (EtOH)/nm 254; IR ν_max_/cm^–1^ 3126, 2977, 2932, 2860, 1645, 1592; ^1^H NMR (500 MHz; DMSO-*d*_6_) δ_H_ 1.09 (2H, qd, *J* = 3.6 and 12.3 Hz, 2 × CH-piperidine), 1.42 (9H, s, C(CH_3_)_3_), 1.44–1.52 (2H, m, 2 × H-piperidine), 1.94–2.07 (1H, m, H-piperidine), 2.60–2.80 (m, 2 × CH-N-piperidine), 3.87–4.01 (2H, m, 2 × CH-N-piperidine), 4.02 (2H, d, *J* = 7.1 Hz, N-C*H*_2_-CH), 7.36 (1H, s, H-pyrrole), 7.56 (1H, dd, *J* = 1.3 and 8.6 Hz, H-4′), 7.58 (s, H-pyrazole), 7.60 (1H, s, H-pyrrole), 7.82 (1H, app t, *J* = 8.6 Hz, H-3′), 7.99 (1H, s, H-pyrazole), 10.29 (1H, s, CO-NH), 12.66 (1H, s, NH-pyrrole); ^13^C NMR (125 MHz; DMSO-*d*_6_) δ_C_ 28.1 (C(*C*H_3_)_3_), 30.0 (C-piperidine), 36.6 (2 × C-piperidine), 42.8 (2 × C-piperidine), 56.3 (N-*C*H_2_-CH), 78.5 (*C*(CH_3_)_3_), 110.1 (CH-pyrrole), 119.3 (d, *J*_CF_ = 18.0 Hz, C-3′), 120.9 (C-pyrazole), 121.2 (C-pyrazole), 124.7 (C-pyrrole), 126.9 (d, *J*_CF_ = 3.8 Hz, C-5′), 128.7 (C-pyrrole), 129.2 (d, *J*_CF_ = 23.0 Hz, C-1′), 129.2 (d, *J*_CF_ = 5.1 Hz, C-6′), 129.5 (C-pyrrole), 130.2 (C-3-pyrazole), 131.8 (C-4′), 153.8 (CO-carbamate), 153.8 (d, *J*_CF_ = 248.5 Hz, C-2′), 156.8 (CO-NH), 182.6 (CO); ^19^F NMR (470 MHz; DMSO-*d*_6_) δ_F_ −116.7; HRMS calcd for C_26_H_29_^35^Cl_2_F_1_N_5_O_4_ [M + H]^+^ 564.1575, found 564.1566.

##### 4-(3,6-Dichloro-2-fluorobenzoyl)-*N*-(1-(piperidin-4-ylmethyl)-1*H*-pyrazol-4-yl)-1*H*-pyrrole-2-carboxamide (**34j**)

Prepared according to general procedure J using carbamate **34y** (200 mg, 0.35 mmol), Et_3_SiH (283 μL, 1.77 mmol), TFA (1.5 mL), and CH_2_Cl_2_ (1.5 mL). Purification by MPLC on NH_2_ SiO_2_ with a gradient elution from 3 to 15% MeOH/EtOAc gave a white solid (106 mg, 64%); *R*_f_ 0.05 (SiO_2_, 5% MeOH/EtOAc); mp 147 °C dec.; λ_max_ (EtOH)/nm 254; IR ν_max_/cm^–1^ 2926, 1633, 1592; ^1^H NMR (500 MHz; DMSO-*d*_6_) δ_H_ 1.09 (2H, qd, *J* = 3.6 and 12.0 Hz, 2 × H-piperidine), 1.39–1.47 (2H, m, 2 × H-piperidine), 1.82–1.94 (1H, m, H-piperidine), 2.43 (td, *J* = 12.0 and 2.2 Hz, 2 × CH-N-piperidine), 2.90–2.97 (2H, m, 2 × CH-N-piperidine), 3.97 (2H, d, *J* = 7.1 Hz, pyrazole-CH_2_-piperidine), 7.33 (1H, s, H-pyrrole), 7.55 (1H, dd, *J* = 1.3 and 8.7 Hz, H-4′), 7.57 (1H, s, H-pyrazole), 7.58 (1H, s, H-pyrrole), 7.82 (1H, app t, *J* = 8.7 Hz, H-3′), 7.97 (1H, s, H-pyrazole), 10.26 (1H, s, CO-NH); ^13^C NMR (125 MHz; DMSO-*d*_6_) δ_C_ 30.2 (C-piperidine), 37.3 (2 × C-piperidine), 45.5 (2 × C-piperidine), 57.2 (pyrazole-*C*H_2_-piperidine), 110.2 (CH-pyrrole), 119.3 (d, *J*_CF_ = 18.2 Hz, C-3′), 120.9 (C-pyrazole), 121.1 (C-pyrazole), 124.7 (C-pyrrole), 126.8 (d, *J*_CF_ = 3.8 Hz, C-5′), 129.1 (C-pyrrole), 129.2 (d, *J*_CF_ = 11.6 Hz, C-6′), 129.2 (CH-pyrrole), 129.3 (d, *J*_CF_ = 20.5 Hz, C-1′), 130.0 (C-pyrazole), 131.7 (C-4′), 153.8 (d, *J*_CF_ = 248.86 Hz, C-2′), 157.0 (CO-NH), 182.4 (CO); ^19^F NMR (470 MHz; DMSO-*d*_6_) δ_F_ −116.6; HRMS calcd for C_21_H_21_^35^Cl_2_F_1_N_5_O_2_ [M + H]^+^ 464.1051, found 464.1038.

##### 1-Methyl-4-((4-nitro-1*H*-pyrazol-1-yl)methyl)piperidine (**27d**)

Prepared according to general procedure G using 1-methyl-4-piperidinemethanol (349 μL, 2.7 mmol), PPh_3_ (1.04 g, 4.0 mmol), 4-nitropyrazole (300 mg, 2.7 mmol), and DEAD (626 μL, 4.0 mmol) in THF (6 mL). Purification by MPLC on NH_2_ SiO_2_ with a gradient elution from 30 to 80% CH_2_Cl_2_/petrol gave a white solid. This solid was dissolved in 5% MeOH/CH_2_Cl_2_ and passed through an SCX ion-exchange column, eluting with 5% MeOH/CH_2_Cl_2_ followed by 80:20:2 CH_2_Cl_2_/MeOH/NH_4_OH to give a beige solid (200 mg, 34%). *R*_f_ 0.30 (NH_2_ SiO_2_, 100% CH_2_Cl_2_); mp 70–73 °C; λ_max_ (EtOH)/nm 274; IR ν_max_/cm^–1^ 3067, 2943, 2903, 2797, 1511, 1312; ^1^H NMR (500 MHz; DMSO-*d*_6_) δ_H_ 1.24 (2H, qd, *J* = 3.6 and 12.5 Hz, 2 × H-piperidine), 1.42–1.50 (2H, m, 2 × H-piperidine), 1.77–1.89 (3H, m, 3 × H-piperidine), 2.16 (3H, s, CH_3_), 2.72–2.80 (2H, m, 2 × H-piperidine), 4.11 (2H, d, *J* = 7.3 Hz, pyrazole-CH_2_-piperidine), 8.30 (1H, s, H-pyrazole), 8.93(1H, s, H-pyrazole); ^13^C NMR (125 MHz; DMSO-*d*_6_) δ_C_ 28.9 (2 × CH_2_-piperidine), 35.5 (CH-piperidine), 46.0 (CH_3_), 54.6 (2 × CH_2_-piperidine), 57.5 (N-*C*H_2_-CH), 130.8 (CH-pyrazole), 134.7 (C-pyrazole), 135.6 (CH-pyrazole); HRMS calcd for C_10_H_17_N_4_O_2_ [M + H]^+^ 225.1346, found 225.1341.

##### 1-((1-Methylpiperidin-4-yl)methyl)-1*H*-pyrazol-4-amine (**28e**)

Prepared according to general procedure D using nitropyrazole **27d** (190 mg, 0.85 mmol) and MeOH (20 mL) for 2 h to give a pale brown oil (150 mg, 91%); *R*_f_ 0.40 (NH_2_ SiO_2_, 7% MeOH/CH_2_Cl_2_); mp 50–55 °C; λ_max_ (EtOH)/nm 246; IR ν_max_/cm^–1^ 3304, 3127, 2919, 2794; ^1^H NMR (500 MHz; DMSO-*d*_6_) δ_H_ 1.18 (2H, qd, *J* = 12.5 and 3.7 Hz, 2 × H-piperidine), 1.39–1.47 (2H, m, 2 × H-piperidine), 1.61–1.72 (1H, m, H-piperidine), 1.74–1.84 (2H, m, 2 × H-piperidine), 2.15 (3H, s, CH_3_), 2.70–2.78 (2H, m, 2 × H-piperidine), 3.80 (2H, d, *J* = 7.3 Hz, N-C*H*_*2*_-CH), 6.91 (1H, s, H-pyrazole), 7.02 (1H, s, H-pyrazole); ^13^C NMR (125 MHz; DMSO-*d*_6_) δ_C_ 29.3 (2 × CH_2_-piperidine), 36.3 (2 × CH_2_-piperidine), 46.1 (CH_3_), 54.9 (2 × CH_2_-piperidine), 56.5 (N-*C*H_2_-CH), 116.9 (CH-pyrazole), 129.0 (CH-pyrazole), 130.5 (C-pyrazole); HRMS calcd for C_10_H_18_N_4_ [M + H]^+^ 195.1604, found 195.1600.

##### 4-(3,6-Dichloro-2-fluorobenzoyl)-*N*-(1-((1-methylpiperidin-4-yl)methyl)-1*H*-pyrazol-4-yl)-1*H*-pyrrole-2-carboxamide (**34k**)

Amine **28e** (140 mg, 0.43 mmol) was added to carboxylic acid **31b** (87 mg, 0.29 mmol), pyridine (23 μL, 0.29 mmol), and bromotripyrrolidinophosphonium hexafluorophosphate (PyBrOP) (200 mg, 0.43 mmol) in MeCN (2 mL). The mixture was stirred at RT for 1 h and then partitioned between EtOAc (2 × 30 mL) and H_2_O (20 mL). The organic layers were combined, washed with brine, dried (MgSO_4_), and the solvent was removed *in vacuo*. Purification by MPLC on NH_2_ SiO_2_ with gradient elution from 1 to 4% MeOH/CH_2_Cl_2_ gave a beige solid (53 mg, 39%); *R*_f_ 0.40 (NH_2_ SiO_2_, 7% MeOH/CH_2_Cl_2_); mp 124–128 °C; λ_max_ (EtOH)/nm 252; IR ν_max_/cm^–1^ 2935, 1639, 1592; ^1^H NMR (500 MHz; DMSO-*d*_6_) δ_H_ 1.23 (2H, qd, *J* = 3.0 and 12.5 Hz, 2 × H-piperidine), 1.41–1.51 (2H, m, 2 × H-piperidine), 1.70–1.85 (3H, m, 3 × H-piperidine), 2.15 (CH_3_), 2.72–2.80 (2H, m, 2 × H-piperidine), 4.00 (2H, d, *J* = 7.3 Hz, N-C*H*_2_-CH), 7.35 (1H, s, H-pyrrole), 7.53–7.61 (3H, m, H-4′, H-pyrrole and H-pyrazole), 7.82 (1H, app t, *J* = 8.4 Hz, H-3′), 7.97 (1H, s, H-pyrazole), 10.28 (1H, s, CO-NH), 12.52 (1H, br s, NH-pyrrole); ^13^C NMR (125 MHz; DMSO-*d*_6_) δ_C_ 29.2 (2 × C-piperidine), 36.2 (C-piperidine), 46.1 (CH_3_), 54.8 (2 × C-piperidine), 56.7 (N-*C*H_2_-CH), 110.5 (CH-pyrrole), 121.0 (d, *J*_CF_ = 29.7 Hz, C-3′), 121.3 (CH-pyrazole), 124.7 (C-pyrrole), 126.9 (d, *J*_CF_ = 4.1 Hz, C-5′), 128.2 (C-pyrrole), 128.7 (CH-pyrrole), 129.2 (d, *J*_CF_ = 21.8 Hz, C-1′), 129.2 (d, *J*_CF_ = 5.4 Hz, C-6′), 130.1 (C-1′), 130.5 (C-pyrazole), 131.8 (C-4′), 153.8 (d, *J*_CF_ = 248.2 Hz, C-2′), 156.8 (CO-NH), 182.6 (CO); ^19^F NMR (470 MHz; DMSO-*d*_6_) δ_F_ −116.7; HRMS calcd for C_22_H_23_^35^Cl_2_F_1_N_5_O_2_ [M + H]^+^ 478.1207, found 478.1195.

### Biological Assay Protocols

#### ERK5 IMAP Assay Protocol

##### Preparation of Assay Buffer (1×)

The 0.01% Tween-20 5× stock was supplied as part of the IMAP FP progressive binding system kit (Molecular Devices R7436) and was diluted to 1× using Milli-Q H_2_O. One microliter of a 1 M dithiothreitol (DTT) stock was added for every 1 mL of a 1× assay buffer to give a final concentration of 1 mM DTT. Preparation of ERK5 working solution. The final dilution was dependent on the activity of the enzyme batches. The initial batch (08/08/08) was used as a 1 in 1 in a 350 final dilution in assay buffer. A 1:175 dilution of ERK5 stock was performed in a 1× assay buffer. For 1 plate, 13 μL of ERK5 stock was added to 2262 μL of a 1× assay buffer. Aliquots were stored at −80 °C. Batch PO080808 was used at a stock concentration of 73.4 ng/μL.

##### Preparation of ATP/Substrate Working Solution

For one plate, ATP disodium salt (90 μL, 20 mM) (Sigma-Aldrich A7699) and FAM-EGFR-derived peptide (15 μL, 100 μM) (LVEPLTPSGEAPNQ(K-5FAM)-COOH) (Molecular Devices RP7129; reconstituted in Milli-Q H_2_O to a stock concentration of 100 μL; stored at −20°C) were added to 2295 μL of a 1× assay buffer.

##### Preparation of IMAP Binding Solution

For one plate, 20.5 μL of IMAP binding reagent stock, 1476 μL of 1× binding buffer A (60%), and 984 μL of binding buffer B (40%) [IMAP FP Progressive screening express kit (Molecular Devices R8127)] were added to 9819.5 μL of Milli-Q H_2_O.

##### Assay Procedure

One microliter of compound (in 60:40 H_2_O/DMSO) or 60:40 H_2_O/DMSO (for controls and blanks) was dry-spotted into the relevant wells of a 384-well assay plate using a MATRIX PlateMate Plus. Five microliters of ERK5 working solution was added to test and control wells, and 5 μL of a 1× assay buffer was added to blanks; 4 μL of ATP/substrate working solution was added to all wells using a Matrix multichannel pipette. The plate was sealed using DMSO-resistant 205 clear seal and incubated for 2 h at 37 °C. One microliter of the kinase reaction mixture from the first plate was dry-spotted into a second 384-well assay plate using the MATRIX PlateMate Plus. Nine microliters of assay buffer was added, followed by 30 μL of IMAP binding solution using a multichannel pipette. The plate was incubated at RT in darkness for 2 h. The assay plate was then read on an Analyst HT plate reader (Molecular Devices) using the settings described below; measurement mode = fluorescence polarization; method ID = ERK5; integration time = 100 ms; excitation filter = fluorescein 485–20; emission filter = 530–25; dichroic mirror = 505 nm; plate definition file = Corning 384 black fb; Z-height = 5.715 mm (middle); G-factor = 1; attenuator = out; detector counting = Smartread+; and sensitivity = 2.

#### p38α LANCE Assay Protocol

##### Preparation of Assay Buffer (1×)

A 1× assay buffer was prepared freshly from the following reagents: 250 mM tris(hydroxymethyl)aminomethane (Tris) pH 7.5, 25 mM MgCl_2_, 2.5 mM ethylene glycol tetraacetic acid (EGTA), 10 mM dithiothreitol (DTT), and 0.05% Triton X100 in Milli-Q H_2_O (NB: 1× buffer final assay concentrations were 5× lower than stated above).

##### Preparation of p38α/SAPK2 Working Solution

The p38α/SAPK2, active N-terminal GST-tagged recombinant full-length protein (Millipore 14–251) was supplied as a 10 μg/4 μL stock. This was diluted to a 10 μg/40 μL (1 μM) concentration by the addition of 156 μL of tris/HCl (pH 7.5, 50 mM), NaCl (150 mM), EGTA (0.1 mM), Brij-35 surfactant (0.03%), glycerol (50%), and 0.1% 2-mercaptoethanol (0.1%). The final dilution was dependent on the activity of the enzyme batches. The p38α concentration used in the assay was 1 nM. A 2× working stock solution (2 nM, 500-fold dilution of 1 μM stock) in a 1× assay buffer was prepared. For one plate, 9.4 μL of p38α (1 μM) was added to 1870.6 μL of Milli-Q H_2_O.

##### Preparation of ATP/Substrate Working Solution

For one plate, ATP disodium salt (17.5 μL, 200 mM stock) (Sigma-Aldrich A7699) and Ulight-MBP Peptide (50 μL, 5 μM stock) (Perkin Elmer TRF0109) were added to 400 μL of 5× assay buffer and 1532.5 μL of Milli-Q H_2_O.

##### Preparation of Ethylenediaminetetraacetic Acid (EDTA)/Antibody Detection Reagent

For one plate, 84 μL of ethylenediaminetetraacetic acid (EDTA) (0.5 M) (Sigma-Aldrich E4378-100G) and 27 μL of Europium-anti-phospho-MBP antibody (0.625 μM) (Perkin Elmer) were added to 420 μL of LANCE detection buffer (1×) and 3669 of Milli-Q H_2_O.

##### Assay Procedure

One microliter of compound (in 80:20 H_2_O/DMSO) or 80:20 H_2_O/DMSO was dry-spotted into the relevant wells of a 384-well assay plate using the MATRIX PlateMate Plus. Five microliters of p38α working solution was added to test and control wells, and 5 μL of assay buffer was added to blanks; 4 μL of the ATP/substrate working solution was added to all wells using a Thermo Multidrop Combi or Matrix multichannel pipette. The plate was sealed using DMSO-resistant clear seal and incubated for 1 h at 37 °C. Ten microliters of the EDTA/antibody working solution was added to all wells using a Thermo Multidrop Combi or Matrix multichannel pipette. The plate was incubated at RT in darkness for 2 h. The assay plate was then read on a PheraStar microplate reader using the settings described below; Pherastar: measurement mode = TRF; method ID = LANCE HTRF ERK5; optic module: 337, 665, 620 nm. Focal height = 6.0, positioning delay, 0.1 s, number of flashes per well = 100, integration start = 60.

### Cell Growth Inhibition Assays

Human cell lines were obtained from the American Type Culture Collection (ATCC) and maintained at 37 °C in 5% CO_2_ with 95% humidity. Cells were cultured in RPMI-1640 medium containing 2 mM l-glutamine and 10% (v/v) fetal bovine serum (Life Technologies). Cell lines were authenticated by short tandem repeat profiling (LGC Standards) and routinely tested for mycoplasma contamination at 3 monthly intervals. Cell proliferation was assessed using a previously described sulforhodamine B (SRB) assay^51^ following a 72 h incubation with compound.

### Western Blot Densitometry Cell-Based Assay in Hela Cells

#### Protocol

HeLa cells were serum-starved overnight followed by treatment with ERK5 inhibitors for 1 h. Cells were then stimulated with 100 ng/mL EGF for 10 min. The cells were harvested and lysed at 4 °C for 5–10 min in Laemmli buffer containing Halt protease and phosphate inhibitors (Pierce). The lysates were boiled for 10 min at 100 °C. A 20 μm sample was run on a 6% tris–glycine gel and transferred to nitrocellulose. Western blotting was done with ERK5 antibody (Cell Signaling #3372S). The IC_50_ was calculated from densitometry of the top (phospho-ERK) bands. Values represent single determinations or the mean ± standard deviation (SD) (*n* = 3–5).

#### BRD4 Expression, Purification, and Surface Plasmon Resonance Protocols

##### Expression and Purification of Recombinant BRD4 Bromodomain 1

Harvested bacterial cells were resuspended in lysis buffer comprising 50 mM HEPES (pH 7.4), 200 mM NaCl, 10 mM imidazole, 0.5 mg mL^–1^ lysozyme, and 0.2 mg mL^–1^ DNAse at 4 °C for 1 h. After sonication and centrifugation (1 h at 35,000*g*), the supernatant was purified by immobilized Ni^2+^ ion affinity chromatography. The peak fractions were pooled and incubated with GST-tagged HRV 3C protease (50:1) at 4 °C overnight. The cleaved His-tag was separated from BRD4 by size exclusion chromatography using a Superdex 75 (26/60) column (GE Healthcare), equilibrated, and run in 50 mM HEPES (pH 7.4), 200 mM NaCl, and 1 mM DTT. All purification steps were performed using an ÄKTA Pure (GE Healthcare) at 4 °C.

##### Surface Plasmon Resonance

SPR-based ligand binding assays were performed using a BIAcore S200 (GE Healthcare) at 25 °C using single cycle affinity. Immobilization of BRD4 was achieved using standard amine coupling on a CM5 chip surface. The surface was prepared through activation with 1-ethyl-3-(3-dimethylaminopropyl)carbodiimide/*N*-hydroxy succinimide (EDC/NHS), followed by injection of 10 μg mL^–1^ BRD4 until a target level of 8000 RU was reached. The surface was then quenched using 1 M ethanolamine and washed with running buffer (10 mM HEPES, 150 mM NaCl, 0.01% (v/v) Tween-20, 0.5 mM TCEP, and 1% (v/v) DMSO) at a flow rate of 30 μL min^–1^. XMD8-92 and 46 were injected in a dose–response manner (nine points ranging from 0 to 20 μM) with a contact time of 30 s and a dissociation time of 160 s in series across the reference and BRD4-immobilized flow cells using solvent correction to account for bulk refractive index changes. The reference channel response was subtracted from the BRD4-immobilized channel response, and dose–response data were fitted using an affinity steady-state 1:1 binding model to determine the *K*_d_.

#### ERK5-Dependent Cellular Reporter Assay

To examine the inhibition of ERK5 kinase and transcriptional activity in cells, a previously described ERK5:MEF2D reporter assay was used.^21^ Using Lipofectamine 2000 (ThermoFisher Scientific), HEK293 cells in 96-well plates were transfected with a constitutively active form of MEK5 (pEGFR–MEK5D), HA-tagged ERK5 (either full-length or a.a. 1–492, which lacked the NLS and C-terminal TAD), a GAL4-activated DNA-binding domain fused to the ERK5 substrate MEF2D (rat, a.a. 87–428), a 5XGAL4–luciferase reporter construct, and a CMV–renilla luciferase reporter construct. Compound **34b** was added 4 h after transfection, and cells were incubated (37 °C, 5% CO_2_) for a further 20 h, prior to the determination of firefly and renilla luciferase using a dual luciferase reporter assay kit (Promega). The firefly luciferase activity was normalized to the renilla luciferase signal to quantify the ERK5-driven transcriptional activity.

### Crystallographic Protocols

#### Preparation of ERK5–**34b** Complex Crystals

The purified unphosphorylated ERK5 kinase domain (residues 46–402) was purchased from Proteros Biostructures GmbH, and cocrystals with compound were prepared in a similar manner as described.^52^ ERK5 (46–402) at 11.5 mg mL^–1^ in storage buffer (50 mM HEPES (pH 6.5), 150 mM NaCl, 10% (v/v) glycerol, 2 mM DTT) was mixed with **34b** (100 mM in 100% DMSO) to give a final concentration of 1 mM **34b** and 1% (v/v) DMSO. Complex formation was allowed to proceed for 2 h on ice. The sample was then clarified by centrifugation (5 min, 16,000*g*, 4 °C) immediately before use in crystallization. Crystals were grown by sitting drop vapor diffusion at 20 °C in 96-well MRC plates by mixing the protein: compound complex with crystallization buffer comprising 5% (v/v) PEG 6000, 0.1 M 2-(*N*-morpholino)ethanesulfonic acid (MES) (pH 6.0), 5 mM DTT in a 1:1 ratio to give a 0.8 μL drop. Drops were immediately streak-seeded with a seed stock prepared from crystals of ERK5 with an indazole ERK5 inhibitor (in house series—unpublished structure). The seed stock was prepared by looping two crystals of the ERK5: indazole complex into a 2 μL crystallization buffer [5% (v/v) PEG 6000, 0.1 M MES (pH 6.0), 5 mM DTT]. The buffer and crystals were transferred into a microcentrifuge tube containing a stabilization buffer [20 μL 5% (v/v) PEG 6000, 0.1 M MES (pH 6.0), 5 mM DTT plus 80 μL 6% (v/v) PEG 6000, 0.1 M MES (pH 6.25), 5 mM DTT], and the crystals were crushed by vortexing with a Teflon bead. The seed stock was aliquoted into cryotubes, flash-frozen in liquid nitrogen, and stored at −80 °C until use.

#### X-ray Diffraction Data Collection, Structure Solution, and Refinement for the Complex of ERK5 with **34b**

Crystals were passed briefly through a cryoprotectant solution comprising 4.9% (v/v) PEG 6000, 70 mM MES (pH 6.0), 3.5 mM DTT, 30% (v/v) glycerol, 1% (v/v) DMSO, and 10 mM **34b** before flash cooling in liquid nitrogen. Data were collected at 100K on beamline I04 at the DIAMOND Light Source (Oxford, U.K.). Data processing was carried out using XDS, POINTLESS/AIMLESS (PMID: 21460446), and other programs of the CCP4i suite (PMID: 15299374) run through the CCP4i2 gui. Structures were solved by molecular replacement using PHASER (PMID: 19461840) and pdb 5BYZ as a starting model. REFMAC (PMID: 15299926) was employed for refinement, and model building was performed using COOT (PMID: 20383002). PDB was deposited within the protein database www.pdb.org using accession code: 7PUS. The authors will release the atomic coordinates upon article publication.

*In vitro* pharmacokinetic profiling was performed at Cyprotex. Assay protocols can be found at https://www.cyprotex.com/admepk.

### ***In Vivo*** Pharmacokinetic Methods

Mice were treated intravenously with 10 mg/kg of compound in a vehicle of 10% *N*-methyl pyrrolidone (NMP) in saline. Blood samples were collected via the tail vein at 15, 30, and 60 min and by cardiac puncture under terminal anesthesia at 120, 240, and 360 min (nine mice in total; three per time point with serial sampling). Oral pharmacokinetic studies were performed in an analogous manner following the administration of a 10 mg/kg compound by oral gavage in the same vehicle. Drug levels were determined by liquid chromatography–mass spectrometry (LC–MS) analysis against a standard curve prepared in control plasma. All *in vivo* experiments were reviewed and approved by institutional animal welfare committees.

## Supporting Information Available

Additional figures of the spectroscopic characterization of all new compounds (PDF)
Crystallographic data of **34b** bound to ERK5 (PDB)
Molecular formula strings (CSV)
PDB file of **34b** bound to ERK5 (PDF)

## Supplementary Material

jm1c01756_si_001.pdf

jm1c01756_si_002.pdb

## Accession Codes

PDB was deposited within the protein database at www.pdb.org using accession code: 7PUS. The authors will release the atomic coordinates upon article publication.

## Abbreviations Used

ERK5: extracellular regulated kinase 5
ER: efflux ratio
MLM: mouse liver microsomes
MPLC: medium-pressure liquid chromatography
LC–MS: liquid chromatography–mass spectrometry
IC_50_: half-maximal inhibitory concentration
