# Supplementary material for: Parallel Optimization of Potency and Pharmacokinetics Leading to the Discovery of a Pyrrole Carboxamide ERK5 Kinase Domain Inhibitor
Source: J Med Chem. 2022 Apr 25;65(9):6513–40. doi: 10.1021/acs.jmedchem.1c01756 (PMC9109144; doi:10.1021/acs.jmedchem.1c01756)
Supplement: Supplementary file 1 — jm1c01756_si_001.pdf [file jm1c01756_si_001.pdf]

Supporting Information: Parallel Optimisation of Potency and Pharmacokinetics Leading to the Discovery of a Pyrrole Carboxamide ERK5 Kinase Domain Inhibitor

*Duncan C. Miller,[a‡] Tristan Reuillon,[a‡] Lauren Molyneux,[a] Timothy Blackburn,[a] Simon J. Cook,[c] Noel Edwards,[b] Jane A. Endicott,[b] Bernard T. Golding,[a] Roger J. Griffin,[a✝] Ian Hardcastle,[a] Suzannah J. Harnor,[a] Amy Heptinstall,[a] Pamela Lochhead,[c] Mathew P. Martin,[b] Nick C. Martin,[a] Stephanie Myers,[a] David R. Newell,[b] Richard A. Noble,[b] Nicole Phillips, Laurent Rigoreau,[d] Huw Thomas,[b] Julie A. Tucker,[b] Lan-Zhen Wang,[b] Michael J. Waring,[a] Ai-Ching Wong,[e] Stephen R. Wedge,[b] Martin E. M. Noble[b] and Celine Cano[a]**

a. Cancer Research UK Newcastle Drug Discovery Unit, Newcastle University Centre for Cancer, School of Natural and Environmental Sciences, Bedson Building, Newcastle University, Newcastle upon Tyne, NE1 7RU, UK.

b. Cancer Research UK Newcastle Drug Discovery Unit, Newcastle University Centre for Cancer, Paul O'Gorman Building, Medical School, Framlington Place, Newcastle upon Tyne, NE2 4HH, UK.

c. Signalling Laboratory, The Babraham Institute, Babraham Research Campus, Cambridge, CB22 3AT, UK

d. Cancer Research UK Therapeutic Discovery Laboratories, Jonas Webb Building, Babraham Campus, Babraham, Cambridgeshire CB22 3AT, UK

e. Cancer Research UK Therapeutic Discovery Laboratories, London Bioscience Innovation Centre, 2 Royal College Street, London NW1 0NH, UK.

[‡] These authors contributed equally to the work.

† Corresponding author: C.C. Tel (44) 191 208 7060. E-mail: celine.cano@ncl.ac.uk.

[✝]Deceased 24 September 2014

Table of Contents

Figure S1: ^{1}H NMR spectrum of compound **32a** S-4
Figure S2: HPLC purity assessment of compound **32a** S-5
Figure S3: ^{1}H NMR spectrum of compound **32b** S-6
Figure S4: HPLC purity assessment of compound **32b** S-7
Figure S5: ^{1}H NMR spectrum of compound **32c** S-8
Figure S6: ^{13}C NMR spectrum of compound **32c** S-9
Figure S7: ^{1}H NMR spectrum of compound **32d** S-10
Figure S8: ^{13}C NMR spectrum of compound **32d** S-11
Figure S9: ^{1}H NMR spectrum of compound **32e** S-12
Figure S10: ^{13}C NMR spectrum of compound **32e** S-13
Figure S11: ^{1}H NMR spectrum of compound **32f** S-14
Figure S12: ^{13}C NMR spectrum of compound **32f** S-15
Figure S13: ^{1}H NMR spectrum of compound **32g** S-16
Figure S14: ^{13}C NMR spectrum of compound **32g** S-17
Figure S15: ^{1}H NMR spectrum of compound **32i** S-18
Figure S16: ^{1}H NMR spectrum of compound **32k** S-19
Figure S17: HPLC purity assessment of compound **32k** S-20
Figure S18: ^{1}H NMR spectrum of compound **32l** S-21
Figure S19: ^{1}H NMR spectrum of compound **32m** S-22
Figure S20: ^{1}H NMR spectrum of compound **33f** S-23
Figure S21: ^{13}C NMR spectrum of compound **33f** S-24
Figure S22: HPLC purity assessment of compound **33f** S-25
Figure S23: ^{1}H NMR spectrum of compound **33g** S-26
Figure S24: ^{1}H NMR spectrum of compound **33h** S-27
Figure S25: ^{1}H NMR spectrum of compound **33i** S-28
Figure S26: ^{13}C NMR spectrum of compound **33i** S-29
Figure S27: ^{1}H NMR spectrum of compound **33j** S-30
Figure S28: ^{13}C NMR spectrum of compound **33j** S-31
Figure S29: HPLC purity assessment of compound **33j** S-32
Figure S30: ^{1}H NMR spectrum of compound **33k** S-33
Figure S31: ^{13}C NMR spectrum of compound **33k** S-34
Figure S32: HPLC purity assessment of compound **33k** S-35
Figure S33: ^{1}H NMR spectrum of compound **34a** S-36

Figure S34: ^{1}H NMR spectrum of compound **34b** S-37
Figure S35: ^{13}C NMR spectrum of compound **34b** S-38
Figure S36: HPLC purity assessment of compound **34b** S-39
Figure S37: ^{1}H NMR spectrum of compound **34c** S-40
Figure S38: ^{1}H NMR spectrum of compound **34d** S-41
Figure S39: ^{13}C NMR spectrum of compound **34d** S-42
Figure S40: ^{1}H NMR spectrum of compound **34e** S-43
Figure S41: ^{13}C NMR spectrum of compound **34e** S-44
Figure S42: ^{1}H NMR spectrum of compound **34h** S-45
Figure S43: ^{13}C NMR spectrum of compound **34h** S-46
Figure S44: HPLC purity assessment of compound **34b** S-47
Figure S45: ^{1}H NMR spectrum of compound **34i** S-48
Figure S46: ^{13}C NMR spectrum of compound **34i** S-49
Figure S47: ^{1}H NMR spectrum of compound **34j** S-50
Figure S48: ^{13}C NMR spectrum of compound **34j** S-51
Figure S49: ^{1}H NMR spectrum of compound **34k** S-52
Table S1: Kinome Selectivity Data for Compound **34b** S-53
Table S2: Kinome Selectivity (Kd) Data for Compound **34b** S-62
Figure S50: Cellular dependency on MAPK7 (RNAi gene silencing, DepMap) S-63
Figure S51: Quantification of p-ERK5 by Western blotting and densitometry S-64

Figure S1: ^{1}H NMR spectrum of compound **32a**

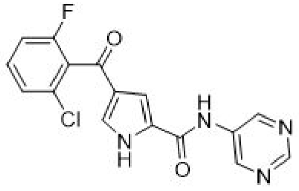

32a

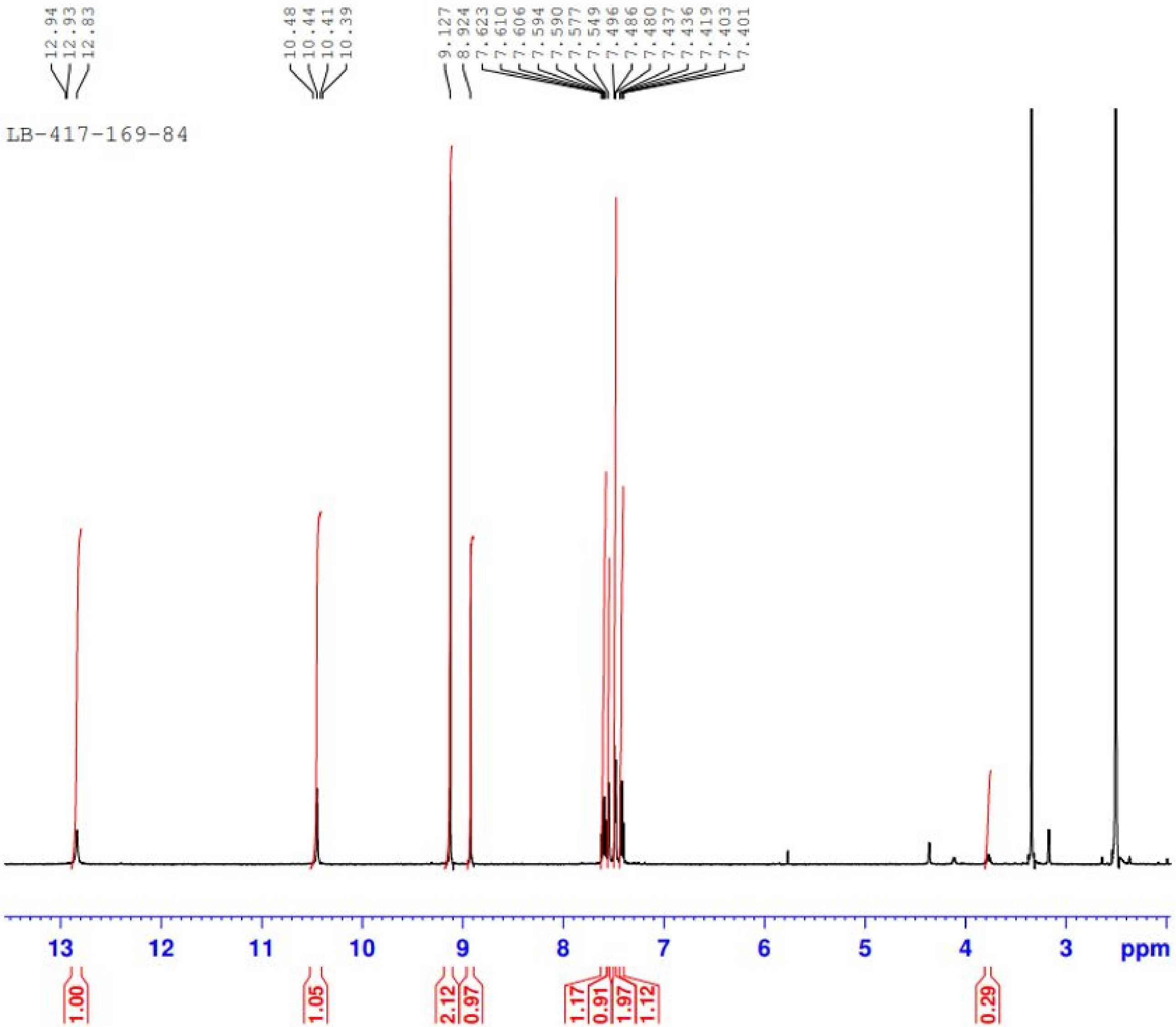

Figure S2: HPLC purity assessment of compound **32a**

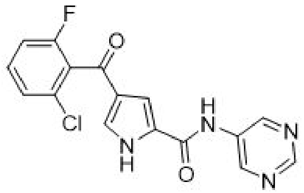

32a

Data File F:\07FEB12_03.D
Sample Name: LB/417/169/84

===

Acq. Operator : Karen Haggerty Seq. Line : 3
Acq. Instrument : Instrument 1 Location : Vial 1
Injection Date : 2/7/2012 8:09:09 AM Inj : 1
Inj Volume : 5.000 µl
Acq. Method : C:\CHEM32\1\DATA\FEB2012\07FEB12_LB 2012-02-07 07-26-28\XSELECT_ACIDIC.M
Last changed : 1/31/2012 4:49:10 PM by Karen Haggerty
Analysis Method : F:\07FEB12_03.D\DA.M (XSELECT_ACIDIC.M)
Last changed : 2/7/2012 10:50:37 AM by Karen Haggerty
(modified after loading)
Method Info : Waters XSELECT CSH C18 3.5um 100 x 4.6mm
0.1% Formic Acid (aq)/MeCN, 1.0mL/min

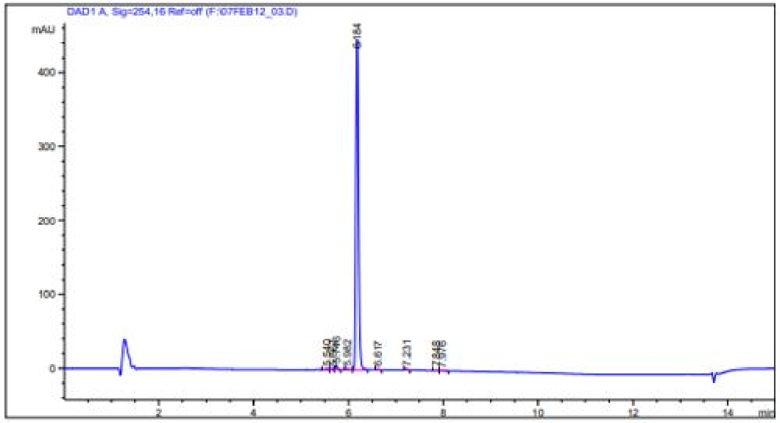

===
Area Percent Report
===

Sorted By : Signal
Multiplier: : 1.0000
Dilution: : 1.0000
Use Multiplier & Dilution Factor with ISTDs

Signal 1: DAD1 A, Sig=254,16 Ref=off

Peak #	RetTime [min]	Type	Width [min]	Area [mAU*s]	Height [mAU]	Area %
1	5.540	MF	0.0684	5.03878	1.22813	0.3134
2	5.667	MF	0.0583	4.54787	1.30081	0.2828
3	5.746	FM	0.0591	19.93161	5.61810	1.2396
4	5.982	FM	0.0763	5.52756	1.20686	0.3438
5	6.184	FM	0.0576	1555.92200	450.07758	96.7658

Sample Name: LB/417/169/84

Peak #	RetTime [min]	Type	Width [min]	Area [mAU*s]	Height [mAU]	Area %
6	6.617	FM	0.0702	3.74173	8.88406e-1	0.2327
7	7.231	FM	0.0667	6.40444	1.60114	0.3983
8	7.848	FM	0.0604	3.09037	8.52760e-1	0.1922
9	7.976	FM	0.0867	3.72125	7.15505e-1	0.2314

Totals : 1607.92562 463.48928

===
*** End of Report ***

Figure S3: ^{1}H NMR spectrum of compound **32b**

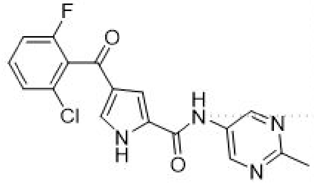

32b

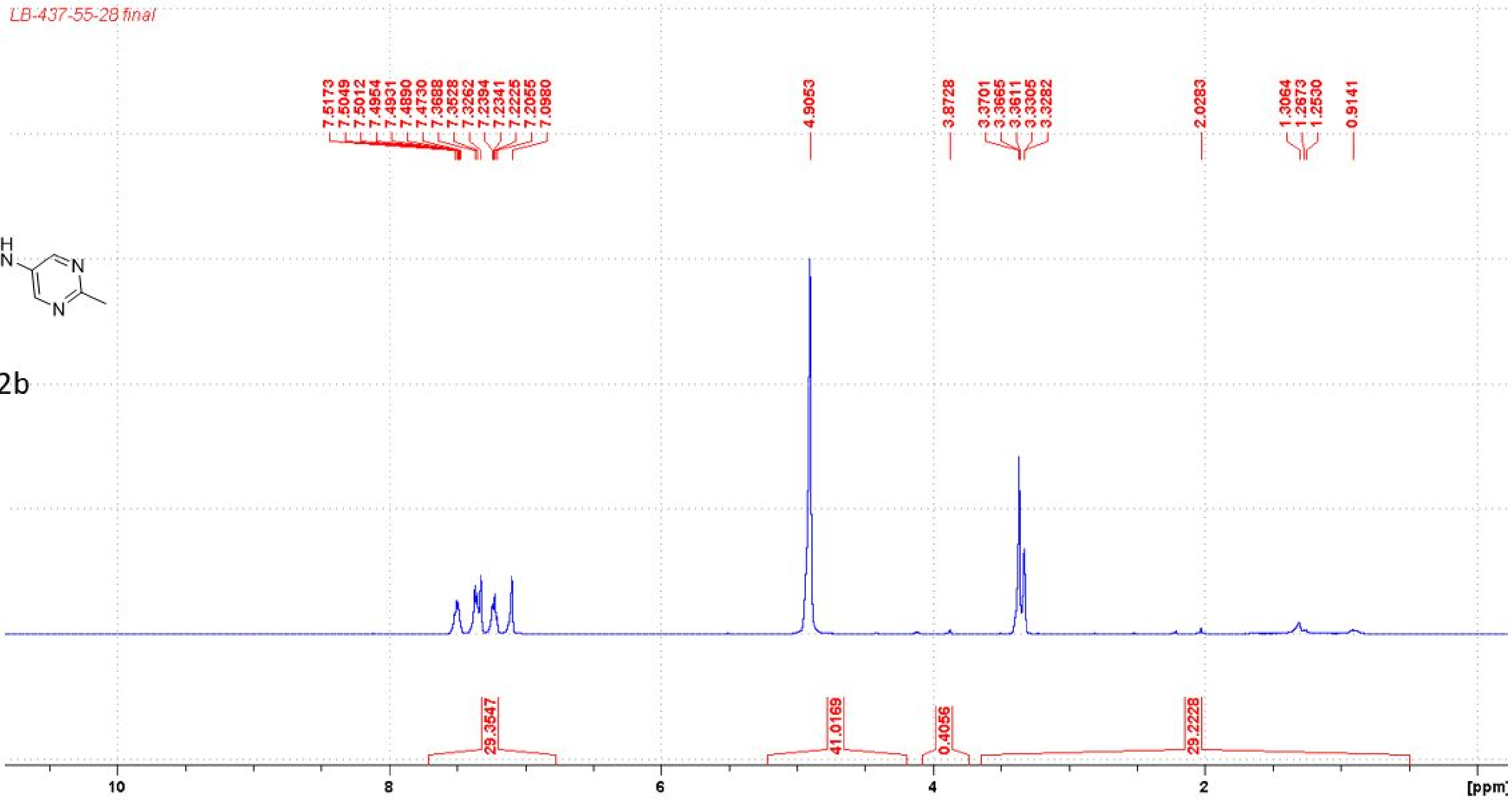

Figure S4: HPLC purity assessment of compound **32b**

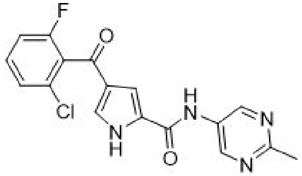

32b

```
Data File F:\23MAY12_11.D
Sample Name: LB/437/55/28

=====================================================================
Acq. Operator   : Karen Haggerty                  Seq. Line :    7
Acq. Instrument : Instrument 1                     Location : Vial 65
Injection Date  : 5/23/2012 11:35:43 AM                  Inj :    1
                                                Inj Volume : 5.000 µl
Different Inj Volume from Sequence !     Actual Inj Volume : 10.000 µl
Acq. Method     : C:\CHEM32\1\DATA\MAY2012\23MAY12_SMM_LB 2012-05-23 08-40-14\XSELECT_ACIDIC.
                  M
Last changed    : 5/23/2012 8:40:21 AM by Karen Haggerty
Analysis Method : F:\23MAY12_11.D\DA.M (XSELECT_ACIDIC.M)
Last changed    : 5/23/2012 2:00:11 PM by Karen Haggerty
                  (modified after loading)
Method Info     : Waters XSELECT CSH C18 3.5um 100 x 4.6mm
                  0.1% Formic Acid (aq)/MeCN, 1.0mL/min
```

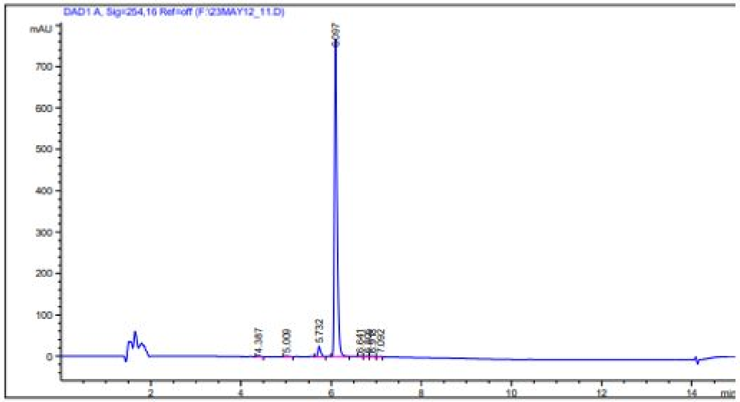


```
=====================================================================
                        Area Percent Report
=====================================================================

Sorted By             :      Signal
Multiplier:                  :      1.0000
Dilution:                    :      1.0000
Use Multiplier & Dilution Factor with ISTDs

Signal 1: DAD1 A, Sig=254,16 Ref=off
```

Sample Name: LB/437/55/28

Peak #	RetTime [min]	Type	Width [min]	Area [mAU*s]	Height [mAU]	Area %
1	4.387	BB	0.0566	10.54312	2.88661	0.3109
2	5.009	BB	0.0692	7.75120	1.63698	0.2286
3	5.732	FM	0.0697	102.63313	24.52467	3.0265
4	6.097	FM	0.0699	3236.33252	772.19928	95.4348
5	6.641	FM	0.0832	7.20853	1.44415	0.2126
6	6.805	FM	0.1067	9.30931	1.45399	0.2745
7	6.918	FM	0.1028	9.30425	1.50831	0.2744
8	7.092	FM	0.0889	8.06266	1.51183	0.2378
Totals :				3391.14472	807.16584	

```
=====================================================================
                      *** End of Report ***
```

Figure S5: ^{1}H NMR spectrum of compound **32c**

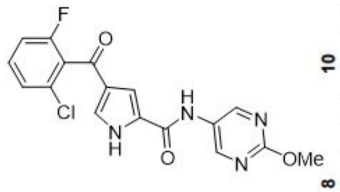

32c

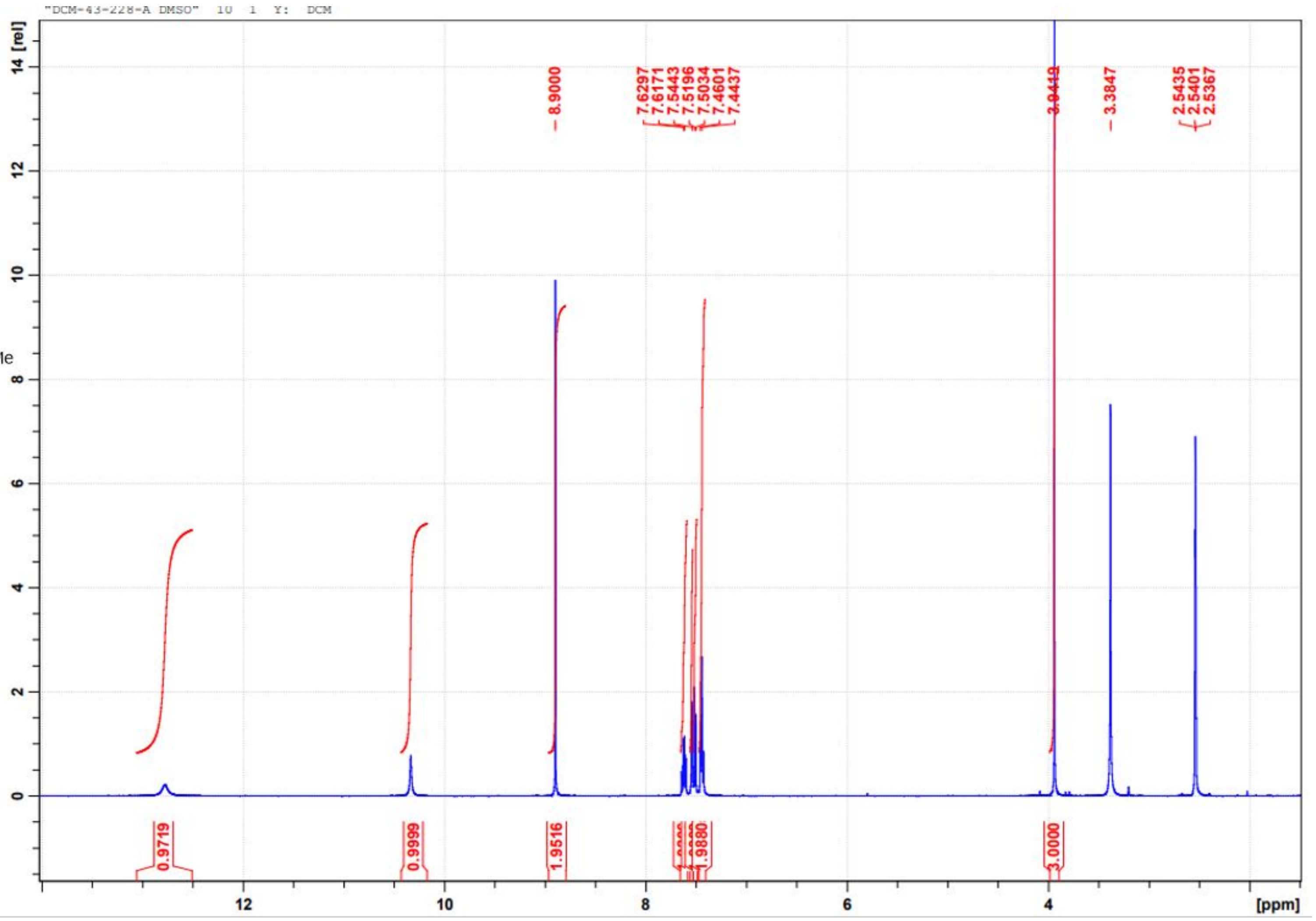

Figure S6: ^{13}C NMR spectrum of compound **32c**

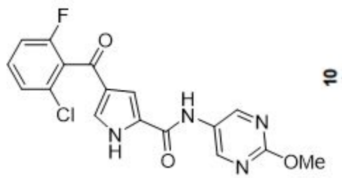

32c

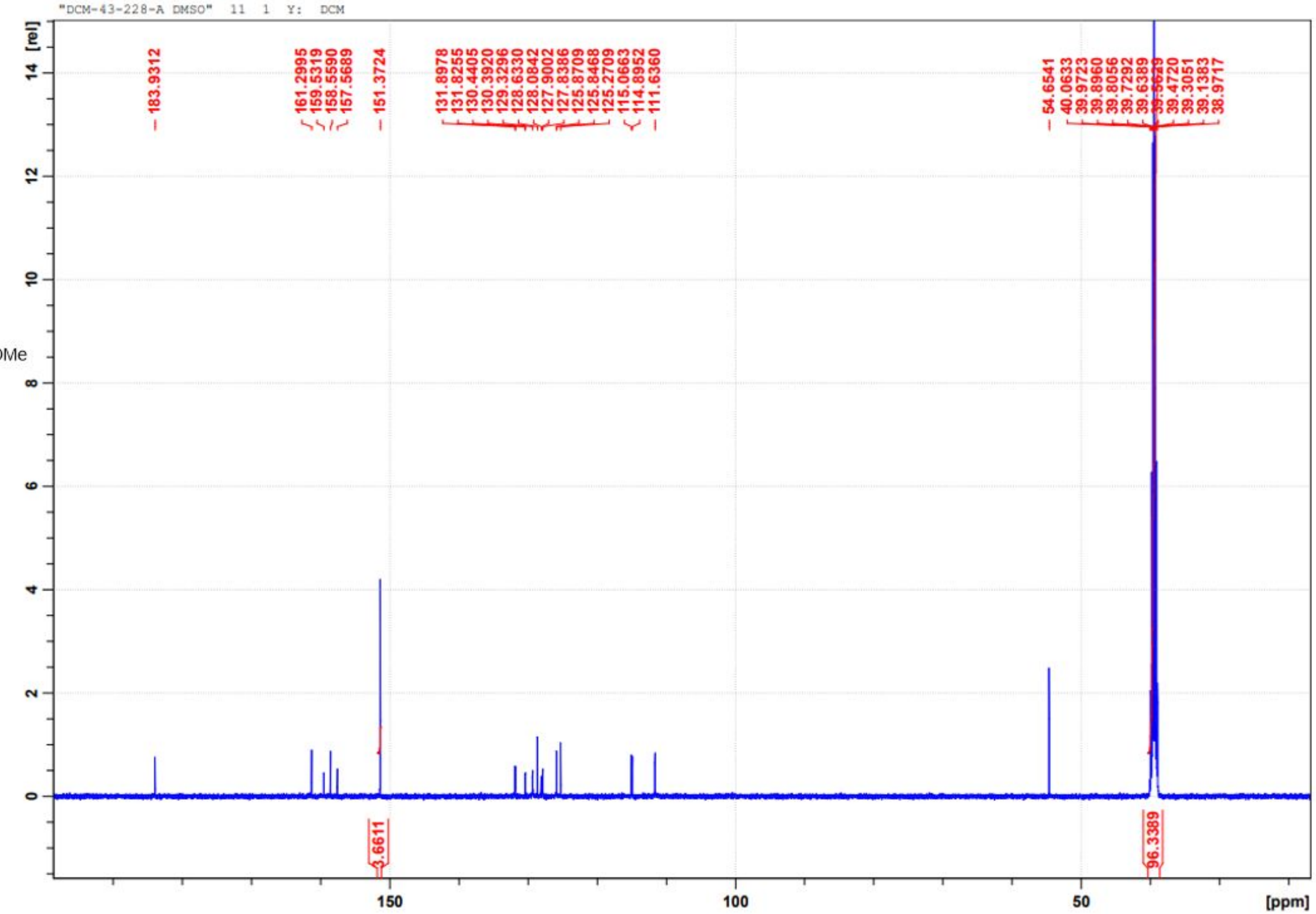

Figure S7: ^1H NMR spectrum of compound **32d**

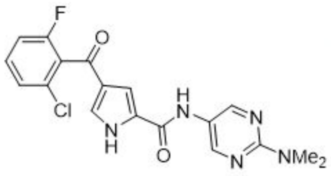

32d

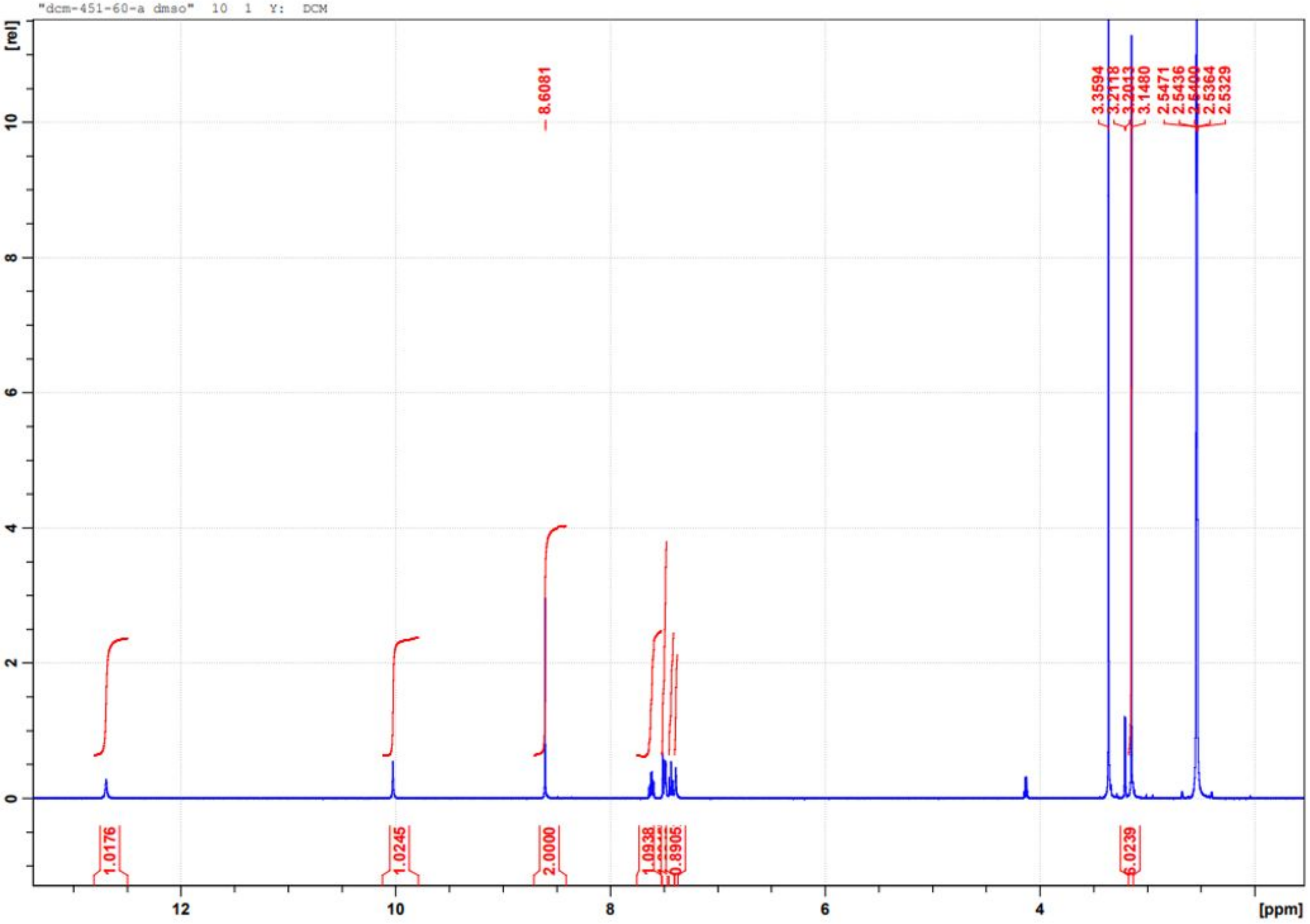

Figure S8: ^{13}C NMR spectrum of compound **32d**

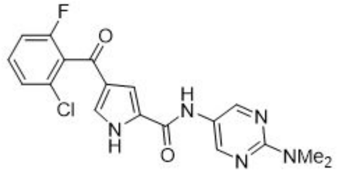

32d

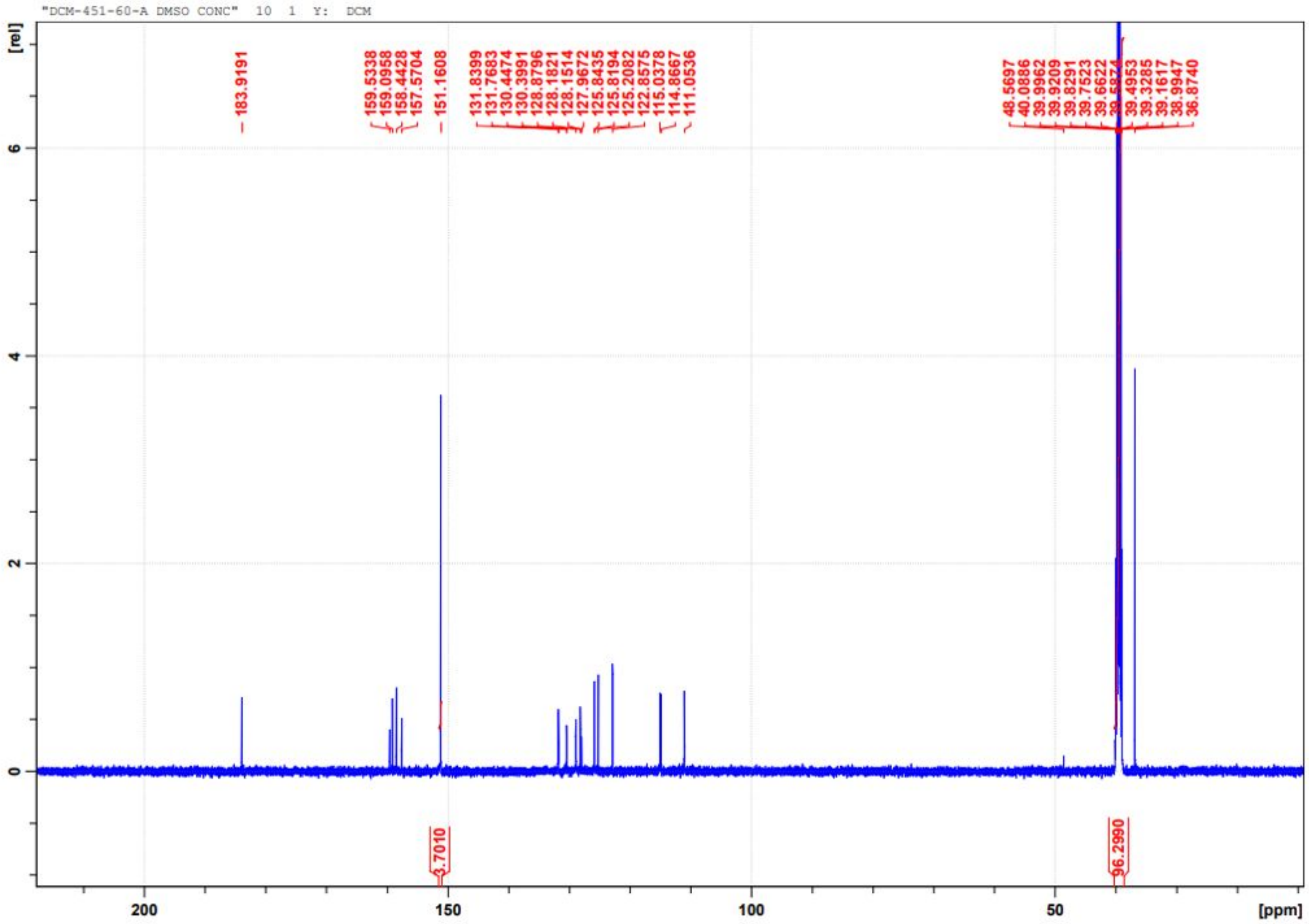

Figure S9: ^{1}H NMR spectrum of compound **32e**

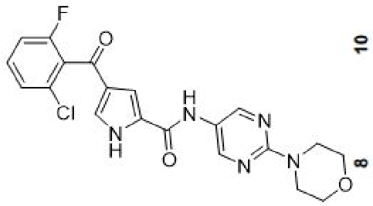

32e

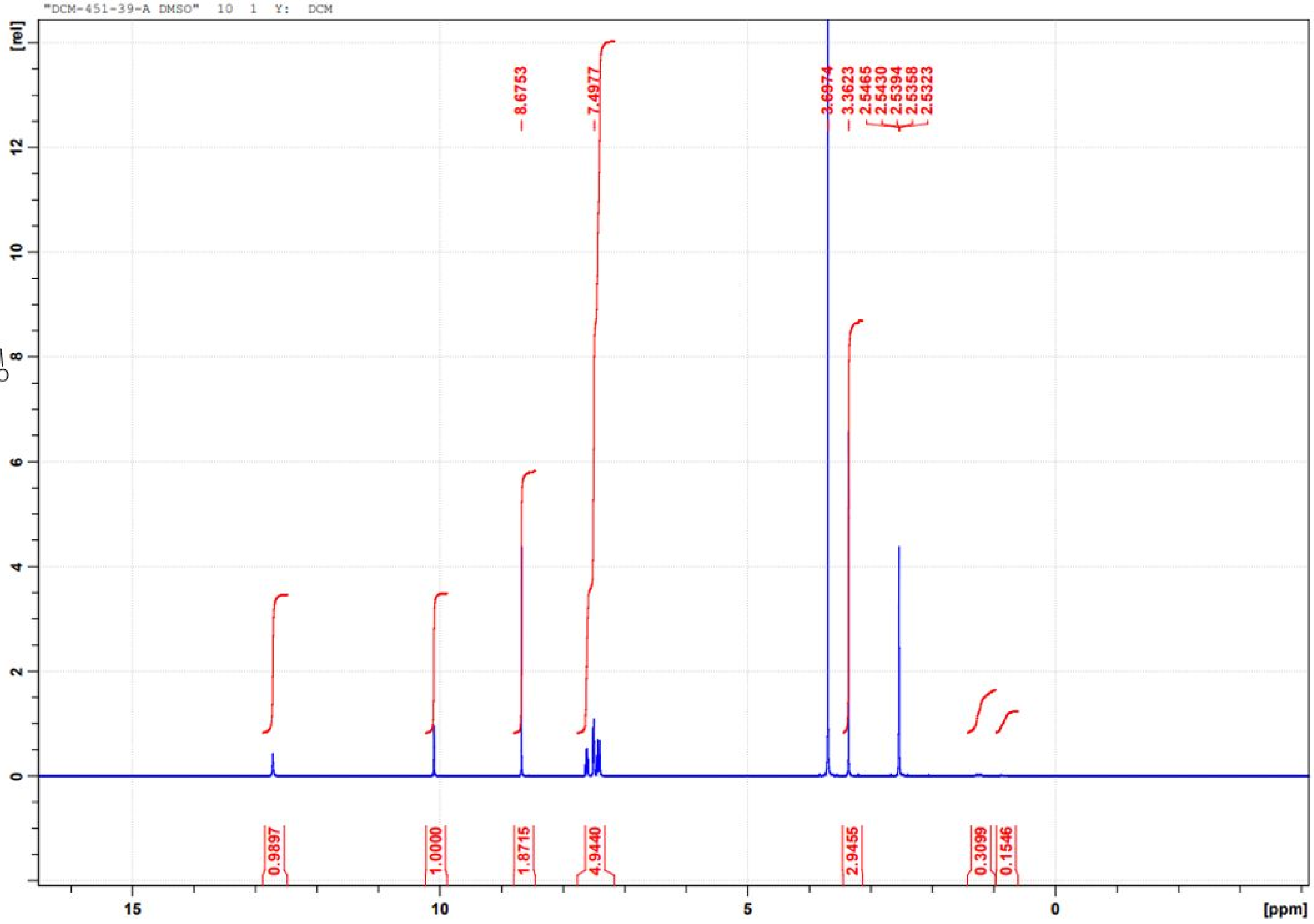

Figure S10: ^{13}C NMR spectrum of compound **32e**

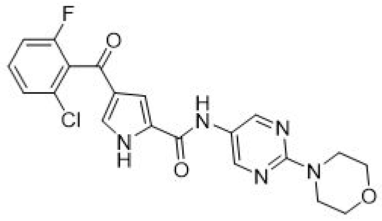

32e

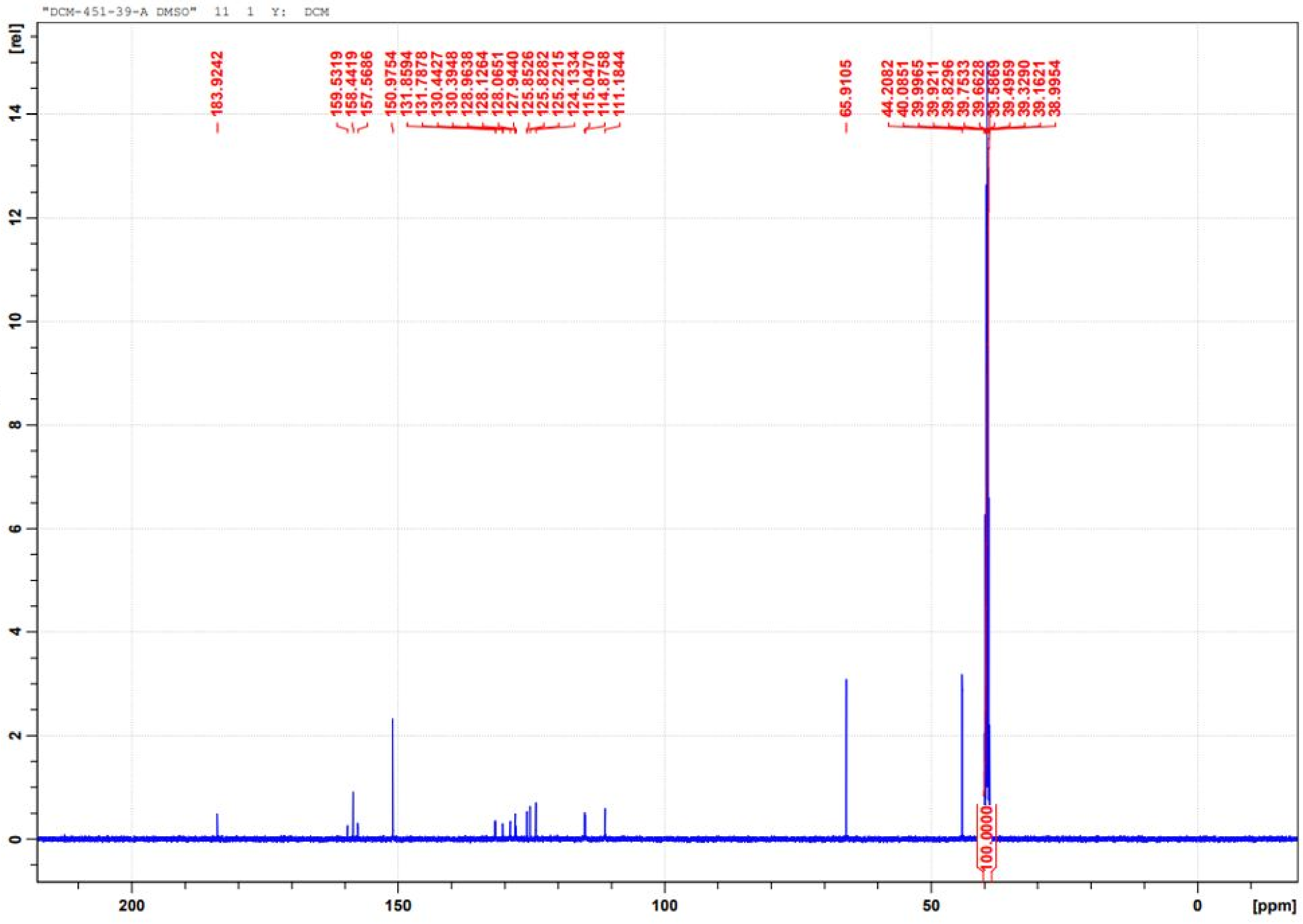

Figure S11: ^{1}H NMR spectrum of compound **32f**

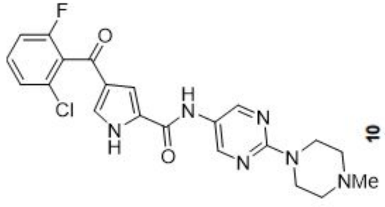

32f

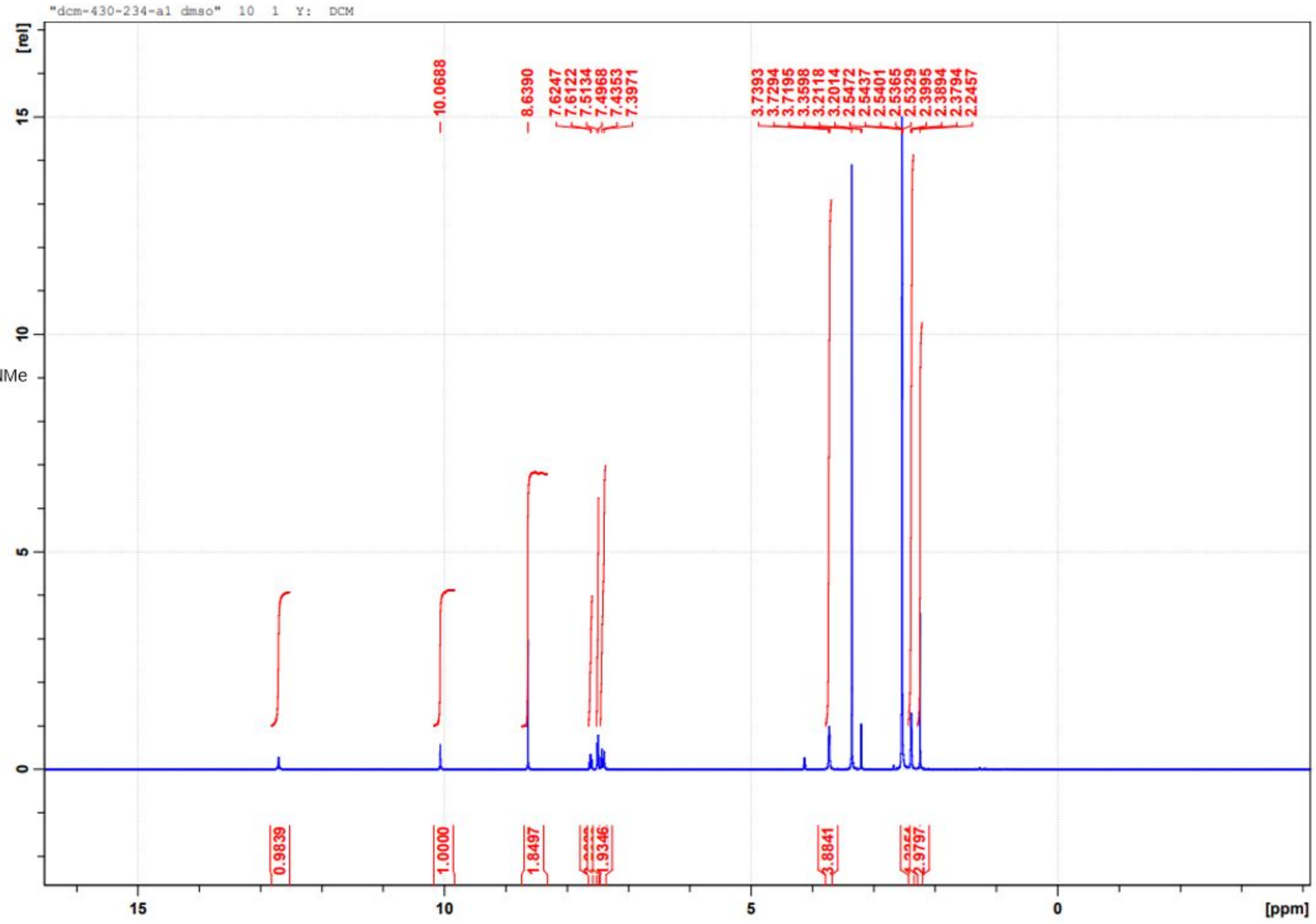

Figure S12: ^{13}C NMR spectrum of compound **32f**

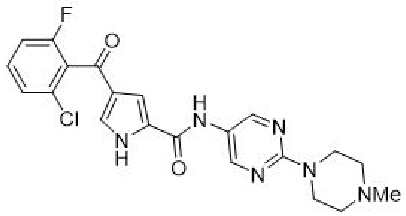

32f

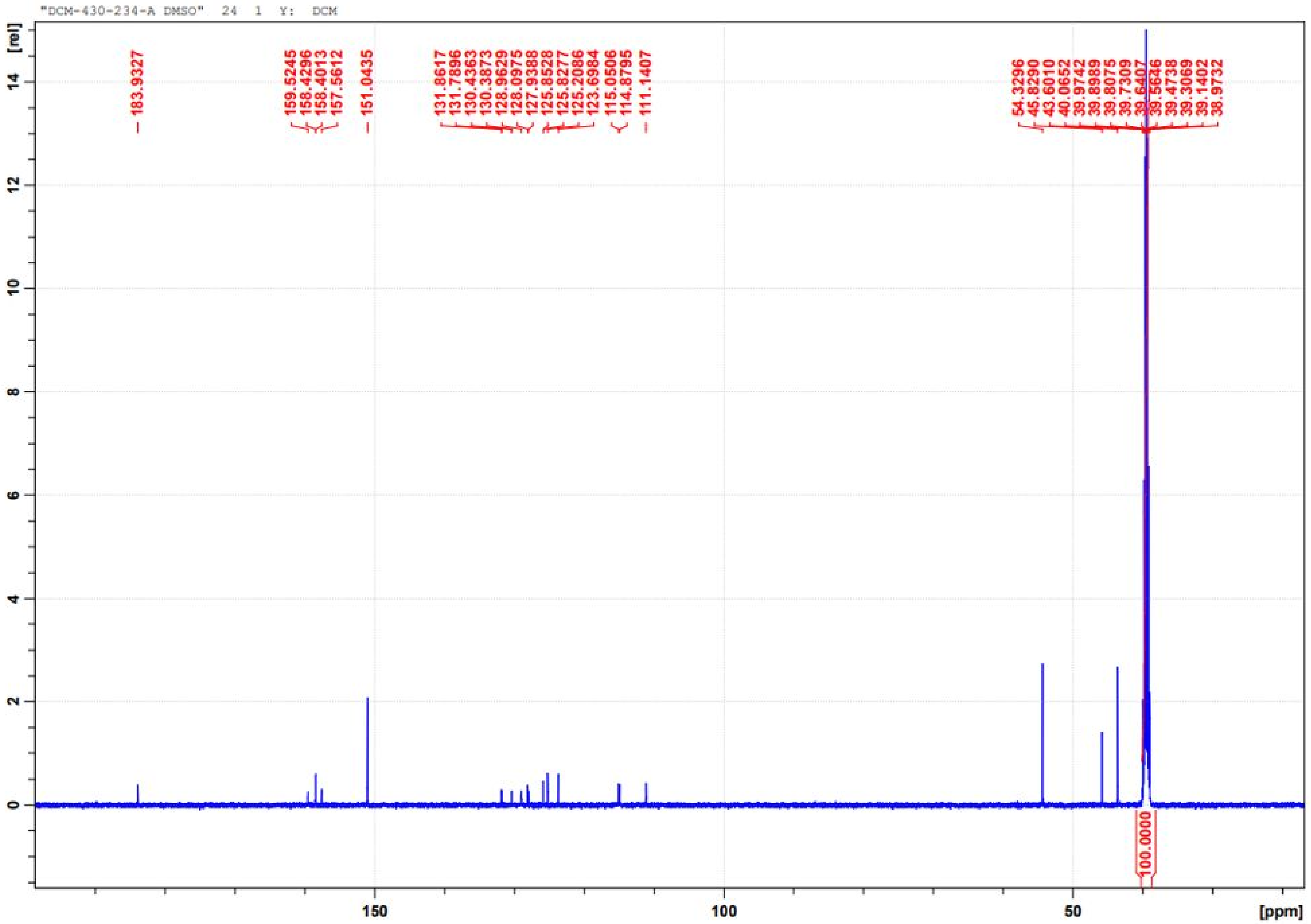

Figure S13: ^{1}H NMR spectrum of compound **32g**

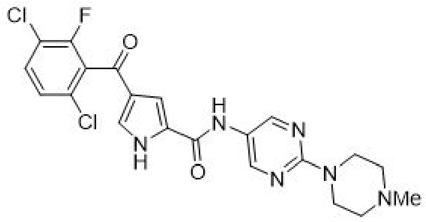

32g

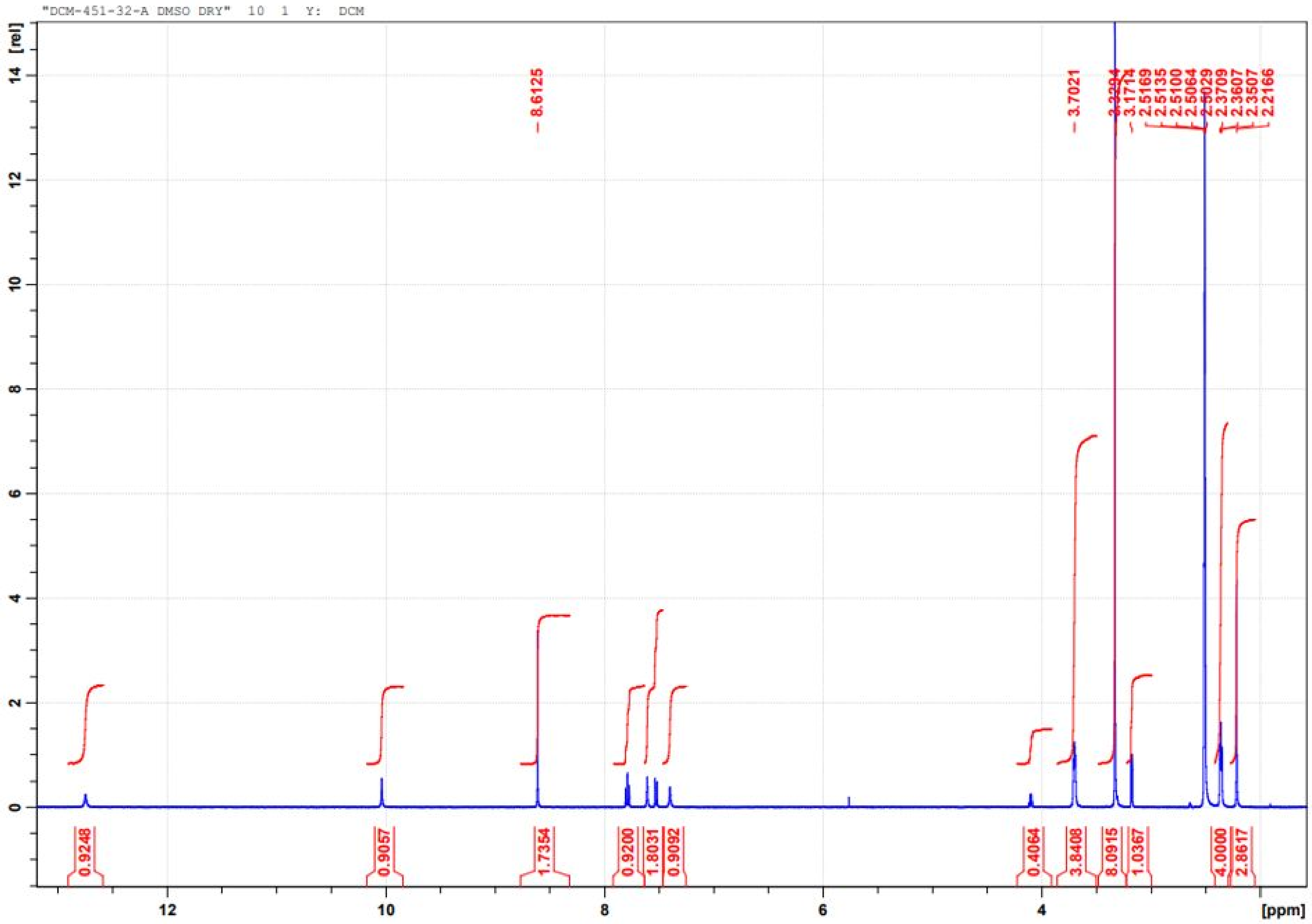

Figure S14: ^{13}C NMR spectrum of compound **32g**

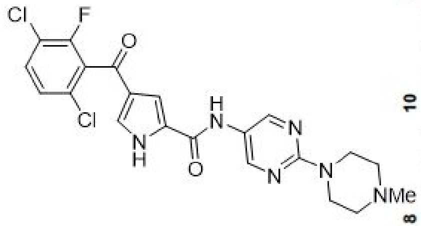

32g

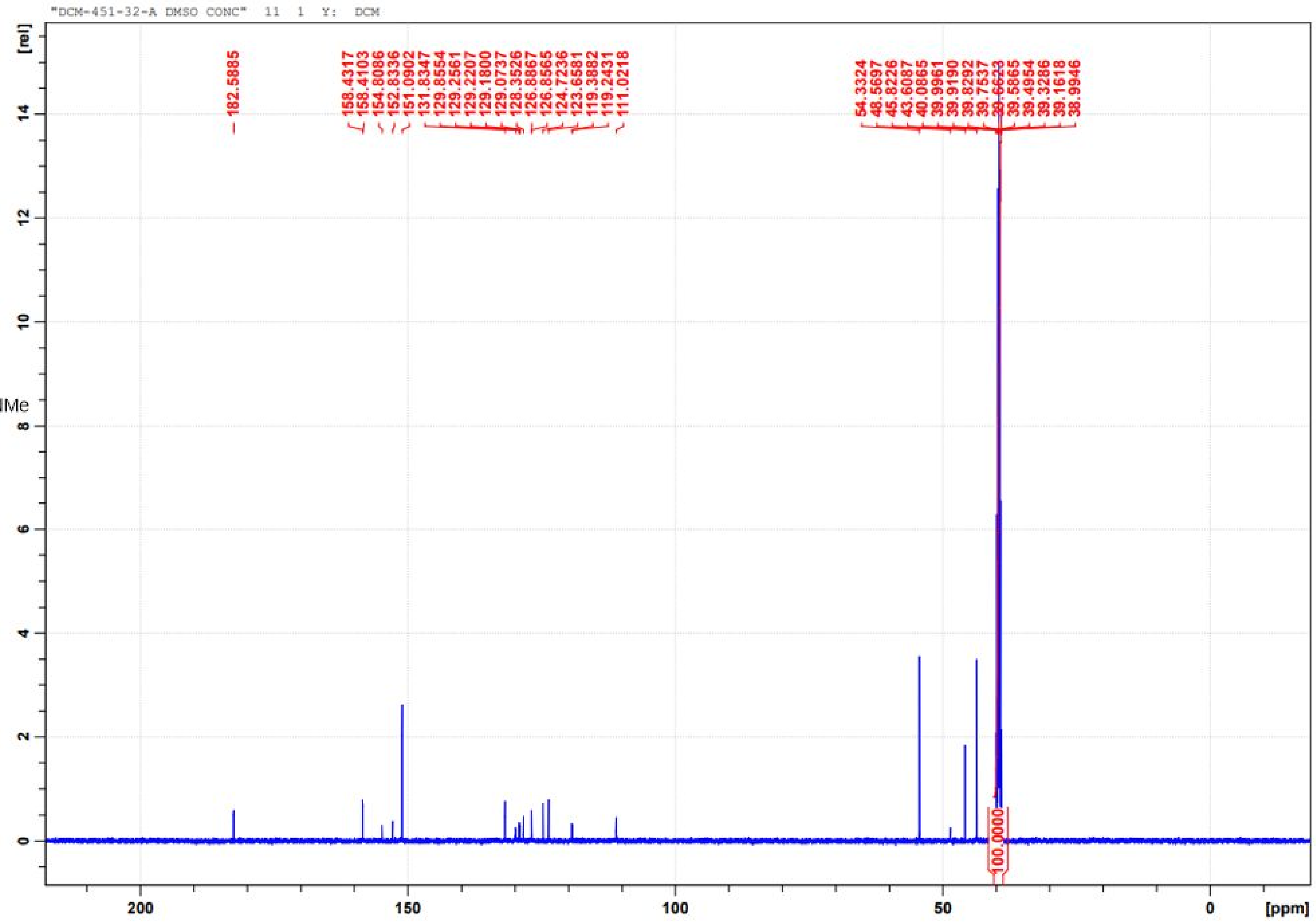

Figure S15: ^{1}H NMR spectrum of compound **32i**

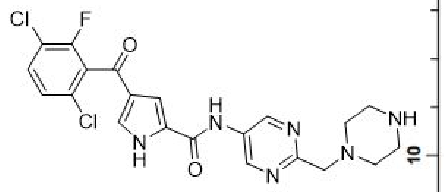

32i

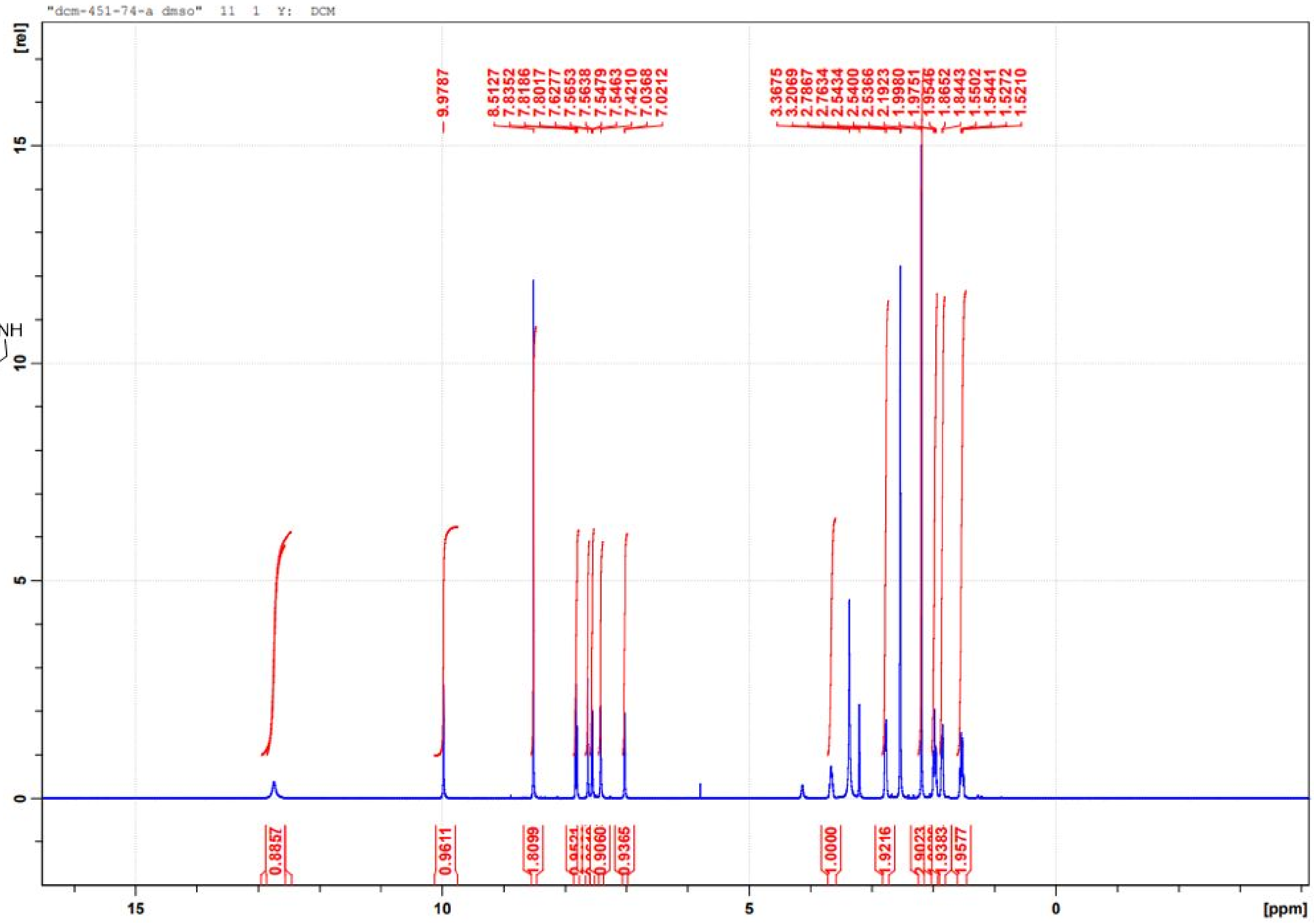

Figure S16: ^{1}H NMR spectrum of compound **32k**

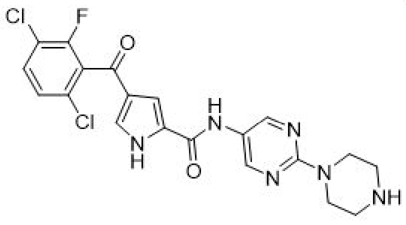

32k

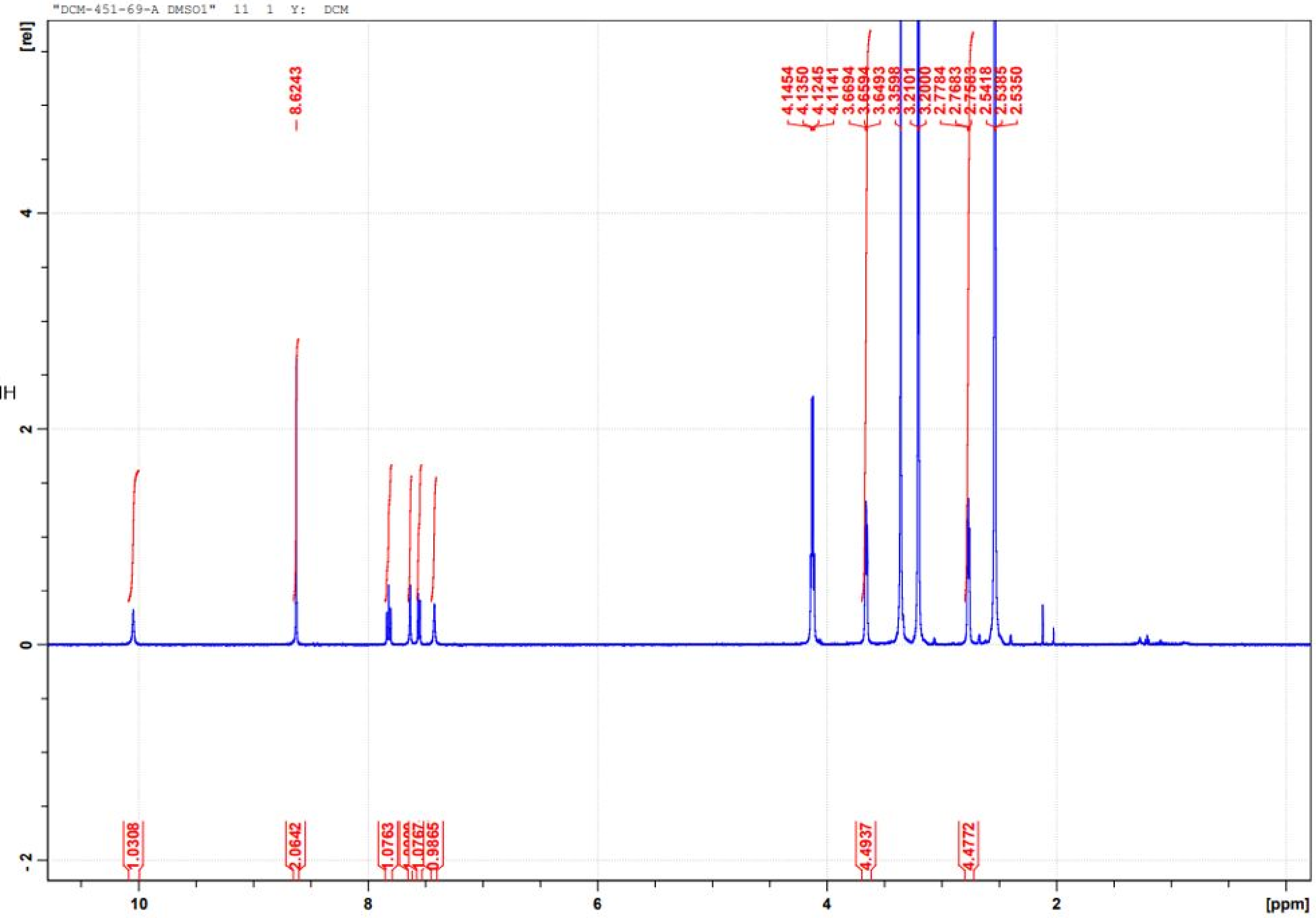

Figure S17: HPLC purity assessment of compound **32k**

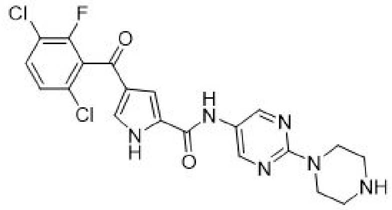

32k

```
Data File F:\31JAN12_12.D
Sample Name: DCM/451/69/A

=====================================================================
Acq. Operator   : Karen Haggerty                  Seq. Line :    8
Acq. Instrument : Instrument 1                     Location : Vial 65
Injection Date  : 1/31/2013 7:28:11 PM                  Inj :    1
                                                 Inj Volume : 5.000 µl
Different Inj Volume from Sequence !      Actual Inj Volume : 2.000 µl
Acq. Method     : C:\CHEM32\1\DATA\JAN2013\31JAN13_AES_RHB_DCM_BJC 2013-01-31 16-16-16\
                  XSELECT_ACIDIC.M
Last changed    : 1/31/2013 4:16:23 PM by Karen Haggerty
Analysis Method : F:\31JAN12_12.D\DA.M (XSELECT_ACIDIC.M)
Last changed    : 2/1/2013 6:48:49 AM by Karen Haggerty
                  (modified after loading)
Method Info     : Waters XSELECT CSH C18 3.5um 100 x 4.6mm
                  0.1% Formic Acid (aq)/MeCN, 1.0mL/min
```

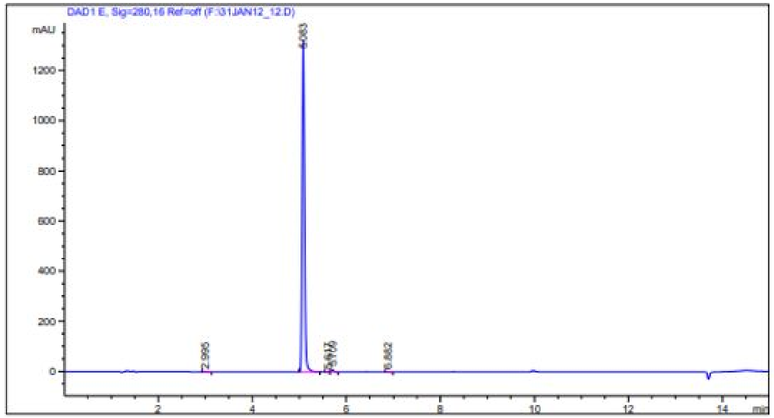


```
=====================================================================
                          Area Percent Report
=====================================================================

Sorted By             :      Signal
Multiplier:                   :      1.0000
Dilution:                     :      1.0000
Use Multiplier & Dilution Factor with ISTDs


Signal 1: DAD1 E, Sig=280,16 Ref=off
```

Peak #	RetTime [min]	Type	Width [min]	Area [mAU*s]	Height [mAU]	Area %
1	2.995	BB	0.0614	12.25065	2.89338	0.2575
2	5.083	VB	0.0553	4698.15674	1328.35876	98.7493
3	5.617	BV	0.0641	5.05713	1.17785	0.1063

Figure S18: ^{1}H NMR spectrum of compound **32l**

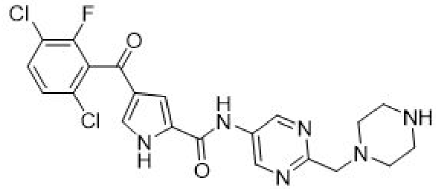

32l

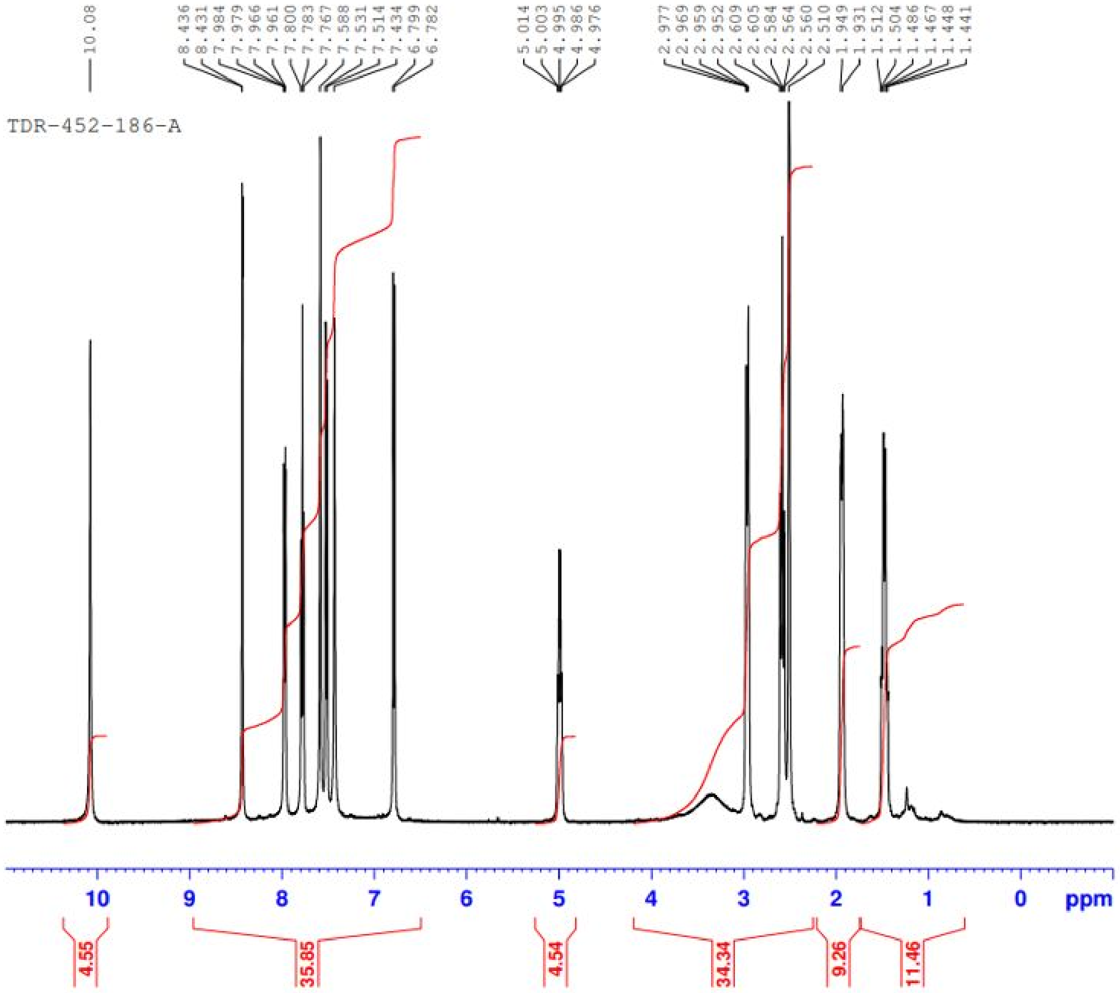

Figure S19: ^{1}H NMR spectrum of compound **32m**

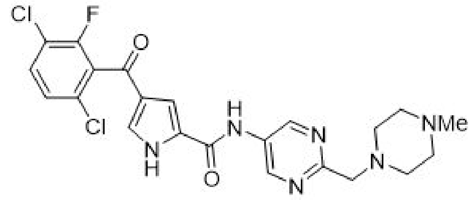

32m

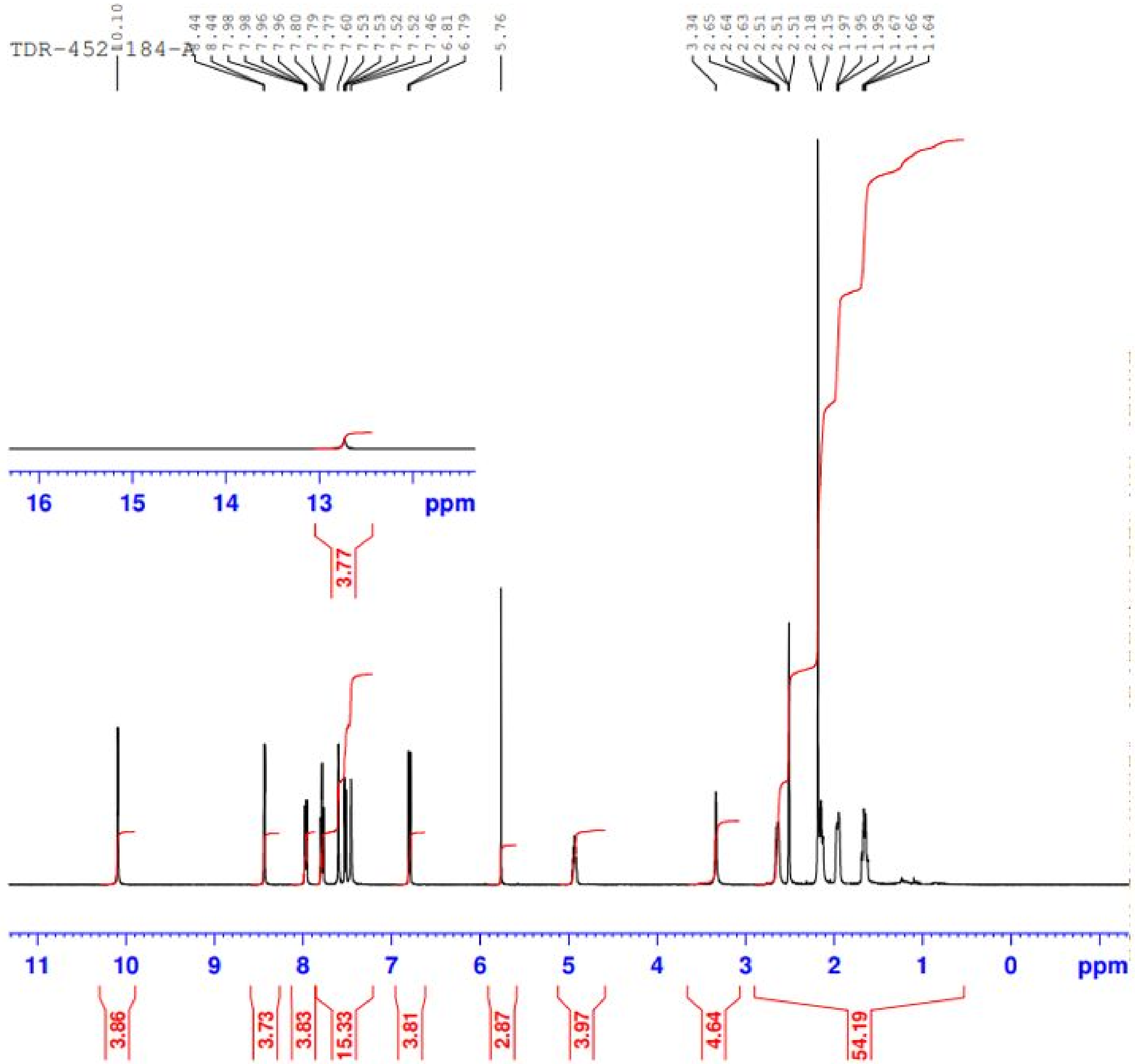

Figure S20: ^{1}H NMR spectrum of compound **33f**

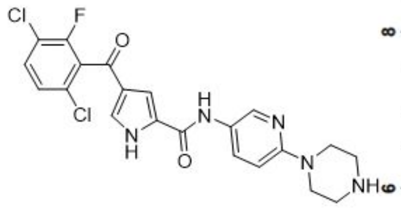

33f

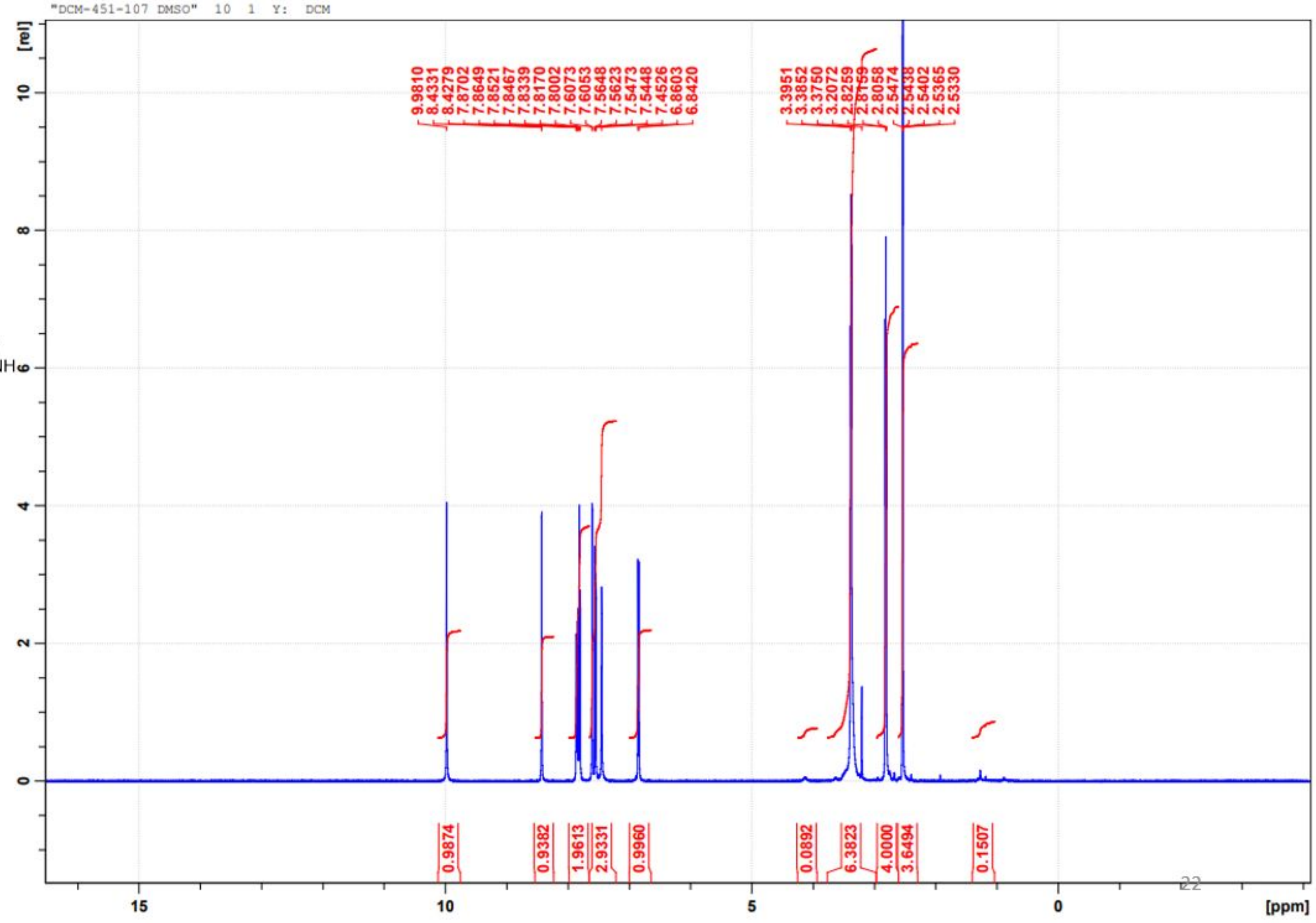

Figure S21: ^{13}C NMR spectrum of compound **33f**

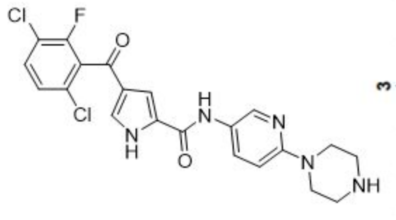

33f

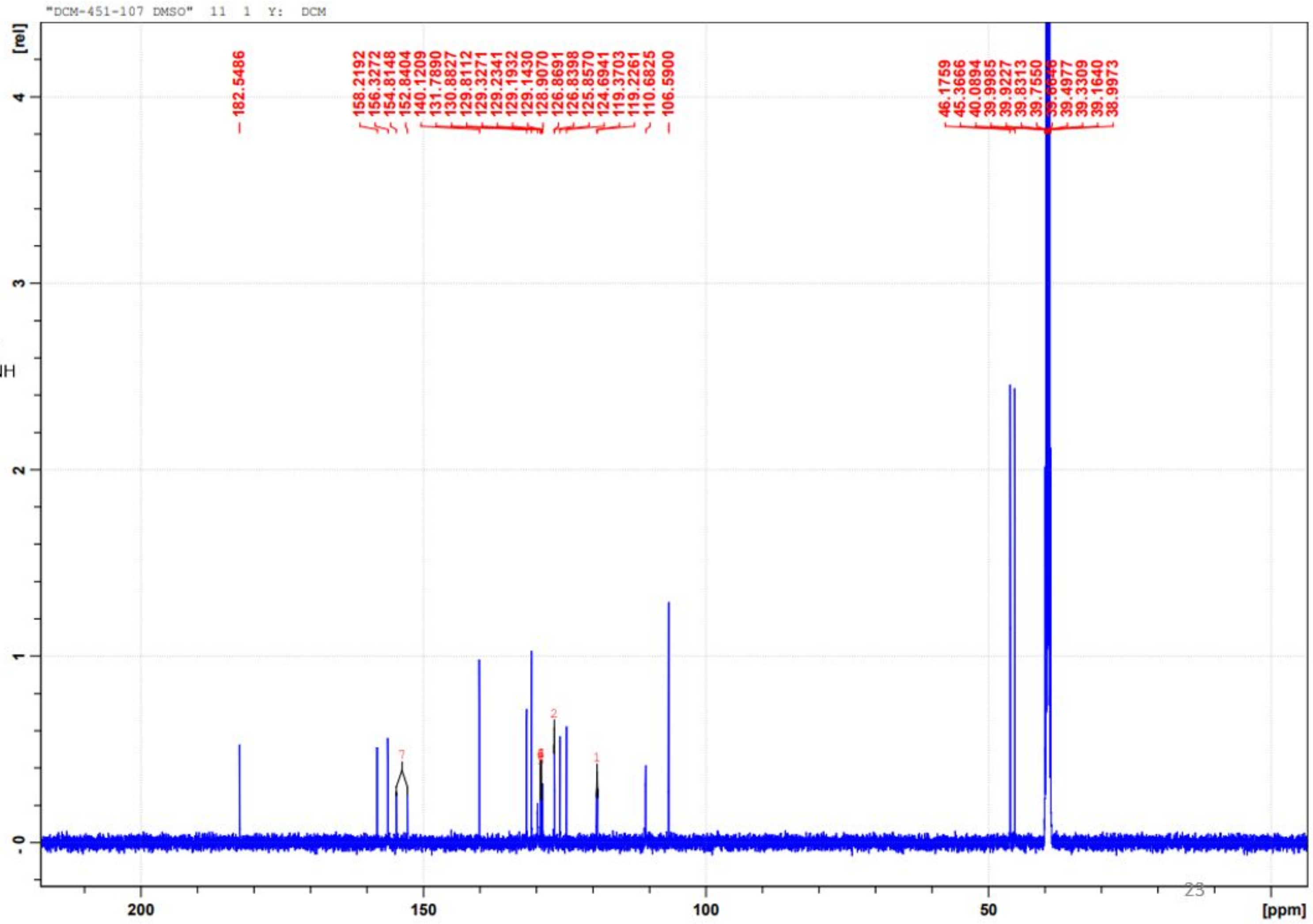

Figure S22: HPLC purity assessment of compound **33f**

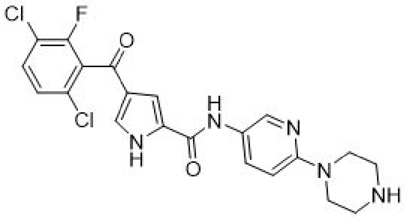

33f

Sample Name: DCM/451/107

```
=====================================================================
Acq. Operator   : Karen Haggerty                  Seq. Line :   5
Acq. Instrument : Instrument 1                     Location : Vial 3
Injection Date  : 3/4/2013 11:36:45 AM                  Inj :   1
                                                 Inj Volume : 5.000 µl
Different Inj Volume from Sequence !     Actual Inj Volume : 1.000 µl
Acq. Method     : C:\CHEM32\1\DATA\MAR2013\04MAR13_DCM_JEP 2013-03-04 09-47-43\XSELECT_
                  ACIDIC.M
Last changed    : 3/4/2013 9:47:51 AM by Karen Haggerty
Analysis Method : F:\04MAR13_07.D\DA.M (XSELECT_ACIDIC.M)
Last changed    : 3/5/2013 6:56:02 AM by Karen Haggerty
                  (modified after loading)
Method Info     : Waters XSELECT CSH C18 3.5um 100 x 4.6mm
                  0.1% Formic Acid (aq)/MeCN, 1.0mL/min
```

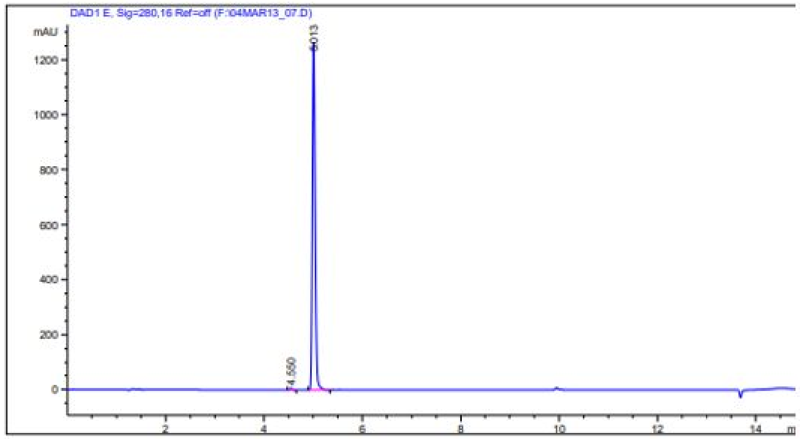


```
=====================================================================
                         Area Percent Report
=====================================================================

Sorted By              :      Signal
Multiplier:                   :       1.0000
Dilution:                     :       1.0000
Use Multiplier & Dilution Factor with ISTDs


Signal 1: DAD1 E, Sig=280,16 Ref=off
```

Peak #	RetTime [min]	Type	Width [min]	Area [mAU*s]	Height [mAU]	Area %
1	4.550	BB	0.0572	24.22210	6.54375	0.4918
2	5.013	VB	0.0611	4901.33496	1268.07605	99.5082

Figure S23: ^{1}H NMR spectrum of compound **33g**

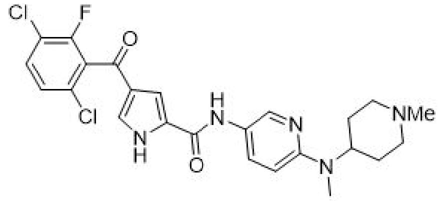

33g

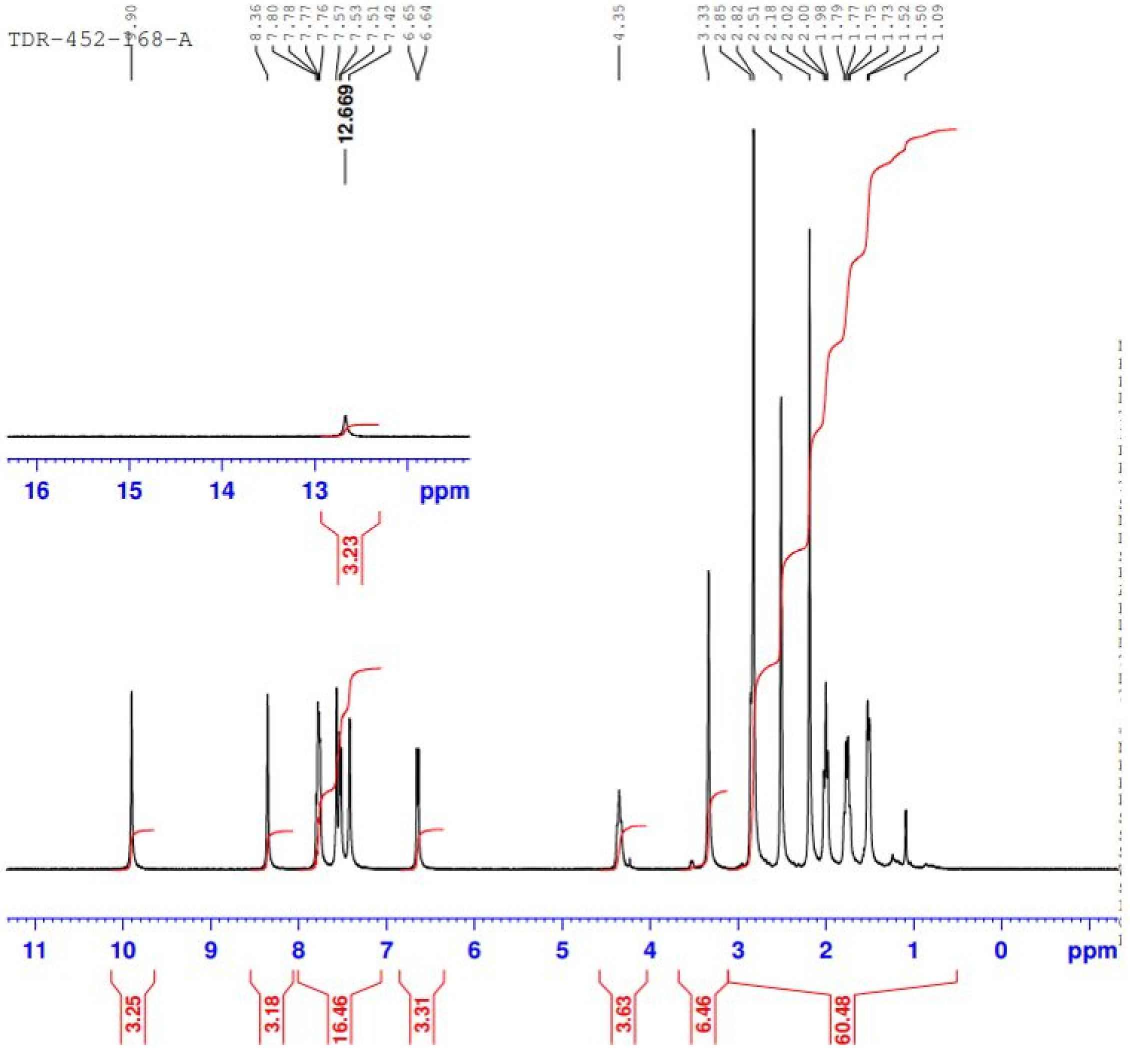

Figure S24: ^{1}H NMR spectrum of compound **33h**

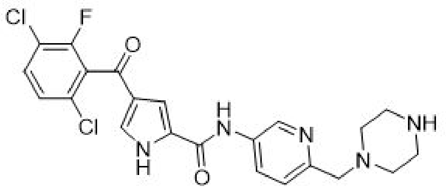

33h

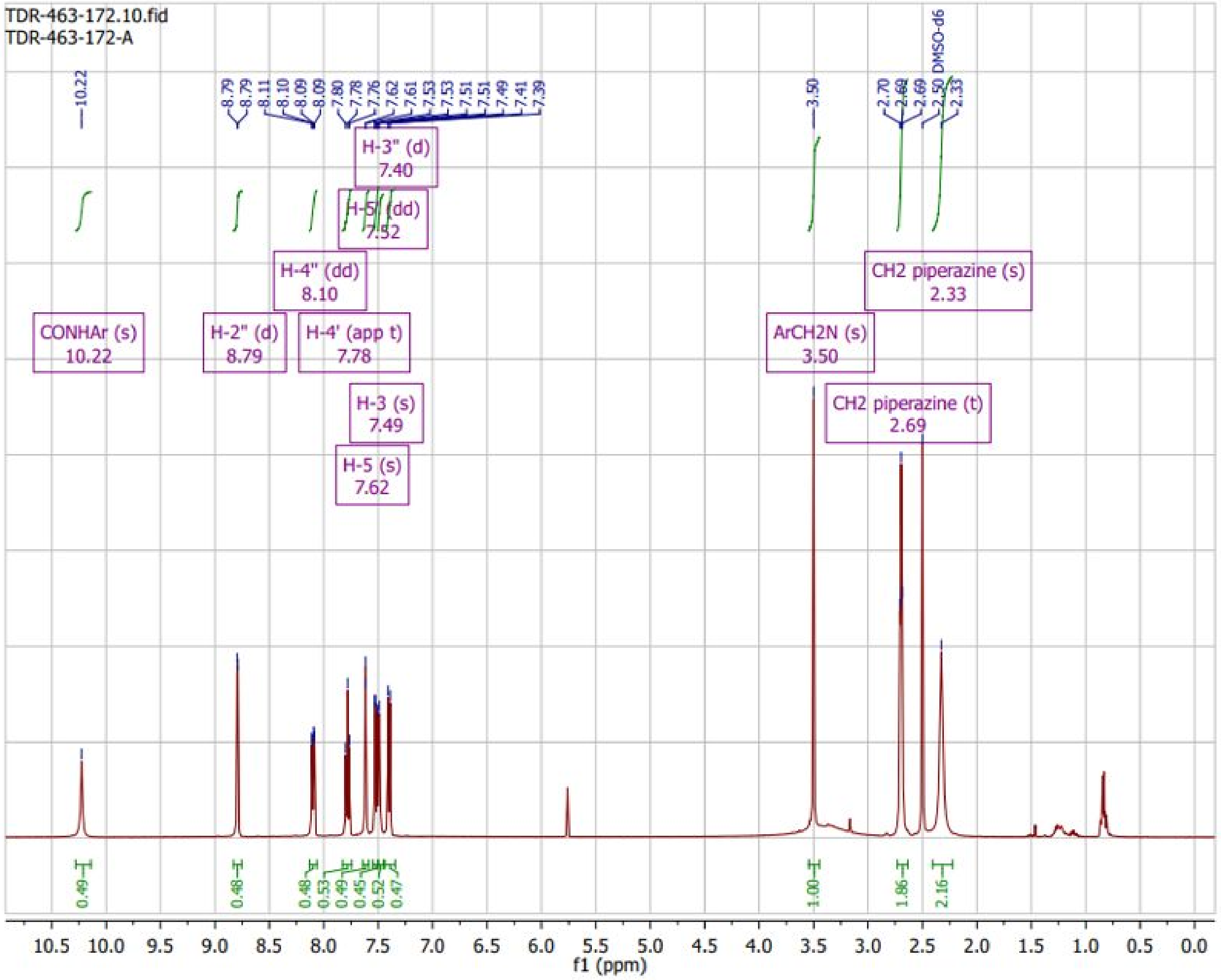

^{1}H NMR (500 MHz, DMSO-d_6) δ 2.33 (s, 4H), 2.69 (t, J = 4.8 Hz, 4H), 3.50 (s, 2H), 7.40 (d, J = 8.4 Hz, 1H), 7.49 (s, 1H), 7.52 (dd, J = 8.8, 1.4 Hz, 1H), 7.62 (s, 1H), 7.78 (app t, J = 8.4 Hz, 1H), 8.10 (dd, J = 8.5, 2.6 Hz, 1H), 8.79 (d, J = 2.5 Hz, 1H), 10.22 (s, 1H).

Figure S25: ^{1}H NMR spectrum of compound **33i**

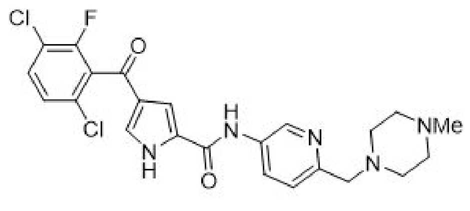

33i

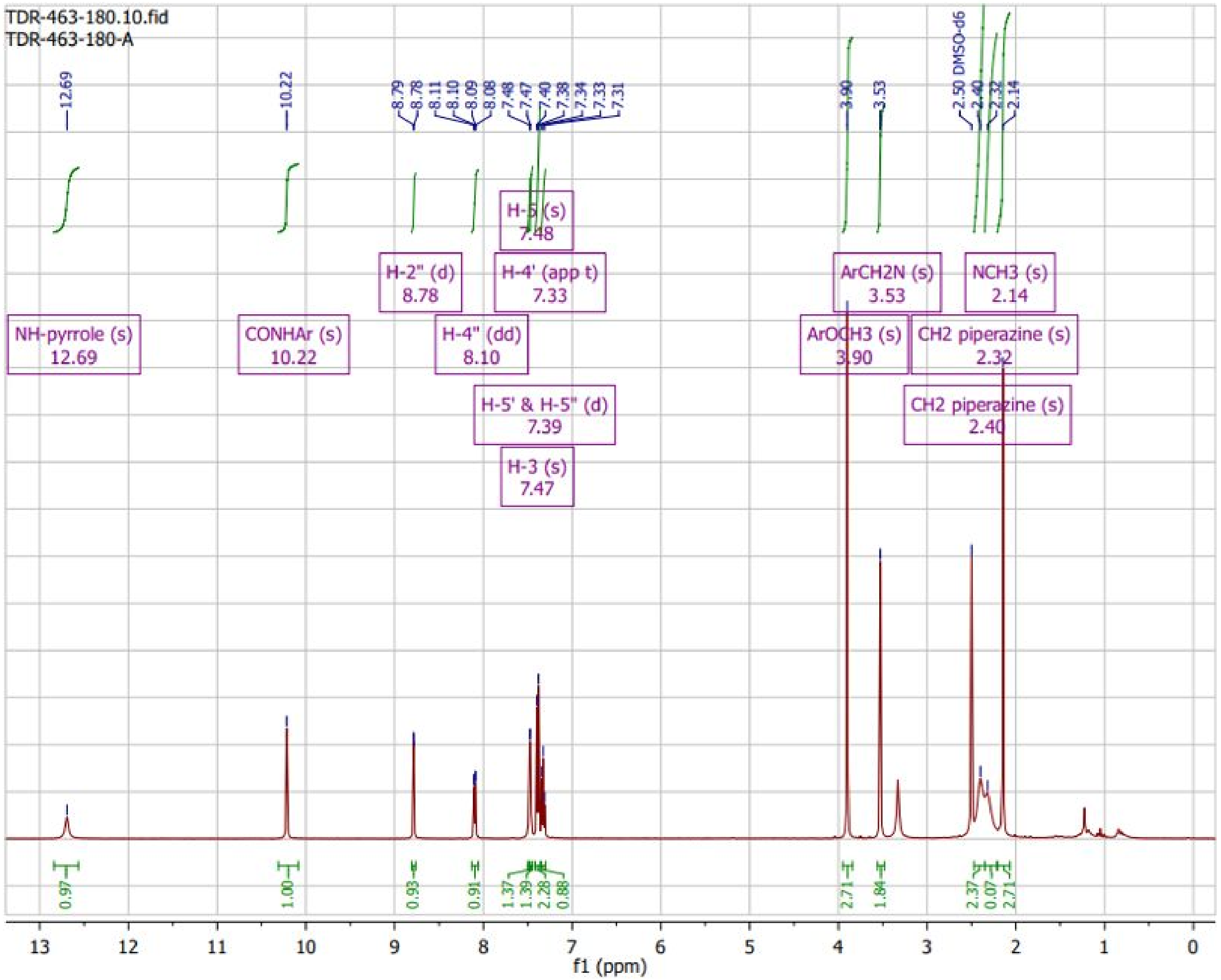

^{1}H NMR (500 MHz, DMSO-d_6) δ 2.14 (s, 3H), 2.32 (s, 4H), 2.40 (s, 4H), 3.53 (s, 2H), 3.90 (s, 3H), 7.33 (app t, *J* = 8.9 Hz, 1H), 7.39 (d, *J* = 8.8 Hz, 2H), 7.47 (s, 1H), 7.48 (s, 1H), 8.10 (dd, *J* = 8.4, 2.6 Hz, 1H), 8.78 (d, *J* = 2.5 Hz, 1H), 10.22 (s, 1H), 12.69 (s, 1H).

Figure S26: ^{13}C NMR spectrum of compound **33i**

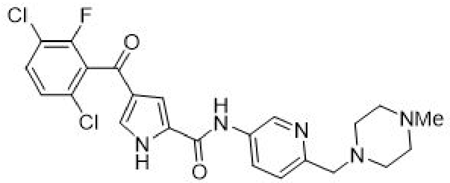

33i

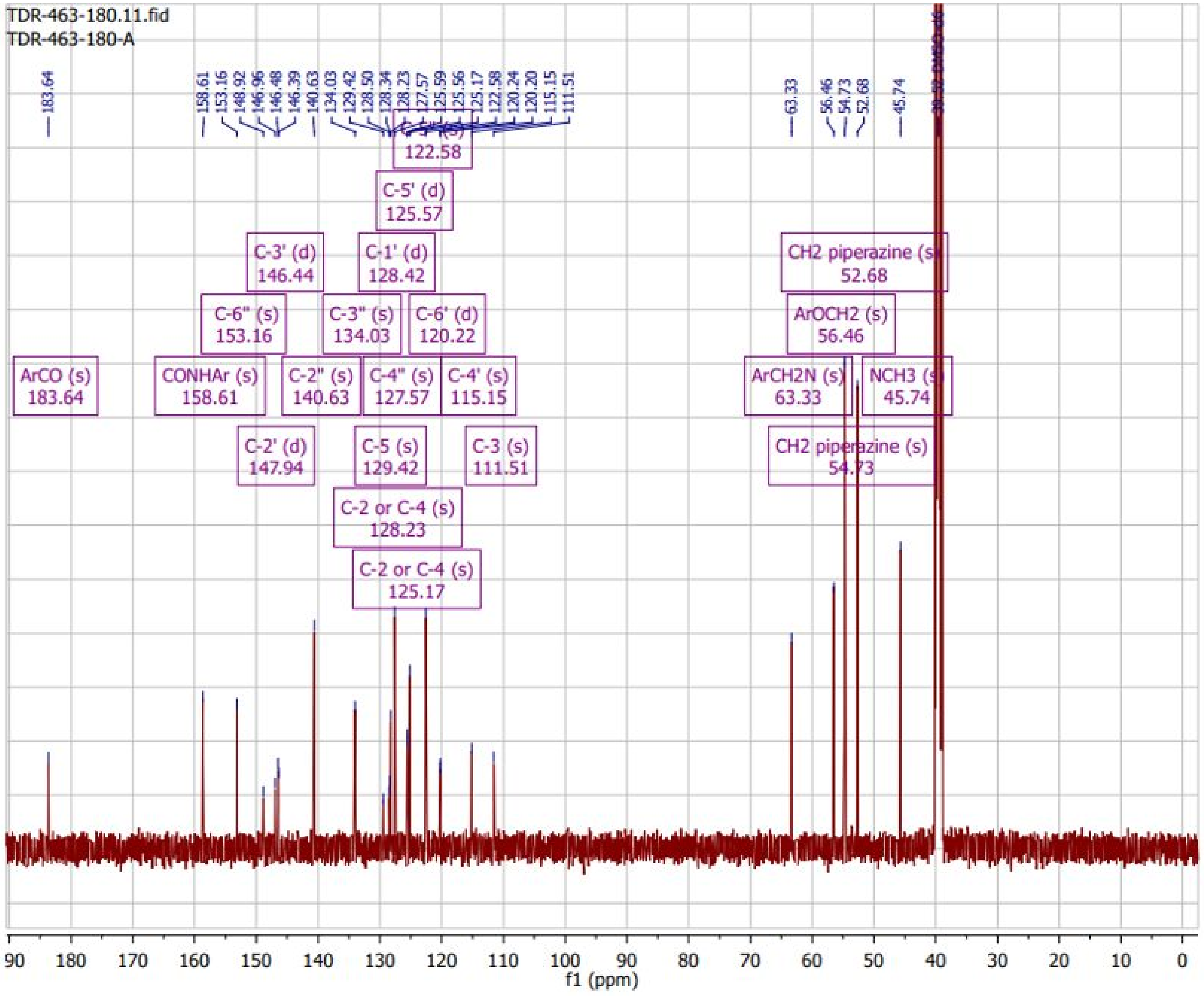

^{13}C NMR (126 MHz, DMSO-d_6) δ 45.7 , 52.7 , 54.7 , 56.5 , 63.3 , 111.5 , 115.1 , 120.2 (d, J = 4.9 Hz), 122.6 , 125.2 , 125.6 (d, J = 3.8 Hz), 127.6 , 128.2 , 128.4 (d, J = 19.9 Hz), 129.4 , 134.0 , 140.6 , 146.4 (d, J = 10.8 Hz), 147.9 (d, J = 247.0 Hz), 153.2 , 158.6 , 183.6 .

Figure S27: ^{1}H NMR spectrum of compound **33j**

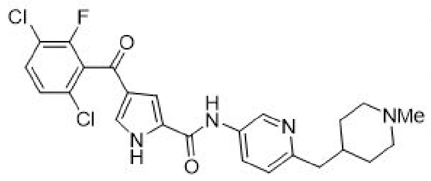

33j

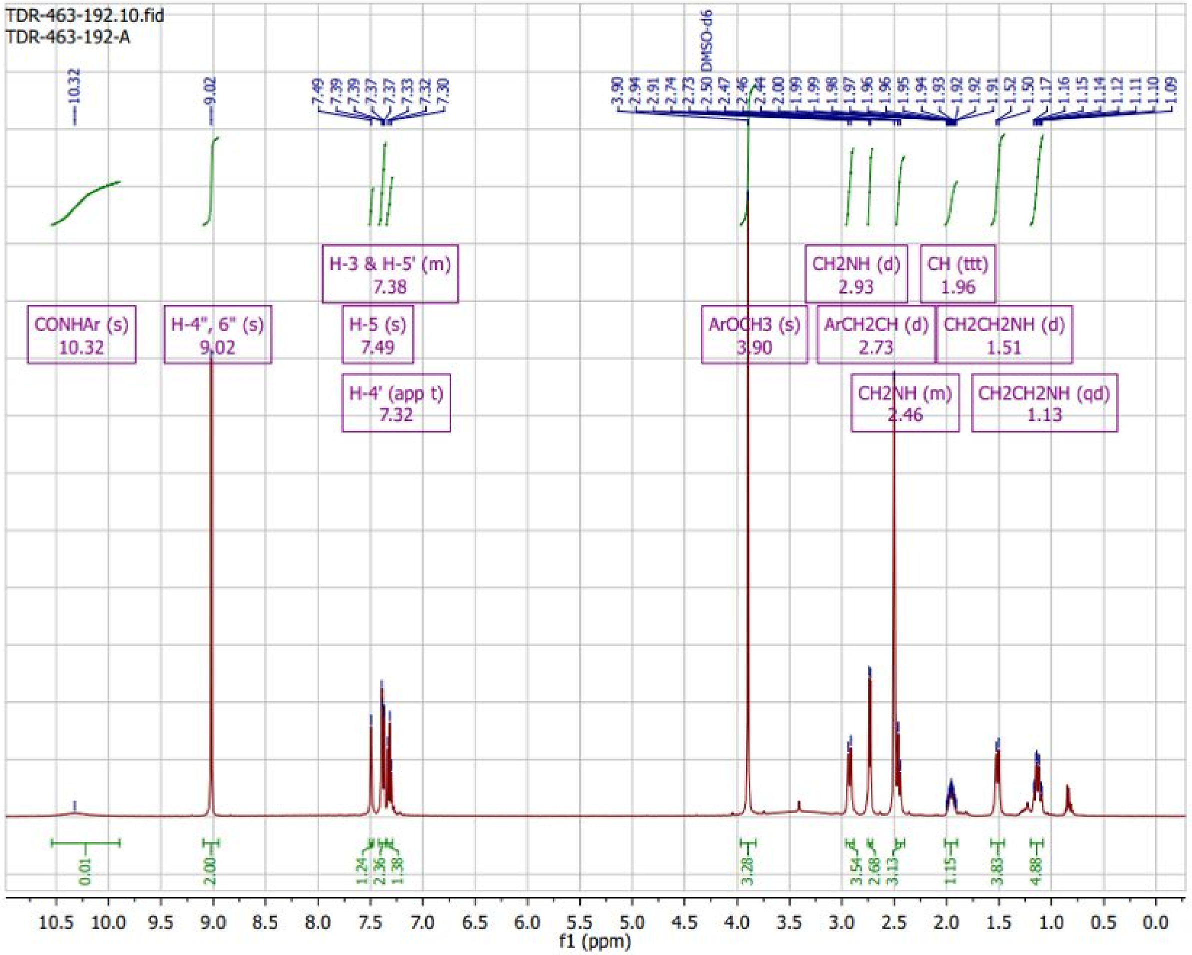

^{1}H NMR (500 MHz, DMSO-d_6) δ 1.13 (qd, J = 12.3, 3.8 Hz, 2H), 1.51 (d, J = 11.1 Hz, 2H), 1.96 (ttt, J = 11.2, 7.2, 3.9 Hz, 1H), 2.40 – 2.48 (m, 2H), 2.73 (d, J = 7.1 Hz, 2H), 2.93 (d, J = 12.1 Hz, 2H), 3.90 (s, 3H), 7.32 (app t, J = 9.0 Hz, 1H), 7.35 – 7.42 (m, 2H), 7.49 (s, 1H), 9.02 (s, 2H), 10.32 (s, 1H).

Figure S28: ^{13}C NMR spectrum of compound **33j**

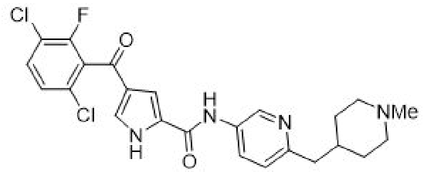

33j

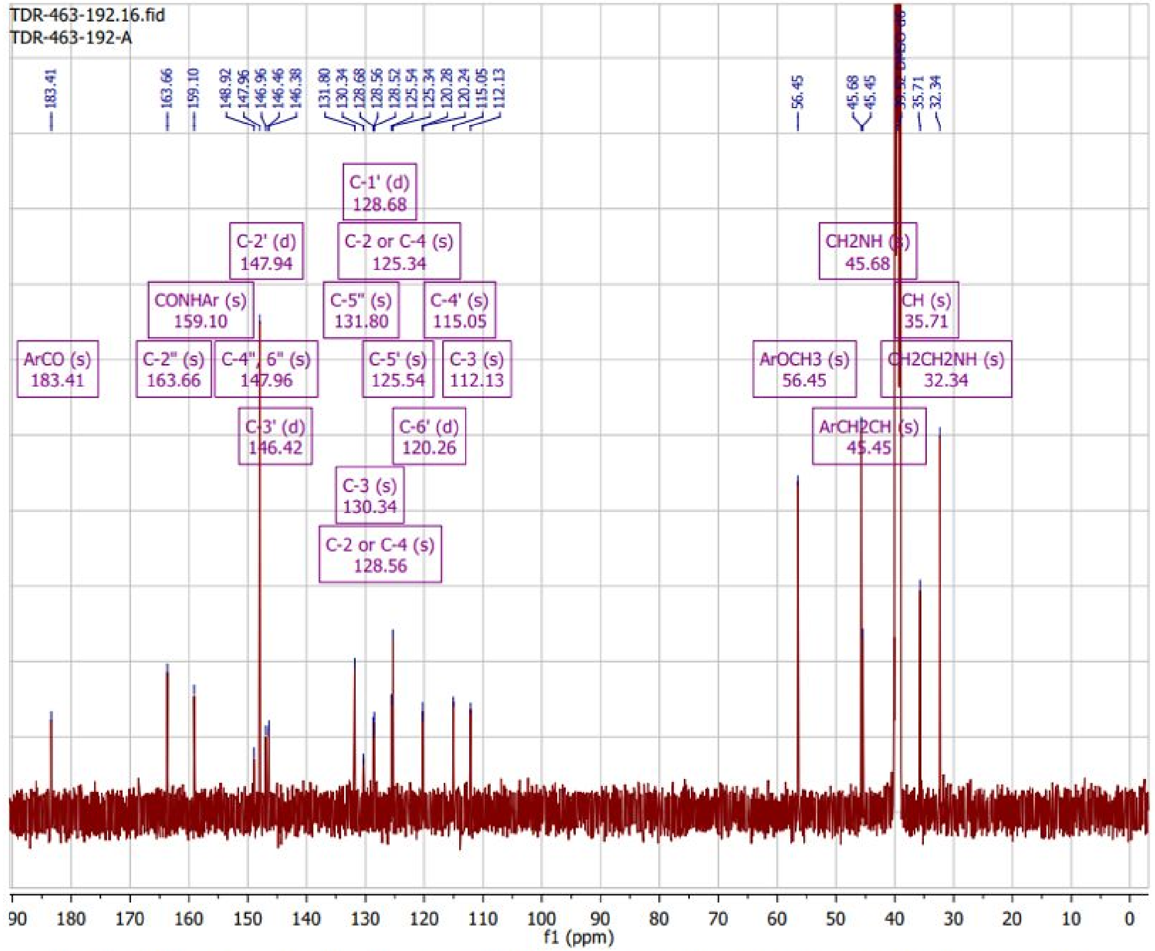

^{13}C NMR (126 MHz, DMSO-d_6) δ 32.3 , 35.7 , 45.5 , 45.7 , 56.4 , 112.1 , 115.0 , 120.3 (d, J = 5.0 Hz), 125.3 , 125.5 , 128.6 , 128.7 (d, J = 20.6 Hz), 130.3 , 131.8 , 146.4 (d, J = 10.6 Hz), 147.9 (d, J = 246.8 Hz), 148.0 , 159.1 , 163.7 , 183.4 .

Figure S29: HPLC purity assessment of compound **33j**

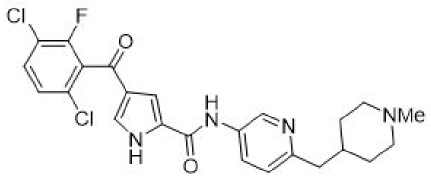

33j

Data File D:\DATASHARE\06NOV13_12.D
Sample Name: TDR/463/192/A

```
=====================================================================
Acq. Operator   : Karen Haggerty                  Seq. Line :    8
Acq. Instrument : Instrument 1                     Location : Vial 45
Injection Date  : 11/6/2013 7:05:11 PM                  Inj :    1
                                                Inj Volume : 5.000 µl
Different Inj Volume from Sequence !      Actual Inj Volume : 4.000 µl
Acq. Method     : C:\CHEM32\1\DATA\NOV2013\06NOV13_TDR_SA 2013-11-06 15-53-38\XSELECT_ACIDIC.
                  M
Last changed    : 11/6/2013 3:53:44 PM by Karen Haggerty
Analysis Method : D:\DATASHARE\06NOV13_12.D\DA.M (XSELECT_ACIDIC.M)
Last changed    : 11/7/2013 6:19:39 AM by Karen Haggerty
                  (modified after loading)
Method Info     : Waters XSELECT CSH C18 3.5um 100 x 4.6mm
                  0.1% Formic Acid (aq)/MeCN, 1.0mL/min
```

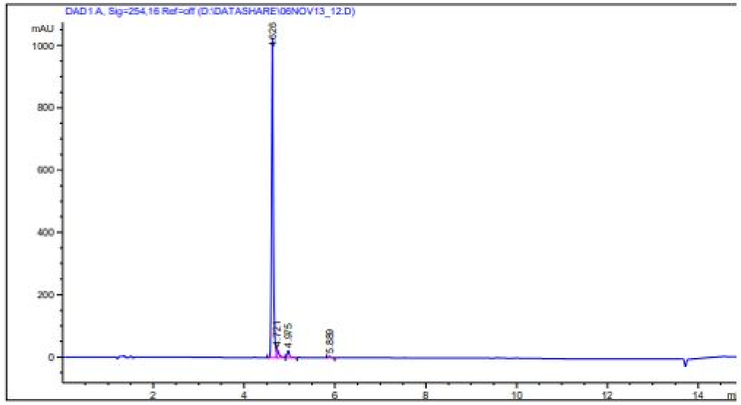


```
=====================================================================
                         Area Percent Report
=====================================================================

Sorted By              :      Signal
Multiplier:                   :      1.0000
Dilution:                     :      1.0000
Use Multiplier & Dilution Factor with ISTDs


Signal 1: DAD1 A, Sig=254,16 Ref=off
```

Peak #	RetTime [min]	Type	Width [min]	Area [mAU*s]	Height [mAU]	Area %
1	4.626	MF	0.0551	3420.13989	1034.51111	94.8151
2	4.721	FM	0.0542	94.03802	28.89498	2.6070
3	4.975	MF	0.0615	79.54386	21.56478	2.2052

Figure S30: ^{1}H NMR spectrum of compound **33k**

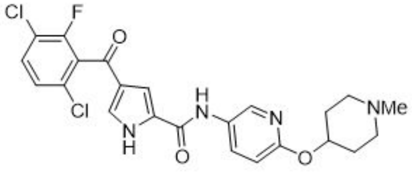

33k

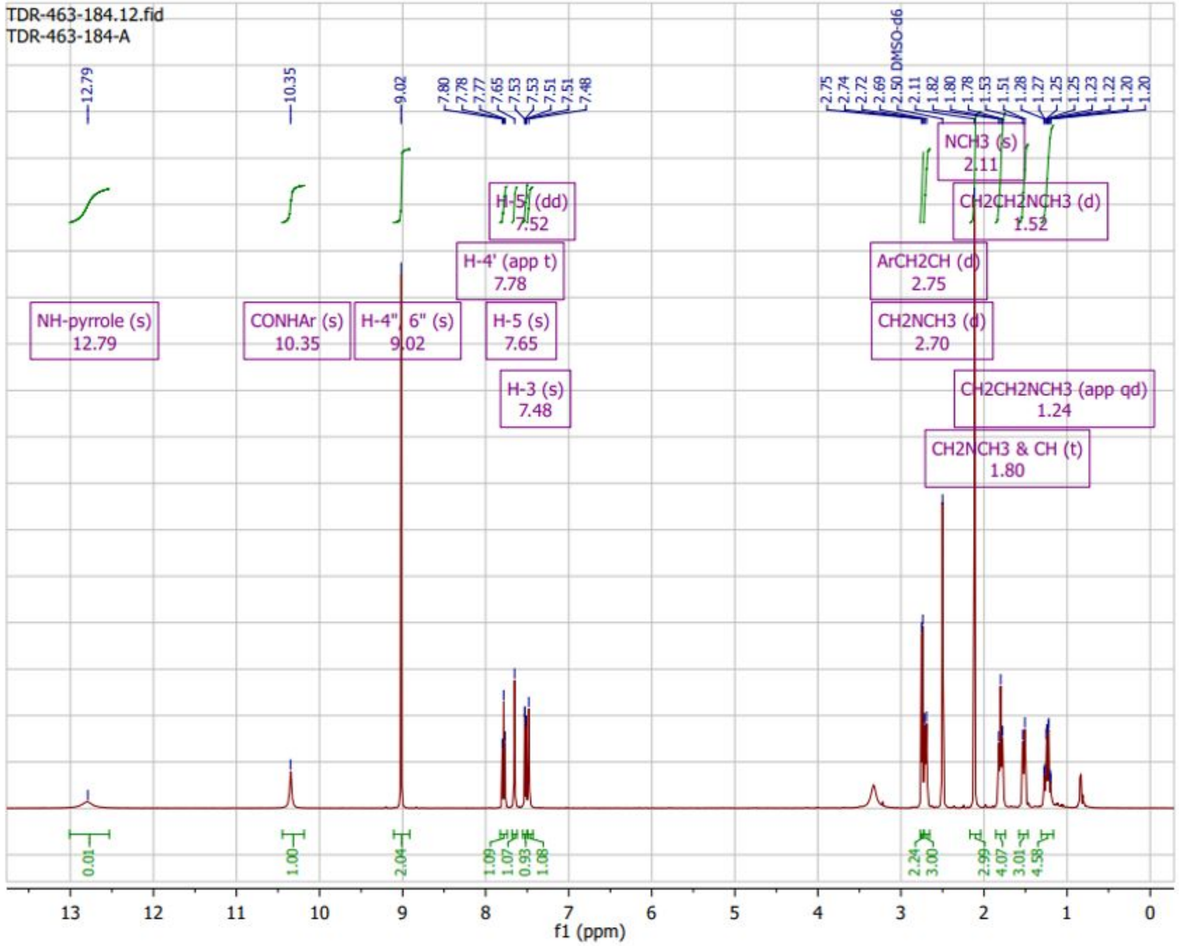

^{1}H NMR (500 MHz, DMSO-d_6) δ 1.24 (app qd, *J* = 12.3, 3.6 Hz, 2H), 1.52 (d, *J* = 12.2 Hz, 2H), 1.80 (t, *J* = 11.0 Hz, 4H), 2.11 (s, 3H), 2.70 (d, *J* = 11.4 Hz, 2H), 2.75 (d, *J* = 7.1 Hz, 2H), 7.48 (s, 1H), 7.52 (dd, *J* = 8.7, 1.3 Hz, 1H), 7.65 (s, 1H), 7.78 (app t, *J* = 8.4 Hz, 1H), 9.02 (s, 2H), 10.35 (s, 1H), 12.79 (s, 1H).32

Figure S31: ^{13}C NMR spectrum of compound **33k**

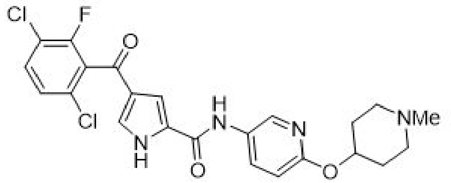

33k

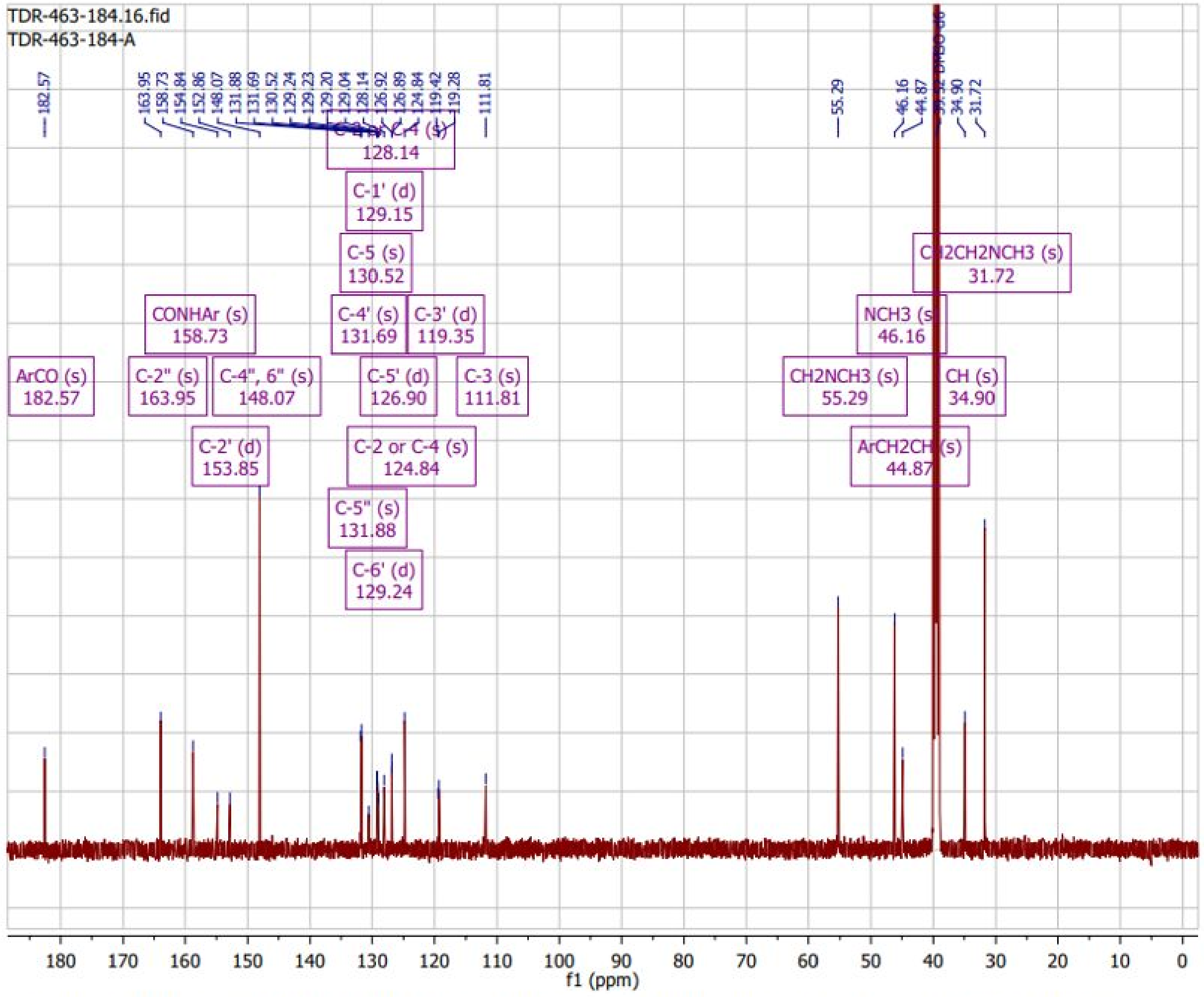

^{13}C NMR (126 MHz, DMSO-d_6) δ 31.7 , 34.9 , 44.9 , 46.2 , 55.3 , 111.8 , 119.4 (d, J = 18.1 Hz), 124.8 , 126.9 (d, J = 3.9 Hz), 128.1 , 129.2 (d, J = 23.3 Hz), 129.2 (d, J = 5.0 Hz), 130.5 , 131.7 , 131.9 , 148.1 , 153.9 (d, J = 248.7 Hz), 158.7 , 163.9 , 182.6 .

Figure S32: HPLC purity assessment of compound **33k**

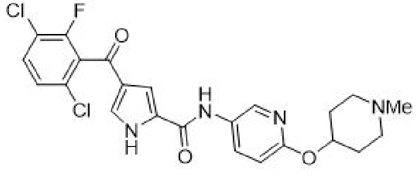

33k

Data File D:\DATASHARE\05NOV13_12.D
Sample Name: TDR/463/184/A

```
=====================================================================
Acq. Operator   : Karen Haggerty                  Seq. Line :   8
Acq. Instrument : Instrument 1                     Location : Vial 45
Injection Date  : 11/5/2013 7:11:29 PM                   Inj :   1
                                                Inj Volume : 5.000 µl
Different Inj Volume from Sequence !     Actual Inj Volume : 2.000 µl
Acq. Method     : C:\CHEM32\1\DATA\NOV2013\05NOV13_TDR_JEP_AB 2013-11-05 16-00-04\XSELECT_
                  ACIDIC.M
Last changed    : 11/5/2013 4:00:12 PM by Karen Haggerty
Analysis Method : D:\DATASHARE\05NOV13_12.D\DA.M (XSELECT_ACIDIC.M)
Last changed    : 11/6/2013 7:37:34 AM by Karen Haggerty
                  (modified after loading)
Method Info     : Waters XSELECT CSH C18 3.5um 100 x 4.6mm
                  0.1% Formic Acid (aq)/MeCN, 1.0mL/min
```

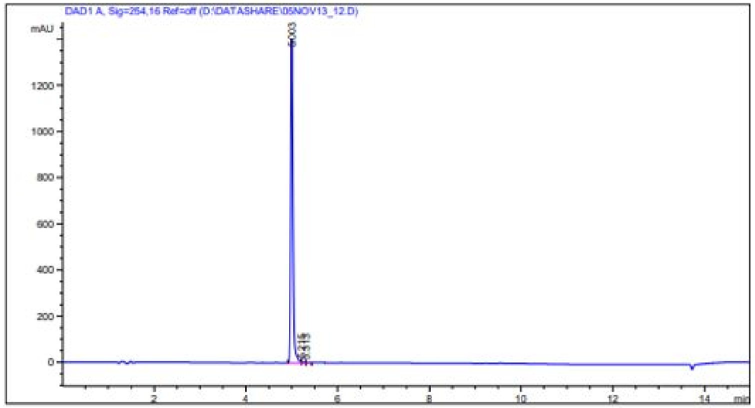


```
=====================================================================
                        Area Percent Report
=====================================================================

Sorted By             :      Signal
Multiplier:                  :      1.0000
Dilution:                    :      1.0000
Use Multiplier & Dilution Factor with ISTDs
```

Signal 1: DAD1 A, Sig=254,16 Ref=off

Peak #	RetTime [min]	Type	Width [min]	Area [mAU*s]	Height [mAU]	Area %
1	5.003	MF	0.0567	4798.38086	1409.70972	99.5686
2	5.215	FM	0.0568	14.24326	4.18153	0.2956
3	5.313	FM	0.0844	6.54838	1.29375	0.1359

Figure S33: ^{1}H NMR spectrum of compound **34a**

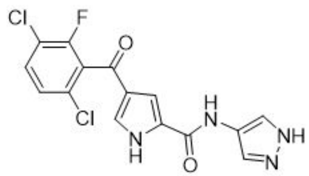

34a

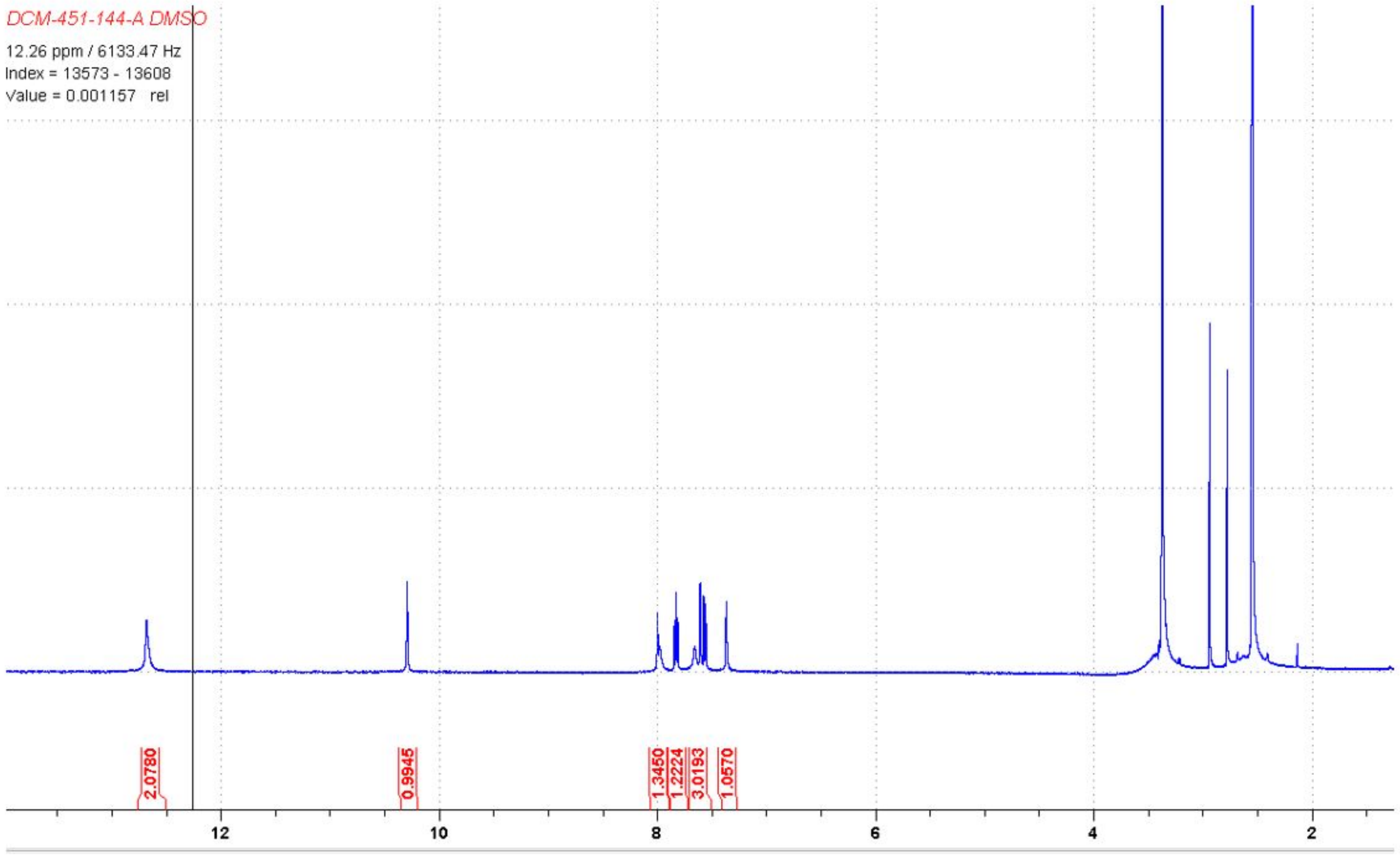

Figure S34: ^{1}H NMR spectrum of compound **34b**

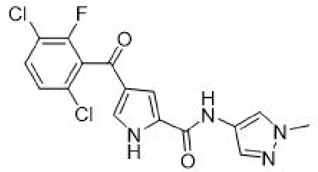

34b

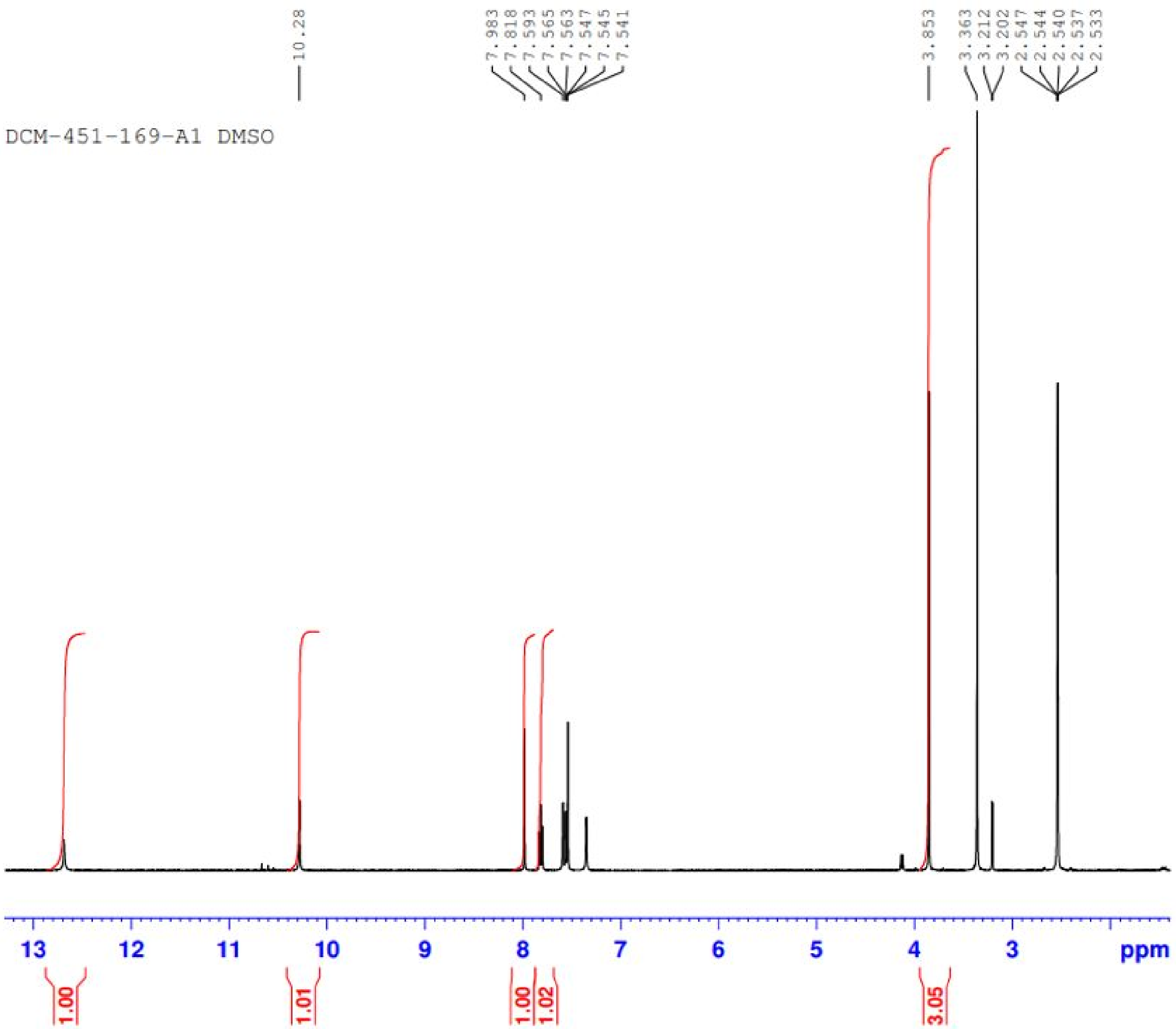

Figure S35: ^{13}C NMR spectrum of compound **34b**

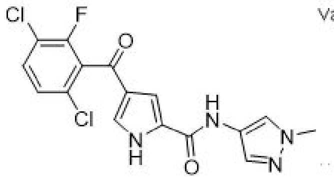

34b

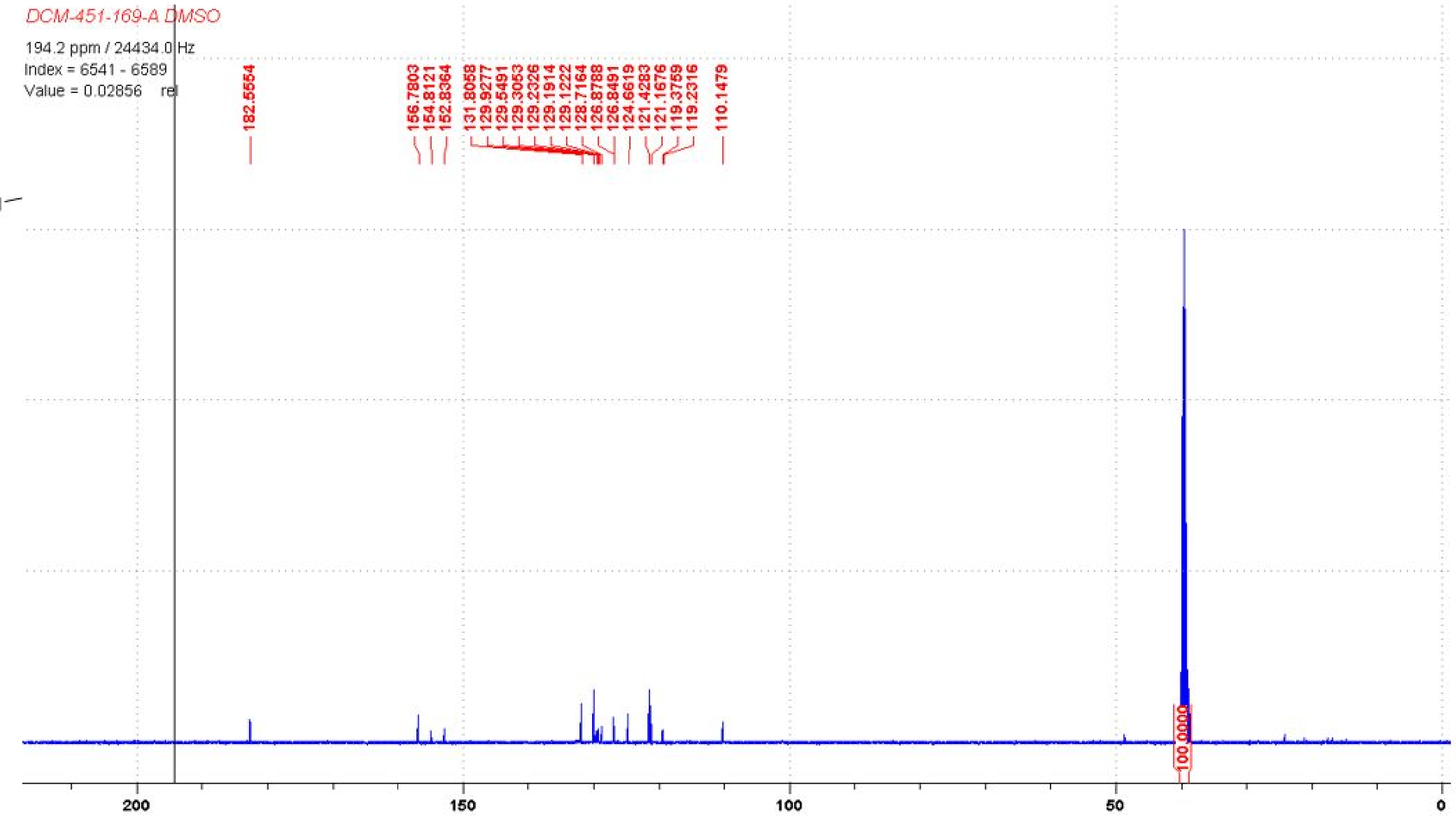

Figure S36: HPLC purity assessment of compound **34b**

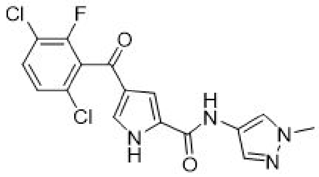

34b

Sample Name: DCM/451/169

===
Acq. Operator   : Karen Haggerty   Seq. Line : 11
Acq. Instrument : Instrument 1   Location : Vial 48
Injection Date : 5/24/2013 9:01:10 PM   Inj :   1
                                                 Inj Volume : 5.000 µl
Different Inj Volume from Sequence !   Actual Inj Volume : 3.000 µl
Acq. Method   : C:\CHEM32\1\DATA\MAY2013\24MAY13_BZ_TDR_SJH_HL_DCM_BJC_NCM_SCOUT 2013-05-24
                  16-08-36\XSELECT_ACIDIC.M
Last changed   : 5/24/2013 4:08:45 PM by Karen Haggerty
Analysis Method : F:\24MAY13_18.D\DA.M (XSELECT_ACIDIC.M)
Last changed   : 5/28/2013 7:14:44 AM by Karen Haggerty
                  (modified after loading)
Method Info   : Waters XSELECT CSH C18 3.5um 100 x 4.6mm
                  0.1% Formic Acid (aq)/MeCN, 1.0mL/min

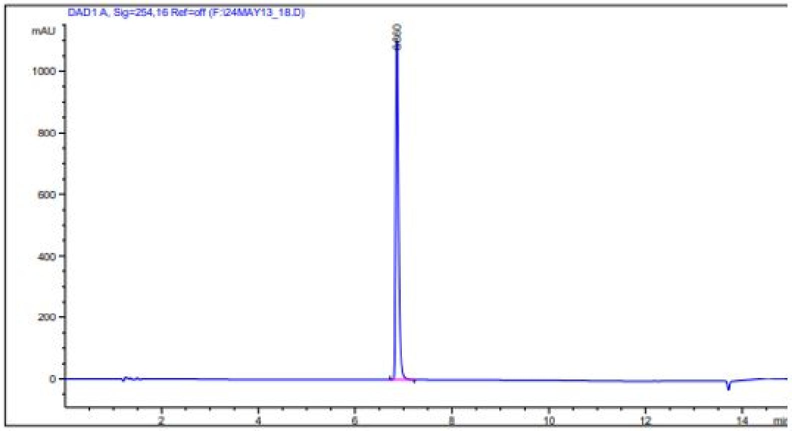

===
Area Percent Report
===

Sorted By   :   Signal
Multiplier: :   1.0000
Dilution: :   1.0000
Use Multiplier & Dilution Factor with ISTDs

Signal 1: DAD1 A, Sig=254,16 Ref=off

Peak #	RetTime [min]	Type	Width [min]	Area [mAU*s]	Height [mAU]	Area %
1	6.860	VB	0.0614	4472.47998	1101.00159	100.0000

Totals :   4472.47998 1101.00159

Figure S37: ^{1}H NMR spectrum of compound **34c**

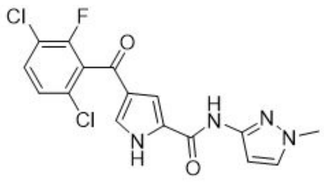

34c

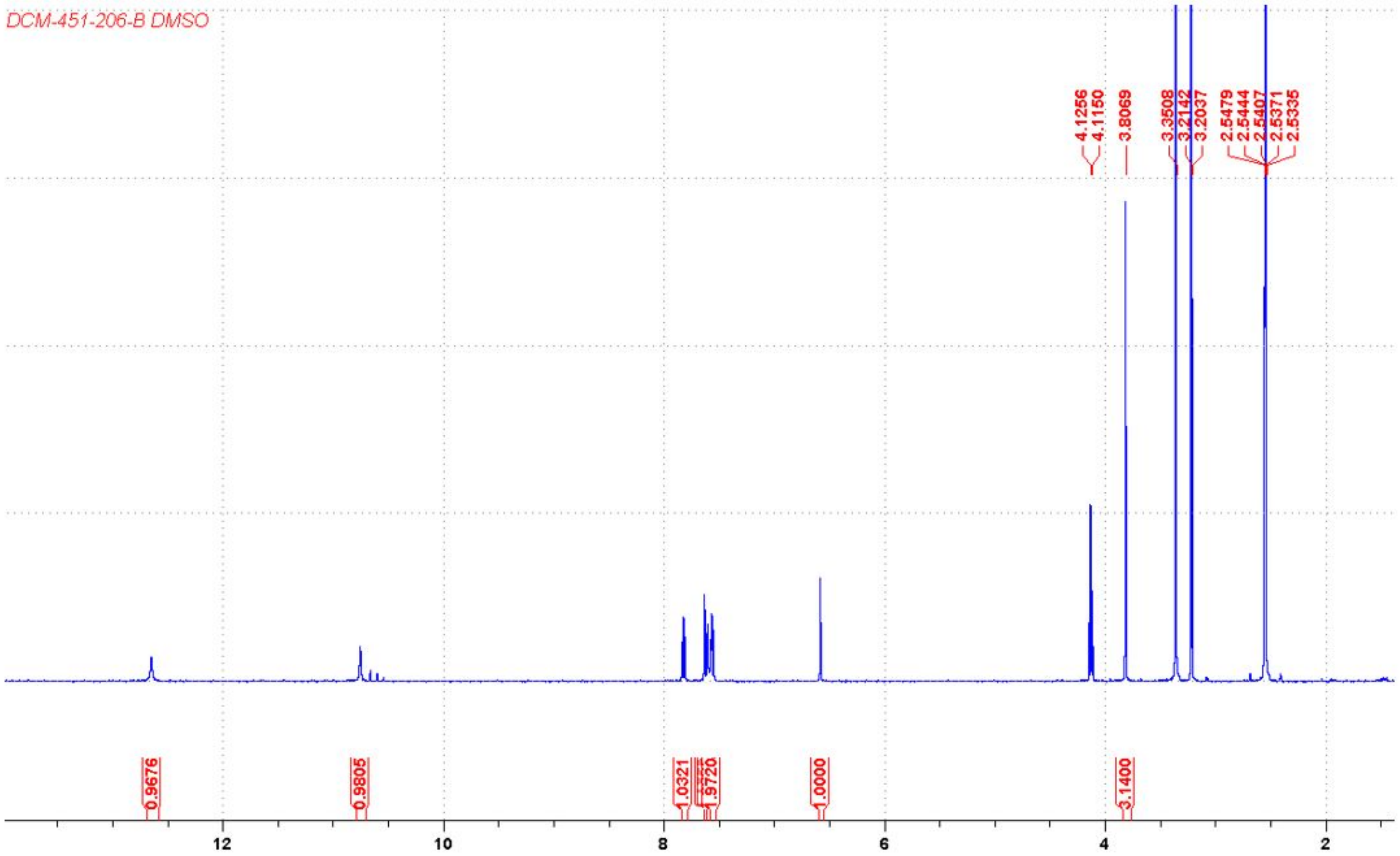

Figure S38: ^{1}H NMR spectrum of compound **34d**

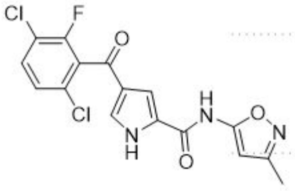

34d

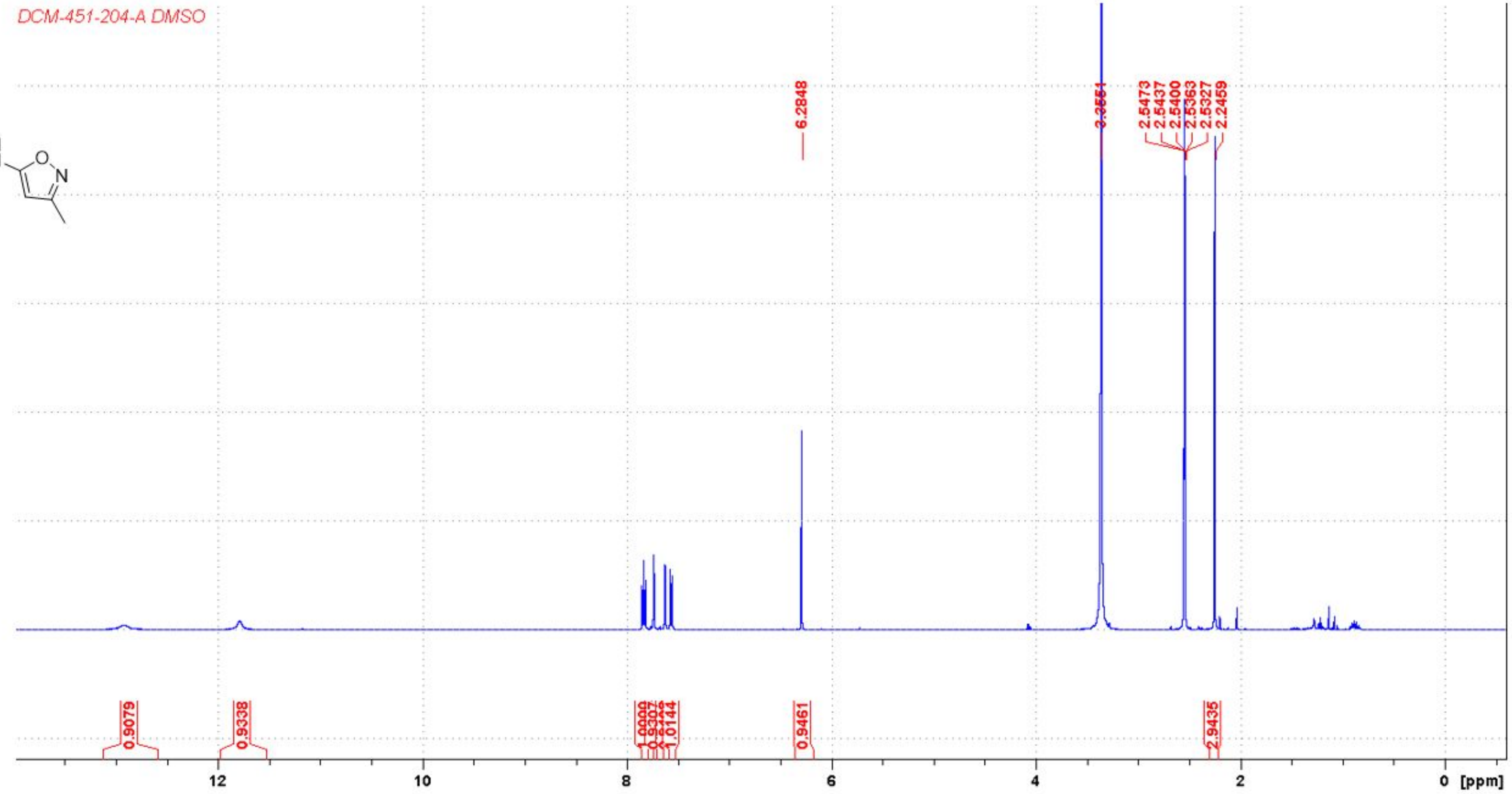

Figure S39: ^{13}C NMR spectrum of compound **34d**

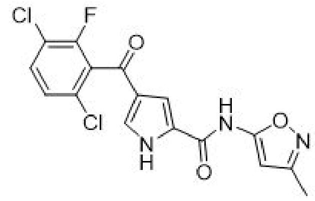

34d

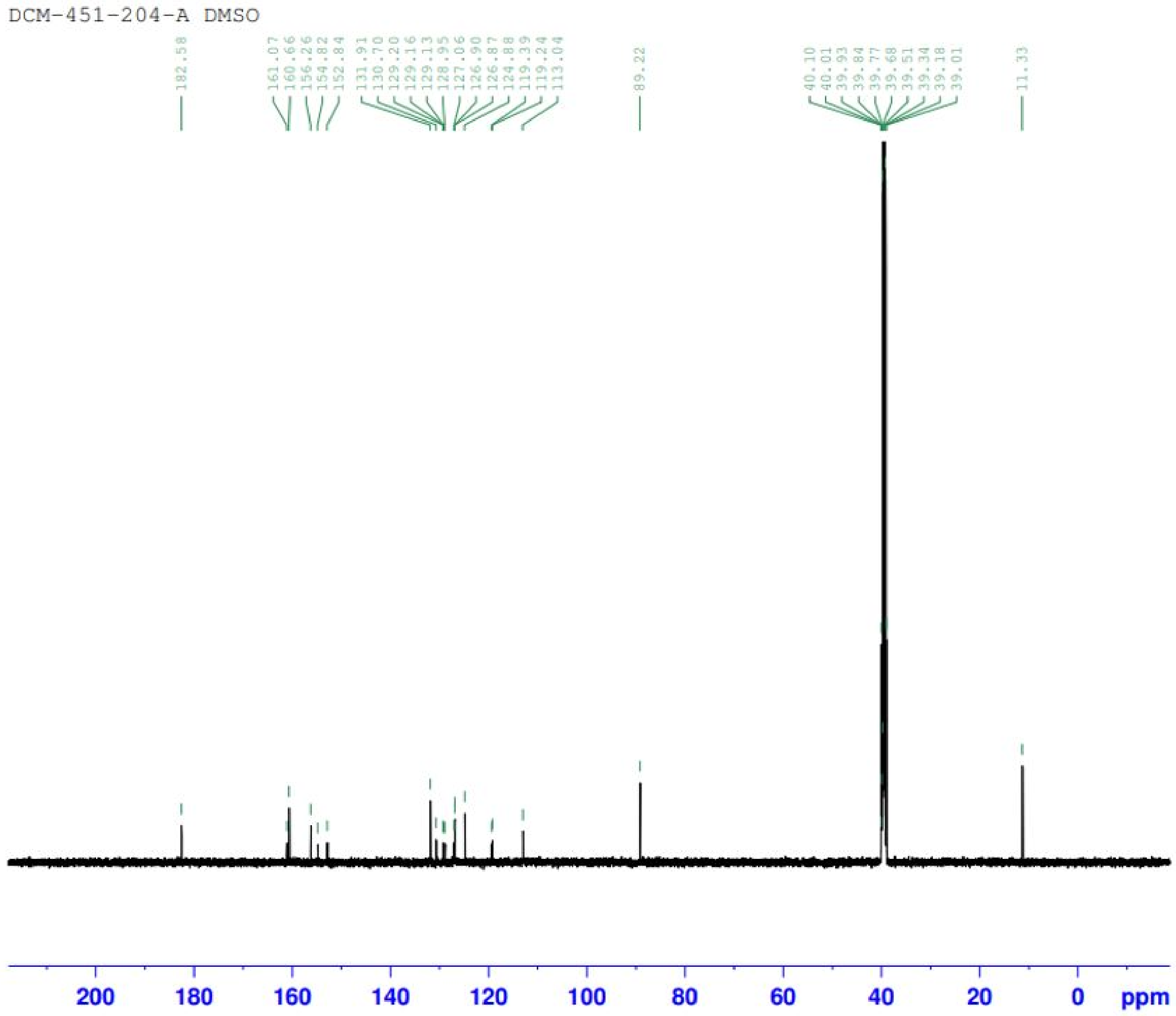

Figure S40: ^{1}H NMR spectrum of compound **34e**

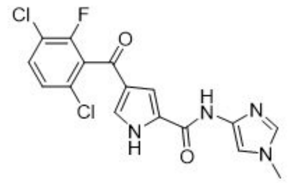

34e

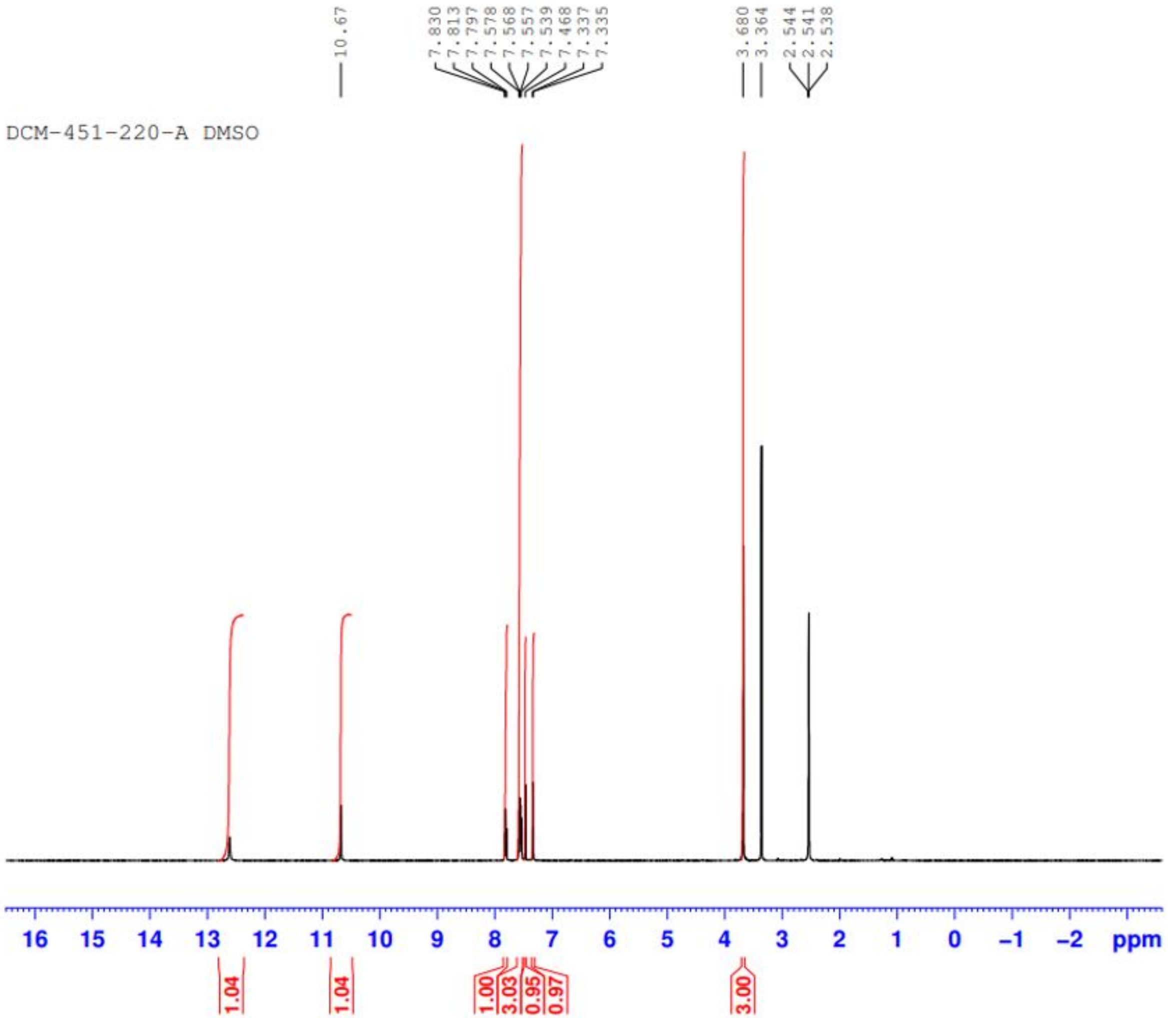

Figure S41: ${}^{13}C$ NMR spectrum of compound **34e**

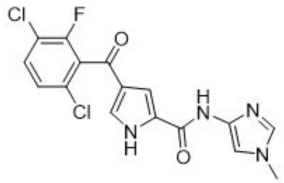

34e

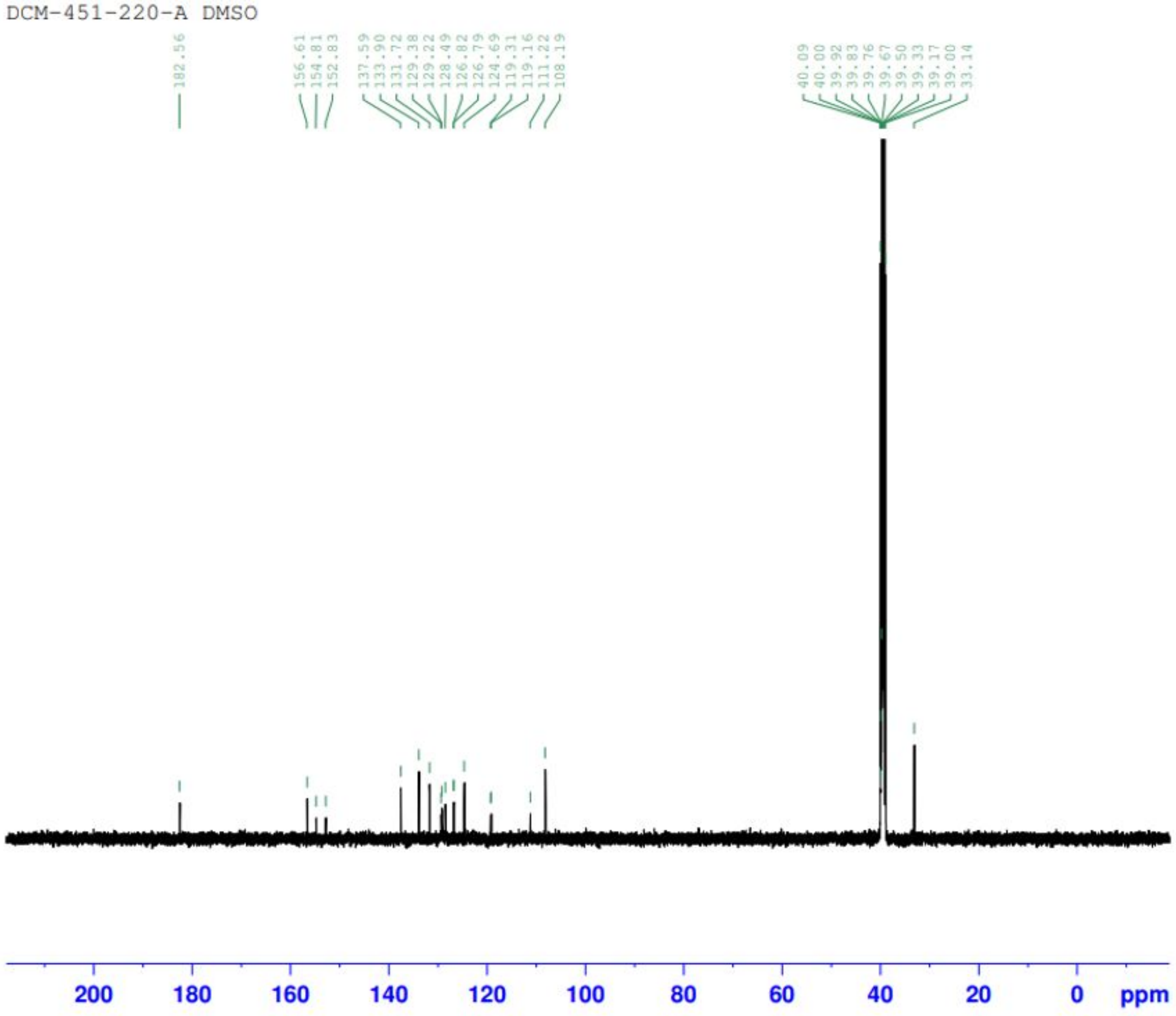

Figure S42: ^{1}H NMR spectrum of compound **34h**

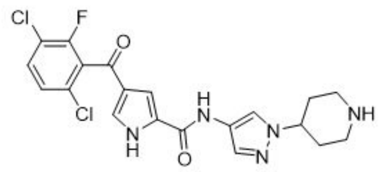

34h

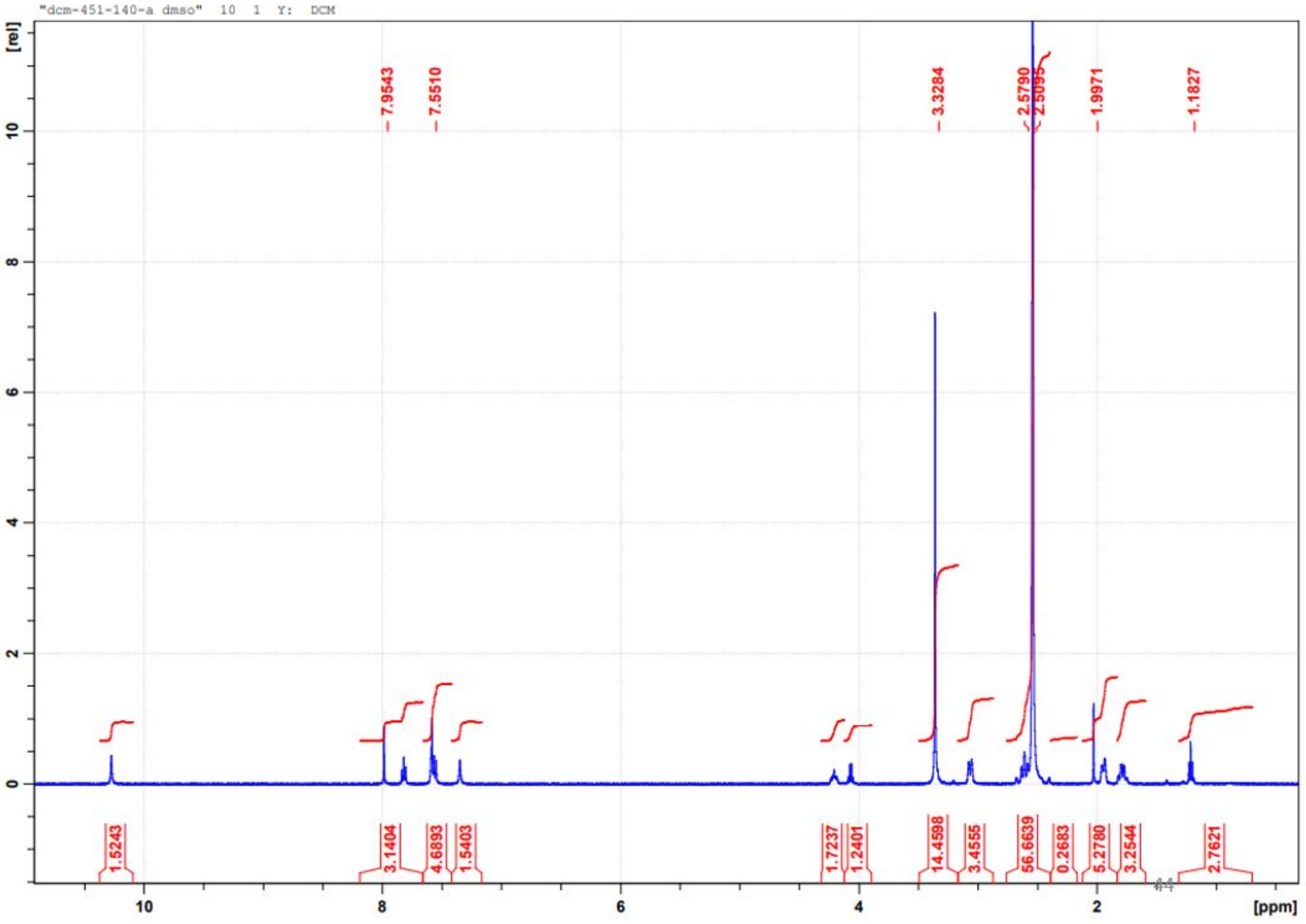

Figure S43: ^{13}C NMR spectrum of compound **34h**

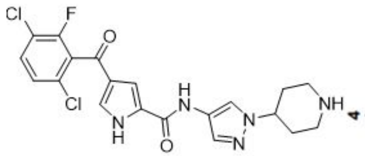

34h

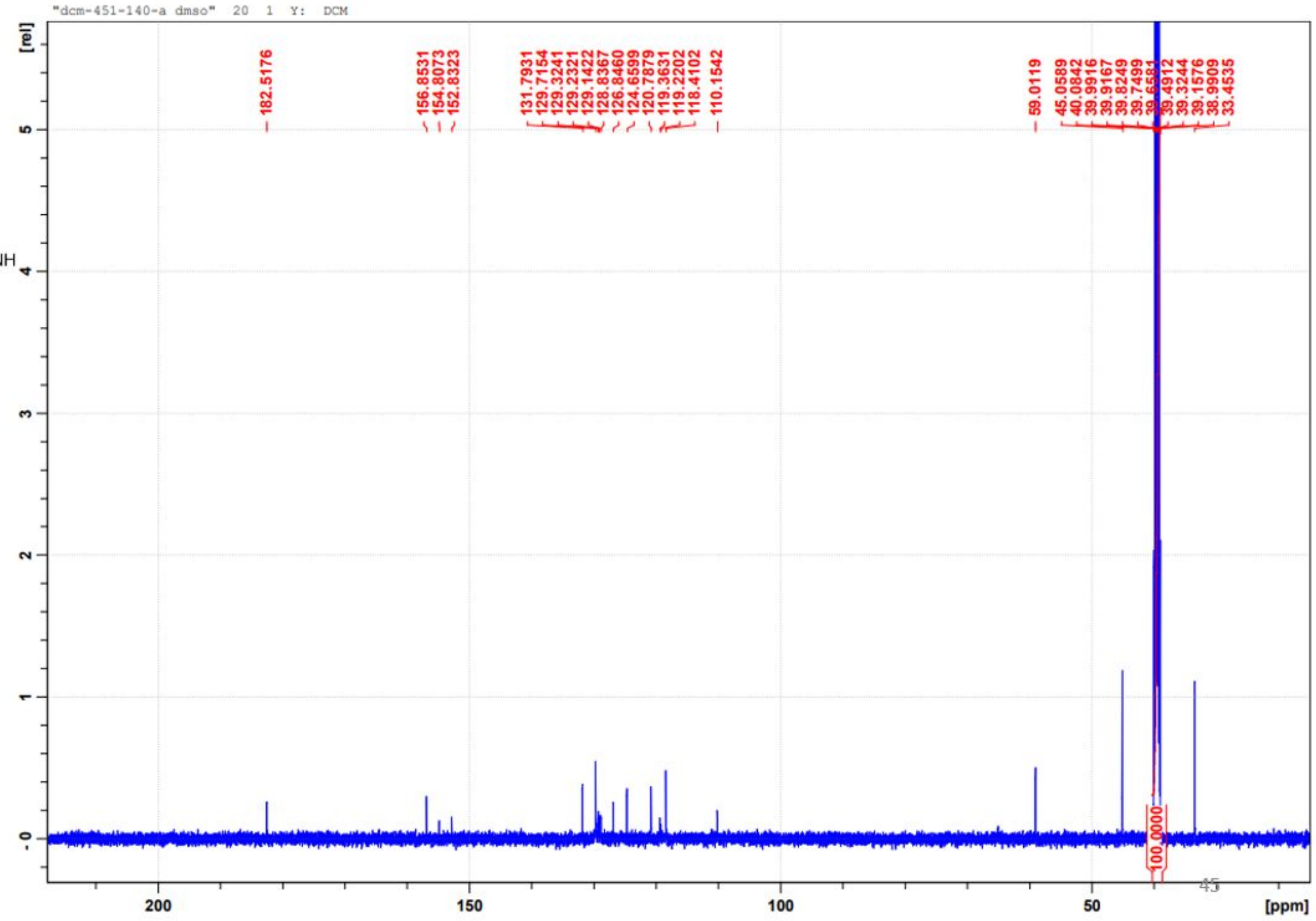

Figure S44: HPLC purity assessment of compound **34b**

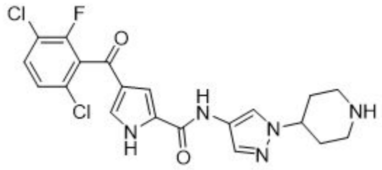

34h

```
Data File F:\09APR13_09.D
Sample Name: DCM/451/140/A

    =====================================================================
    Acq. Operator   : Karen Haggerty                  Seq. Line :   7
    Acq. Instrument : Instrument 1                     Location : Vial 3
    Injection Date  : 4/10/2013 6:15:20 PM                  Inj :   1
                                                    Inj Volume : 5.000 µl
    Different Inj Volume from Sequence !     Actual Inj Volume : 4.000 µl
    Acq. Method     : C:\CHEM32\1\DATA\APR2013\10APR13_HL_BJC_DCM_BZ_SA 2013-04-10 15-53-10\
                      XSELECT_ACIDIC.M
    Last changed    : 4/10/2013 3:53:18 PM by Karen Haggerty
    Analysis Method : F:\09APR13_09.D\DA.M (XSELECT_ACIDIC.M)
    Last changed    : 4/11/2013 8:00:45 AM by Karen Haggerty
                      (modified after loading)
    Method Info     : Waters XSELECT CSH C18 3.5um 100 x 4.6mm
                      0.1% Formic Acid (aq)/MeCN, 1.0mL/min
```

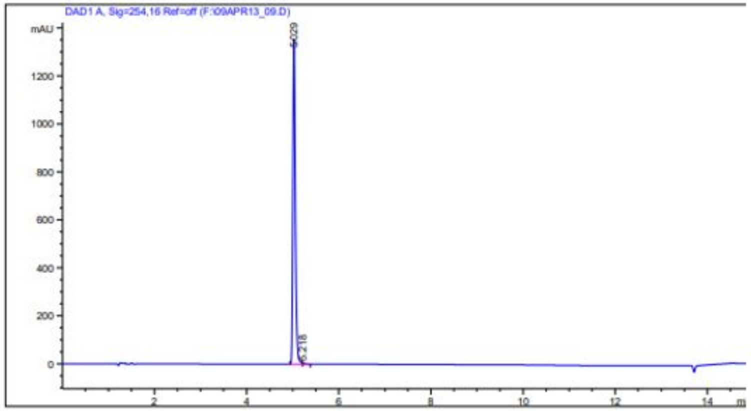


```
    =====================================================================
                           Area Percent Report
    =====================================================================

    Sorted By             :      Signal
    Multiplier:                  :       1.0000
    Dilution:                    :       1.0000
    Use Multiplier & Dilution Factor with ISTDs


    Signal 1: DAD1 A, Sig=254,16 Ref=off

    Peak RetTime Type  Width     Area      Height     Area
      #   [min]        [min]   [mAU*s]     [mAU]        %
    ----|-------|----|-------|----------|----------|--------|
       1   5.029 FM     0.0618 5047.23096 1361.69678 99.7055
       2   5.218 FM     0.0564   14.90839    4.40299   0.2945
```

Figure S45: ^{1}H NMR spectrum of compound **34i**

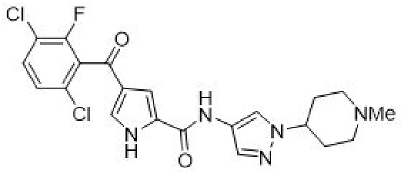

34i

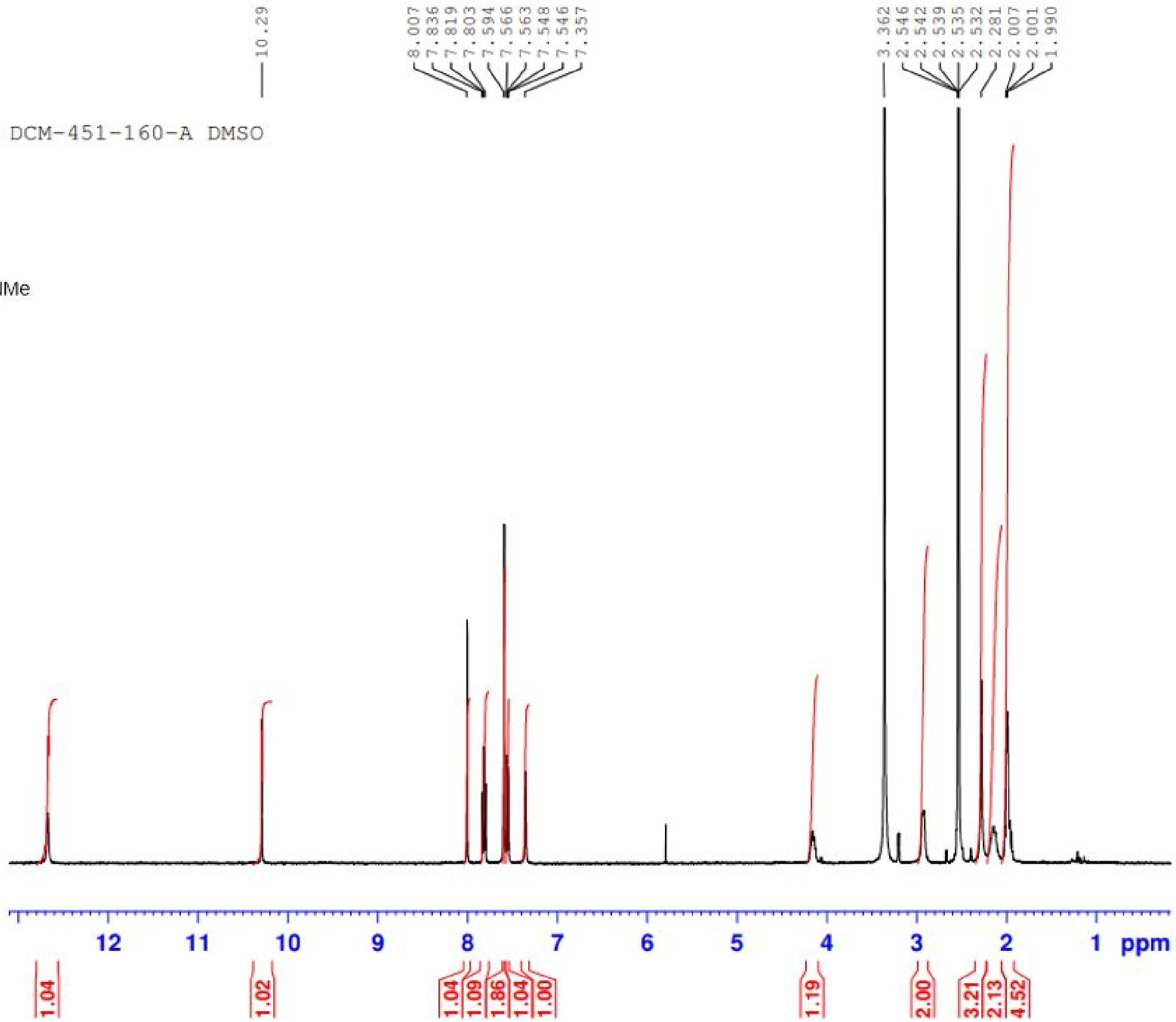

Figure S46: ^{13}C NMR spectrum of compound **34i**

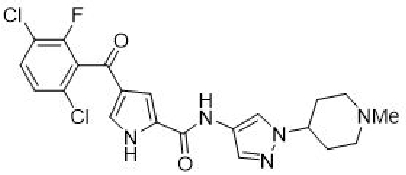

34i

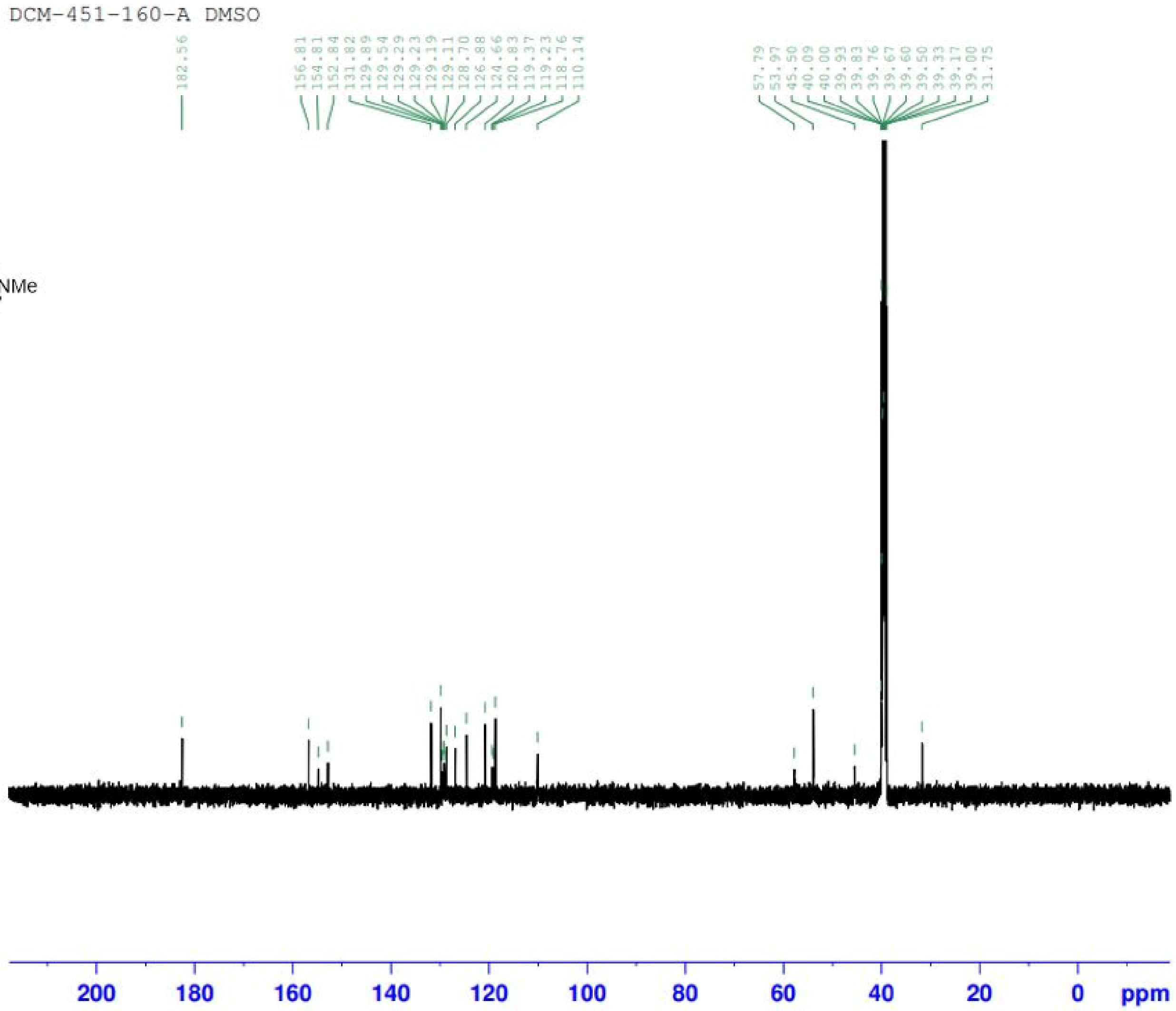

Figure S47: ^{1}H NMR spectrum of compound **34j**

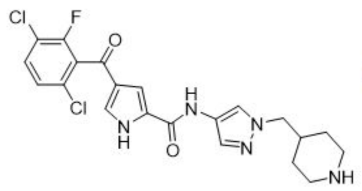

34j

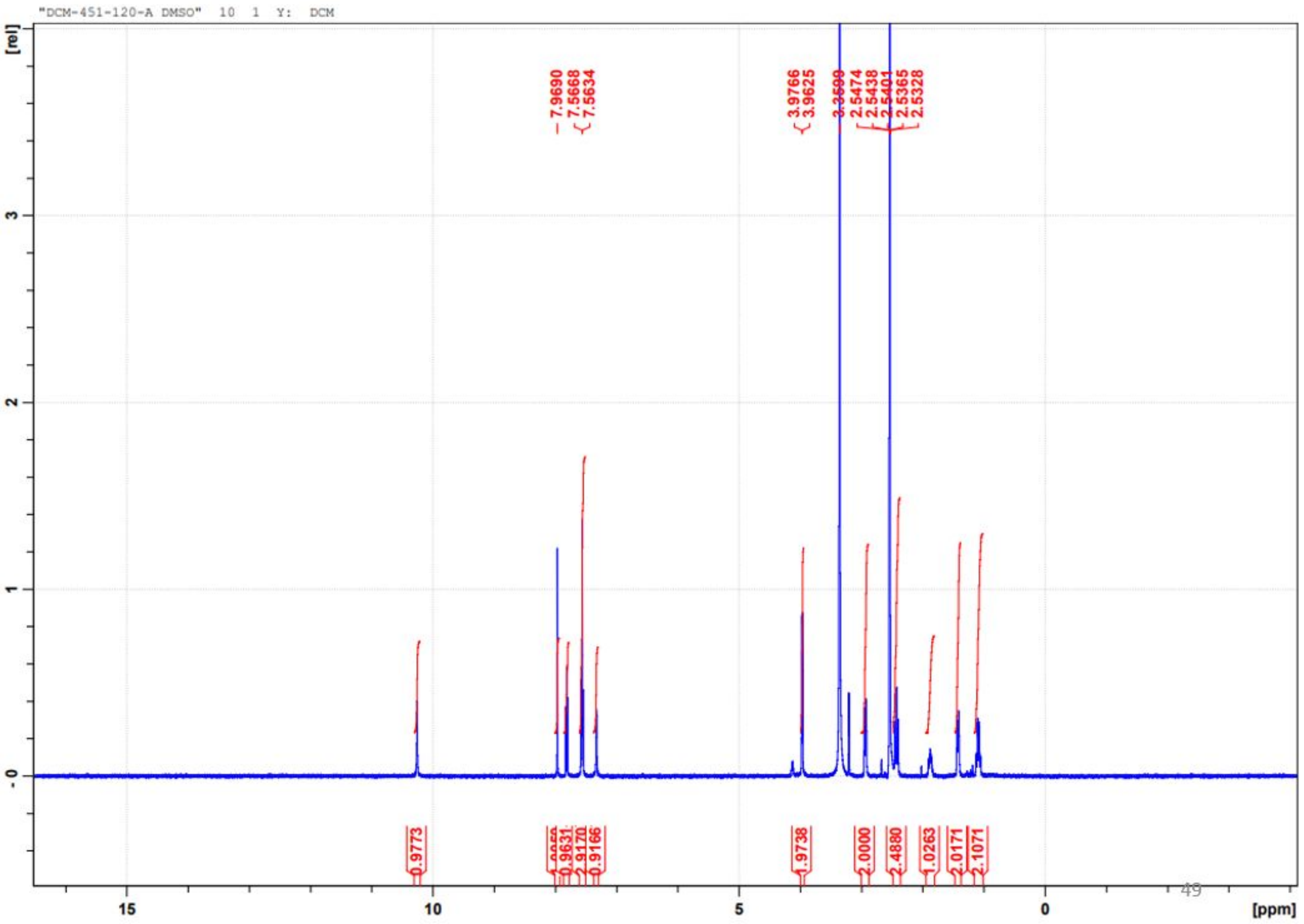

Figure S48: ^{13}C NMR spectrum of compound **34j**

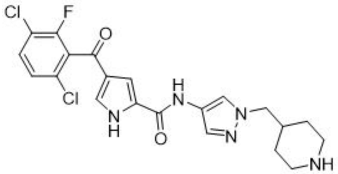

34j

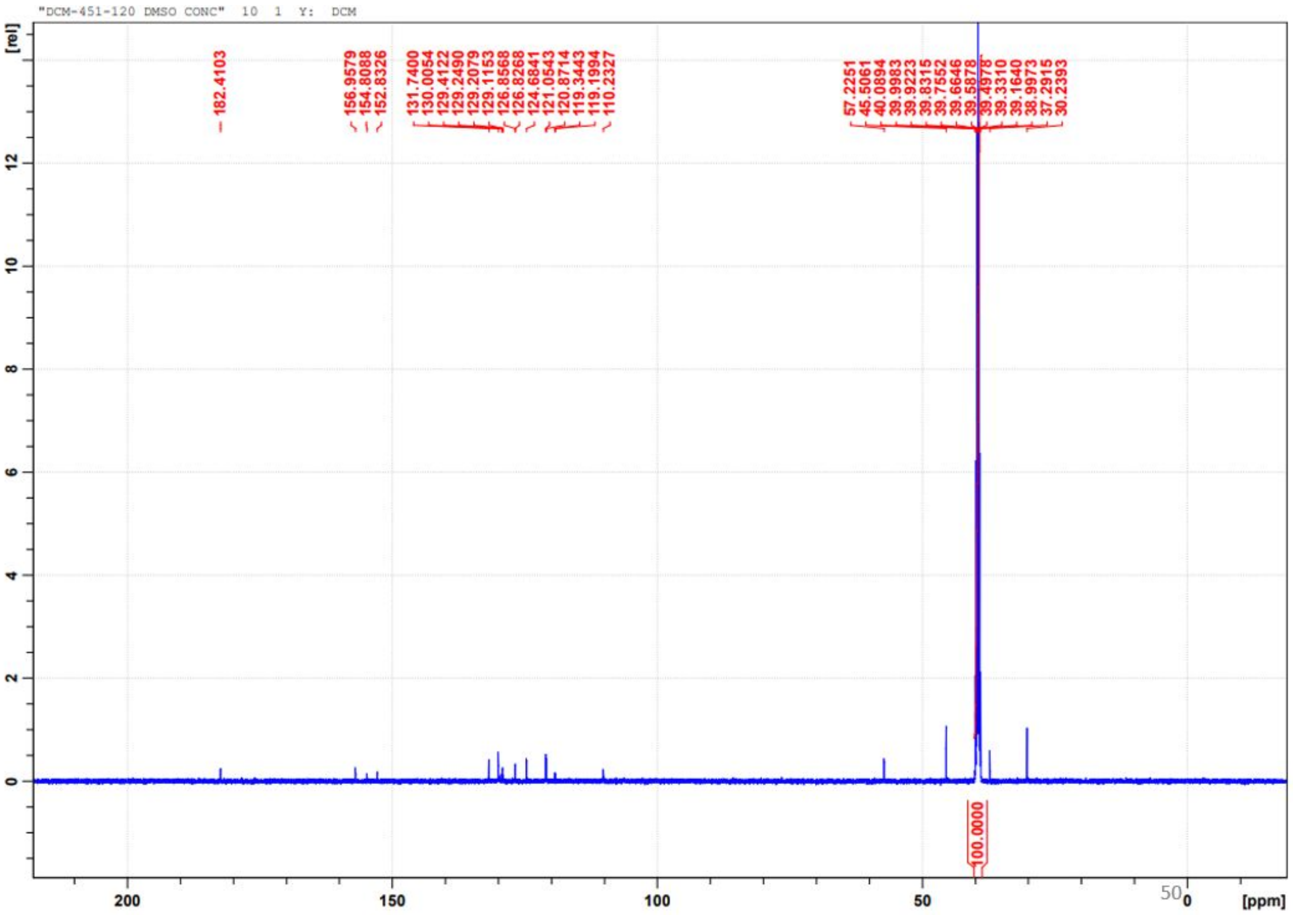

Figure S49: ^{1}H NMR spectrum of compound **34k**

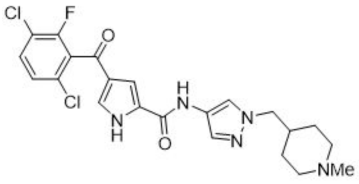

34k

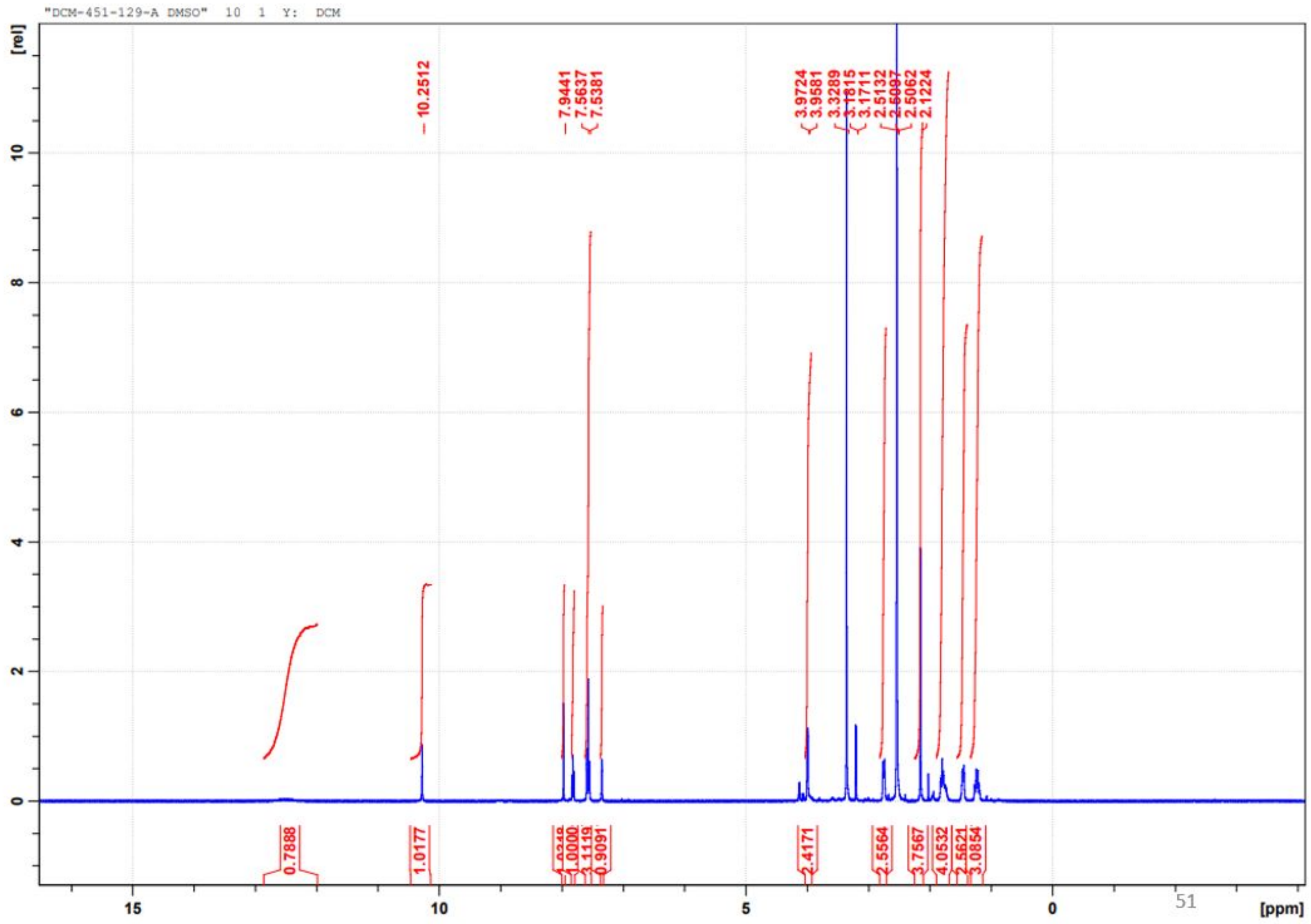

Table S1: Kinome Selectivity Data for Compound **34b**

Kinome panel selectivity screening was performed at DiscoverX using their kinomescan screen.

DiscoveRx Gene Symbol	Percent Control	Compound Concentration (nM)
AAK1	56	10000
ABL1(E255K)-phosphorylated	17	10000
ABL1(F317I)-nonphosphorylated	100	10000
ABL1(F317I)-phosphorylated	95	10000
ABL1(F317L)-nonphosphorylated	100	10000
ABL1(F317L)-phosphorylated	78	10000
ABL1(H396P)-nonphosphorylated	49	10000
ABL1(H396P)-phosphorylated	32	10000
ABL1(M351T)-phosphorylated	30	10000
ABL1(Q252H)-nonphosphorylated	25	10000
ABL1(Q252H)-phosphorylated	29	10000
ABL1(T315I)-nonphosphorylated	16	10000
ABL1(T315I)-phosphorylated	3.4	10000
ABL1(Y253F)-phosphorylated	29	10000
ABL1-nonphosphorylated	24	10000
ABL1-phosphorylated	34	10000
ABL2	70	10000
ACVR1	100	10000
ACVR1B	94	10000
ACVR2A	88	10000
ACVR2B	78	10000
ACVRL1	100	10000
ADCK3	100	10000
ADCK4	92	10000
AKT1	100	10000
AKT2	100	10000
AKT3	100	10000
ALK	13	10000
AMPK-alpha1	13	10000
AMPK-alpha2	11	10000
ANKK1	63	10000
ARK5	64	10000
ASK1	94	10000
ASK2	100	10000
AURKA	1	10000
AURKB	15	10000
AURKC	9	10000
AXL	6.8	10000
BIKE	88	10000
BLK	9	10000
BMPR1A	99	10000

BMPR1B	67	10000
BMPR2	82	10000
BMX	100	10000
BRAF	85	10000
BRAF(V600E)	86	10000
BRK	100	10000
BRSK1	13	10000
BRSK2	6.2	10000
BTK	100	10000
BUB1	22	10000
CAMK1	100	10000
CAMK1D	100	10000
CAMK1G	66	10000
CAMK2A	14	10000
CAMK2B	14	10000
CAMK2D	13	10000
CAMK2G	24	10000
CAMK4	100	10000
CAMKK1	57	10000
CAMKK2	50	10000
CASK	23	10000
CDC2L1	86	10000
CDC2L2	94	10000
CDC2L5	96	10000
CDK11	100	10000
CDK2	69	10000
CDK3	93	10000
CDK4-cyclinD1	89	10000
CDK4-cyclinD3	100	10000
CDK5	100	10000
CDK7	16	10000
CDK8	98	10000
CDK9	83	10000
CDKL1	79	10000
CDKL2	100	10000
CDKL3	100	10000
CDKL5	100	10000
CHEK1	8	10000
CHEK2	45	10000
CIT	100	10000
CLK1	100	10000
CLK2	22	10000
CLK3	57	10000
CLK4	100	10000
CSF1R	0.3	10000
CSF1R-autoinhibited	12	10000
CSK	83	10000
CSNK1A1	100	10000

CSNK1A1L	100	10000
CSNK1D	84	10000
CSNK1E	59	10000
CSNK1G1	78	10000
CSNK1G2	100	10000
CSNK1G3	100	10000
CSNK2A1	74	10000
CSNK2A2	100	10000
CTK	100	10000
DAPK1	79	10000
DAPK2	47	10000
DAPK3	56	10000
DCAMKL1	2.4	10000
DCAMKL2	3.6	10000
DCAMKL3	0.35	10000
DDR1	0.45	10000
DDR2	31	10000
DLK	100	10000
DMPK	60	10000
DMPK2	100	10000
DRAK1	49	10000
DRAK2	72	10000
DYRK1A	91	10000
DYRK1B	66	10000
DYRK2	91	10000
EGFR	66	10000
EGFR(E746-A750del)	97	10000
EGFR(G719C)	100	10000
EGFR(G719S)	100	10000
EGFR(L747-E749del, A750P)	78	10000
EGFR(L747-S752del, P753S)	88	10000
EGFR(L747-T751del,Sins)	97	10000
EGFR(L858R)	84	10000
EGFR(L858R,T790M)	96	10000
EGFR(L861Q)	100	10000
EGFR(S752-I759del)	100	10000
EGFR(T790M)	100	10000
EIF2AK1	91	10000
EPHA1	100	10000
EPHA2	100	10000
EPHA3	42	10000
EPHA4	100	10000
EPHA5	100	10000
EPHA6	100	10000
EPHA7	100	10000
EPHA8	94	10000
EPHB1	99	10000
EPHB2	71	10000

EPHB3	100	10000
EPHB4	100	10000
EPHB6	67	10000
ERBB2	86	10000
ERBB3	100	10000
ERBB4	100	10000
ERK1	100	10000
ERK2	100	10000
ERK3	100	10000
ERK4	73	10000
ERK5	0.3	10000
ERK8	100	10000
ERN1	43	10000
FAK	76	10000
FER	100	10000
FES	100	10000
FGFR1	4.6	10000
FGFR2	7.2	10000
FGFR3	8.5	10000
FGFR3(G697C)	9.2	10000
FGFR4	51	10000
FGR	55	10000
FLT1	19	10000
FLT3	90	10000
FLT3(D835H)	77	10000
FLT3(D835Y)	77	10000
FLT3(ITD)	91	10000
FLT3(K663Q)	93	10000
FLT3(N841I)	50	10000
FLT3(R834Q)	88	10000
FLT3-autoinhibited	100	10000
FLT4	65	10000
FRK	100	10000
FYN	100	10000
GAK	59	10000
GCN2(Kin.Dom.2,S808G)	37	10000
GRK1	100	10000
GRK4	78	10000
GRK7	66	10000
GSK3A	100	10000
GSK3B	100	10000
HASPIN	81	10000
HCK	39	10000
HIPK1	77	10000
HIPK2	99	10000
HIPK3	95	10000
HIPK4	76	10000
HPK1	15	10000

HUNK	100	10000
ICK	91	10000
IGF1R	100	10000
IKK-alpha	99	10000
IKK-beta	95	10000
IKK-epsilon	70	10000
INSR	83	10000
INSRR	95	10000
IRAK1	3.2	10000
IRAK3	66	10000
IRAK4	58	10000
ITK	90	10000
JAK1(JH1domain-catalytic)	6.8	10000
JAK1(JH2domain-pseudokinase)	19	10000
JAK2(JH1domain-catalytic)	0.05	10000
JAK3(JH1domain-catalytic)	0	10000
JNK1	86	10000
JNK2	93	10000
JNK3	88	10000
KIT	0.3	10000
KIT-autoinhibited	76	10000
LATS1	97	10000
LATS2	100	10000
LCK	29	10000
LIMK1	95	10000
LIMK2	100	10000
LKB1	74	10000
LOK	0.55	10000
LRRK2	3.2	10000
LTK	49	10000
LYN	53	10000
LZK	81	10000
MAK	94	10000
MAP3K1	66	10000
MAP3K15	61	10000
MAP3K2	0.3	10000
MAP3K3	0.8	10000
MAP3K4	100	10000
MAP4K2	5.8	10000
MAP4K3	32	10000
MAP4K4	78	10000
MAP4K5	41	10000
MAPKAPK2	99	10000
MAPKAPK5	92	10000
MARK1	54	10000
MARK2	32	10000
MARK3	13	10000
MARK4	62	10000

MAST1	49	10000
MEK1	85	10000
MEK2	72	10000
MEK3	80	10000
MEK4	100	10000
MEK5	18	10000
MEK6	100	10000
MELK	43	10000
MERTK	3.6	10000
MET	18	10000
MET(M1250T)	10	10000
MET(Y1235D)	51	10000
MINK	35	10000
MKK7	84	10000
MKNK1	98	10000
MKNK2	93	10000
MLCK	100	10000
MLK1	91	10000
MLK2	83	10000
MLK3	100	10000
MRCKA	100	10000
MRCKB	100	10000
MST1	53	10000
MST1R	53	10000
MST2	2.8	10000
MST3	27	10000
MST4	38	10000
MTOR	97	10000
MUSK	95	10000
MYLK	29	10000
MYLK2	93	10000
MYLK4	91	10000
MYO3A	97	10000
MYO3B	93	10000
NDR1	85	10000
NDR2	99	10000
NEK1	79	10000
NEK10	86	10000
NEK11	94	10000
NEK2	9	10000
NEK3	93	10000
NEK4	76	10000
NEK5	90	10000
NEK6	100	10000
NEK7	100	10000
NEK9	100	10000
NIK	100	10000
NIM1	100	10000

NLK	100	10000
OSR1	100	10000
p38-alpha	100	10000
p38-beta	100	10000
p38-delta	97	10000
p38-gamma	100	10000
PAK1	100	10000
PAK2	55	10000
PAK3	12	10000
PAK4	63	10000
PAK6	68	10000
PAK7	45	10000
PCTK1	92	10000
PCTK2	97	10000
PCTK3	94	10000
PDGFRA	18	10000
PDGFRB	3	10000
PDPK1	30	10000
PFCDPK1(P.falciparum)	67	10000
PFPK5(P.falciparum)	94	10000
PFTAIRE2	100	10000
PFTK1	81	10000
PHKG1	92	10000
PHKG2	46	10000
PIK3C2B	100	10000
PIK3C2G	99	10000
PIK3CA	100	10000
PIK3CA(C420R)	86	10000
PIK3CA(E542K)	93	10000
PIK3CA(E545A)	55	10000
PIK3CA(E545K)	80	10000
PIK3CA(H1047L)	86	10000
PIK3CA(H1047Y)	46	10000
PIK3CA(I800L)	100	10000
PIK3CA(M1043I)	100	10000
PIK3CA(Q546K)	86	10000
PIK3CB	92	10000
PIK3CD	100	10000
PIK3CG	100	10000
PIK4CB	100	10000
PIM1	94	10000
PIM2	100	10000
PIM3	92	10000
PIP5K1A	78	10000
PIP5K1C	49	10000
PIP5K2B	92	10000
PIP5K2C	100	10000
PKAC-alpha	100	10000

PKAC-beta	100	10000
PKMYT1	94	10000
PKN1	43	10000
PKN2	46	10000
PKNB(M.tuberculosis)	72	10000
PLK1	65	10000
PLK2	1.8	10000
PLK3	66	10000
PLK4	29	10000
PRKCD	99	10000
PRKCE	100	10000
PRKCH	100	10000
PRKCI	90	10000
PRKCQ	99	10000
PRKD1	32	10000
PRKD2	38	10000
PRKD3	58	10000
PRKG1	61	10000
PRKG2	85	10000
PRKR	80	10000
PRKX	86	10000
PRP4	100	10000
PYK2	62	10000
QSK	36	10000
RAF1	100	10000
RET	33	10000
RET(M918T)	18	10000
RET(V804L)	70	10000
RET(V804M)	91	10000
RIOK1	88	10000
RIOK2	81	10000
RIOK3	99	10000
RIPK1	100	10000
RIPK2	100	10000
RIPK4	94	10000
RIPK5	72	10000
ROCK1	67	10000
ROCK2	72	10000
ROS1	100	10000
RPS6KA4(Kin.Dom.1-N-terminal)	95	10000
RPS6KA4(Kin.Dom.2-C-terminal)	81	10000
RPS6KA5(Kin.Dom.1-N-terminal)	100	10000
RPS6KA5(Kin.Dom.2-C-terminal)	100	10000
RSK1(Kin.Dom.1-N-terminal)	50	10000
RSK1(Kin.Dom.2-C-terminal)	26	10000
RSK2(Kin.Dom.1-N-terminal)	91	10000
RSK2(Kin.Dom.2-C-terminal)	30	10000
RSK3(Kin.Dom.1-N-terminal)	43	10000

RSK3(Kin.Dom.2-C-terminal)	2.9	10000
RSK4(Kin.Dom.1-N-terminal)	92	10000
RSK4(Kin.Dom.2-C-terminal)	9.6	10000
S6K1	39	10000
SBK1	34	10000
SGK	92	10000
SgK110	53	10000
SGK2	100	10000
SGK3	95	10000
SIK	76	10000
SIK2	52	10000
SLK	0.8	10000
SNARK	33	10000
SNRK	94	10000
SRC	100	10000
SRMS	92	10000
SRPK1	73	10000
SRPK2	89	10000
SRPK3	88	10000
STK16	77	10000
STK33	69	10000
STK35	100	10000
STK36	100	10000
STK39	100	10000
SYK	100	10000
TAK1	99	10000
TAOK1	64	10000
TAOK2	5.7	10000
TAOK3	54	10000
TBK1	72	10000
TEC	100	10000
TESK1	21	10000
TGFBR1	100	10000
TGFBR2	100	10000
TIE1	20	10000
TIE2	33	10000
TLK1	77	10000
TLK2	96	10000
TNIK	33	10000
TNK1	70	10000
TNK2	96	10000
TNNI3K	92	10000
TRKA	30	10000
TRKB	18	10000
TRKC	35	10000
TRPM6	79	10000
TSSK1B	77	10000
TTK	76	10000

TXK	100	10000
TYK2(JH1domain-catalytic)	0.05	10000
TYK2(JH2domain-pseudokinase)	12	10000
TYRO3	100	10000
ULK1	0.15	10000
ULK2	0.05	10000
ULK3	1.5	10000
VEGFR2	57	10000
VRK2	89	10000
WEE1	79	10000
WEE2	100	10000
WNK1	100	10000
WNK3	85	10000
YANK1	94	10000
YANK2	100	10000
YANK3	100	10000
YES	98	10000
YSK1	63	10000
YSK4	79	10000
ZAK	100	10000
ZAP70	100	10000

Table S2: Kinome Selectivity (Kd) Data for Compound **34b**

DiscoveRx Gene Symbol	Entrez Gene Symbol	Kd (nM)
ABL1-nonphosphorylated	ABL1	1200
AURKA	AURKA	290
CSF1R	CSF1R	46
DCAMKL1	DCLK1	61
ERK5	MAPK7	180
FGFR1	FGFR1	380
JAK3(JH1domain-catalytic)	JAK3	1300
KIT	KIT	420
LRRK2	LRRK2	220
MEK5	MAP2K5	2800

Figure S50: Cellular dependency on MAPK7 (RNAi gene silencing, DepMap)

a)

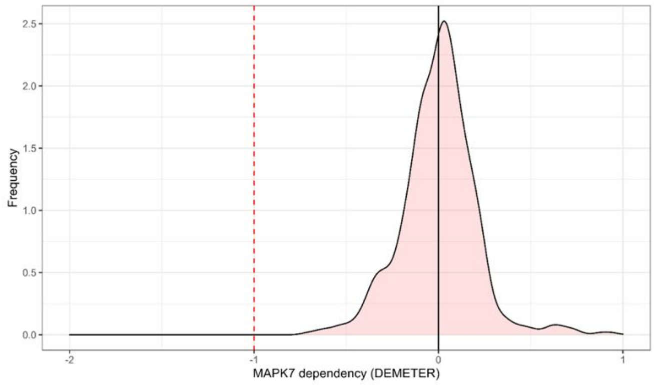

b)

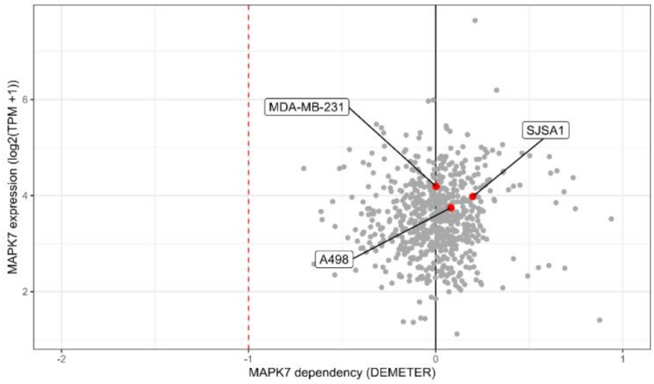

a) Distribution of DEMETER gene dependency scores (DepMap RNAi) for *MAPK7* across all cell lines. b) Scatterplot of DEMETER gene dependency score (DepMap RNAi) and RNA expression values (log2(TPM +1) for *MAPK7*.

Genetic dependency was assessed using combined RNA interference (RNAi) data from 660 unique tumour cell lines treated with shRNA to *MAPK7* (the gene encoding ERK5) where the effect on growth had been determined. A dependency score of zero or greater (observed with MDA-MB-231, A498, and MDA-MB-231 in response to *MAPK7* silencing) indicates that silencing of a given gene has not had an inhibitory effect on cell growth. A dependency score of − 1 is comparable to the median of all pan-essential genes (red dashed line). Data was downloaded from the DepMap Portal and processed through the DEMETER2 pipeline.[1] The mRNA expression values were taken from the DepMap 21Q4 release. All data are available at depmap.org/portal/.

A. Improved estimation of cancer dependencies from large-scale RNAi screens using model-based normalization and data integration. *Nat. Commun.* **2018,** 9, 4610.

Figure S51: Quantification of p-ERK5 by Western blotting and densitometry

a)

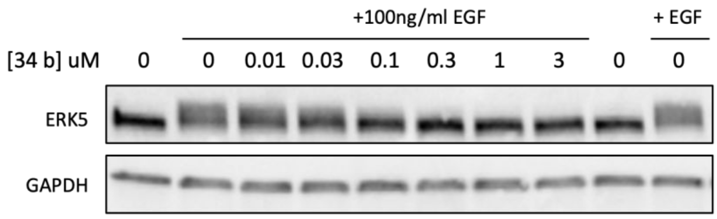

b)

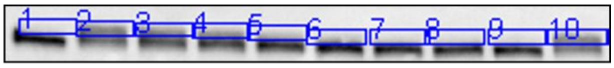

c)

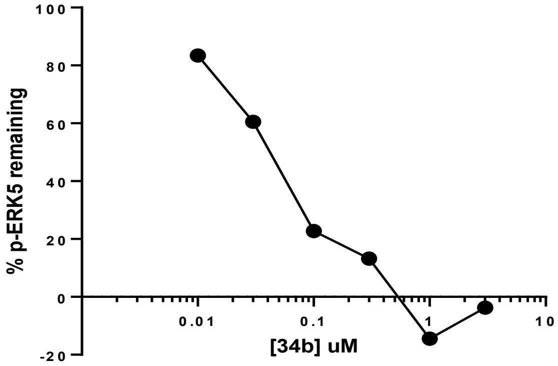

HeLa cells were serum starved overnight, treated with various concentrations of compound **34b** for 1h and then stimulated with EGF (100ng/ml) for 10 minutes. a) Western blot showing upper phospho-ERK5 band with EGF stimulation and inhibition by compound 34b, b) densitometry to quantitate upper band, c) densitometry values plotted as % p-ERK5 remaining (IC_{50} = 42nM).

Reference

[1] McFarland, J. M.; Ho, Z. V.; Kugener, G; Dempster, J. M.; Montgomery, P. G.; Bryan, J. G.; Krill-Burger, J. M., Green, T. M.; Vazquez, F; Boehm, J. S.; Golub, T. R.; Hahn, W. C.; Root, D. E.; Tsherniak,
