# Supplementary material for: Parallel Optimization of Potency and Pharmacokinetics Leading to the Discovery of a Pyrrole Carboxamide ERK5 Kinase Domain Inhibitor
Source: J Med Chem. 2022 Apr 25;65(9):6513–40. doi: 10.1021/acs.jmedchem.1c01756 (PMC9109144; doi:10.1021/acs.jmedchem.1c01756)
Supplement: Supplementary file 4 — jm1c01756_si_004.pdf [file jm1c01756_si_004.pdf]

Preliminary Full wwPDB X-ray Structure Validation Report (i)

Sep 30, 2021 – 01:42 pm BST

Deposition ID : D_1292118411

This is a Preliminary Full wwPDB X-ray Structure Validation Report.

This report is produced by the wwPDB Deposition System during initial deposition but before annotation of the structure.

We welcome your comments at *validation@mail.wwpdb.org*
A user guide is available at
https://www.wwpdb.org/validation/2017/XrayValidationReportHelp
with specific help available everywhere you see the (i) symbol.

PRELIMINARY VALIDATION REPORT

The following versions of software and data (see references (i)) were used in the production of this report:

MolProbity	:	4.02b-467
Mogul	:	1.8.5 (274361), CSD as541be (2020)
Xtriage (Phenix)	:	1.13
EDS	:	2.23.2
buster-report	:	1.1.7 (2018)
Percentile statistics	:	20191225.v01 (using entries in the PDB archive December 25th 2019)
Refmac	:	5.8.0267
CCP4	:	7.1.010 (Gargrove)
Ideal geometry (proteins)	:	Engh & Huber (2001)
Ideal geometry (DNA, RNA)	:	Parkinson et al. (1996)
Validation Pipeline (wwPDB-VP)	:	2.23.2

1 Overall quality at a glance (i)

The following experimental techniques were used to determine the structure:
X-RAY DIFFRACTION

The reported resolution of this entry is 2.59 Å.

Percentile scores (ranging between 0-100) for global validation metrics of the entry are shown in the following graphic. The table shows the number of entries on which the scores are based.

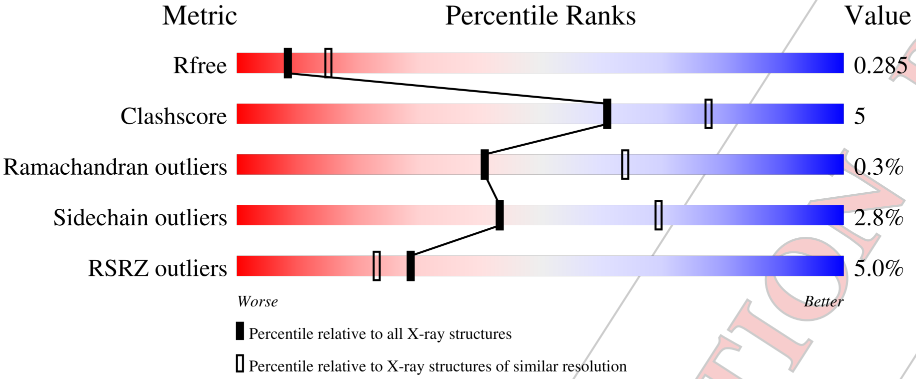

Metric	Whole archive (#Entries)	Similar resolution (#Entries, resolution range(Å))
R_{free}	130704	3163 (2.60-2.60)
Clashscore	141614	3518 (2.60-2.60)
Ramachandran outliers	138981	3455 (2.60-2.60)
Sidechain outliers	138945	3455 (2.60-2.60)
RSRZ outliers	127900	3104 (2.60-2.60)

The table below summarises the geometric issues observed across the polymeric chains and their fit to the electron density. The red, orange, yellow and green segments of the lower bar indicate the fraction of residues that contain outliers for >=3, 2, 1 and 0 types of geometric quality criteria respectively. A grey segment represents the fraction of residues that are not modelled. The numeric value for each fraction is indicated below the corresponding segment, with a dot representing fractions <=5% The upper red bar (where present) indicates the fraction of residues that have poor fit to the electron density. The numeric value is given above the bar.

Mol	Chain	Length	Quality of chain
1	AAA	358	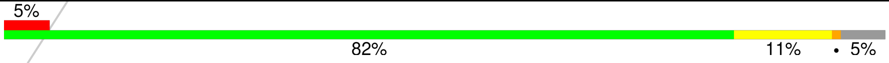

PRELIMINARY VALIDATION REPORT

2 Entry composition (i)

There are 3 unique types of molecules in this entry. The entry contains 2782 atoms, of which 0 are hydrogens and 0 are deuteriums.

In the tables below, the ZeroOcc column contains the number of atoms modelled with zero occupancy, the AltConf column contains the number of residues with at least one atom in alternate conformation and the Trace column contains the number of residues modelled with at most 2 atoms.

- Molecule 1 is a protein.

Mol	Chain	Residues	Atoms	ZeroOcc	AltConf	Trace
1	AAA	340	Total C N O S<br>2744 1757 492 483 12	0	3	0

- Molecule 2 is a ligand with the chemical component id DRG but there is no existing wwPDB Chemical Component Dictionary definition for DRG. Consequently no firm identification of ligand chemistry can be made. Once the structure is annotated then an identification and diagram will be given here.

Mol	Chain	Residues	Atoms	ZeroOcc	AltConf
2	AaA	1	Total C Cl F N O<br>25 16 2 1 4 2	0	0

- Molecule 3 is water.

Mol	Chain	Residues	Atoms	ZeroOcc	AltConf
3	CCC	13	Total O<br>13 13	0	0

PRELIMINARY VALIDATION REPORT

3 Residue-property plots (i)

These plots are drawn for all protein, RNA, DNA and oligosaccharide chains in the entry. The first graphic for a chain summarises the proportions of the various outlier classes displayed in the second graphic. The second graphic shows the sequence view annotated by issues in geometry and electron density. Residues are color-coded according to the number of geometric quality criteria for which they contain at least one outlier: green = 0, yellow = 1, orange = 2 and red = 3 or more. A red dot above a residue indicates a poor fit to the electron density (RSRZ > 2). Stretches of 2 or more consecutive residues without any outlier are shown as a green connector. Residues present in the sample, but not in the model, are shown in grey.

- Molecule 1:

Chain AAA:

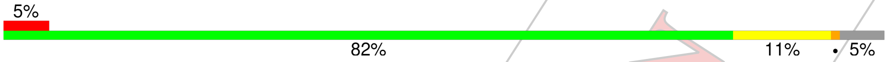

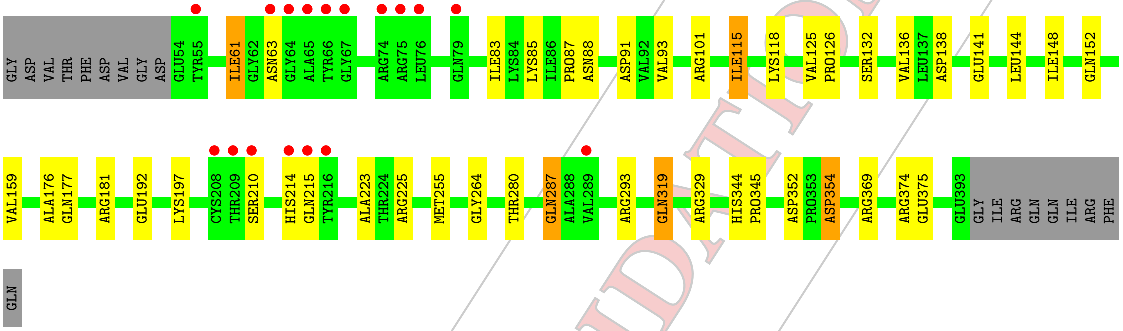

PRELIMINARY VALIDATION REPORT

4 Data and refinement statistics (i)

Property	Value	Source
Space group	P 41 21 2	Depositor
Cell constants<br>a, b, c, α, β, γ	91.67Å 91.67Å 113.81Å<br>90.00° 90.00° 90.00°	Depositor
Resolution (Å)	71.49 – 2.59<br>71.39 – 2.59	Depositor<br>EDS
% Data completeness<br>(in resolution range)	100.0 (71.49-2.59)<br>100.0 (71.39-2.59)	Depositor<br>EDS
R_{merge}	0.37	Depositor
R_{sym}	(Not available)	Depositor
$< I/\sigma(I) >$ [1]	1.82 (at 2.58Å)	Xtriage
Refinement program	refmac 5.8.0258, refmac 5.8.0258	Depositor
R, R_{free}	0.219 , 0.285<br>0.219 , 0.285	Depositor<br>DCC
R_{free} test set	790 reflections (5.03%)	wwPDB-VP
Wilson B-factor (Å^2)	52.8	Xtriage
Anisotropy	0.399	Xtriage
Bulk solvent k_{sol}(e/Å^3), B_{sol}(Å^2)	(Not available) , (Not available)	EDS
L-test for twinning[2]	$< \|L\| > = 0.49$, $< L^2 > = 0.32$	Xtriage
Estimated twinning fraction	No twinning to report.	Xtriage
F_o,F_c correlation	0.94	EDS
Total number of atoms	2782	wwPDB-VP
Average B, all atoms (Å^2)	60.0	wwPDB-VP

Xtriage's analysis on translational NCS is as follows: *The largest off-origin peak in the Patterson function is 4.49% of the height of the origin peak. No significant pseudotranslation is detected.*

[1]Intensities estimated from amplitudes.
[2]Theoretical values of $< |L| >$, $< L^2 >$ for acentric reflections are 0.5, 0.333 respectively for untwinned datasets, and 0.375, 0.2 for perfectly twinned datasets.

PRELIMINARY VALIDATION REPORT

5 Model quality (i)

5.1 Standard geometry (i)

Bond lengths and bond angles in the following residue types are not validated in this section: DRG

The Z score for a bond length (or angle) is the number of standard deviations the observed value is removed from the expected value. A bond length (or angle) with $|Z| > 5$ is considered an outlier worth inspection. RMSZ is the root-mean-square of all Z scores of the bond lengths (or angles).

Mol	Chain	Bond lengths		Bond angles	
		RMSZ	#|Z| >5	RMSZ	#|Z| >5
1	AAA	0.45	0/2812	0.86	3/3817 (0.1%)

There are no bond length outliers.

All (3) bond angle outliers are listed below:

Mol	Chain	Res	Type	Atoms	Z	Observed(°)	Ideal(°)
1	AAA	101	ARG	NE-CZ-NH1	5.18	122.89	120.30
1	AAA	375	GLU	CB-CA-C	5.12	120.64	110.40
1	AAA	152	GLN	CB-CA-C	-5.12	100.17	110.40

There are no chirality outliers.

There are no planarity outliers.

5.2 Too-close contacts (i)

In the following table, the Non-H and H(model) columns list the number of non-hydrogen atoms and hydrogen atoms in the chain respectively. The H(added) column lists the number of hydrogen atoms added and optimized by MolProbity. The Clashes column lists the number of clashes within the asymmetric unit, whereas Symm-Clashes lists symmetry-related clashes.

Mol	Chain	Non-H	H(model)	H(added)	Clashes	Symm-Clashes
1	AAA	2744	0	2729	25	0
2	AaA	25	0	0	0	0
3	CCC	13	0	0	0	0
All	All	2782	0	2729	25	0

The all-atom clashscore is defined as the number of clashes found per 1000 atoms (including hydrogen atoms). The all-atom clashscore for this structure is 5.

PRELIMINARY VALIDATION REPORT

All (25) close contacts within the same asymmetric unit are listed below, sorted by their clash magnitude.

Atom-1	Atom-2	Interatomic distance (Å)	Clash overlap (Å)
1:AAA:319:GLN:HE21	1:AAA:319:GLN:HA	1.43	0.82
1:AAA:61:ILE:C	1:AAA:61:ILE:HD13	2.16	0.65
1:AAA:141:GLU:OE1	1:AAA:192:GLU:HG3	1.96	0.65
1:AAA:87:PRO:HA	1:AAA:132:SER:HB3	1.84	0.59
1:AAA:118:LYS:HE3	1:AAA:138:ASP:OD1	2.03	0.58
1:AAA:352:ASP:C	1:AAA:354:ASP:H	2.10	0.55
1:AAA:210:SER:HB2	1:AAA:369:ARG:HG2	1.89	0.54
1:AAA:181:ARG:O	1:AAA:223:ALA:HB2	2.08	0.53
1:AAA:225:ARG:NH2	1:AAA:264:GLY:O	2.42	0.48
1:AAA:115:ILE:HD12	1:AAA:197:LYS:HB2	1.96	0.47
1:AAA:352:ASP:C	1:AAA:354:ASP:N	2.66	0.47
1:AAA:159:VAL:HG22	1:AAA:255:MET:HB3	1.96	0.46
1:AAA:144:LEU:O	1:AAA:148:ILE:HG13	2.16	0.46
1:AAA:93:VAL:HG22	1:AAA:374:ARG:HG3	1.98	0.46
1:AAA:88:ASN:HB3	1:AAA:91:ASP:OD2	2.16	0.45
1:AAA:176:ALA:O	1:AAA:177:GLN:HB2	2.16	0.45
1:AAA:144:LEU:HD12	1:AAA:144:LEU:HA	1.84	0.45
1:AAA:87:PRO:HA	1:AAA:132:SER:CB	2.46	0.43
1:AAA:214:HIS:NE2	1:AAA:215[A]:GLN:HG3	2.34	0.43
1:AAA:344:HIS:CG	1:AAA:345:PRO:HD2	2.54	0.43
1:AAA:319:GLN:HA	1:AAA:319:GLN:NE2	2.21	0.43
1:AAA:125:VAL:HB	1:AAA:126:PRO:HD2	2.02	0.42
1:AAA:83:ILE:HG12	1:AAA:136:VAL:HG22	2.03	0.41
1:AAA:85:LYS:O	1:AAA:87:PRO:HD3	2.21	0.40
1:AAA:287:GLN:HE21	1:AAA:287:GLN:HB3	1.71	0.40

There are no symmetry-related clashes.

5.3 Torsion angles (i)

5.3.1 Protein backbone (i)

In the following table, the Percentiles column shows the percent Ramachandran outliers of the chain as a percentile score with respect to all X-ray entries followed by that with respect to entries of similar resolution.

The Analysed column shows the number of residues for which the backbone conformation was analysed, and the total number of residues.

PRELIMINARY VALIDATION REPORT

Mol	Chain	Analysed	Favoured	Allowed	Outliers	Percentiles	
1	AAA	341/358 (95%)	327 (96%)	13 (4%)	1 (0%)	41	64

All (1) Ramachandran outliers are listed below:

Mol	Chain	Res	Type
1	AAA	63	ASN

5.3.2 Protein sidechains (i)

In the following table, the Percentiles column shows the percent sidechain outliers of the chain as a percentile score with respect to all X-ray entries followed by that with respect to entries of similar resolution.

The Analysed column shows the number of residues for which the sidechain conformation was analysed, and the total number of residues.

Mol	Chain	Analysed	Rotameric	Outliers	Percentiles	
1	AAA	287/308 (93%)	278 (97%)	9 (3%)	40	66

All (9) residues with a non-rotameric sidechain are listed below:

Mol	Chain	Res	Type
1	AAA	61	ILE
1	AAA	115	ILE
1	AAA	280	THR
1	AAA	287	GLN
1	AAA	293	ARG
1	AAA	319	GLN
1	AAA	329[A]	ARG
1	AAA	329[B]	ARG
1	AAA	354	ASP

Sometimes sidechains can be flipped to improve hydrogen bonding and reduce clashes. There are no such sidechains identified.

5.3.3 RNA (i)

There are no RNA molecules in this entry.

PRELIMINARY VALIDATION REPORT

5.4 Non-standard residues in protein, DNA, RNA chains (i)

There are no non-standard protein/DNA/RNA residues in this entry.

5.5 Carbohydrates (i)

There are no monosaccharides in this entry.

5.6 Ligand geometry (i)

Of 1 ligands modelled in this entry, 1 could not be matched to an existing wwPDB Chemical Component Dictionary definition at this stage - leaving 0 for Mogul analysis.

There are no bond length outliers.

There are no bond angle outliers.

There are no chirality outliers.

There are no torsion outliers.

There are no ring outliers.

No monomer is involved in short contacts.

5.7 Other polymers (i)

There are no such residues in this entry.

5.8 Polymer linkage issues (i)

There are no chain breaks in this entry.

PRELIMINARY VALIDATION REPORT

6 Fit of model and data (i)

6.1 Protein, DNA and RNA chains (i)

In the following table, the column labelled '#RSRZ> 2' contains the number (and percentage) of RSRZ outliers, followed by percent RSRZ outliers for the chain as percentile scores relative to all X-ray entries and entries of similar resolution. The OWAB column contains the minimum, median, 95th percentile and maximum values of the occupancy-weighted average B-factor per residue. The column labelled 'Q< 0.9' lists the number of (and percentage) of residues with an average occupancy less than 0.9.

Mol	Chain	Analysed	<RSRZ>	#RSRZ>2			OWAB(Å^2)	Q<0.9
1	AAA	340/358 (94%)	0.57	17 (5%)	28	23	31, 54, 114, 150	0

All (17) RSRZ outliers are listed below:

Mol	Chain	Res	Type	RSRZ
1	AAA	63	ASN	6.0
1	AAA	66	TYR	5.5
1	AAA	214	HIS	4.6
1	AAA	208	CYS	3.9
1	AAA	76	LEU	3.7
1	AAA	209	THR	3.7
1	AAA	75	ARG	3.1
1	AAA	64	GLY	2.9
1	AAA	74	ARG	2.7
1	AAA	55	TYR	2.5
1	AAA	67	GLY	2.4
1	AAA	215[A]	GLN	2.3
1	AAA	65	ALA	2.3
1	AAA	216	TYR	2.3
1	AAA	79	GLN	2.2
1	AAA	289	VAL	2.2
1	AAA	210	SER	2.1

6.2 Non-standard residues in protein, DNA, RNA chains (i)

There are no non-standard protein/DNA/RNA residues in this entry.

6.3 Carbohydrates (i)

There are no monosaccharides in this entry.

PRELIMINARY VALIDATION REPORT

6.4 Ligands (i)

In the following table, the Atoms column lists the number of modelled atoms in the group and the number defined in the chemical component dictionary. The B-factors column lists the minimum, median, 95th percentile and maximum values of B factors of atoms in the group. The column labelled 'Q< 0.9' lists the number of atoms with occupancy less than 0.9.

Mol	Type	Chain	Res	Atoms	RSCC	RSR	B-factors($Å^2$)	Q<0.9
2	DRG	AaA	601	25/?	0.88	0.27	64,92,143,148	0

The following is a graphical depiction of the model fit to experimental electron density of all instances of the Ligand of Interest. In addition, ligands with molecular weight > 250 and outliers as shown on the geometry validation Tables will also be included. Each fit is shown from different orientation to approximate a three-dimensional view.

Electron density around DRG AaA 601:

$2mF_o$-DF_c (at 0.7 rmsd) in gray
mF_o-DF_c (at 3 rmsd) in purple (negative)
and green (positive)

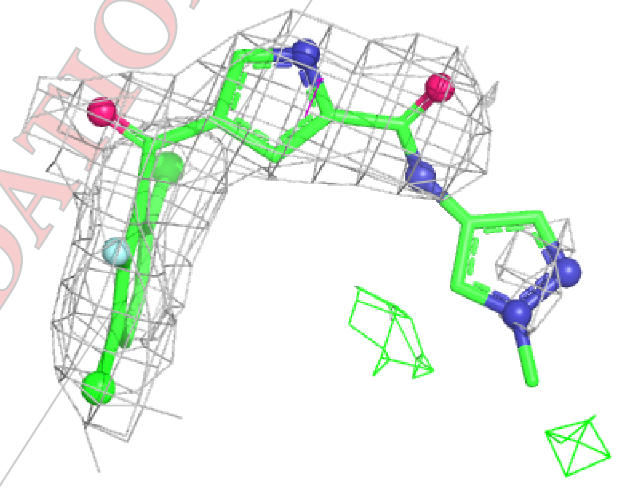

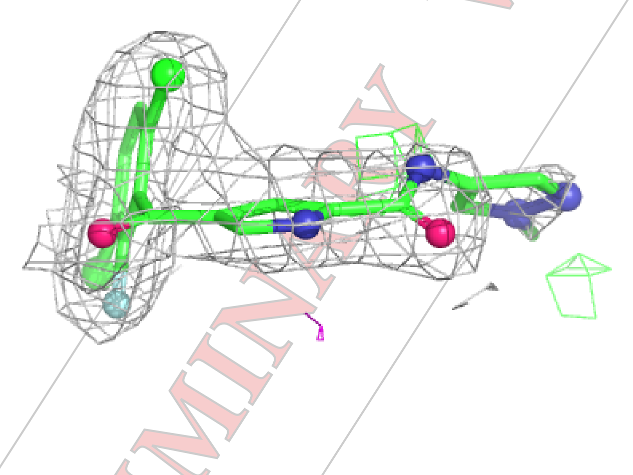

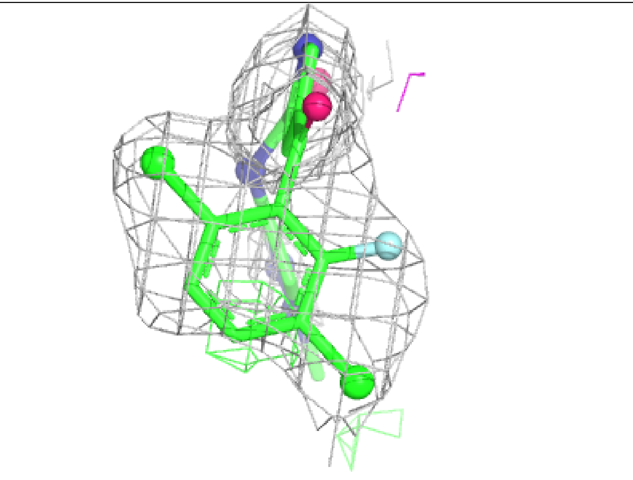

6.5 Other polymers (i)

There are no such residues in this entry.

PRELIMINARY VALIDATION REPORT
